# Murine Neonatal Oxidant Lung Injury: NRF2-Dependent Predisposition to Adulthood Respiratory Viral Infection and Protection by Maternal Antioxidant

**DOI:** 10.3390/antiox10121874

**Published:** 2021-11-24

**Authors:** Hye-Youn Cho, Laura Miller-DeGraff, Ligon A. Perrow, Wesley Gladwell, Vijayalakshmi Panduri, Fred B. Lih, Steven R. Kleeberger

**Affiliations:** 1 Immunity, Inflammation and Disease Laboratory, National Institute of Environmental Health Sciences, National Institutes of Health, Durham, NC 27709, USA; miller12@niehs.nih.gov (L.M.-D.); perrow@niehs.nih.gov (L.A.P.); gladwell@niehs.nih.gov (W.G.); kleeber1@niehs.nih.gov (S.R.K.); 2 Epigenetic and Stem Cell Biology Laboratory, National Institute of Environmental Health Sciences, National Institutes of Health, Durham, NC 27709, USA; panduriv@niehs.nih.gov; 3 Mass Spectrometry Research and Support Group, National Institute of Environmental Health Sciences, National Institutes of Health, Durham, NC 27709, USA; lih@niehs.nih.gov

**Keywords:** hyperoxia, bronchopulmonary dysplasia, lung, respiratory syncytial virus, sulforaphane, prenatal, microarray, neonate, mice

## Abstract

NRF2 protects against oxidant-associated airway disorders via cytoprotective gene induction. To examine if NRF2 is an important determinant of respiratory syncytial virus (RSV) susceptibility after neonate lung injury, *Nrf2*-deficient (*Nrf2*^−/−^) and wild-type (*Nrf2*^+/+^) mice neonatally exposed to hyperoxia were infected with RSV. To investigate the prenatal antioxidant effect on neonatal oxidative lung injury, time-pregnant *Nrf2*^−/−^ and *Nrf2*^+/+^ mice were given an oral NRF2 agonist (sulforaphane) on embryonic days 11.5–17.5, and offspring were exposed to hyperoxia. Bronchoalveolar lavage and histopathologic analyses determined lung injury. cDNA microarray analyses were performed on placenta and neonatal lungs. RSV-induced pulmonary inflammation, injury, oxidation, and virus load were heightened in hyperoxia-exposed mice, and injury was more severe in hyperoxia-susceptible *Nrf2*^−/−^ mice than in *Nrf2*^+/+^ mice. Maternal sulforaphane significantly alleviated hyperoxic lung injury in both neonate genotypes with more marked attenuation of severe neutrophilia, edema, oxidation, and alveolarization arrest in *Nrf2*^−/−^ mice. Prenatal sulforaphane altered different genes with similar defensive functions (e.g., inhibition of cell/perinatal death and inflammation, potentiation of angiogenesis/organ development) in both strains, indicating compensatory transcriptome changes in *Nrf2*^−/−^ mice. Conclusively, oxidative injury in underdeveloped lungs NRF2-dependently predisposed RSV susceptibility. In utero sulforaphane intervention suggested NRF2-dependent and -independent pulmonary protection mechanisms against early-life oxidant injury.

## 1. Introduction

Lungs of preterm infants born at 24–36 weeks of gestational age are in the saccular phase of lung development. Extensive developmental changes are represented by widening of distal airways for subsequent formation of alveoli, differentiation of type 1 and 2 alveolar cells, and thinning of the air–blood barrier [1]. Alveolarization in fetal and new-born lungs is critical as the type 2 cells not only produce surfactants but are also involved in innate immunity [2]. In addition, type 1 cells differentiated from type 2 pneumocytes are responsible for barrier function and gas exchange [3]. Bronchopulmonary dysplasia (BPD) is a common outcome of the very low birth weight premature infants who required mechanical ventilation and oxygen therapy for acute respiratory distress. It is one of the most frequent causes of chronic respiratory morbidity in survivors of premature birth [4,5]. BPD is characterized by failure of alveolarization leading to alveolar simplification and dysmorphic pulmonary vascularization that results from an imbalance between lung injury and lung repair processes, which interrupts normal lung development and leads to diffuse lung fibrosis [2,6]. Persistent lung impairment later in life leads to long-term negative pulmonary outcomes and BPD survivors often have functional abnormalities and increased risk for adverse respiratory symptoms (e.g., asthma, bronchiolitis, infectious airway diseases, sleep disorders) as young adolescents and adults [7,8,9,10]. Knowledge about BPD pathogenesis has markedly accumulated and advances in neonatal care has improved survival rate in recent years. However, the pathogenesis and molecular mechanisms that lead to lung damage in BPD are not completely understood and there are limited therapeutic options available for prevention and treatment of BPD, which have been only partly satisfactory [8].

Hyperoxia exposure to newborn rodents whose lungs are in the saccular phase has been a model for BPD because their lungs are structurally simplified and functionally affected similar to BPD phenotypes [11,12]. Long-term consequence of neonatally exposed hyperoxia including significantly altered pulmonary function, pulmonary hypertension, and shortened life span were found in rodents [13]. Exposure to early postnatal hyperoxia in mice was also reported to exacerbate cigarette smoke-induced obstructive pulmonary disorder and influenza and rhinovirus infection later in life [14,15,16].

Nuclear factor, erythroid derived 2, like 2 (NFE2L2, NRF2) is a transcriptional inducer of antioxidant response element (ARE)-bearing host defense genes. Studies with mice genetically deficient in *Nrf2* (*Nrf2*^−/−^) found that NRF2-mediated induction of antioxidants and various cytoprotective genes is essential in mitigation of inflammatory and oxidative airway diseases [17]. Lack of *Nrf2* exacerbated hyperoxia-induced BPD-like arrest in alveolarization evidenced by lower radial alveolar count and reduced appearance of multilobular alveoli/branched septi as well as severe exudative-phase diffuse alveolar damage including edema and inflammation in saccular stage lung [18,19]. Sulforaphane (4-methylsulfinylbutyl isothiocyanate, SFN) is a bioactive phytochemical antioxidant and one of its mechanisms is through NRF2 stabilization by inhibition of the cytoplasmic suppressor KEAP1 [20,21]. SFN was determined as a potential drug target that can perturb hyperoxia-induced transcriptome changes in mouse neonates [22]. We recently reported that orally administered SFN lessened hyperoxia-induced lung injury in adult mice in a NRF2-dependent manner [23]. In addition, SFN attenuated airway inflammation and injury against bacterial infection following emphysema, respiratory syncytial virus (RSV), and inhaled arsenic [17,23]. However, overall clinical efficacy of SFN is undetermined [24,25]. Furthermore, little is known about prenatal effects of NRF2 modulation or SFN in postnatal respiratory health.

RSV is a ubiquitous seasonal pathogen that may cause acute lower respiratory tract infection in children and infant hospitalization [26,27]. More recent studies have demonstrated increased risk of severe RSV disease among infants with BPD compared to those with no BPD [28,29]. Gestational age is one of the critical risk factors for infant RSV hospitalization rates and appearance of respiratory outcomes [30]. Palivizumab, the humanized RSV monoclonal antibody, has been the only approved option for RSV prophylaxis and it is indicated to reduce RSV-caused serious lower airway infection in high-risk premature infants within six months of the anticipated RSV season [31,32]. NRF2 and antioxidants played a protective role in RSV-induced upper and lower airway disorders and SFN inhibited lung viral replication and neutrophilic inflammation in mice [33,34].

The current study was designed to investigate whether (1) hyperoxia-induced injury in the developmental murine lung affects the later life RSV susceptibility, and (2) maternal supplementation of SFN protects newborn mice against hyperoxic lung injury. We also examined the effect of *Nrf2* deficiency in these two models using wild-type (*Nrf2*^+/+^) and *Nrf2*^−/−^ mice.

## 2. Materials and Methods

### 2.1. Animals and Time Mating

*Nrf2*^−/−^ (ICR.129P2-*Nfe2l2^tm1Mym^*) and *Nrf2*^+/+^ (Crl:CD1(ICR)) mice were generated [19] and maintained in the animal facility at the National Institute of Environmental Health Sciences (NIEHS). *Nrf2*^+/+^ and *Nrf2*^−/−^ mice were time mated in the NIEHS animal facility. Time-pregnant foster (Black Swiss) mice were purchased from Taconic Farms, Inc. (Hudson, NY, USA). All animals were housed in a virus- and antigen-free room. Water and mouse chow (modified AIN76A; Harlan Teklad, Madison, WI, USA) were provided ad libitum. All animal use was approved by the NIEHS Animal Care and Use Committee.

### 2.2. Gestational SFN Administration and Placenta Collection

Right lungs Time-pregnant *Nrf2*^+/+^ and *Nrf2*^−/−^ mice and time-pregnant foster dams received approximately 9 μmol (1.67 mg) of pure SFN (R-SFN, LKT Laboratories, Inc., St. Paul, MN, USA) in PBS or vehicle (PBS) by oral gavage (100 μL) on embryonic (E) days 11.5, 13.5, 15.5, and 17.5 based on regimens [23,35]. Placenta was collected on E18.5 following the procedure described previously [36]. At E18.5, a subset of *Nrf2*^+/+^ and *Nrf2*^−/−^ dams were euthanatized by carbon dioxide, the abdominal cavity opened, and the uterus and ovaries were removed intact. The uterus was spread out, extraembryonic membranes (amnion, yolk sac, chorion, allantois) from each embryo were removed and placenta was separated from embryo. The placenta was snap frozen and stored at −80 °C for later molecular analysis.

### 2.3. Neonate Hyperoxia Exposure

Two days before scheduled delivery, time-pregnant *Nrf2*^+/+^ and *Nrf2*^−/−^ mice were cohabitated with time-pregnant foster dams. Upon birth (PND0), neonatal mice from multiple litters with the same prenatal treatment or same genotype were pooled and randomly reassigned to exposure groups with a foster dam per group. The number of mice per cage was kept at a maximum of 12 and matched between comparison groups to control for the effects of the litter size on nutrition and growth. At 24 h after birth (PND1), neonatal mice were placed in cages an inserted into an inhalation chamber and exposed to hyperoxia (UHP grade, Min. purity 99.994% O_2_, National Welders, Durham, NC, USA) continuously for 2 or 3 days with their foster dams. During exposure, foster dams had free access to food (modified AIN76A; Harlan Teklad) and water. The temperature (72 ± 3°F) and humidity (50 ± 15%) of the chamber were monitored, and mice were exposed to a 12-h light–dark cycle. The entire volume of gas in the chamber was flushed via total system flow every 3–4 min to maintain O_2_ concentration consistent and to avoid CO_2_ accumulation. Neonatal mice and their foster dams assigned to room air controls were placed in cages placed on a countertop with food and water provided ad libitum for the same exposure duration. The hyperoxia chamber was opened briefly after 24 h exposure to replace foster dams and to check animal health (morbidity and mortality). For neonate hyperoxia end points in the prenatal SFN study, *Nrf2*^+/+^ and *Nrf2*^−/−^ pups with foster dams were removed from the chamber at the end of 3-day exposure to hyperoxia or room air and pups were euthanized by sodium pentobarbital overdose (0.02 mL per neonate). For the adulthood RSV study, *Nrf2*^+/+^ and *Nrf2*^−/−^ pups and their foster dams were removed from the chamber and foster dams were replaced after the end of 2-day exposure to hyperoxia or room air. *Nrf2*^+/+^ and *Nrf2*^−/−^ pups with their foster dam were kept in a virus- and antigen-free room. After weaning at PND14, *Nrf2*^+/+^ and *Nrf2*^−/−^ mice were housed individually with water and mouse chow (modified AIN76A) until RSV infection at 5 wk of age.

### 2.4. RSV Infection

Neonatal room air- or hyperoxia-exposed mice at 5 wk of age were intranasally (IN) instilled with human RSV19 (ViraSource, Durham, NC, USA; 10^6^ plaque forming unit/mouse in 50 μL HBSS) or vehicle (Hep-2 cell extracts in 50 μL HBSS). After 1 and 5 d post-IN, mice were euthanized with sodium pentobarbital overdose (104 mg/kg).

### 2.5. Bronchoalveolar Lavage (BAL) Analyses

Whole lungs of each neonate from prenatal treatment groups were lavaged in situ, four consecutive times with Hank’s balanced salt solution (HBSS, 0.08 mL/2.5 g body weight) using a 24-gauge BD Angiocath cannula (BD Biosciences, San Diego, CA) as previously described in detail [19]. The pooled BAL fluid returns were centrifuged (1000× *g*, 10 min at 4 °C). Aliquots of lavage supernatants (50 μL) were analyzed for total protein concentration (a marker of hyperpermeability/edema) in Bradford reagent (Bio-Rad, Hercules, PA, USA) following the manufacturer’s procedure. Cell pellets from lavage returns were resuspended in 1 mL HBSS and 10 μL aliquots were counted for total cells using a hemocytometer. Aliquots of cell suspension (150 μL) were cytocentrifuged and stained with Wright–Giemsa for differential epithelial and inflammatory cell counts. For young adult mice in RSV study groups, the right lung of each adult mouse was lavaged in situ four consecutive times with HBSS (0.5 mL/25 g b.w.) as indicated elsewhere [37]. Briefly, The BAL fluid returns were centrifuged and an aliquot of the 1st BAL return supernatants (50 μL) and aliquots of pooled cell pellet suspension were analyzed for total protein concentration and cell differentials, respectively, as described above. Lactate dehydrogenase (LDH) content was evaluated in aliquots of pooled (neonates, 50 μL) or 1st (adults, 25 μL) BAL return fluids by a colorimetric assay (Sigma-Aldrich, St. Louis, MO, USA) as a cell lysis and cytotoxicity marker.

### 2.6. Lung Lipid Oxidation Measurement

Lung lipid oxidation levels were measured in BAL fluids by estimation of released malondialdehyde (MDA) which forms 1:2 adduct with added thiobarbituric acid (TBA) in acidic condition. Briefly, aliquots of BAL fluid (75 μL) were treated with acid reagent (75 μL) at room temperature for 15 min and centrifuged (14,000× *g*, 4 min). Supernatants were incubated with TBA reagent at 47 °C for 2 h. MDA-TBA adducts formation indicated by color changes was determined spectrophotometrically at 532 nm and pre-incubation absorbance at 532 nm was subtracted. The amount of MDA in BAL fluids was quantified from a standard curve (TBARS Parameter Assay; R&D Systems, Minneapolis, MN, USA) that was run in parallel.

### 2.7. Sandwich Enzyme-Linked Immunosorbent Assay (ELISA)

The quantity of chemokine (C-X-C motif) ligand 1 (CXCL1/KC) and interleukin (IL)-1β were determined in BAL fluid (50 μL) using colorimetric mouse-specific ELISA kits following the manufacturer’s instructions (R&D Systems). To quantify neutrophil abundance in BAL samples (25 μL), neutrophil myeloperoxidase (MPO) level was determined using a mouse specific ELISA kit following the manufacturer’s directions (R&D Systems).

### 2.8. Lung Histopathology

Whole lungs of neonate mice were inflated intratracheally in situ with 10% neutral buffered formalin (NBF) and the trachea was ligated. The inflated lung was removed from the mouse and fixed in a contained filled with 10% NBF for 2 days. The fixed left lung was trimmed into two equal sections and tissue blocks were embedded in paraffin and cut into 4 μm thickness for hematoxylin and eosin (H&E) staining. The left lung from each adult mouse was inflated intratracheally in situ with 10% NBF and fixed following the routine procedure described elsewhere [38]. The fixed lung lobe was cut at proximal (around generation 5) and distal (around generation 11) levels of the main axial airway [39], and paraffin-embedded tissue blocks were cut into 5 μm-thickness to be stained with H&E and Masson’s trichrome.

### 2.9. Serum Immunoglobulin E (IgE) Measurement

An aliquot of serum (1 μL) from each mouse blood sample was collected by cardiac puncture and diluted in an assay diluent (1:100). IgE levels were colorimetically determined using a mouse-specific sandwich ELISA kit (BD Opt EIA). Similarly prepared serial dilution of IgE standards (BD Biosciences) were used for IgE quantification.

### 2.10. SFN Metabolites Detection in Urine and Milk Bands

Non-metabolized (unconjugated) SFN and major conjugated metabolites, including SFN-GSH, SFN-cysteine, and SFN-NAC, in mouse urine and milk bands were analyzed by HPLC-mass spectrometry as previously described [23]. That is, an aliquot (10 μL) of pooled urine from neonates (*n* = 8–13/group) and foster dams (*n* = 2 for PBS/Air, *n* = 2 for SFN/Air) was diluted with 90 μL 5% methanol and 0.1% formic acid. The sample was centrifuged at 8 °C and 80 μL of supernatant was transferred to an autosampler vial containing a low volume insert. Milk bands were weighed and homogenized in 300 μL of 0.1% formic acid using a bead mill. A 30 mg of milk homogenate was diluted to 500 μL with 0.1% formic acid and centrifuged at 8 °C. The supernatant was loaded onto Oasis HLB solid phase extraction cartridges. Cartridges were then washed with 1 mL of 0.1% formic acid, dried with a stream of nitrogen, washed with 1 mL hexane to remove neutral lipids, and eluted with 2 mL of 9:1 acetonitrile:methanol containing 0.1% formic acid. Eluted milk band samples were dried under vacuum then reconstituted in 50 μL 5% methanol/0.1% formic acid. An aliquot (10 μL) of prepared urine or milk sample was injected onto an ACE Excel 3 CN-ES 2.1 × 100 mm HPLC column with gradient separation at a flow rate of 250 μL/min. Electrospray ionization and selected reaction monitoring mass spectrometry were used for analyte quantitation.

### 2.11. Protein Extraction and Detection by Western Blot Analysis

Right lung or placenta tissues (*n* = 3/group) were homogenized in RIPA buffer containing PMSF (10 μg/mL) and protease/phosphatase inhibitor cocktail (Sigma-Aldrich, St. Louis, MO, USA) and total proteins were isolated by centrifugation. Snap-frozen neonate lung tissues were pulverized in dry ice (two pooled sample/group, four lungs/sample) for nuclear proteins isolation using a kit following the manufacturer’s direction (Active Motif, Carlsbad, CA, USA). Quantified proteins were aliquoted and stored at −80 °C until used. Lung nuclear (7 μg) or pooled total placenta (50 μg) proteins were subjected to Western blotting using antibodies against mouse c-Fos (Santa Cruz Biotechnology, Inc., Dallas, TX, USA), lamin B (Santa Cruz, Dallas, TX, USA), phosphor-p42/p44 mitogen-activated protein kinase/extracellular signal-regulated kinase (*P*-p42/p44 MAPK/*P*-ERK, Santa Cruz) and pan-actin (Santa Cruz). The assay was done in duplicate and scanned band images were quantitated by densitometry using Image J Gel Analysis software and relative protein band intensities normalize to PBS/air-*Nrf2*^+/+^ (lung c-Fos) or PBS-*Nrf2*^+/+^ (placenta *P*-ERK) were depicted.

### 2.12. Protein Oxidation Detection

Protein carbonyl groups introduced into protein side chains by oxidative reactions were quantified in lung total protein aliquots by a colorimetric analysis. Briefly, an aliquot of total lung protein (1 μg in 100 μL volume) and oxidized/reduced BSA standards were adsorbed onto a 96-well plate (OxiSelect Protein Carbonyl ELISA kit; Cell Biolabs, Inc., San Diego, CA, USA) overnight at 4 °C, and protein carbonyls were derivatized by 2.4-dinitrophenyl hydrazine (DNPH). The DNPH-derivatized protein samples were then incubated with an anti-DNP antibody and an HRP-conjugated secondary antibody in turn following the manufacturer’s instructions. The protein carbonyl contents indicating oxidated protein level were determined by colorimetric analysis at 450 nm using a reduced and oxidized BSA standard curve.

### 2.13. Nuclear DNA Binding Assay

Nuclear factor kappa-light-chain-enhancer of activated B cells (NF-κB) P65-DNA binding activity was quantitated using a transcription factor binding assay kit (Abcam, Cambridge, MA, USA). Briefly, nuclear protein aliquots (2 μg, duplicates) were added to ELISA plate wells coated with NF-κB consensus dsDNA sequence and incubated overnight at 4 °C. The wells were then washed and added with NF-κB p65 primary antibody for incubation. After consecutive incubation with secondary antibody and detection reagent, colorimetric analysis at 450 nm determined DNA-bound NF-κB p65 amount.

### 2.14. Lung DNA Damage Measurement

Quantitative polymerase chain reaction (PCR) was used to determine the presence of genomic or mitochondrial DNA lesions (including base modifications, AP sites, and strand breaks) which blocks the progression of DNA polymerase so that only undamaged templates can be amplified [40]. Briefly, total cellular DNA was isolated from frozen lungs using a kit (Qiagen Inc., Valencia, CA, USA), and a fragment of DNA from either nuclei (6.5 kb of DNA polymerase β) or mitochondria (117-bp fragment and 10-kb fragment) were amplified in ABI Prism 7700 Sequence Detection System (Applied Biosystems by Life Technologies, Foster City, CA, USA) using specific primer sets [19]. The amount of DNA amplification is inversely proportional to the amount of DNA damage such that increasing damage causes a decrease in amplification (1, 10). Amplification (as determined by picogreen fluorescence) of hyperoxia-treated samples was compared with that of undamaged controls to calculate the relative amplification. Relative amplification values were then used to calculate the average number of lesions per 10 kb of the genomic or mitochondrial DNA, using a Poisson distribution. The amount of damage is presented as ln (mean of hyperoxia amplification/mean of the control samples). The amount of long mitochondrial amplification product (10 kb) was normalized to the mitochondrial DNA copy number using the small 117-bp fragment.

### 2.15. Lung RNA Isolation and Reverse Transcriptase-Polymerase Chain Reaction (RT-PCR)

Total RNA was isolated from left lung or placenta homogenates (RNeasy Mini Kit, Qiagen Inc., Dusseldorf, Germany). One μg of RNA was reverse transcribed into cDNAs using GeneAmp PCR System 9700 (Applied Biosystems) and an aliquot of cDNA (40 ng) was added to the reaction for semi-quantitative PCR (1 kb *Nrf2* amplicon) as previously described [33] or quantitative PCR in 25 μL reaction containing 12.5 μL 2X Power SYBR Green Master Mix (Applied Biosystems, Foster City, CA, USA) and 240 nM of custom-designed [41] or commercially available (Real Time Primers, LLC, Elkins Park, PA, USA) primers for mouse *Nrf2*, glutamate-cysteine ligase catalytic subunit (*Gclc*), glutathione-s-transferase P1 (*Gstp1*), *Gstt1*, NAD(P)H quinone dehydrogenase (quinone) 1 (*Nqo1*), ADAM like decysin 1 (*Adamdec1*), and ubiquitin C-terminal hydrolase L1 (*Uchl1*) by 10 min hold at 95 °C and up to 45 cycles of 95 °C (15 s)–60 °C (1 min) using an ABI Prism 7700 Sequence Detection System (Applied Biosystems) or CFX Connect Realtime System (Bio-Rad). The target gene expression was calculated using the comparative threshold cycle (C_T_) method from the fluorescence detected C_T_ difference between18s rRNA and target gene (ΔC_T_) in the same sample.

### 2.16. cDNA Microarray Analyses 

Total lung RNA (100 ng, *n* = 3/group) from placenta (E18.5) or neonate lung (PND4) was applied to mouse 430 2.0 arrays (Affymetrix, Inc., Santa Clara, CA, USA) in the NIEHS Microarray Core Facility as described previously [19]. Array data were analyzed statistically using GeneSpring software (Agilent Technologies, Inc., Santa Clara, CA, USA). Briefly, array raw data were filtered by lower expression percentile (at least 1 sample had values within 20% cut-off rage) and the expression levels were normalized to the mean value of the experimental control (lung-PBS/Air/*Nrf2*^+/+^, placenta-PBS/*Nrf2*^+/+^) for each gene by quantile algorithm. Student’s *t*-test (PBS vs. SFN in each genotype for placenta or lung) or two-way ANOVA (PBS vs SFN and air vs. hyperoxia in each genotype lung) identified differentially expressed genes in *Nrf2*^+/+^ or in *Nrf2*^−/−^ mice using GeneSpring (Agilent Technologies, Inc., Santa Clara, CA, USA). Overlapping or unique genes in both genotypes were determined by Venn Diagram analyses. Potential molecular interactions, biological functions, downstream and upstream pathways were determined by Ingenuity Pathway Analysis (IPA, Qiagen). Microarray data are deposited in Gene Expression Omnibus (accession numbers: GSE164699 for the neonate lungs and GSE164700 for the placenta).

### 2.17. Statistics

Data are presented as the group mean ± standard error of the mean (S.E.M.). The effects of neonate inhalation exposure/adulthood intranasal treatment (air/vehicle, air/RSV, O_2_/vehicle, or O_2_/RSV) and genotype (*Nrf2*^+/+^, *Nrf2*^−/−^) in adulthood RSV study or the effects of prenatal treatment/neonate exposure (PBS/air, PBS/O_2_, SFN/air, SFN/O_2_) and genotype (*Nrf2*^+/+^, *Nrf2*^−/−^) in gestational SFN study was evaluated by two-way ANOVA. *Nrf2* mRNA assessment in *Nrf2*^+/+^ mice was done by One-way ANOVA. Student–Newman–Keuls test was used for a posteriori comparisons of means (*p* < 0.05). SigmaPlot 13.0 or 14.0 package (Systat Software, San Jose, CA, USA) was used for all statistical analyses.

## 3. Results

### 3.1. Effect of Neonatal Hyperoxia on Adulthood RSV Pathogenesis

#### 3.1.1. NRF2-Dependent Augmentation of Lung Inflammation and Injury

**Figure 1:**
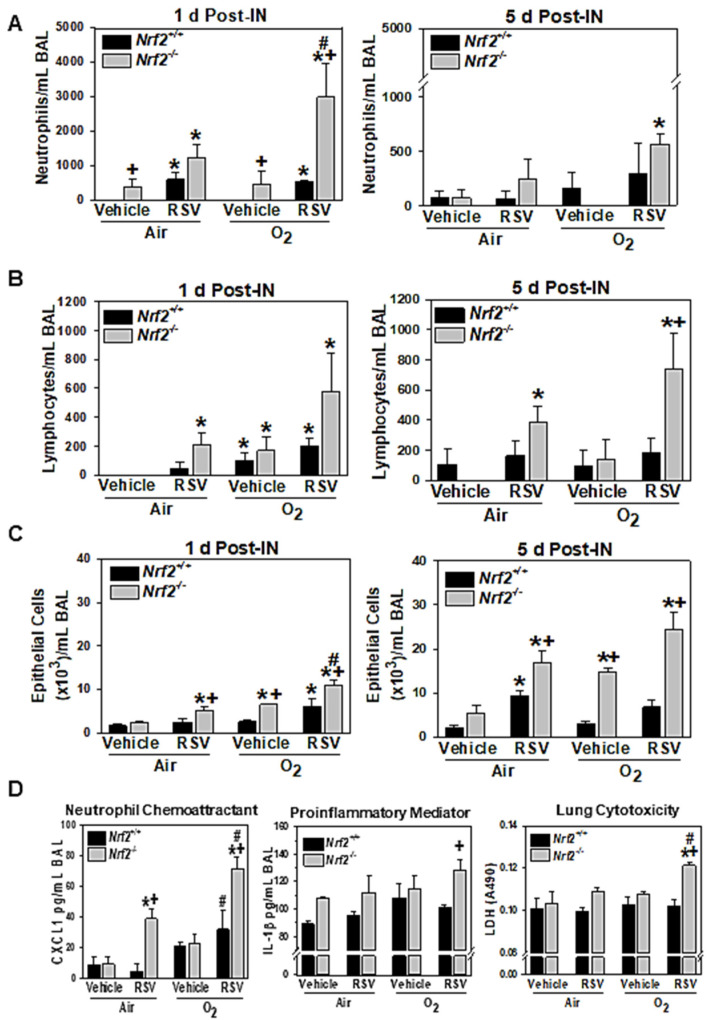
Differential lung inflammation and injury in response to respiratory syncytial virus (RSV) infection following neonatal hyperoxia (O_2_) exposure in wild-type (*Nrf2*^+/+^) and *Nrf2*-deficient (*Nrf2*^−/−^) mice. Lung injury phenotypes were assessed by the number of neutrophils (**A**), lymphocytes (**B**), and epithelial cells (**C**) in bronchoalveolar lavage (BAL) fluids at 1 d and 5 d post-intranasal (IN) instillation of vehicle or RSV. Data presented as group mean ± S.E.M (*n* = 3–5/group). (**D**) A neutrophil chemoattractant, *chemokine* (C-X-C motif) ligand 1 (CXCL1), a proinflammatory mediator interleukin (IL)-1β, and a lung injury and cytotoxicity marker lactate dehydrogenase (LDH) were determined in BAL fluids at 1 d post-IN. Group mean ± S.E.M. (*n* = 3–4/group) presented. *, significantly different from genotype-matched air/vehicle controls (*p* < 0.05). +, significantly different from exposure/intranasal treatment matched *Nrf2*^+/+^ mice (*p* < 0.05). #, significantly different from genotype-matched, intranasal treatment-matched air (*p* < 0.05).

Significantly greater RSV-induced neutrophilic inflammation (1 d post-IN) was found in *Nrf2*^−/−^ mice neonatally exposed to O_2_ than in those neonatally exposed to air, and the heightened neutrophilic infiltration was remained elevated in neonatal O_2_-exposed *Nrf2*^−/−^ mice at 5 d (Figure 1A). Vehicle treatment also caused significant increase of neutrophils in neonatal air- or O_2_-exposed *Nrf2*^−/−^ mice (Figure 1A). In *Nrf2*^+/+^ mice, there was no significant additive effect of neonatal O_2_ exposure in RSV-induced neutrophilic inflammation (Figure 1A). RSV infection caused significant lymphocyte infiltration in *Nrf2*^−/−^ mice among mice pre-exposed to air at 1 d (Figure 1B). Neonatal O_2_ exposure significantly potentiated lymphocytic inflammation in both *Nrf2*^+/+^ and *Nrf2*^−/−^ mice after either vehicle or RSV at 1 d (Figure 1B). RSV-induced lymphocytic inflammation remained elevated by 5 d in *Nrf2*^−/−^ mice exposed to either air or O_2_, while there was no significant exacerbation of lymphocytic infiltration by previous O_2_ exposure in either genotype of mice at this time (Figure 1B). Significant RSV-induced epithelial cell sloughing was found in neonatal air-exposed *Nrf2*^+/+^ (5 d) and *Nrf2*^−/−^ (1 and 5d) mice (Figure 1C). BAL epithelial cell number was significantly abundant in *Nrf2*^−/−^ mice than in *Nrf2*^+/+^ mice either exposed to prenatal air or O_2_ and RSV-induced epithelial injury was exacerbated by neonatally exposed O_2_ in *Nrf2*^+/+^ mice at 1 d (Figure 1C). Consistent with neutrophil numbers, neutrophil chemoattractant CXCL1 level at 1 d was significantly increased by neonate O_2_ relative to neonate air in both genotypes while the augmentation by O_2_ was more significant in *Nrf2*^−/−^ mice than in *Nrf2*^+/+^ mice (Figure 1D). Proinflammatory cytokine IL-1β contents in BAL were also significantly greater in O_2_/RSV-*Nrf2*^−/−^ mice compared to O_2_/RSV-*Nrf2*^+/+^ mice at 1 d (Figure 1D). BAL LDH levels reflected significantly enhanced lung tissue damage and epithelial cell death in neonatal O_2_-exposed, RSV-infected *Nrf2*^−/−^ mice (Figure 1D).

#### 3.1.2. NRF2-Dependent Augmentation of Lung Histopathology

**Figure 2:**
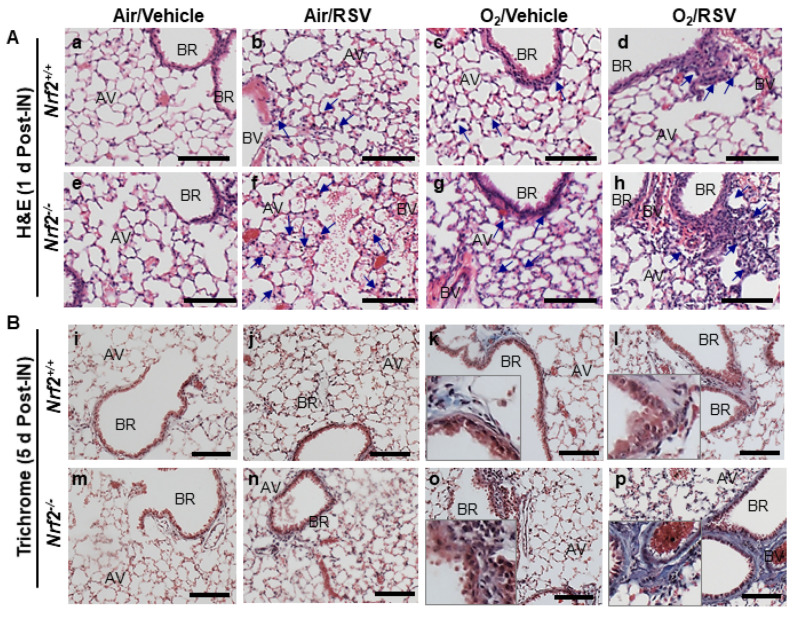
Differential pulmonary histopathology after respiratory syncytial virus (RSV) infection following neonatal hyperoxia (O_2_) exposure in wild-type (*Nrf2*^+/+^) and *Nrf2*-deficient (*Nrf2*^−/−^) mice. Formalin-fixed lung tissue sections were processed and stained with H&E at 1 d post-intranasal (IN) instillation (**A**) and Masson’s trichrome (**B**) at 5 d post-IN instillation of vehicle or RSV in mice neonatally exposed to O_2_ or room air. BR = bronchi or bronchiole; AV = alveoli; BV = blood vessel. Arrows = injured regions (inflammation, alveolar thickening and vacuolization, bronchial epithelium/smooth muscle thickening). Blue in trichrome staining = collagen accumulation in fibrogenic regions (higher magnifications in insets). Bars = 100 μm.

H&E (Figure 2A) and Masson’s trichrome (Figure 2B) staining showed that RSV infection caused more severe alveolar inflammation and small bronchial smooth muscle thickening in neonatal air-exposed *Nrf2*^−/−^ mice (Figure 2A(f),B(n)) than in neonatal air-exposed *Nrf2*^+/+^ mice at 1 d post-IN (Figure 2A(b),B(j)). Neonatal O_2_ exposure augmented airway inflammatory cell accumulation and bronchial smooth muscle thickening in both strains but more severely in *Nrf2*^−/−^ mice (Figure 2A(h),B(p)) than in *Nrf2*^+/+^ mice (Figure 2A(d),B(l)). Slightly thickened alveolar septum, alveolar vacuolization, and moderate alveolar and perivascular–peribronchiolar fibroproliferation with collagen accumulation (Figure 2A(c),B(k)) were found in O_2_/vehicle-*Nrf2*^+/+^ mice compared to air/vehicle-*Nrf2*^+/+^ mice (Figure 2A(a),B(i)). These histologic features in O_2_/vehicle-*Nrf2*^+/+^ mice were more predominant in O_2_/vehicle-*Nrf2*^−/−^ mice (Figure 2A(g),B(o)). RSV-induced exacerbation of collagen accumulation in alveoli as well as perivascular–peribronchiolar region was marked in O_2_/RSV-*Nrf2*^−/−^ mice at 5 d post-IN (Figure 2B(p)).

#### 3.1.3. NRF2-Dependent Potentiation of Lung Viral Replication

**Figure 3:**
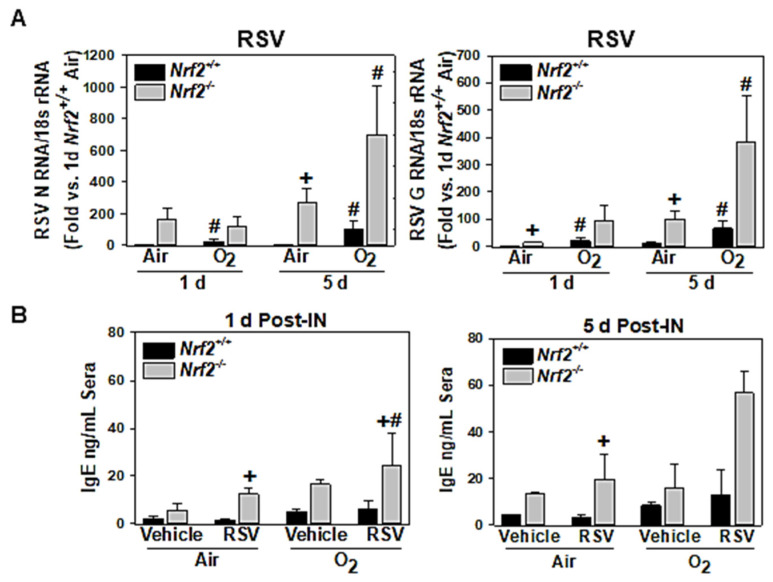
Differential lung respiratory syncytial virus (RSV) expression and serum IgE level in wild-type (*Nrf2*^+/+^) and Nrf2-deficient (*Nrf2*^−/−^) mice. (**A**) qRT-PCR determined relative pulmonary RSV N and G gene expressions compared to the level of air/RSV-*Nrf2*^+/+^ mice at 1 d post-intranasal (IN) instillation. (**B**) Serum IgE concentration was determined by mouse-specific ELISA. Mean ± S.E.M. (*n* = 3/group) presented. +, significantly different from exposure/treatment-matched *Nrf2*^+/+^ mice (*p* < 0.05). #, significantly different from genotype-matched, air/RSV (*p* < 0.05). O_2_ = hyperoxia.

Compared to air/RSV-*Nrf2*^+/+^ mice, RSV N and G gene expressions were significantly higher in air/RSV-*Nrf2*^−/−^ mice at 1 d (G gene) and 5 d (N and G genes) (Figure 3A). Neonatal O_2_ exposure significantly potentiated RSV gene replication in *Nrf2*^+/+^ (1 d and 5 d) and *Nrf2*^−/−^ (5 d) mice (Figure 3A). O_2_-augmented RSV gene replication was higher, but not statistically significant, in *Nrf2*^−/−^ mice compared to *Nrf2*^+/+^ mice (Figure 3A).

#### 3.1.4. NRF2-Dependent Potentiation of Serum IgE Increase

Serum IgE level detected as a marker for RSV-induced bronchial epithelial hyperplasia/smooth muscle thickening was significantly greater in *Nrf2*^−/−^ mice exposed to neonatal air compared with neonatal air/vehicle-*Nrf2*^−/−^ mice at 1 d and 5 d post-RSV (Figure 3B). Neonatal O_2_ exposure further heightened circulating IgE level in *Nrf2*^−/−^ mice at 1 d post-RSV, which was not evident in *Nrf2*^+/+^ mice (Figure 3B).

#### 3.1.5. NRF2-Dependent Augmentation of Lung Oxidative Stress

**Figure 4:**
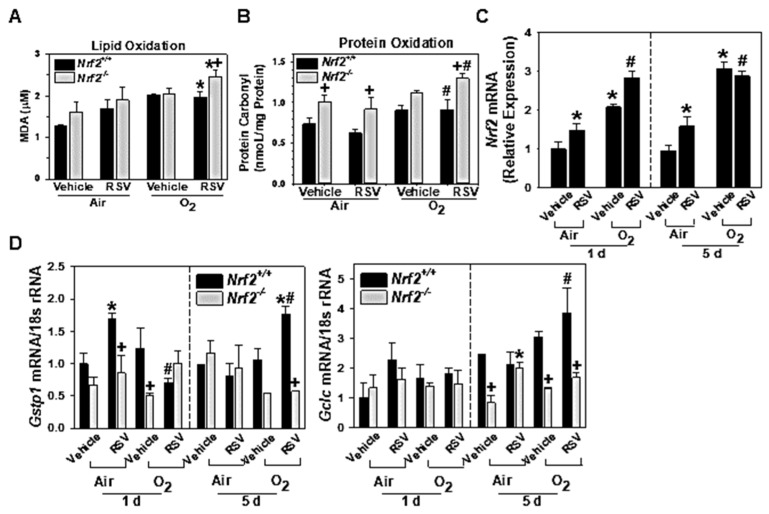
Differential oxidative damage markers and antioxidant expression in wild-type (*Nrf2*^+/+^) and *Nrf2*-deficient (*Nrf2*^−/−^) mice. (**A**) Lung lipid oxidation determined by malondialdehyde (MDA) level in 50 μL aliquots of bronchoalveolar lavage (BAL) fluids from each mouse (*n* = 3–4/group). (**B**) Oxidized protein level in the lung determined by protein carbonyl amount in 1 μg of total protein (*n* = 3/group). (**C**) Lung *Nrf2* mRNA expression determined by semi-quantitative RT-PCR (*n* = 3/group). (**D**) Lung expressions of NRF2-dependent antioxidant enzyme genes, glutathione-s-transferase P1 (*Gstp1*) and glutamate-cysteine ligase catalytic subunit (*Gclc*), were determined by quantitative RT-PCR (*n* = 3/group). Mean ± S.E.M. presented. *, significantly different from genotype matched air/vehicle controls (*p* < 0.05). +, significantly different from similarly treated and -exposed *Nrf2*^+/+^ mice (*p* < 0.05). #, significantly different from genotype-matched air/RSV mice (*p* < 0.05). O_2_ = Hyperoxia.

Oxidation levels of endogenous macromolecules, proteins, and lipids, were measured to estimate the lung oxidative stress at 5 d post-IN. RSV infection significantly increased BAL oxidized lipid contents (determined as MDA levels) only in neonatal O_2_-exposed groups, and it was significantly more increased in O_2_/RSV-*Nrf2*^−/−^ mice than in O_2_/RSV-*Nrf2*^+/+^ mice (Figure 4A). The amount of oxidized protein carbonyl was significantly higher basally (air/vehicle) in *Nrf2*^−/−^ mice than in *Nrf2*^+/+^ mice (Figure 4B). Relative to O_2_/vehicle groups, oxidized protein level in O_2_/RSV groups was significantly greater in both *Nrf2*^−/−^ and *Nrf2*^+/+^ mice and it was significantly more potentiated in O_2_/RSV-*Nrf2*^−/−^ mice than in O_2_/RSV-*Nrf2*^+/+^ mice (Figure 4B).

#### 3.1.6. Lung Nrf2 and ARE-Bearing Antioxidant Enzyme Expression

Pulmonary *Nrf2* mRNA abundance was significantly increased by RSV infection at 1 d and 5 d in air-exposed *Nrf2*^+/+^ mice compared to time-matched air/vehicle-*Nrf2*^+/+^ mice (Figure 4C). Neonatal exposure to O_2_ also significantly upregulated the lung *Nrf2* mRNA expression compared to air/vehicle at both time points (Figure 4C). Compared to air/RSV-*Nrf2*^+/+^ mice, *Nrf2* message was significantly heightened in O_2_/RSV-*Nrf2*^+/+^ mice (Figure 4C). RSV infection significantly induced pulmonary *Gstp1* expression at 1 d (in air group) and 5 d (in O_2_ group) in *Nrf2*^+/+^ mice (Figure 4D). *Gstp1* and *Gclc* mRNA levels were significantly enhanced in O_2_/RSV-*Nrf2*^+/+^ mice than in air/RSV-*Nrf2*^+/+^ mice at 5 d (Figure 4D). Abundance of these antioxidant enzyme genes was relatively or significantly lower in most experimental groups of *Nrf2*^−/−^ mice compared to time- and exposure-matched *Nrf2*^+/+^ mice (Figure 4D).

### 3.2. Effect of Prenatal SFN on Neonatal Hyperoxia-Induced Lung Injury

#### 3.2.1. Decreased Lung Injury in Neonates Supplemented with Prenatal SFN

**Figure 5:**
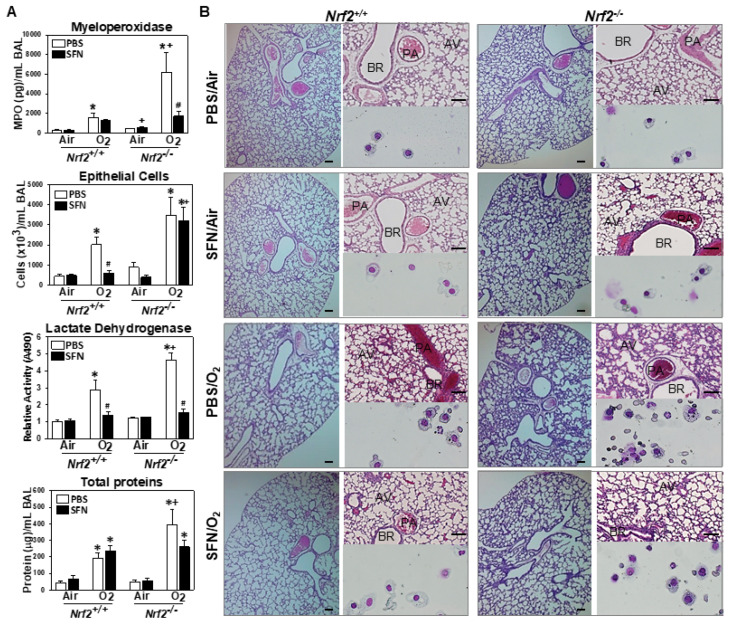
Effect of prenatal maternal sulforaphane on hyperoxia (O_2_)-induced lung injury in mouse neonates. (**A**) Lung injury was assessed by neutrophil myeloperoxidase (MPO) concentration, epithelial cell numbers, lactate dehydrogenase (LDH) level, and total protein concentration in bronchoalveolar lavage (BAL) fluids after 3-day exposure to O_2_ or room air exposure (PND1-PND4) to newborn mice given prenatal sulforaphane (SFN) or PBS. Group mean ± S.E.M presented. (*n* = 6–13/group). *, vs. genotype-matched PBS/air (*p* < 0.05). +, vs. prenatal treatment/exposure matched *Nrf2*^+/+^ (*p* < 0.05). #, vs. genotype matched O_2_/PBS (*p* < 0.05). (**B**) H&E-stained lung sections (lower and higher magnification light micrographs) and Giemsa-stained cytocentrifuged BAL cells (bottom right) depicted gestational SFN effects on neonatal lung injury by hyperoxia. BR = bronchi or bronchiole. AV = alveoli. PA = pulmonary artery. Bars = 50 μm.

Hyperoxia-induced lung injury determined by BAL neutrophil MPO concentration (a marker of neutrophilia), epithelial cell injury (a marker of epithelial sloughing), LDH level (a marker of lung damage and cytotoxicity), and total protein (a marker of edema and hyperpermeability) were relatively mild in *Nrf2*^+/+^ neonates compared to *Nrf2*^−/−^ neonates (Figure 5A). Significantly reduced epithelial cell number and LDH were found in hyperoxia-exposed *Nrf2*^+/+^ neonates from gestational SFN supplemented dams (Figure 5A). In *Nrf2*^−/−^ pups, prenatal SFN significantly reduced hyperoxia-increased MPO and LDH levels (Figure 5A). There was no significant effect of prenatal SFN on hyperoxia-induced lung edema in either genotype while it was significantly severe in O_2_/PBS-*Nrf2*^−/−^ pups compared to O_2_/PBS-*Nrf2*^+/+^ pups (Figure 5A). In *Nrf2*^+/+^ lungs, neonatal hyperoxia-caused alveolar hypotrophy accompanying focal cellular proliferation was lessened in the SFN-treated group relative to the PBS-treated group (Figure 5B). Decreased BAL cells lysis and infiltrated leukocytes were found in SFN/O_2_-*Nrf2*^+/+^ than in PBS/O_2_-*Nrf2*^+/+^ as shown by Giemsa staining (Figure 5B). In PBS-treated *Nrf2*^−/−^ neonates, hyperoxia caused severe protein exudates and alveolar thickening indicating arrested saccular-to-alveolar transition (Figure 5B) as we reported previously [19]. Prenatal SFN markedly lessened these histopathologic phenotypes in *Nrf2*^−/−^ neonates (Figure 5B). We also found mitigation of inflammatory cell and congestion (filling of the alveoli with blood) in SFN/ O_2_-*Nrf2*^−/−^ neonates than in PBS/O_2_-*Nrf2*^−/−^ neonates (BAL cell cytospins, Figure 5B).

#### 3.2.2. Detection of Gestational SFN Metabolites in Urine and Milk Bands

**Figure 6:**
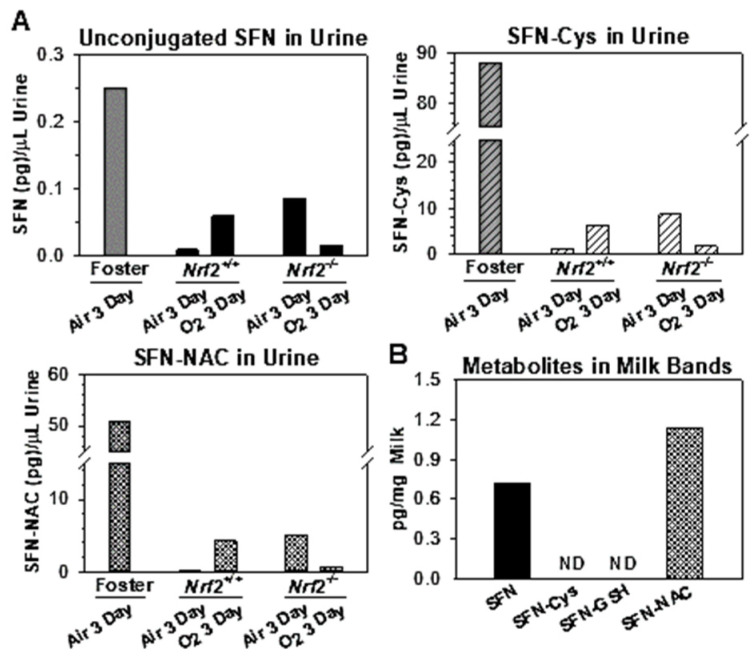
Sulforaphane (SFN) and metabolites detection. HPLC-mass spectrometry (ACE Excel 3 CN-ES 2.1 × 100 mm) determined SFN and its three major metabolites, SFN-N-acetyl cysteine (NAC), SFN-cysteine (Cys), and SFN-glutathione (GSH), in pooled neonate (*n* = 8–13/group) and foster dam (Black Swiss; *n* = 2 of SFN/Air) urine diluents or in deproteinated pooled milk band extracts (*n* = 2 of SFN/air-*Nrf2*^−/−^). Specimens were collected at PND4 after four oral doses of SFN (9.4 μmol at E11.5, 13.5, 15.5, 17.5) to foster dams. Samples were run three times and mean values are presented. ND = not detected. O_2_ = hyperoxia. Urine samples from gestational PBS treated foster dams and milk bands from their pups showed no metabolites detected.

HPLC-mass spectrometry detected SFN supplemented during gestation and its conjugated metabolites in milk bands (SFN-NAC, SFN) and urine (SFN-Cys, SFN-NAC, SFN) 8 d (PND4) after the last oral administration to foster dams (i.e., E17.5). Primary metabolites were SFN-Cys and SFN-NAC in neonate and foster urine samples, and unconjugated SFN was minimally detected (Figure 6A). In neonate milk bands, only unconjugated SFN and SFN-NAC were detected at PND4 (Figure 6B). Specimens of neonates from dams or dams given prenatal PBS had no SFN or its conjugates (SFN-Cys, SFN-GSH, SFN-NAC) detected. Metabolite levels or composition was not affected by genotype or exposure.

#### 3.2.3. Decreased Oxidative Stress and Antioxidant Expression in Neonates by Prenatal SFN

**Figure 7:**
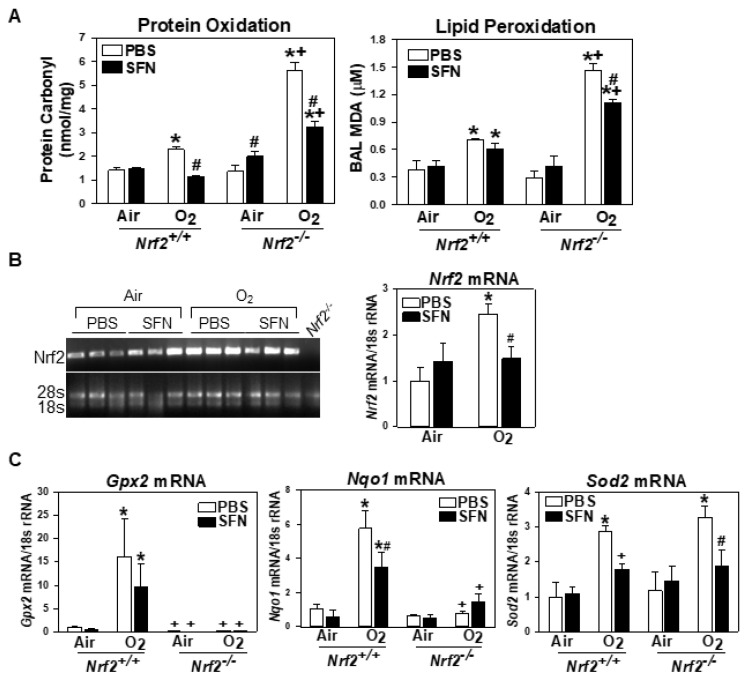
Oxidative damage markers and antioxidant expressions in wild-type (*Nrf2*^+/+^) and *Nrf2*-deficient (*Nrf2*^−/−^) mice. (**A**) Lung lipid oxidation determined by released malondialdehyde (MDA) level in 75 μL aliquots of bronchoalveolar lavage (BAL) fluids from mouse (*n* = 4/group). Oxidized protein level in the lung determined by protein carbonyl amount in 1 μg of total protein (*n* = 3/group). (**B**) Lung *Nrf2* mRNA expression determined by semi-quantitative (1 kb amplicons) and quantitative RT-PCR (*n* = 3/group). (**C**) Lung expressions of NRF2-dependent antioxidant enzyme genes, glutathione peroxidase 2 (*Gpx2*) and NAD(P)H dehydrogenase (quinone) 1 (*Nqo1*), and classical antioxidant superoxide dismutase 2 (*Sod2*) were determined by quantitative RT-PCR (*n* = 3/group). Mean ± S.E.M. presented. *, significantly different from genotype-matched PBS/Air controls (*p* < 0.05). +, significantly different from prenatal treatment/neonate exposure-matched *Nrf2*^+/+^ mice (*p* < 0.05). #, significantly different from genotype-matched PBS/hyperoxia (O_2_) mice (*p* < 0.05).

HPLC-mass spectrometry *Nrf2*^+/+^ neonates had lower magnitude of neonatal hyperoxia-increased protein and lipid oxidation than *Nrf2*^−/−^ neonates, and prenatal SFN treatment significantly suppressed the enhanced protein oxidation (Figure 7A). Gestational SFN significantly suppressed both lung protein and lipid oxidation in hyperoxia-exposed *Nrf2*^−/−^ neonates (Figure 7A). However, the SFN-reduced protein and lipid oxidation levels in hyperoxia-exposed *Nrf2*^−/−^ neonates were significantly higher than those in similarly exposed *Nrf2*^+/+^ neonates (Figure 7A). Lung *Nrf2* mRNA expression was decreased in SFN/O_2_-*Nrf2*^+/+^ neonates than in PBS/O_2_-*Nrf2*^+/+^ neonates (Figure 7B). As we reported previously [19], ARE-bearing antioxidants, *Gpx2* and *Nqo1*, were transcriptionally induced by neonatal hyperoxia only in *Nrf2*^+/+^ neonates (Figure 7C). Prenatal SFN treatment lowered the hyperoxia-induced antioxidant expressions in *Nrf2*^+/+^ neonates (Figure 7C), indicating lowered oxidative stress by SFN. This corresponded to the lowered *Nrf2* mRNA expression in SFN/O_2_-*Nrf2*^+/+^ pups (Figure 7B) and was consistent with the results of SFN pretreatment in adult *Nrf2*^+/+^ mice [23,33]. Message levels of *Sod2*, a NRF2-independent antioxidant enzyme, increased by hyperoxia in both genotypes were also decreased by maternal SFN (Figure 7C), indicating the role of reactive oxygen species-quenching direct antioxidants including SOD2 in reduced oxidation and inflammation of SFN/O_2_-*Nrf2*^−/−^ lungs.

#### 3.2.4. SFN Effects on Lung Transcriptomics

**Figure 8:**
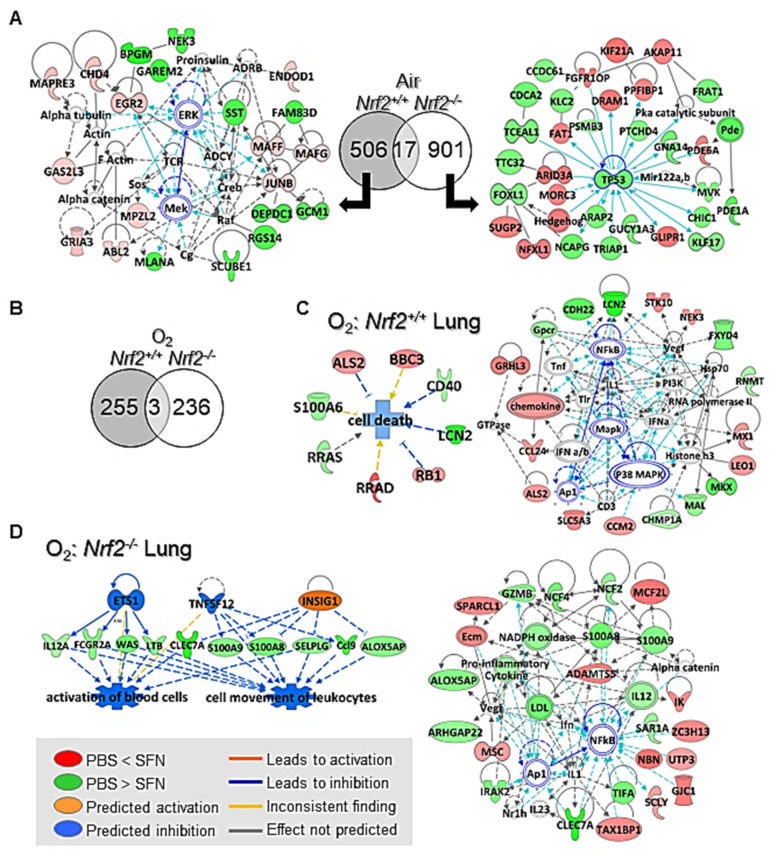
Lung transcriptome changes by prenatal sulforaphane (SFN) and affected biological functions and molecular networks predicted by pathway analyses in wild-type (*Nrf2*^+/+^) and *Nrf2*-deficient (*Nrf2*^−/−^) neonates. (**A**) Prenatal SFN changed lung transcriptome differentially in air-exposed *Nrf2*^+/+^ (*n* = 523) and in *Nrf2*^−/−^ (*n* = 918) neonates with 17 genes in common at postnatal day 4 (PND4). Pathway analysis demonstrated that mitogen-activated protein kinase (MAPK) cascade including extracellular-signal-regulated kinase (ERK) may play central roles in SFN-altered cell survival and organ growth and development transcriptome in *Nrf2*^+/+^ lungs. In *Nrf2*^−/−^ lungs, SFN mainly modulated genes facilitating cell morphology and cellular maintenance. (**B**) In neonate lungs exposed to hyperoxia (O_2_), transcriptome changes by maternal SFN in *Nrf2*^+/+^ (*n* = 258) and *Nrf2*^−/−^ (*n* = 239) were different. Pathway analysis indicated that prenatal SFN effect in O_2_-exposed *Nrf2*^+/+^ neonate lung favors to inhibit cell death and potentiate a network of tissue development (**C**), while SFN in *Nrf2^−/−^* neonate lungs may inhibit upstream regulators such as tumor necrosis factor ligand (e.g., TNF12) to suppress inflammatory response genes and activate cell-to-cell signaling and cellular maintenance network genes (**D**). In both strains of neonates exposed hyperoxia, nuclear factor kappa-light-chain-enhancer of activated B cells (NF-κB) and/or MAPK/AP-1 were predicted to play central roles in crosstalk of SFN-affected lung genes and their influence on cell/tissue functions. Molecular color and its intensity indicate up (red) or down (green) regulation degree of the genes by prenatal SFN compared to prenatal PBS after air or O_2_ exposure in each genotype. Ingenuity Pathway Analysis and GeneSpring software were used for data analyses.

*Air-exposure.* In air-exposed *Nrf2*^+/+^ neonates (PND4), SFN treatment significantly increased (*n* = 283) or decreased (*n* = 240) lung gene expressions relative to prenatal PBS (moderated *t*-test, *p* < 0.01; Table 1 and Table S1). Prenatal SFN-altered *Nrf2*^+/+^ neonates genes included integrin binding sialoprotein (*Ibsp*), cathepsin K (*Ctsk*), ARE-responsive antioxidants (e.g., *Aldh3a1*), Nrf2 heterodimerizing small Maf transcription factors (*Maff*, *Mafg*), and insulin-like growth factor binding protein 5 (*Igfbp5*) and they are proposed to be important in organ/cell development and growth (Figure 8A). In air-exposed *Nrf2*^−/−^ neonates, prenatal SFN altered the expression of genes (moderated *t*-test, *p* < 0.01; Table 1 and Table S2) including nuclear paraspeckle assembly transcript 1 (*Neat1*), olfactomedin 4 (*Olfm4*), NADPH oxidase 4 (*Nox4*), vascular endothelial growth factor A (*Vegfa*), *Igfbp2*, transformation related protein 53 (*Trp53*), and pathway analysis predicted their roles to favor cell morphogenesis and inhibit cell death and organismal injury/abnormality (Figure 8A). Venn diagram analysis indicated a significant dissociation of SFN-altered genes between air-exposed *Nrf2*^+/+^ and *Nrf2*^−/−^ neonates (Figure 8A) except 17 common genes such as transcription factors and developmental genes (e.g., *Crygf*, *Zfp551*, See Table 1). The baseline lung transcriptome changes by SFN were mostly less than twofold in both genotypes of mice (Table 1, Tables S1 and S2).

*Hyperoxia-exposure.* Similar to air controls, prenatal SFN-modulated lung genes in hyperoxia exposed neonates were also segregated between *Nrf2*^+/+^ (2-Way ANOVA, *p* < 0.01) and *Nrf2*^−/−^ (2-Way ANOVA, *p* < 0.01) and only solute carrier family 25 (mitochondrial carrier, dicarboxylate transporter), member 10 (*Slc25a10*), MIF4G domain containing (*Mif4gd*,), and centrosomal protein 128 (*Cep128*) were commonly changed by SFN in both strains (Figure 8B, Tables S3 and S4). In *Nrf2*^+/+^ neonates exposed to hyperoxia, transcriptome changes by prenatal SFN were favored to inhibit cell death and inflammation and activate cellular growth and development (Figure 8C). Increased organogenesis/development genes included protease, serine 35 (*Prss35*), *Cep128* and decreased inflammatory genes included histocompatibility 2, D region locus 1 (*H2-D1*), CD40 antigen (*Cd40*), lipocalin 2 (*Lcn2*), and cadherin 22 (*Cdh22*) were evident in these mice treated with prenatal SFN (Table 2 and Table S3). In *Nrf2*^−/−^ neonates exposed to hyperoxia, prenatal SFN decreased many hyperoxia-upregulated immune and inflammatory response genes as well as carcinogenesis-related genes including chemokine (C-C motif) ligand (*Ccl9*), B and T lymphocyte associated (*Btla*), and neutrophil cytosolic factors (e.g., *Ncf4*), lymphotoxin B (*Ltb*), selectin, platelet ligand (*Selplg*), C-type lectin domain family 7, member a (*Clec7a*), colony stimulating factor 2 receptor, beta, low-affinity (*Csf2rb*) (Figure 8D, Table 2 and Table S4). In addition, upregulation of many DNA repair/damage checkpoint genes such as tin interaction motif containing 1 (*Uimc1*), nei like 3 (*Neil3*), niblin (*Nbn*), and structural maintenance of chromosomes (*Smc4, Smc6*) compared to prenatal PBS treatment (Table 2 and Table S4). Insulin-induced gene 1 (INSIG1) and tumor necrosis factor ligand superfamily member 12 (TNFSF12) were among the potential upstream molecules affected by SFN in *Nrf2*^−/−^ transcriptome changes (Figure 8D). Although gestational SFN supplementation affected lung transcriptomics differentially in *Nrf2*^+/+^ and *Nrf2*^−/−^ pups, pathway analysis indicated that NF-κB and MAPK/activator protein 1 (AP-1) may play key roles in the downstream molecular networks of lung genes changed by SFN in both strains (Figure 8C,D). Importantly, hyperoxia effects on up or down regulation pattern of gene expression in prenatal PBS-treated neonates were reversed by prenatal SFN (Table 2, Tables S3 and S4). That is, hyperoxia-suppressed genes (i.e., decreased in PBS/O_2_ vs. PBS/Air) were mostly increased by prenatal SFN (i.e., higher in SFN/O_2_ vs. PBS/O_2_) and vice versa in both strains, indicating lowered hyperoxia-induced oxidative stress and inflammation in neonates given maternal SFN.

#### 3.2.5. SFN Effects on Placenta Transcriptomics

**Figure 9:**
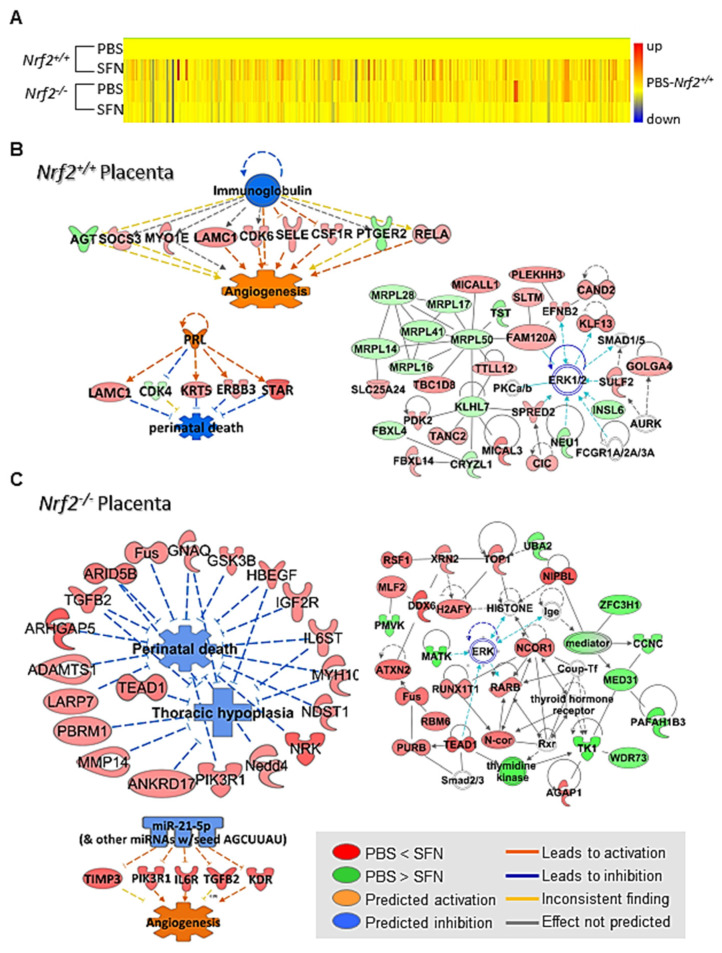
Placenta transcriptome changes by prenatal sulforaphane (SFN) and affected biological functions and molecular networks predicted by pathway analyses in wild-type (*Nrf2*^+/+^) and *Nrf2*-deficient (*Nrf2*^−/−^) mice at embryonic day 18.5. (**A**) Hierarchical clustering analysis generated a heap map depicting expression profiles of placenta genes altered by maternal SFN in *Nrf2*^+/+^ mice and *Nrf2*^−/−^ mice. Color bar indicates average expression intensity (*n* = 3/group) normalized to PBS-*Nrf2*^+/+^ group. (**B**) Prenatal SFN modulated 814 placenta genes in *Nrf2*^+/+^ mice (1.5-fold changed 708 genes). Pathway analysis of SFN-altered transcriptome predicted inhibition of perinatal death and activation of vasculature development by upstream modulators prolactin (PRL) and immunoglobulin in *Nrf2*^+/+^ placenta. A key network of SFN-influenced *Nrf2*^+/+^ placenta genes was connected with p42/p44 mitogen-activated protein kinase/extracellular-signal-regulated kinase (MAPK/ERK) and predicted to play roles in protein synthesis and cellular assembly and organization. (**C**) Prenatal SFN modulated 634 placenta genes in *Nrf2*^−/−^ mice (1.5-fold changed 367 genes). Pathway analysis of SFN-altered *Nrf2*^−/−^ transcriptome also suggested potential inhibition of perinatal death and activation of vasculature development. MicroRNA miR-21-5p was suggested as one of the upstream modulators of *Nrf2*^−/−^ placenta transcriptome changes by maternal SFN. ERK was also predicted to play central roles in crosstalk of SFN-modulated *Nrf2*^−/−^ placenta genes involved in cellular assembly and organization and organ development. Molecular color and its intensity indicate increased (red) or decreased (green) regulation degree of the genes by maternal SFN compared to maternal PBS in each genotype. Ingenuity Pathway Analysis and GeneSpring software were used for data analyses.

At E18.5, SFN treatment significantly altered placenta transcriptomics compared to PBS treatment in both genotypes of mice (*t*-test unpaired, *p* < 0.05). Transcriptome changes in *Nrf2*^+/+^ placenta varied from those in *Nrf2*^−/−^ placenta as illustrated in the heat map of hierarchical clustering analysis (Figure 9A). In *Nrf2*^+/+^ placenta, genes involved in estrogen receptor signaling and reproductive system development such as *Med* subfamily, *Igf1r*, and nuclear receptor co-repressor 2 (*Ncor2*), vasculature and embryonic development including ADAM-like, decysin 1 (*Adamdec1*), platelet derived growth factor receptor, beta polypeptide (*Pdgfrb*), collagens including *Col8a2*, *Sema3g*, keratin 5 (*Krt5*), and late cornified envelope 1 subfamilies (e.g., *Lce1a1*), and prenatal death and cell morbidity-related genes (e.g., DNA methyltransferase 3A (*Dnmt3a*), *Col4a2*) were predicted to be inhibited by SFN (Figure 9B, Table 3 and Table S5). Multiple granzyme isozymes (*Gzmd*, *Gzmg*, *Gzmf*, *Gzmc*) and oxidoreduction genes including GST isozymes (e.g., *Gstt1*, *Gstt2*), peroxiredoxin 5 (*Prdx5*), hydroxyacid oxidase (glycolate oxidase) 3 (*Hao3*), hydroxysteroid (17-beta) dehydrogenase 10 (*Hsd17b10*), and peroxisomal trans-2-enoyl-CoA reductase (*Pecr*) were suppressed in *Nrf2*^+/+^ placenta treated with SFN (Table 3 and Table S5). Overall transcriptome changes indicated reduced cytotoxicity and activated feto-placenta barrier functions. Potential upstream molecules including prolactin (PRL, activated), immunoglobulins (inhibited), or various microRNAs such as miR-29b-3p (inhibited) were proposed to be affected by SFN in *Nrf2*^+/+^ placenta transcriptome changes (Figure 9B). As seen in the postnatal lung transcriptomics, gestational SFN altered different placenta genes with similar cellular functions beneficial to host defense in *Nrf2*^+/+^ and *Nrf2*^−/−^ mice (Figure 9B,C). That is, SFN-altered transcriptome changes in *Nrf2*^−/−^ placenta were predicted to inhibit prenatal death via regulation of genes including *Igf2r*, transforming growth factor, beta 2 (*Tgfb2*), AT rich interactive domain 5B (*Arid5b*), and IL-6 signal transducer (*Il6st*) and to activate angiogenesis and cell growth by alteration of genes such as tissue inhibitor of metalloproteinase 3 (*Timp3*), kinase insert domain protein receptor (*Kdr*), and *Il6ra* (Figure 9C, Table 3 and Table S6). miR-21-5p was proposed to be one of the upstream regulators in SFN-altered *Nrf2*^−/−^ transcriptome changes for vascular development and prevention of cell death (Figure 9C). Pathway analysis revealed that MAPKs including ERK may be one of the key central players for crosstalk of SFN-altered genes in cellular organization and organ development processes in both genotypes (Figure 9B,C).

#### 3.2.6. Validation of Microarray and Pathway Analyses Results

**Figure 10:**
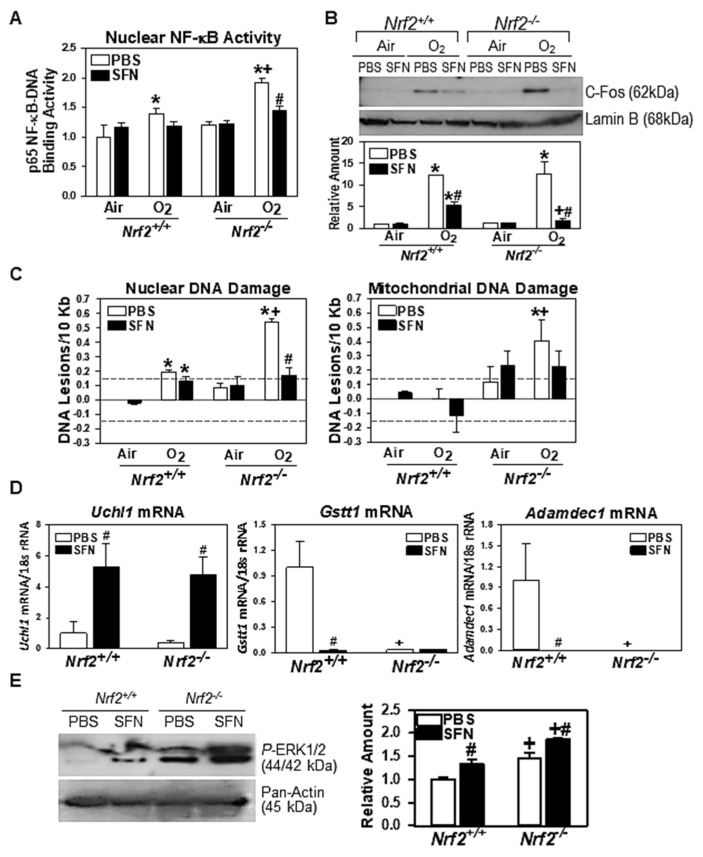
Validation of prenatal sulforaphane (SFN) effects on neonate lung and placenta transcriptome and signaling pathways. (**A**) Aliquots of nuclear proteins (7 μg) were subjected to colorimetric DNA-binding activity assay for nuclear factor kappa-light-chain-enhancer of activated B cells (NF-κB) p65 which was predicted to be involved in *Nrf2*^+/+^ and *Nrf2*^−/−^ neonate lung transcriptomics altered by maternal SFN after hyperoxia (O_2_) exposure. Group mean ± S.E.M presented (*n* = 4/group). (**B**) Western blotting determined nuclear protein level of c-Fos transcription factor which was proposed to play roles in SFN-altered lung transcriptomics in *Nrf2*^+/+^ and *Nrf2*^−/−^ neonates exposed to O_2_. Lamin B level was detected as a housekeeping control for nuclear proteins. Representative images from duplicate analyses presented. Scanned band images were quantitated by densitometry and relative protein band intensities normalize to PBS/air-*Nrf2*^+/+^ were depicted (mean ± S.E.M.). kDa = kilodalton. (**C**) qPCR was performed to determine SFN-mediated changes in DNA base lesions caused by O_2_ in nuclear and mitochondrial genomes. Lesion frequencies in genomic (DNA polymerase β gene) and mitochondrial DNA were compared in *Nrf2*^+/+^ and *Nrf2*^−/−^ neonate lungs after air or O_2_. All data were normalized to air-exposed *Nrf2*^+/+^ and group mean ± S.E.M. presented (*n* = 4/group). Background noise level (dashed lines) is set at ± 0.15. *, significantly different from genotype-matched PBS/Air controls (*p* < 0.05). +, significantly different from prenatal treatment/neonatal exposure matched *Nrf2*^+/+^ mice (*p* < 0.05). #, significantly different from genotype-matched PBS/hyperoxia (O_2_) mice (*p* < 0.05). (**D**) Placenta expressions of SFN-altered genes, ubiquitin carboxy-terminal hydrolase L1 (*Uchl1*), glutathione-s-transferase, theta 1 (*Gstt1*) and ADAM-like, decysin 1 (*Adamdec1*) were determined by qRT-PCR. Group mean ± S.E.M. presented (*n* = 3/group). (**E**) Western blotting determined placenta protein level of activated (phosphor) p42/p44 mitogen-activated protein kinase/extracellular signal-regulated kinase (*P*-ERK) which was proposed to play roles in SFN-altered placenta transcriptomics of *Nrf2*^+/+^ and *Nrf2*^−/−^ mice. Pan-actin level was determined as a housekeeping protein control for total proteins. Representative images from duplicate analyses presented. Scanned *P*-ERK band images were quantitated by densitometry and relative protein band intensities normalize to PBS-*Nrf2*^+/+^ were depicted (mean ± S.E.M.). kDa = kilodalton. #, significantly different from genotype-matched PBS controls (*p* < 0.05). +, significantly different from prenatal treatment matched *Nrf2*^+/+^ mice (*p* < 0.05).

*Neonate lung.* NF-κB and MAPK/AP-1 have been determined to be potential central molecules in the networks of prenatal SFN-modulated genes in *Nrf2*^+/+^ and *Nrf2*^−/−^ lungs exposed to hyperoxia (see Figure 8C,D). Hyperoxia-increased lung nuclear NF-κB (p65)-DNA binding activity was significantly greater in PBS/O_2_-*Nrf2*^−/−^ neonates than in PBS/O_2_-*Nrf2*^+/+^ neonates (Figure 10A). Activity was decreased in SFN-treated lungs of both strains but significant suppression of NF-κB activity was more evident in SFN/O_2_-*Nrf2*^−/−^ neonates (Figure 10A). The magnitude of NF-κB activity in all experimental groups paralleled expression patterns of many immune and inflammatory response genes (see Table 2, Tables S3 and S4). Redox responsive c-Fos, an AP-1 transcription factor, was increased markedly after neonatal hyperoxia exposure in both strains (Figure 10B). Concurrent with the lung levels of SFN-reduced oxidative stress and direct antioxidant (*Sod2*) expression in hyperoxia-exposed *Nrf2*^+/+^ and *Nrf2*^−/−^ lungs (see Figure 7A,C), the amount of c-Fos in both strains exposed to hyperoxia was lower in SFN-treated groups than in PBS-treated groups (Figure 10B). In addition, consistent with upregulation of multiple genes encoding DNA damage repair proteins in SFN/O_2_-*Nrf2*^−/−^ mice than in PBS/O_2_-*Nrf2*^−/−^ mice, hyperoxia-enhanced nuclear DNA damage was significantly reduced in SFN/O_2_-*Nrf2*^−/−^ lungs than in PBS/O_2_-*Nrf2*^−/−^ lungs. Significantly increased mitochondrial DNA damage was found only in PBS/O_2_-*Nrf2*^−/−^ lungs, and prenatal SFN decreased mitochondrial DNA damage in *Nrf2*^−/−^ lungs (Figure 10C). Genomic and mitochondrial DNA damages were significantly less frequent in *Nrf2*^+/+^ mice compared to *Nrf2*^−/−^ mice, and they were not significantly altered by gestational SFN (Figure 10C).

*Placenta.* qRT-PCR confirmed significant induction of ubiquitin carboxy-terminal hydrolase L1 (*Uchl1*), one of the commonly altered placenta genes by SFN in both mice (Figure 10D). Prenatal SFN markedly reduced expression of *Gstt1* and *Adamdec1* expression in *Nrf2*^+/+^ placenta while they were barely expressed in PBS-treated *Nrf2*^−/−^ placenta (Figure 10D). Activated (phosphorylated) protein level of placenta *P*-ERK1/2, a molecule proposed to be important in the networks of SFN-altered placenta transcriptomics in both genotypes (see Figure 9B,C) was heightened by SFN in both *Nrf2*^+/+^ and *Nrf2*^−/−^ placenta (Figure 10E).

## 4. Discussion

The current study demonstrated that oxidative lung injury caused by hyperoxia during postnatal lung development increased pulmonary susceptibility to RSV-induced injury and inflammation in young adulthood, indicating long term respiratory consequences of BPD-like phenotypes. In addition, genetic deletion of murine *Nrf2* further exacerbated RSV disease in later life. We also demonstrated that prenatal maternal administration of the phytochemical antioxidant SFN significantly reduced neonatal oxidative lung injury similar to BPD phenotypes in both *Nrf2*^+/+^ and *Nrf2*^−/−^ mice. The protective effects of gestational SFN were greater in hyperoxia-susceptible *Nrf2*^−/−^ pups than in hyperoxia-resistant *Nrf2*^+/+^ pups, indicating NRF2-dependent and -independent mechanisms of SFN actions. Supporting this outcome, comparative transcriptome analysis in *Nrf2*^+/+^ and *Nrf2*^−/−^ lungs determined that maternal SFN modulated different sets of genes involved in similar host defense pathways to facilitate lung cell survival and organ development and to suppress oxidative inflammation and DNA damage responses. Similarly, differential placenta transcriptome changes in *Nrf2*^+/+^ and *Nrf2*^−/−^ mice suggested a common beneficial role for prenatal SFN in utero signaling associated with inhibition of perinatal death and activation of angiogenesis.

Increasing evidence indicates that, compared to full-term infants, babies born prematurely are at significantly greater risk of adverse respiratory outcomes including BPD, respiratory failure, and infectious diseases [42,43,44]. Infants with BPD had increased risk of severe RSV disease relative to those without BPD [28,29]. Furthermore, low gestational age (<32 wk) and low birth weight (<1500 g) are critical risk factors for infant RSV hospitalization [30,43,44,45]. Consistent with these clinical observations, we found that young adult mice that were exposed to hyperoxia at the saccular stage of neonatal lung development carried on persistent lung injury including impaired alveoli and focal fibrotic changes and predisposed to more severe RSV disease. In addition, these long-term outcomes and RSV susceptibility were NRF2-dependent as were the hyperoxia-caused BPD-like lung injury in neonate mice and RSV disease severity in adult mice [19,33]. McGrath-Morrow et al. [18] also demonstrated NRF2-dependent persistent BPD-like phenotypes, and neonatally hyperoxia-exposed juvenile *Nrf2*^−/−^ mice had greater delay in lung damage repair relative to similarly-exposed juvenile *Nrf2*^+/+^ mice. Overall, our results indicate an essential role for NRF2-ARE-responsive defense and immune system in protection of prematurity with BPD against later life airway viral infection. Orally administered butylated hydroxyanisole, a NRF2 agonist, protected against RSV-induced lung oxidation, inflammation, viral replication, and airway hyperreactivity [34]. We also reported that RSV replication and lung neutrophilia was significantly decreased by SFN only in *Nrf2*^+/+^ mice [33]. Moreover, SFN pretreatment significantly induced transcriptome of mitochondrial oxidative phosphorylation and energy metabolism and ameliorated hyperoxia-induced acute lung injury in adult mice [23]. The collective observations strongly suggest that therapeutic intervention of NRF2-ARE modulation may significantly impact RSV disease severity, post-BPD phenotypes, and perhaps other oxidative airway disorders in adulthood.

Recent research has highlighted the prenatal effects of functional foods or stimuli on postnatal offspring health to investigate the fetal determinants on human disorders. As we reported in the current study, beneficial effects of prenatal maternal SFN on fetal programming or postnatal health has been reported in model disorders. In spontaneously hypertensive stroke-prone rats, dietary supplementation of SFN precursor during pregnancy reduced oxidative stress and hypertension and offspring also had lower blood pressure and tissue inflammation tissue inflammation [46]. Prenatal SFN precursor diet also markedly decreased breast cancer growth in mice and the anti-carcinogenesis efficacy of prenatal diet regimen was greater than that of early postnatal or adulthood supplementation [47]. In mouse embryos, intraperitoneally given SFN significantly reduced maternal ethanol exposure-induced apoptosis [48]. In newborn mouse skin, maternal intraperitoneal SFN treatment prevented *Krt14* mutation-related epidermis loses, and SFN was suggested to reprogram offspring keratin biosynthesis for skin integrity [35]. SFN nanoparticles in early chick culture alleviated heterocyclic aromatic amine compound-induced defects in central and peripheral nervous system and increased the embryo survival rate [49].

Unexpectedly, our results indicated that prenatal SFN-mediated postnatal protection against BPD-like phenotypes are not NRF2-dependent. Prenatal SFN markedly improved hyperoxia-caused severe BPD-like lung injury parameters in *Nrf2*^−/−^ pups while we observed relatively marginal protection by in utero SFN in hyperoxia-resistant *Nrf2*^+/+^ pups. SFN is a strong NRF2 and ARE gene inducer for cytoprotection by NRF2 stabilization through inhibition of a cytoplasmic NRF2 inhibitor Kelch-like ECH-associated protein 1 (KEAP1) via its covalent binding to KEAP1 thiol groups [20,21]. However, SFN also acts through other mechanisms including NF-κB inhibition [50,51], MAPK activation [52,53], and histone deacetylase inhibition [54] for anti-inflammation, chemoprevention, apoptosis, and autophagy. From our microarray analyses, we found that gestational SFN supplementation affected different transcriptomes of postnatal lungs in *Nrf2*-sufficient and -deficient mice. However, pathway analyses indicated that similar biological functions were activated or inhibited by the SFN-altered genes in the two strains to prevent perinatal death and activate organogenesis. In addition, NF-κB, MAPK, and AP-1 were predicted to be common central components of crosstalk among the SFN-altered genes in both strains. These analyses indicated the role for these proteins in NRF2-independent protection by SFN. In support of these findings, we reported inhibition of hyperoxia-increased nuclear NF-κB activity and c-Fos level by SFN in postnatal lungs of two strains. SFN-induced decreased expressions of many NF-κB target inflammatory genes, particularly in *Nrf2*^−/−^ lungs (e.g., *Ltb*, *S100a8*, *Il12a*, *Igll1*, see Table 2 and Table S4), further supported SFN-mediated inhibition of NF-κB pathway in oxidative inflammation for neonate lung protection against hyperoxia. In addition to SFN, known NRF2 agonists include *N*-acetyl -L-cysteine, synthetic triterpenoids (e.g., CDDO-Im (1[2-Cyano-3,12-dioxooleana-1,9(11)-dien-28-oyl]imidazole), and resveratrol, curcumin, and oltipraz [22,55]. On the other hand, there are pipeline NRF2 antagonists (pharmacological inhibitors) as anti-cancer therapies because NRF2 provides growth advantage of tumor cells [56].

Studies have demonstrated that hyperoxia causes formation of DNA adducts such as 7,8-dihydro-8-oxo-guanine and DNA strand breakage in mouse lungs [57]. Relative to DNA adduct formation common in most lung cell types, phosphodiester backbone breakage is the most severe level of damage and was unique in type 2 cells, and it led to the cell death and lung injury [57]. Genetic loss of *Nrf2* dysregulated redox homeostasis which promoted DNA lesions in neonatal lungs exposed to hyperoxia as seen previously [19]. Importantly, we determined in the present study that maternal SFN suppressed hyperoxia-induced nuclear and mitochondrial DNA damage in *Nrf2*^−/−^ neonate lungs, and it is postulated to be one of the NRF2-independent protective mechanisms of SFN. This was consistent with upregulation of multiple DNA repair/damage checkpoint genes (e.g., *Uimc1*, *Nbn*, *Neil3*, *Smc4, Smc6, Tonsl, Wdr48*, see Table S4) by SFN in hyperoxia-exposed *Nrf2*^−/−^ lungs. Genes involved in microtubule assembly, chromatin modification, histone acetylation, and genetic imprinting in *Nrf2*^−/−^ lungs (e.g., *Cep128*, *Clip1*, *Dek*, *Arid4b*, *Tdrd3*, *Ehmt1*) as well as in *Nrf2*^+/+^ lungs (e.g., *Stk10*, *Cep128*, *Drosha*, *Phf20*) were also increased by maternal SFN, indicating association of DNA methylation events (see Tables S3 and S4). Evidence indicated that hyperoxia causes increased DNA methylation (hypermethylation) in rat lung genes [58]. Lung epithelial cells also had hyperoxia-induced DNA damage which affected global DNA methylation status [59]. Studies with maternal SFN supplementation also suggested epigenetic alteration which was thought to underlie the positive effects of maternal SFN. In neural crest cells of mouse embryos, ethanol-induced reduction in histone acetylation at the *Bcl2* promoter was reversed by SFN, and it may be involved in anti-apoptosis function of SFN [48]. In utero SFN also downregulated histone deacetylase 1 and increased histone acetylation in the mouse mammary tumors for reactivation of tumor suppressor genes (e.g., *p16* and *p53*), and it may have governed the transcriptome changes affecting BRCA1 signaling pathway for anti-carcinogenesis [47]. Investigation of SFN-reprogramed epigenomes in embryonic and extra-embryonic tissues as well as postnatal lungs are further warranted.

A recent study found that NRF2 is critical in placenta integrity for fetal growth and reduced total and labyrinth volume of placenta in *Nrf2*^−/−^ mice at E18.5 was predicted to compromise and abolish the adaptational increase of nutrient transfer capacity to meet fetal growth demands [60]. Investigation of the NRF2 pathway detected significant compositional changes of amniotic fluids during late gestation and upregulated ARE-bearing epidermal differentiation complex genes, small proline-rich protein 2 (*Sprr2d*, *Sprr2h*), which reverted the defected epidermal barrier formation in loricrin (*Lor*)-deficient mouse embryos [61]. In the current study, we compared maternal SFN-induced placenta transcriptome changes between *Nrf2*^+/+^ and *Nrf2*^−/−^ mice at E18.5. SFN significantly induced multiple epidermal differentiation complex genes *(Lor* and Lce1 subfamily genes) in *Nrf2*^+/+^ placenta, but not in *Nrf2*^−/−^ placenta. However, the *Nrf2*^−/−^ placenta differentially responded to SFN and modulated compensatory epidermal/mesenchymal development (e.g., *Lect1*, *Irs2*, *Arhgap5*, *Thoc2*, *Tead1*) and angiogenesis (e.g., *Hbegf*, *Kdr*, *Apold1*) genes to reprogram the feto–placenta barrier function. Interestingly, we found that SFN-altered placenta genes such as *Adamdec1*, *H2-D1*, *Gstt1*, and *Igfbp1* in *Nrf2*^+/+^ mice were also changed by maternal SFN in fetal lungs of the same gestation age. Pathway analyses also indicated similar functional networks (e.g., organ and system development, lipid metabolism, cell death and survival) affected by maternal SFN in placenta and fetal lungs.

In conclusion, we demonstrated that early-life oxidant-induced acute lung injury had significant consequences later in life on NRF2-dependent RSV susceptibility in mice. We also determined that increased antioxidant conditions in utero potentially contribute to a decreased risk of postnatal airway disease as we found that prenatal antioxidant SFN protected developing lungs from BPD-like oxidative pathogenesis in mice. Among the NRF2-independent protective mechanisms of SFN in *Nrf2*^−/−^ lungs were upregulation of DNA damage repair molecules and NF-κB inhibition. Our study provided new insights into the infant oxidant lung injury severity influence on persistence of pulmonary morbidity and the therapeutic intervention for NRF2 agonists. Our results also provided justification for further studies on feto–placental barrier crossing of SFN metabolites and SFN-triggered molecular and epigenetic aspects of maternal cues for barrier and fetal lung signaling.

## Figures and Tables

**Table 1:** Representative lung genes significantly changed by prenatal maternal sulforaphane (SFN) in air-exposed wild-type (*Nrf2*^+/+^) and *Nrf2*-deficient (*Nrf2*^−/−^) neonates at postnatal day (PND) 4.

Genotype	^†^ FC	Gene Symbol	Gene Title	Gene Ontology
*Nrf2+/+*	6.85	*Ibsp*	integrin binding sialoprotein	cell adhesion
	5.31	*Ctsk*	cathepsin K	proteolysis
	2.07	*Calcb*	calcitonin-related polypeptide, beta	vasodilation
	2.05	*Lilrb4*	leukocyte immunoglobulin-like receptor, subfamily B, member 4	immune system process
	2.01	*Dnm3os*	dynamin 3, opposite strand (*Mir214*)	skeletal system development
	1.69	*Egr2*	early growth response 2	transcription
	1.69	*Myof*	Myoferlin	vascular endothelial growth factor receptor signaling
	1.53	*Aldh3a1*	aldehyde dehydrogenase family 3, subfamily A1	response to hypoxia
	1.52	*Ereg*	Epiregulin	angiogenesis
	1.49	*Igfbp5*	insulin-like growth factor binding protein 5	cell growth, glucose metabolism
	1.42	*Maff*	v-maf musculoaponeurotic fibrosarcoma oncogene family, protein F	embryonic development, transcription regulation
	−1.64	*Gpr165*	G protein-coupled receptor 165	signal transduction
	−1.60	*Tspo2*	translocator protein 2	transport
	−1.56	*Bpgm*	2,3-bisphosphoglycerate mutase	glycolytic process
	−1.52	*Lce1i*	late cornified envelope 1I	epidermal development
	−1.48	*Il20*	interleukin 20	hemopoiesis
	−1.42	*Nkx2-9*	NK2 homeobox 9	respiratory tube development
	2.73	*Olfm4*	olfactomedin 4	cell adhesion
*Nrf2* ^−/−^	2.66	*Neat1*	nuclear paraspeckle assembly transcript 1	cellular component maintenance
	2.46	*Meg3*	maternally expressed 3	in utero embryonic development
	2.33	*Stfa1*	stefin A1	negative regulation of peptidase activity
	1.98	*Amy1*	amylase 1, salivary	carbohydrate metabolism
	1.65	*Ptprc*	protein tyrosine phosphatase, receptor type, C	MAPK activation
	1.55	*Cd14*	CD14 antigen	immune system process
	1.50	*Sema4a*	sema domain, immunoglobulin domain (Ig), transmembrane domain (TM) and short cytoplasmic domain, (semaphorin) 4A	angiogenesis
	1.30	*Vegfa*	vascular endothelial growth factor A	angiogenesis
	−2.48	*Cda*	cytidine deaminase	negative regulation of cell growth
	−2.44	*St6gal2*	beta galactoside alpha 2,6 sialyltransferase 2	carbohydrate metabolism
	−2.18	*Arsk*	arylsulfatase K	metabolic process
	−2.15	*Igfbp2*	insulin-like growth factor binding protein 2	cell growth, response to stress
	−1.67	*Cdc25c*	cell division cycle 25C	mitotic cell cycle
	−1.61	*Igf1*	insulin-like growth factor 1	cell activation, vessel remodeling
	−1.41	*Nox4*	NADPH oxidase 4	oxidation–reduction process pppprocessremodeling

SFN or PBS was orally administrated to foster dams on embryonic days 11.5, 13.5, 15.5, and 17.5. Microarray analysis was done with age PND4 tissues. ^†^ Fold change in SFN/air vs. PBS/air in each genotype. Full gene list in Supplementary Table S1 (prenatal SFN-altered 523 genes in *Nrf2*^+/+^, moderated *t*-test with *p* < 0.01) and Table S2 (prenatal SFN-altered 918 genes in *Nrf2*^−/−^, moderated *t*-test with *p* < 0.01). Genes commonly altered by SFN in both genotypes includes Alport syndrome, mental retardation, midface hypoplasia and elliptocytosis chromosomal region gene 1 (*Ammecr1*), bromodomain containing 2 (*Brd2*), crystallin, gamma F (*Crygf*), cysteine rich tail 1 (*Cysrt1*), Dmx-like 2 (*Dmxl2*), E74-like factor 5 (*Elf5*), F-box and WD-40 domain protein 22 (*Fbxw22*), growth arrest-specific 2 like 3 (*Gas2l3*), leucine rich repeat containing 36 (*Lrrc36*), lymphocyte antigen 75 (*Ly75*), and zinc finger protein 551 (*Zfp551*).

**Table 2:** Representative lung genes significantly changed by prenatal maternal sulforaphane (SFN) in hyperoxia-exposed wild-type (*Nrf2*^+/+^) and *Nrf2*-deficient (*Nrf2*^−/−^) neonates.

Genotype	* FC by O_2_	^†^ FD PBS:SFN	Gene Symbol	Gene Title	Gene Ontology
*Nrf2+/+*	−2.22	2.32	*Dkk2*	dickkopf homolog 2	transcriptional regulation
	−4.35	1.89	*Prss35*	protease, serine 35	proteolysis, metabolism
	−1.70	1.89	*Frem1*	Fras1 related extracellular matrix protein 1	cell communication
	−2.33	1.86	*Rrad*	Ras-related associated with diabetes	negative regulation of cell growth
	−1.04	1.78	*Klhdc7a*	kelch domain containing 7A (microRNA 2139)	protein binding
	−1.46	1.61	*Fam26e*	family with sequence similarity 26, member E	transport
	−1.55	1.52	*Cep128*	centrosomal protein 128	microtubule organization
	−1.29	1.28	*Ggt7*	gamma-glutamyltransferase 7	glutathione metabolism
	3.50	1.19	*Ephx1*	epoxide hydrolase 1, microsomal	response to toxicant
	4.39	−3.07	*Lcn2*	lipocalin 2	immune system process
	1.10	−2.33	*H2-D1*	histocompatibility 2, D region locus 1	T cell mediated cytotoxicity
	1.82	−2.29	*Cdh22*	cadherin 22	cell adhesion
	2.13	−2.28	*Mkx*	mohawk homeobox	negative regulation of transcription
	3.03	−2.19	*Msln*	mesothelin	cell adhesion
	1.88	−1.75	*Tgm1*	transglutaminase 1, K polypeptide	organ morphogenesis
	2.75	−1.31	*Liph*	lipase, member H	lipid metabolism
	1.98	−1.28	*Ctsh*	cathepsin H	T cell mediated cytotoxicity
*Nrf2* ^−/−^	−1.69	3.10	*Amy1*	amylase 1, salivary	carbohydrate metabolism
	−2.03	2.24	*Scara5*	scavenger receptor class A, member 5	transport
	−1.80	1.79	*Fap*	fibroblast activation protein	proteolysis
	−1.73	1.73	*Cep128*	centrosomal protein 128	microtubule organization
	−1.98	1.65	*Cc2d2a*	coiled-coil and C2 domain containing 2A	cilium assembly
	−1.71	1.64	*Uimc1*	ubiquitin interaction motif containing 1	DNA repair
	−1.37	1.57	*Cep83*	centrosomal protein 83	cilium assembly
	−1.37	1.56	*Nbn*	nibrin	DNA damage checkpoint
	−2.11	1.51	*Smc4*	structural maintenance of chromosomes 4	DNA repair
	4.51	−2.77	*Clec7a*	C-type lectin domain family 7, member a	pattern recognition receptor signaling
	1.89	−2.40	*Cd52*	CD52 antigen	immune response
	4.53	−2.26	*Ccl9*	chemokine (C-C motif) ligand 9	chemotaxis
	1.67	−1.67	*Ncf4*	neutrophil cytosolic factor 4	cell communication
	1.66	−1.67	*Tifa*	TRAF-interacting protein with forkhead-associated domain	NF-kappa B signaling
	1.74	−1.52	*Gzmb*	granzyme B	T cell mediated cytotoxicity
	2.11	−1.46	*Csf2rb*	colony stimulating factor 2 receptor, beta, low-affinity (granulocyte-macrophage)	cytokine signaling
	2.16	−1.46	*Cyp3a13*	cytochrome P450, family 3, subfamily a, polypeptide 13	oxidation–reduction process
	1.46	−1.45	*Ltb*	lymphotoxin B	immune response
	1.58	−1.40	*Selplg*	selectin, platelet (p-selectin) ligand	leukocyte adhesion

SFN or PBS was orally administration to foster dams on embryonic days 11.5, 13.5, 15.5, and 17.5 and newborn pups were exposed to air or hyperoxia (O_2_) for 3 days during postnatal days (PND) 1–4. Microarray was done for the lungs harvested at the end of exposure (PND4). * Fold change (FC) by hyperoxia (PBS/hyperoxia) vs. PBS/air in each genotype. ^†^ Fold difference (FD) in SFN/O_2_ vs. PBS/O_2_ of each genotype. Full gene list in Supplementary Table S3 (Prenatal SFN-altered genes in hyperoxia-exposed *Nrf2*^+/+^, 2-Way ANOVA with *p* < 0.01 *n* = 258) and Table S4 (Prenatal SFN altered genes in hyperoxia-exposed *Nrf2*^−/−^, 2-way ANOVA with *p* < 0.01, *n* = 239). Commonly altered genes in both genotypes are solute carrier family 25 (mitochondrial carrier, dicarboxylate transporter), member 10 (*Slc25a10*), MIF4G domain containing (*Mif4gd*), and centrosomal protein 128 (*Cep128*).

**Table 3:** Representative genes significantly changed by prenatal maternal sulforaphane (SFN) in wild-type (*Nrf2*^+/+^) and *Nrf2*-deficient (*Nrf2*^−/−^) placenta at embryonic day (E) 18.5.

Genotype	^†^ FC	Gene Symbol	Gene Title	Gene Ontology
*Nrf2+/+*	7.33	*Stfa3*	stefin A3	endopeptidase inhibitor
	4.33	*Tmem178*	transmembrane protein 178	structural molecule activity
	3.69	*Uchl1*	ubiquitin carboxy-terminal hydrolase L1	axonogenesis
	3.04	*Lce1d*	late cornified envelope 1D	epidermal development
	2.92	*Col8a2*	collagen, type VIII, alpha 2	morphogenesis
	2.88	*Slc6a2*	solute carrier family 6 (neurotransmitter transporter, noradrenalin), member 2	monoamine transport
	2.77	*Lor*	loricrin	keratinocyte differentiation
	2.57	*Pdgfrb*	platelet derived growth factor receptor, beta polypeptide	cell proliferation
	2.51	*Dnmt3a*	DNA methyltransferase 3A	DNA methylation
	2.19	*Sema3g*	sema domain, immunoglobulin domain (Ig), short basic domain, secreted, (semaphorin) 3G	organ development
	1.97	*Pik3r1*	phosphatidylinositol 3-kinase, regulatory subunit, polypeptide 1	insulin-like growth factor receptor signaling pathway
	1.92	*Med13l*	mediator complex subunit 13-like	transcription regulation
	1.55	*Aldh1l2*	aldehyde dehydrogenase 1 family, member L2	oxidation reduction
	−109.27	*Adamdec1*	ADAM-like, decysin 1	proteolysis
	−16.14	*H2-D1*	histocompatibility 2, D region locus 1	antigen processing and presentation
	−7.84	*Lect1*	leukocyte cell derived chemotaxin 1	organ development
	−5.37	*Hao3*	hydroxyacid oxidase (glycolate oxidase) 3	oxidation reduction
	−4.43	*Gstt1/t2*	glutathione S-transferase, theta 1/2	glutathione metabolism
	−4.11	*Gzmd*	granzyme D	cytolysis
	−3.86	*Igfbp1*	insulin-like growth factor binding protein 1	cell growth regulation
	−1.80	*Ccnc*	cyclin C	transcription regulation
	−1.78	*Ndufaf1*	NADH dehydrogenase (ubiquinone) 1 alpha subcomplex, assembly factor 1	ubiquinone) activity
	−1.69	*Irak1bp1*	Interleukin-1 receptor-associated kinase 1 binding protein 1	NF-kappa B cascade
	−1.68	*Prdx5*	peroxiredoxin 5	oxidation reduction
*Nrf2* ^−/−^	4.71	*Lect1*	leukocyte cell derived chemotaxin 1	organism development
	2.19	*Uchl1*	ubiquitin carboxy-terminal hydrolase L1	response to ischemia
	2.16	*Ppargc1b*	peroxisome proliferative activated receptor, gamma, coactivator 1 beta	transcription regulation
	1.95	*Arid5b*	AT rich interactive domain 5B	development
	1.74	*Hbegf*	heparin-binding EGF-like growth factor	angiogenesis
	1.74	*Timp3*	tissue inhibitor of metalloproteinase 3	neurotransmitter secretion
	1.72	*Kdr*	kinase insert domain protein receptor	angiogenesis
	1.70	*Figf*	c-fos induced growth factor	angiogenesis
	1.63	*Igf2r*	insulin-like growth factor 2 receptor	post-embryonic development
	−3.13	*Uty*	ubiquitously transcribed tetratricopeptide repeat gene, Y chromosome	in utero embryonic development
	−1.96	*Ccne2*	cyclin E2	mitotic cell cycle
	−1.73	*Tk1*	thymidine kinase 1	DNA replication
	−1.62	*Cox7a1*	cytochrome c oxidase subunit VIIa 1	oxidation–reduction process
	−1.59	*Cyp1a1*	cytochrome P450, family 1, subfamily a, polypeptide 1	response to hypoxia
	−1.59	*Mir17hg*	Mir17 host gene 1	in utero embryonic development
	−1.58	*Pafah1b3*	platelet-activating factor acetylhydrolase, isoform 1b, subunit 3	lipid metabolic process

SFN or PBS was orally administration to foster dams on E11.5, E13.5, E15.5, and E17.5 and placenta were collected at E18.5. **^†^** Fold change (FC) by SFN vs. PBS in each genotype. Full gene list in Supplementary Table S5 (Prenatal SFN altered placenta genes in *Nrf2*^+/+^, unpaired *t*-test with *p* < 0.05, 1.5-fold 708 genes out of 2427 genes) and Table S6 (Prenatal SFN altered placenta genes in *Nrf2*^−/−^, unpaired *t*-test with *p* < 0.05 *n* = 367 out of 634 genes). Commonly altered genes in both genotypes including ubiquitin carboxy-terminal hydrolase L1 (*Uchl1*), Kruppel-like factors (*Klf9*, *Klf7*), and leukocyte cell derived chemotaxin 1 (*Lect1*) are marked in Table S6.

## Data Availability

Microarray data are deposited in a public database repository (Gene Expression Omnibus accession numbers: GSE164699, GSE164700). Data is contained within the article or supplementary material.

## Supplementary Materials

The following are available online at https://www.mdpi.com/article/10.3390/antiox10121874/s1, Table S1: Prenatal sulforaphane-altered lung genes in postnatal air-exposed *Nrf2*^+/+^ mice (*n* = 523, moderated *t*-test, *p* ≤ 0.01), Table S2: Prenatal sulforaphane-altered 918 lung genes in postnatal air-exposed *Nrf2*^−/−^ mice (*n* = 918, moderated *t*-test, *p* ≤ 0.01), Table S3: Prenatal sulforaphane-neonatal hyperoxia interaction 258 genes in *Nrf2*^+/+^ mice (2-Way ANOVA, *p* ≤ 0.01), Table S4: Prenatal sulforaphane and neonatal hyperoxia interaction 239 genes in *Nrf^−/−^* mice (2-Way ANOVA, *p* ≤ 0.01), Table S5: Prenatal sulforaphane-modulated genes in *Nrf2*^+/+^ placenta (*t*-test unpaired, *p* ≤ 0.05), Table S6: Prenatal sulforaphane-modulated genes in *Nrf2*^−/−^ placenta (*t*-test unpaired, *p* ≤ 0.05).
