# Supplementary material for: Murine Neonatal Oxidant Lung Injury: NRF2-Dependent Predisposition to Adulthood Respiratory Viral Infection and Protection by Maternal Antioxidant
Source: Antioxidants (Basel). 2021 Nov 24;10(12):1874. doi: 10.3390/antiox10121874 (PMC8698620; doi:10.3390/antiox10121874)
Supplement: Supplementary file 1 [file antioxidants-10-01874-s001.zip › antioxidants-1453559-supplementary.pdf]

Table S1. Prenatal sulforaphane-altered lung genes in Nrf2$^{+/+}$ mice (n=523, moderated T-Test, P<=0.01)

RIKEN cDNA and predicted genes excluded. Fold Change (FC): Blue (-) - fold lower in SFN than PBS, Red - fold higher in SFN than PBS.

[ICR, Air, PBS]	[ICR, Air, SFN](	[ICR, O2, PBS](	[ICR, O2, SFN](	RefSeq Transcript ID	p	FC Air-PBS:Air-SFN	Gene Symbol	Gene Title	Gene Ontology
31.70388	211.04439	139.37938	102.874115	NM_008318///XM_C	0.003	6.85	***Ibsp***	integrin binding sialoprotein	0001503 // ossification // inferred from electronic annotation///0001649 // osteoblast differentiati
85.096954	466.24686	82.19377	71.791115	NM_007802///XM_C	0.007	5.31	***Ctsk***	cathepsin K	0001957 // intramembranous ossification // inferred from electronic annotation///0006508 // prot
55.982994	120.962814	65.48201	124.95033	NM_019496	0.004	2.18	***Ammecr1***	Alport syndrome, mental retardation, midface hypoplasia and elliptocytosis chromosomal region gene 1	0005515 // protein binding // inferred from electronic annotation
46.458015	95.48914	56.458	47.88668	NM_054084///XM_C	0.005	2.07	***Calcb***	calcitonin-related polypeptide, beta	0042311 // vasodilation // inferred from electronic annotation
140.88501	306.8137	464.6552	672.98926	NM_001291892///N	0.000	2.05	***Gp49a///Lilrb4***	glycoprotein 49 A///leukocyte immunoglobulin-like receptor, subfamily B, member 4	0002376 // immune system process // inferred from electronic annotation
75.313576	154.99268	101.2082	145.34927	NR_002870///NR_02	0.007	2.01	***Dnm3os///Mir214***	dynamin 3, opposite strand///microRNA 214	0001501 // skeletal system development // inferred from mutant phenotype///0003323 // type B p
53.794945	95.00381	121.75727	125.865616	NM_144783	0.001	1.79	***Wt1***	Wilms tumor 1 homolog	0000122 // negative regulation of transcription from RNA polymerase II promoter // not recorded/,
121.42094	227.32707	274.37772	248.9197	NM_001081287///N	0.008	1.77	***Mpp7***	membrane protein, palmitoylated 7 (MAGUK p55 subfamily member 7)	0031334 // positive regulation of protein complex assembly // not recorded///0070830 // tight junc
794.10046	1420.3386	1273.9543	1594.5337	NM_009778	0.002	1.74	***C3***	complement component 3	0001798 // positive regulation of type IIa hypersensitivity // inferred from mutant phenotype///000
60.04707	104.37409	127.874115	56.07898	NM_177595	0.002	1.74	***Mkx***	mohawk homeobox	0000122 // negative regulation of transcription from RNA polymerase II promoter // inferred from
109.92215	196.53908	159.87926	241.24054	NM_001081342///N	0.008	1.72	***Gpr133***	G protein-coupled receptor 133	0007166 // cell surface receptor signaling pathway // inferred from electronic annotation///00071
43.92867	72.22747	69.90488	143.80573	NM_010118///XM_C	0.000	1.69	***Egr2***	early growth response 2	0006351 // transcription, DNA-templated // inferred from electronic annotation///0006355 // regu
51.605183	85.341324	38.49855	69.14183	NM_001112798///N	0.004	1.69	***Slc8a1***	solute carrier family 8 (sodium/calcium exchanger), member 1	0001666 // response to hypoxia // not recorded///0001892 // embryonic placenta development //
70.13851	120.759285	70.42109	71.732956	NM_013549///NM_:	0.001	1.69	***Hist2h2aa1///Hist2h2aa2***	histone cluster 2, H2aa1///histone cluster 2, H2aa2	0006334 // nucleosome assembly // inferred from electronic annotation///0006334 // nucleosome
519.179	912.1046	993.2537	1059.4312	NM_001099634///N	0.001	1.69	***Myof***	myoferlin	0001778 // plasma membrane repair // inferred from mutant phenotype///0006906 // vesicle fusic
24.761656	38.52286	28.732605	40.1172	NM_001164493	0.001	1.67	***Klhl29***	kelch-like 29	0005515 // protein binding // inferred from electronic annotation
28.037014	43.627186	13.981073	24.802511	NM_001271019///N	0.002	1.66	***Siglec5***	sialic acid binding Ig-like lectin 5	0007155 // cell adhesion // inferred from electronic annotation
61.26334	101.78379	89.497955	107.2649	NM_133350///XM_C	0.001	1.66	***Mapre3***	microtubule-associated protein, RP/EB family, member 3	0007049 // cell cycle // inferred from electronic annotation///0007067 // mitotic nuclear division /,
17.816998	27.668245	19.271816	33.47256	NM_001081221///N	0.003	1.65	***Ercc6***	excision repair cross-complementing rodent repair deficiency, complementation group 6	0000303 // response to superoxide // inferred from mutant phenotype///0006200 // ATP catabolic
339.01733	580.95264	391.6923	666.7519	NM_027707///NM_:	0.004	1.64	***Nipbl***	Nipped-B homolog (Drosophila)	0000122 // negative regulation of transcription from RNA polymerase II promoter // not recorded/,
50.261986	79.81739	67.7412	78.21371	NM_001033820///XI	0.008	1.63	***Zfp551***	zinc fingr protein 551	0006355 // regulation of transcription, DNA-templated // inferred from electronic annotation
75.56271	124.41099	152.30849	150.44853	NM_007562	0.002	1.62	***Bnc1***	basonuclin 1	0006351 // transcription, DNA-templated // inferred from electronic annotation///0006355 // regu
20.405077	30.765308	14.209172	25.667747	NM_001164785///N	0.001	1.62	***Adamts20***	a disintegrin-like and metallopeptidase (reprolysin type) with thrombospondin type 1 motif, 20	0006508 // proteolysis // inferred from direct assay///0007229 // integrin-mediated signaling pathy
234.03123	397.14606	314.74744	394.0639	NM_030206	0.002	1.62	***Cygb***	cytoglobin	0006810 // transport // inferred from electronic annotation///0006979 // response to oxidative str
52.2494	81.80647	259.31113	247.28572	NM_009704	0.006	1.60	***Areg***	amphiregulin	0007173 // epidermal growth factor receptor signaling pathway // not recorded///0007186 // G-pr
125.02729	208.29207	171.68842	333.72653	NM_175406	0.003	1.59	***Atp6v0d2***	ATPase, H+ transporting, lysosomal V0 subunit D2	0006810 // transport // inferred from electronic annotation///0006811 // ion transport // inferred
1158.877	1873.5059	1495.2028	1955.8738	NM_001025387///N	0.002	1.59	***Brd2***	bromodomain containing 2	0006334 // nucleosome assembly // not recorded///0006351 // transcription, DNA-templated // in
107.42899	176.12843	235.57437	467.25763	NM_001281929///N	0.005	1.57	***Gria3***	glutamate receptor, ionotropic, AMPA3 (alpha 3)	0001919 // regulation of receptor recycling // not recorded///0006810 // transport // inferred fron
53.70051	83.161995	52.959114	67.9727	NM_001033371///N	0.008	1.57	***Lrrc36***	leucine rich repeat containing 36	
262.46674	431.30695	211.01021	359.76227	NM_001110843///N	0.005	1.56	***Cacna2d1***	calcium channel, voltage-dependent, alpha2/delta subunit 1	0006810 // transport // inferred from electronic annotation///0006811 // ion transport // inferred
35.89475	53.72552	60.431572	51.676067	NM_010408///XM_C	0.007	1.56	***Hcn1***	hyperpolarization-activated, cyclic nucleotide-gated K+ 1	0006810 // transport // inferred from electronic annotation///0006811 // ion transport // inferred
663.3817	1061.6375	529.50555	865.80786	NM_145979///XM_C	0.008	1.55	***Chd4***	chromodomain helicase DNA binding protein 4	0006200 // ATP catabolic process // inferred from electronic annotation///0006325 // chromatin oi
84.82471	132.87904	99.08483	89.56542	NM_198886///XM_C	0.009	1.53	***Zbtb12***	zinc finger and BTB domain containing 12	0003676 // nucleic acid binding // inferred from electronic annotation///0005515 // protein binding
35.902283	52.35833	35.146736	43.93301	NM_001112725///N	0.001	1.53	***Aldh3a1***	aldehyde dehydrogenase family 3, subfamily A1	0001666 // response to hypoxia // not recorded///0006081 // cellular aldehyde metabolic process ,
663.62335	1053.6553	966.693	1393.5911	NM_018826	0.004	1.53	***Irx5***	Iroquois related homeobox 5 (Drosophila)	0002027 // regulation of heart rate // inferred from mutant phenotype///0006351 // transcription,
21.249775	30.12115	66.71911	94.42819	NM_007950	0.004	1.52	***Ereg***	epiregulin	0001525 // angiogenesis // inferred from electronic annotation///0001550 // ovarian cumulus expa
31.079636	44.432465	28.631624	20.66898	NM_001290825///N	0.000	1.52	***Col17a1***	collagen, type XVII, alpha 1	0031581 // hemidesmosome assembly // inferred from sequence or structural similarity///0031581
51.396664	75.82202	73.33496	125.078835	NM_013825	0.003	1.51	***Ly75***	lymphocyte antigen 75	0006897 // endocytosis // inferred from electronic annotation
24.25811	33.944386	30.495024	36.866108	NM_027425///XM_C	0.004	1.51	***Rufy2***	RUN and FYVE domain-containing 2	0046872 // metal ion binding // inferred from electronic annotation
1153.3927	1767.9536	1137.417	1414.4901	NM_010518	0.004	1.49	***Igfbp5***	insulin-like growth factor binding protein 5	0001558 // regulation of cell growth // inferred from genetic interaction///0001649 // osteoblast d
290.9779	454.98215	355.3063	526.86945	NM_001033155///XI	0.003	1.49	***1110018F16Rik///Dnajb1***	RIKEN cDNA 1110018F16 gene///DnaJ (Hsp40) homolog, subfamily B, member 14	0016020 // membrane // inferred from electronic annotation///0016021 // integral component of
33.810482	47.470654	38.702503	32.906536	NM_029360	0.001	1.49	***Tm4sf5***	transmembrane 4 superfamily member 5	0016020 // membrane // inferred from electronic annotation///0016021 // integral component of
535.4431	832.6205	311.0486	563.9149	NM_172471///XM_C	0.009	1.49	***Itih5***	inter-alpha (globulin) inhibitor H5	0006614 // SRP-dependent cotranslational protein targeting to membrane // inferred from electror
122.8398	190.98245	185.37529	149.05247	NM_021719	0.005	1.48	***Cldn15***	claudin 15	0006810 // transport // inferred from electronic annotation///0006811 // ion transport // inferred
19.760483	27.25687	23.63514	21.806305	NR_040389	0.004	1.48	***Gm10785***	predicted gene 10785	
51.851913	74.192406	75.76936	96.692184	NM_027519///XM_C	0.010	1.47	***Medag***	mesenteric estrogen dependent adipogenesis	0045600 // positive regulation of fat cell differentiation // inferred from mutant phenotype
1821.1898	2690.7188	2351.985	2372.9458	NM_011896///XM_C	0.003	1.46	***Spry1***	sprouty homolog 1 (Drosophila)	0000132 // establishment of mitotic spindle orientation // inferred from genetic interaction///0001
99.25308	150.39975	162.50865	308.29987	NM_008331	0.003	1.46	***Ifit1***	interferon-induced protein with tetratricopeptide repeats 1	0002376 // immune system process // inferred from electronic annotation///0009615 // response t
93.340866	140.65144	101.4492	182.96014	NM_011854///XM_C	0.008	1.46	***Oasl2***	2'-5' oligoadenylate synthetase-like 2	0002376 // immune system process // inferred from electronic annotation///0006164 // purine nuc
108.7908	164.34299	176.71744	205.29285	NM_001164528///XI	0.004	1.45	***Ildr2***	immunoglobulin-like domain containing receptor 2	0009749 // response to glucose // inferred from mutant phenotype///0030073 // insulin secretion
115.71432	176.6755	192.81125	243.64786	NM_001081678///XI	0.010	1.45	***Zfp800***	zinc finger protein 800	0006351 // transcription, DNA-templated // inferred from electronic annotation///0006355 // regu
73.61993	106.82851	97.119835	129.07304	NR_033228	0.002	1.44	***E030024N20Rik***	peptidylprolyl isomerase A pseudogene 8	
55.95613	79.1136	63.943695	80.61627	NM_008513///XR_3	0.003	1.44	***Lrp5***	low density lipoprotein receptor-related protein 5	0001702 // gastrulation with mouth forming second // inferred from genetic interaction///0001944
96.959724	144.30843	102.93956	131.93976	NM_001290374///N	0.003	1.44	***Camk1d***	calcium/calmodulin-dependent protein kinase ID///Mus musculus calcium/calmodulin-dependent protein kin	0006468 // protein phosphorylation // not recorded///0006954 // inflammatory response // inferre
240.132	364.36948	373.0754	511.6522	NM_019571///NR_1	0.001	1.44	***Tspan5***	tetraspanin 5	0045747 // positive regulation of Notch signaling pathway // not recorded///0051604 // protein ma
58.78554	82.877174	79.60454	80.72896	NM_001145920///N	0.007	1.44	***Runx2***	runt related transcription factor 2	0001501 // skeletal system development // inferred from mutant phenotype///0001649 // osteobl
128.53464	193.86139	74.97245	108.28588	NM_029617///XM_C	0.003	1.44	***Casc5***	cancer susceptibility candidate 5	0007049 // cell cycle // inferred from electronic annotation///0007059 // chromosome segregation
108.6009	162.08821	148.47493	120.50201	NM_001081128///XI	0.002	1.43	***Mtr***	5-methyltetrahydrofolate-homocysteine methyltransferase	0006479 // protein methylation // not recorded///0006555 // methionine metabolic process // not
20.343548	27.066057	26.439373	34.410282	NM_001252525///N	0.002	1.43	***Cpeb1***	cytoplasmic polyadenylation element binding protein 1	0006397 // mRNA processing // inferred from electronic annotation///0006417 // regulation of trai
33.69915	45.400394	22.629398	34.161465	NM_001081280///N	0.005	1.42	***Nlrc3***	NLR family, CARD domain containing 3	0007249 // I-kappaB kinase/NF-kappaB signaling // not recorded///0007249 // I-kappaB kinase/NF-
230.39433	343.12134	466.976	559.3078	NM_010755///XM_C	0.005	1.41	***Maff***	v-maf musculoaponeurotic fibrosarcoma oncogene family, protein F (avian)	0001701 // in utero embryonic development // inferred from genetic interaction///0006351 // tran
1920.3824	2765.61	2477.2646	3721.227	NM_010515	0.006	1.41	***Igf2r***	insulin-like growth factor 2 receptor	0001889 // liver development // not recorded///0006810 // transport // inferred from electronic ai
498.26935	732.86304	308.37607	466.8467	NM_025995	0.009	1.41	***Fbxo5***	F-box protein 5	0001556 // oocyte maturation // inferred from direct assay///0001556 // oocyte maturation // infe
110.15724	160.86676	88.89565	112.38599	NM_001139509///N	0.003	1.41	***Nr4a2***	nuclear receptor subfamily 4, group A, member 2	0001666 // response to hypoxia // inferred from mutant phenotype///0001764 // neuron migration
67.21004	93.98363	74.082115	79.895386	NM_181584	0.001	1.40	***Gab3***	growth factor receptor bound protein 2-associated protein 3	0030225 // macrophage differentiation // inferred from direct assay
120.54283	176.19016	155.63914	176.87526	NM_001033445	0.007	1.40	***Garem***	GRB2 associated, regulator of MAPK1	0007173 // epidermal growth factor receptor signaling pathway // not recorded///0008284 // posit
18.478449	24.167439	22.991898	24.761549	NR_027799	0.009	1.40	***Tbrg3***	transforming growth factor beta regulated gene 3	
1700.2263	2398.7046	1773.6273	2308.3413	NM_144788///XM_C	0.002	1.40	***Hectd1***	HECT domain containing 1	0001843 // neural tube closure // inferred from mutant phenotype///0016567 // protein ubiquitina
634.7859	914.78436	806.3221	716.2482	NM_010574///XM_C	0.009	1.39	***Irx2***	Iroquois related homeobox 2 (Drosophila)	0001656 // metanephros development // inferred from expression pattern///0006355 // regulatior
37.547443	48.687748	36.727787	52.632854	NM_008069///XM_C	0.006	1.37	***Gabrb1***	gamma-aminobutyric acid (GABA) A receptor, subunit beta 1	0006810 // transport // inferred from electronic annotation///0006811 // ion transport // inferred
30.193584	39.033688	51.984655	73.03613	NR_037986	0.004	1.37	***Gm16880***	predicted gene, 16880	
26.211935	33.26756	20.40155	28.361856	NM_009129///XM_C	0.003	1.37	***Scg2***	secretogranin II	0000165 // MAPK cascade // not recorded///0000165 // MAPK cascade // inferred from sequence
155.71693	224.41743	301.37192	276.14014	NM_007962///XM_C	0.005	1.36	***Mpzl2***	myelin protein zero-like 2	0007155 // cell adhesion // inferred from electronic annotation///0016337 // single organismal cell
304.30743	431.4746	343.19257	380.7025	NM_175179	0.007	1.35	***Amer1***	APC membrane recruitment 1	0001822 // kidney development // inferred from mutant phenotype///0016055 // Wnt signaling pa
29.832485	37.8058	31.621458	33.00762		0.006	1.35	***D2Ertd127e***	DNA segment, Chr 2, ERATO Doi 127, expressed	
176.1112	247.93913	411.68637	526.9774	NM_008416	0.003	1.33	***Junb***	jun B proto-oncogene	0001570 // vasculogenesis // inferred from mutant phenotype///0001649 // osteoblast differentiat
32.19717	40.597176	30.035881	34.680634	NM_026642	0.007	1.33	***Trmt12***	tRNA methyltranferase 12	0008033 // tRNA processing // inferred from electronic annotation///0032259 // methylation // inf
25.756802	31.86897	21.770391	23.634647	NM_001025581///XI	0.009	1.33	***Kcnc2***	potassium voltage gated channel, Shaw-related subfamily, member 2	0001508 // action potential // inferred from mutant phenotype///0006810 // transport // inferred

[ICR, Air, PBS]	[ICR, Air, SFN](	[ICR, O2, PBS](	[ICR, O2, SFN](	RefSeq Transcript ID	p	FC Air-PBS:Air-SFN	*Gene Symbol*	Gene Title	Gene Ontology
250.47769	351.10553	385.72855	523.9787	NM_019570///XM_C	0.007	1.33	***Rev1***	REV1 homolog (S. cerevisiae)	0006260 // DNA replication // inferred from electronic annotation///0006261 // DNA-dependent D
301.80273	418.89847	454.75003	478.62057	NM_011150///XM_C	0.005	1.32	***Lgals3bp***	lectin, galactoside-binding, soluble, 3 binding protein	0006898 // receptor-mediated endocytosis // inferred from sequence or structural similarity///000
204.62753	287.6124	214.9759	330.38858	NM_028013///XM_C	0.004	1.32	***Endod1***	endonuclease domain containing 1	0008152 // metabolic process // inferred from electronic annotation///0090305 // nucleic acid pho
227.09082	318.25958	104.60873	192.18773	NM_001033331///N	0.004	1.32	***Gas2l3***	growth arrest-specific 2 like 3	0000226 // microtubule cytoskeleton organization // not recorded///0007050 // cell cycle arrest //
44.12032	55.75253	58.89628	70.18821	NM_145838///XM_C	0.005	1.32	***St8sia6***	ST8 alpha-N-acetyl-neuraminide alpha-2,8-sialyltransferase 6	0001574 // ganglioside biosynthetic process // inferred from direct assay///0006486 // protein glyc
763.9995	1039.4465	821.5112	980.9611	NM_001001602///N	0.010	1.32	***Dab2ip***	disabled 2 interacting protein	0000122 // negative regulation of transcription from RNA polymerase II promoter // not recorded/,
151.4919	210.18614	245.40456	208.30515	NM_001083903///N	0.007	1.32	***Sbsn***	suprabasin	0005576 // extracellular region // inferred from electronic annotation///0005737 // cytoplasm // ir
163.18095	227.16814	176.77748	175.95743	NM_026551///XM_C	0.004	1.32	***Dcakd***	dephospho-CoA kinase domain containing	0015937 // coenzyme A biosynthetic process // inferred from electronic annotation///0016310 // p
27.693193	34.011208	14.233391	19.101377	NR_024720	0.006	1.31	***2700099C18Rik***	NDC80 homolog, kinetochore complex component pseudogene	
334.70303	459.27002	367.51053	524.10925	NM_172771///XM_C	0.007	1.31	***Dmxl2***	Dmx-like 2	0005515 // protein binding // inferred from electronic annotation///0017137 // Rab GTPase binding
196.89948	272.05154	185.8761	346.56134	NM_001170853///XI	0.009	1.31	***Mndal///Ifi204///Ifi205/,***	myeloid nuclear differentiation antigen like///interferon activated gene 204///interferon activated gene 205/	0030308 // negative regulation of cell growth // inferred from mutant phenotype///0035458 // cell
893.774	1192.8127	2710.3591	1236.5773	NM_018857///XM_C	0.008	1.31	***Msln***	mesothelin	0007155 // cell adhesion // inferred from electronic annotation///0031016 // pancreas developme
549.282	746.8871	615.7252	833.2459	NM_153178	0.008	1.31	***Ago2***	argonaute RISC catalytic subunit 2	0006351 // transcription, DNA-templated // inferred from electronic annotation///0006355 // regu
213.62871	296.5488	215.08699	266.39948	NM_198035	0.006	1.31	***Zbtb39***	zinc finger and BTB domain containing 39	0003676 // nucleic acid binding // inferred from electronic annotation///0005515 // protein binding
160.46207	220.5536	441.01245	337.7131	NM_001083894///N	0.008	1.30	***Liph***	lipase, member H	0006629 // lipid metabolic process // inferred from electronic annotation///0016042 // lipid catabo
501.0726	676.94775	485.26706	606.8007	NM_001122730///N	0.007	1.30	***Tnrc18***	trinucleotide repeat containing 18	0003677 // DNA binding // inferred from electronic annotation///0003682 // chromatin binding // i
55.226788	70.06314	70.15251	85.027565	XR_105200///XR_10	0.010	1.30	***BC035532///Gm17491***	Mus musculus, clone IMAGE:5367487, mRNA.///predicted gene, 17491	
206.10951	282.24988	240.04082	364.49112	NM_029639	0.004	1.30	***Plet1***	placenta expressed transcript 1	0001953 // negative regulation of cell-matrix adhesion // inferred from mutant phenotype///00301
501.13324	675.79376	576.03375	685.29565	NM_001080548///N	0.006	1.29	***Usp6nl***	USP6 N-terminal like	0007030 // Golgi organization // not recorded///0032313 // regulation of Rab GTPase activity // inf
96.074905	126.52572	159.44437	143.65504	NM_001177752///N	0.009	1.28	***Pfkfb3***	6-phosphofructo-2-kinase/fructose-2,6-biphosphatase 3	0006000 // fructose metabolic process // inferred from electronic annotation///0006003 // fructos
31.6456	38.039135	33.43079	31.33232	NR_045294	0.008	1.27	***Gm4285***	predicted gene 4285	
298.94254	396.924	427.09717	403.13385	NM_011212///XM_C	0.009	1.27	***Ptpre***	protein tyrosine phosphatase, receptor type, E	0006470 // protein dephosphorylation // inferred from electronic annotation///0007185 // transme
294.51923	392.93863	367.21036	450.61218	NM_001136104///N	0.010	1.27	***Abl2***	v-abl Abelson murine leukemia viral oncogene 2 (arg, Abelson-related gene)	0006468 // protein phosphorylation // inferred from direct assay///0007015 // actin filament orgar
99.18277	129.61693	88.33103	116.8694	NM_145628///XM_C	0.009	1.27	***Usp11***	ubiquitin specific peptidase 11	0006508 // proteolysis // not recorded///0006511 // ubiquitin-dependent protein catabolic proces:
510.08087	672.6944	593.1886	537.6023	NM_001145813///N	0.006	1.27	***Elf5***	E74-like factor 5	0006351 // transcription, DNA-templated // inferred from electronic annotation///0006355 // regu
72.91295	92.655815	76.369606	76.22667	NM_022983	0.009	1.27	***Lpar3***	lysophosphatidic acid receptor 3	0000187 // activation of MAPK activity // inferred from direct assay///0007165 // signal transductic
25.381077	29.784544	24.001633	26.74695	XR_378441///XR_40	0.008	1.26	***Sox6os***	SRY (sex determining region Y)-box 6, opposite strand	
312.76343	408.10086	488.70282	568.8412	NM_010756///XM_C	0.010	1.25	***Mafg***	v-maf musculoaponeurotic fibrosarcoma oncogene family, protein G (avian)	0000122 // negative regulation of transcription from RNA polymerase II promoter // inferred from
176.90018	148.58652	172.05162	162.12796	NM_212483///XM_C	0.010	-1.24	***Krt42***	keratin 42	0005198 // structural molecule activity // inferred from electronic annotation
112.08529	91.94309	105.989075	93.89197	NM_015808	0.009	-1.25	***Krtap5-1***	keratin associated protein 5-1	0005882 // intermediate filament // inferred from electronic annotation///0045095 // keratin filam
55.690758	42.86803	64.70021	59.890446	NM_183087///XM_C	0.010	-1.25	***Fam189a1***	family with sequence similarity 189, member A1	0016020 // membrane // inferred from electronic annotation///0016021 // integral component of
248.18642	208.43869	228.58908	211.73468	NM_175175	0.009	-1.25	***Plekhf2***	pleckstrin homology domain containing, family F (with FYVE domain) member 2	0006810 // transport // inferred from electronic annotation///0015031 // protein transport // infer
75.543655	59.731426	70.21141	69.80537	NM_001256005///N	0.009	-1.25	***Gbp4///Gbp8***	guanylate binding protein 4///guanylate-binding protein 8	0000122 // negative regulation of transcription from RNA polymerase II promoter // inferred from
1658.202	1363.8546	1565.1398	1253.9458	NM_001110831///N	0.008	-1.25	***Dnpep***	aspartyl aminopeptidase	0006508 // proteolysis // inferred from electronic annotation
108.12305	88.27646	98.52483	83.576065	NM_019506	0.010	-1.25	***Gdf2***	growth differentiation factor 2	0001525 // angiogenesis // not recorded///0001569 // patterning of blood vessels // inferred from
24.755665	18.391495	23.54275	17.694609	NM_008103	0.010	-1.25	***Gcm1***	glial cells missing homolog 1 (Drosophila)	0006351 // transcription, DNA-templated // inferred from electronic annotation///0006355 // regu
420.97266	351.99164	243.6928	215.2877	NM_025566	0.010	-1.26	***Tnfaip8l1***	tumor necrosis factor, alpha-induced protein 8-like 1	
94.800545	75.86157	94.42467	85.864525	NM_172794///XM_C	0.007	-1.26	***Zfp454***	zinc finger protein 454	0006355 // regulation of transcription, DNA-templated // not recorded
90.67854	72.077896	79.34672	71.210754	NM_026419///XR_4(	0.009	-1.26	***Cela3b///LOC102641402***	chymotrypsin-like elastase family, member 3B///uncharacterized LOC102641402	0006508 // proteolysis // inferred from electronic annotation
576.9163	479.62076	518.05316	464.18796	NM_133999	0.010	-1.26	***Fig4***	FIG4 homolog (S. cerevisiae)	0007033 // vacuole organization // inferred from mutant phenotype///0007626 // locomotory beh
127.84914	104.62007	107.02186	99.601685	NM_029383	0.010	-1.26	***Cldn22***	claudin 22	0016338 // calcium-independent cell-cell adhesion // inferred from sequence or structural similarit
647.3494	533.822	553.8348	532.7884	NM_025654///XM_C	0.007	-1.26	***Rdm1***	RAD52 motif 1	0006281 // DNA repair // inferred from electronic annotation///0006310 // DNA recombination // i
253.92201	210.80324	225.56322	194.11992	NM_010149///XM_C	0.008	-1.26	***Epor***	erythropoietin receptor	0003007 // heart morphogenesis // traceable author statement///0007165 // signal transduction /,
196.90826	162.69772	181.02365	147.56238	NM_001289545///N	0.009	-1.27	***Atxn7l2***	ataxin 7-like 2	
93.195366	73.56165	90.40237	72.39487	NM_016879	0.010	-1.27	***Krt85***	keratin 85	0005198 // structural molecule activity // inferred from electronic annotation
127.49215	103.226776	116.942314	112.266106	NM_011575	0.007	-1.27	***Tff3***	trefoil factor 3, intestinal	0043434 // response to peptide hormone // inferred from electronic annotation
17.175562	13.071379	16.109167	15.289264	NM_009216///XM_C	0.009	-1.27	***Sstr1***	somatostatin receptor 1	0007165 // signal transduction // inferred from electronic annotation///0007186 // G-protein coup
123.08723	99.16563	113.7869	87.01594	NM_001271472///N	0.007	-1.27	***Scube1***	signal peptide, CUB domain, EGF-like 1	0051260 // protein homooligomerization // not recorded
95.829056	75.649574	81.587975	72.080086	NM_027010	0.006	-1.27	***Crygf***	crystallin, gamma F	0001654 // eye development // inferred from mutant phenotype
72.24196	55.609127	58.23118	52.30706	NM_020281	0.010	-1.27	***Magea7-ps***	melanoma antigen, family A, 7, pseudogene	
105.112625	83.908676	100.90135	83.98092	NR_033644	0.006	-1.27	***Fut4-ps1***	fucosyltransferase 4, pseudogene 1	
135.38937	109.49695	122.37774	120.672905		0.006	-1.27	***Gm9817***	predicted gene 9817	
98.435875	77.79113	90.10433	82.813156	NM_001286013///N	0.008	-1.28	***Dlk2***	delta-like 2 homolog (Drosophila)	0045598 // regulation of fat cell differentiation // inferred from direct assay///0045746 // negative
203.02264	166.5764	198.47736	169.81013	NM_016758	0.008	-1.28	***Rgs14***	regulator of G-protein signaling 14	0006913 // nucleocytoplasmic transport // inferred from direct assay///0006913 // nucleocytoplasi
122.9893	98.98542	144.21889	144.48506	NM_025293///NR_0	0.007	-1.28	***Map2k3os***	mitogen-activated protein kinase kinase 3, opposite strand	
179.89563	146.35768	145.30615	129.71944	NM_001164504	0.009	-1.28	***Rnf165***	ring finger protein 165	0000209 // protein polyubiquitination // inferred from direct assay///0007409 // axonogenesis // ir
183.17429	148.62523	174.08365	147.92021	NM_029674///XM_C	0.005	-1.28	***Got1l1***	glutamic-oxaloacetic transaminase 1-like 1	0006520 // cellular amino acid metabolic process // inferred from electronic annotation///0009058
52.171646	38.944023	44.56744	37.63602	NM_001291003///N	0.007	-1.28	***Astl***	astacin-like metalloendopeptidase (M12 family)	0006508 // proteolysis // not recorded///0007155 // cell adhesion // inferred from mutant phenoty
38.804817	28.472347	32.544216	27.684437	NM_001113417///N	0.010	-1.29	***Thrb***	thyroid hormone receptor beta	0000122 // negative regulation of transcription from RNA polymerase II promoter // inferred from
158.63492	128.17188	114.1938	98.5476	NM_001014395///XI	0.007	-1.29	***Fbxw22***	F-box and WD-40 domain protein 22	0005515 // protein binding // inferred from electronic annotation
309.8575	253.10463	347.6294	367.98383	NM_001033332///N	0.007	-1.29	***Tmtc3***	transmembrane and tetratricopeptide repeat containing 3	0009791 // post-embryonic development // inferred from mutant phenotype///0010468 // regulat
219.79932	179.45233	271.2101	212.0827	NM_011659///XM_C	0.005	-1.29	***Tnfrsf4***	tumor necrosis factor receptor superfamily, member 4	0006954 // inflammatory response // inferred from mutant phenotype///0006954 // inflammatory
34.802086	25.539406	25.30799	21.920176	NM_010003	0.004	-1.29	***Cyp2c39***	cytochrome P450, family 2, subfamily c, polypeptide 39	0055114 // oxidation-reduction process // not recorded
173.29906	139.96109	162.00342	143.08072	NM_001164787///N	0.009	-1.29	***Sprr2a1///Sprr2a2///Sprr***	small proline-rich protein 2A1///small proline-rich protein 2A2///small proline-rich protein 2A3	0008360 // regulation of cell shape // traceable author statement///0008544 // epidermis develop
90.01554	69.47447	76.992645	62.95424	NM_138942	0.005	-1.29	***Dbh***	dopamine beta hydroxylase	0001816 // cytokine production // inferred from mutant phenotype///0001974 // blood vessel rem
102.199936	80.10488	86.653336	71.46167	NM_001286005///N	0.008	-1.29	***Abcg8***	ATP-binding cassette, sub-family G (WHITE), member 8	0006200 // ATP catabolic process // not recorded///0006810 // transport // inferred from electroni
1912.5457	1521.142	1483.6912	1196.3813	NM_001042451///N	0.006	-1.29	***Snca***	synuclein, alpha	0001774 // microglial cell activation // inferred from mutant phenotype///0001921 // positive regu
278.99896	227.83887	225.8744	223.39742	NM_153778///XR_3	0.008	-1.29	***Atoh8***	atonal homolog 8 (Drosophila)	0006351 // transcription, DNA-templated // inferred from electronic annotation///0006355 // regu
147.75407	118.622314	138.62436	103.58865	NM_001146024///N	0.005	-1.29	***Zfp444***	zinc finger protein 444	0006351 // transcription, DNA-templated // inferred from electronic annotation///0006355 // regu
92.82695	71.83231	85.08331	69.96313	NM_153166///XM_C	0.008	-1.29	***Cpne5***	copine V	0005515 // protein binding // inferred from electronic annotation
298.56296	242.8654	122.60571	131.46358	NM_027975///XM_C	0.004	-1.30	***Fam83d***	family with sequence similarity 83, member D	0007049 // cell cycle // inferred from electronic annotation///0007067 // mitotic nuclear division /,
140.08636	111.52555	123.66003	95.84841	NM_001277891///N	0.008	-1.30	***Fkbp6***	FK506 binding protein 6	0000413 // protein peptidyl-prolyl isomerization // inferred from direct assay///0006457 // protein
289.1875	235.82529	215.61014	244.2957	NM_011465///XR_3	0.010	-1.30	***Spta1***	spectrin alpha, erythrocytic 1	0002260 // lymphocyte homeostasis // inferred from mutant phenotype///0006779 // porphyrin-co
69.31573	52.203815	35.414585	38.15566	NM_029993	0.010	-1.30	***Mlana***	melan-A	0005515 // protein binding // inferred from electronic annotation
726.5355	578.12994	399.21215	448.62204	NM_023716///NR_0	0.005	-1.30	***Tubb2a-ps2///Tubb2b***	tubulin, beta 2a, pseudogene 2///tubulin, beta 2B class IIB	0001764 // neuron migration // not recorded///0006184 // GTP catabolic process // inferred from
119.47572	94.07049	107.52871	94.3088	NM_010594	0.006	-1.30	***Kap***	kidney androgen regulated protein	0005576 // extracellular region // inferred from electronic annotation///0005615 // extracellular sp
75.21571	56.938393	64.870735	60.996178	NM_001291009///N	0.004	-1.30	***Pla2g10***	phospholipase A2, group X	0006629 // lipid metabolic process // inferred from electronic annotation///0006644 // phospholip
105.26999	82.063446	99.39066	86.30274	NM_010359	0.005	-1.30	***Gstm3***	glutathione S-transferase, mu 3	0008152 // metabolic process // inferred from electronic annotation///0035690 // cellular respons
84.331116	64.2512	88.020134	83.82377	NM_177716	0.008	-1.30	***Ccdc184***	coiled-coil domain containing 184	0005737 // cytoplasm // not recorded
52.1203	38.33534	42.99889	35.81575	NM_013513///XM_C	0.009	-1.31	***Epb4.2***	erythrocyte protein band 4.2	0000902 // cell morphogenesis // inferred from mutant phenotype///0008360 // regulation of cell

[ICR, Air, PBS]	[ICR, Air, SFN](	[ICR, O2, PBS](	[ICR, O2, SFN](	RefSeq Transcript ID	p	FC Air-PBS:Air-SFN	*Gene Symbol*	Gene Title	Gene Ontology
212.21895	170.5844	174.9912	169.42062	NM_146257///XM_C	0.004	-1.31	***Slc29a4***	solute carrier family 29 (nucleoside transporters), member 4	0006810 // transport // inferred from electronic annotation///0015844 // monoamine transport //
167.34294	133.49031	145.18858	113.6582	NM_001159658///N	0.007	-1.31	***Aqp12***	aquaporin 12	0006810 // transport // inferred from electronic annotation
19.268366	13.963492	15.410205	16.685665	NM_001164682///N	0.004	-1.31	***Mpp4***	membrane protein, palmitoylated 4 (MAGUK p55 subfamily member 4)	0035418 // protein localization to synapse // inferred from mutant phenotype
854.76196	674.4786	843.9976	619.2106	NM_008026///XM_C	0.010	-1.31	***Fli1***	Friend leukemia integration 1	0006351 // transcription, DNA-templated // inferred from electronic annotation///0006355 // regu
309.1059	247.5186	313.42593	396.6182	NM_153143	0.005	-1.31	***Kctd11***	potassium channel tetramerisation domain containing 11	0007049 // cell cycle // inferred from electronic annotation///0007275 // multicellular organismal c
34.855915	25.014982	51.533577	30.060123	NM_001101482///N	0.010	-1.31	***Mrap2***	melanocortin 2 receptor accessory protein 2	0006112 // energy reserve metabolic process // inferred from mutant phenotype///0007631 // fee
1418.3018	1110.2058	1206.4363	1114.341	NM_018810///XM_C	0.009	-1.32	***Mkrn1***	makorin, ring finger protein, 1	0000209 // protein polyubiquitination // not recorded///0016567 // protein ubiquitination // inferr
159.04988	125.36458	166.0116	161.58946	NM_009086///XM_C	0.004	-1.32	***Polr1b***	polymerase (RNA) I polypeptide B	0006351 // transcription, DNA-templated // inferred from electronic annotation///0006360 // trans
71.58197	53.156208	68.10403	55.51835	NM_009215	0.004	-1.32	***Sst***	somatostatin	0001101 // response to acid // inferred from electronic annotation///0006972 // hyperosmotic resp
202.7817	161.63774	196.9261	174.1229	NM_033175///XM_C	0.003	-1.32	***Lce3c***	late cornified envelope 3C	0008544 // epidermis development // inferred from electronic annotation
89.825806	67.95884	86.30454	81.59401	NM_011564	0.006	-1.32	***Sry***	sex determining region of Chr Y	0000122 // negative regulation of transcription from RNA polymerase II promoter // inferred from g
98.94794	75.56101	85.00992	79.62151	NM_001037752	0.009	-1.32	***Defb45***	defensin beta 45	0006952 // defense response // inferred from electronic annotation///0042742 // defense respons
97.75643	74.36971	95.53492	83.86803	NM_013773	0.008	-1.32	***Tcl1b1***	T cell leukemia/lymphoma 1B, 1	
118.85292	92.06245	100.898254	99.16808	NM_013533///XM_C	0.004	-1.32	***Gpr162***	G protein-coupled receptor 162	0007165 // signal transduction // inferred from electronic annotation///0007186 // G-protein coup
59.832237	43.624027	56.743343	45.28395	NM_133364///NM_:	0.003	-1.32	***Prima1***	proline rich membrane anchor 1	0042135 // neurotransmitter catabolic process // inferred from electronic annotation///0051649 //
81.612	61.001537	68.537994	62.277225	NM_026094///XM_C	0.004	-1.32	***Atp8b3***	ATPase, class I, type 8B, member 3	0006200 // ATP catabolic process // inferred from mutant phenotype///0006810 // transport // inf
112.07336	86.23608	98.88991	77.975	NM_001101507	0.005	-1.32	***Clec2l***	C-type lectin domain family 2, member L	0030246 // carbohydrate binding // inferred from electronic annotation
234.62373	185.58629	203.69936	243.78697	NM_001162947///N	0.009	-1.33	***Nek3***	NIMA (never in mitosis gene a)-related expressed kinase 3	0006468 // protein phosphorylation // inferred from direct assay///0007049 // cell cycle // inferrec
223.89586	177.10966	209.33241	179.66913	NM_026335	0.008	-1.33	***Lce1h***	late cornified envelope 1H	0008544 // epidermis development // inferred from electronic annotation
400.4553	317.61584	124.556145	131.25037	NM_001172092///N	0.004	-1.33	***Depdc1a***	DEP domain containing 1a	0006351 // transcription, DNA-templated // inferred from electronic annotation///0006355 // regu
57.976376	41.63485	49.633217	43.989952	NM_001290138///N	0.005	-1.33	***Ccdc169***	coiled-coil domain containing 169	
119.15712	91.36323	101.499985	112.21039		0.007	-1.33	***Proscos***	proline synthetase co-transcribed, opposite strand	
1830.4143	1409.4471	1678.2566	1495.0209	NM_001033988///N	0.005	-1.34	***Ncoa4***	nuclear receptor coactivator 4	0005739 // mitochondrion // inferred from direct assay
634.9861	496.5077	607.4433	555.71094	NM_001159403///N	0.003	-1.34	***Nwd1***	NACHT and WD repeat domain containing 1	0000166 // nucleotide binding // inferred from electronic annotation///0005524 // ATP binding // i
46.89701	33.35833	38.454548	40.612354	NM_001146002///N	0.004	-1.34	***Txndc2***	thioredoxin domain containing 2 (spermatozoa)	0006457 // protein folding // inferred from direct assay///0006662 // glycerol ether metabolic proc
415.41763	327.161	414.8405	318.99298	NM_009362///XM_C	0.005	-1.34	***Tff1***	trefoil factor 1	0006950 // response to stress // inferred from electronic annotation///0008285 // negative regulat
74.270355	54.45309	65.70019	62.32263	NM_145227///XM_C	0.010	-1.34	***Oas2***	2'-5' oligoadenylate synthetase 2	0002376 // immune system process // inferred from electronic annotation///0006164 // purine nuc
40.116776	28.177563	34.936478	26.704489	NM_009611	0.009	-1.34	***Actl7a***	actin-like 7a	0005515 // protein binding // inferred from electronic annotation
257.62292	201.16614	233.79413	180.62936	NM_001195088///N	0.003	-1.34	***Tmc8***	transmembrane channel-like gene family 8	0001558 // regulation of cell growth // not recorded///0006810 // transport // inferred from electr
90.14916	66.56017	79.27893	66.88353	NM_177693///XM_C	0.004	-1.35	***Lim2***	lens intrinsic membrane protein 2	0002088 // lens development in camera-type eye // inferred from mutant phenotype///0043010 //
144.51654	111.13407	131.40585	112.64854	NM_029937///XM_C	0.005	-1.35	***Nup210l***	nucleoporin 210-like	0007286 // spermatid development // inferred from mutant phenotype///0060009 // Sertoli cell de
123.47595	93.72944	105.83267	86.591354	NM_027066	0.002	-1.35	***Tmem89***	transmembrane protein 89	0005634 // nucleus // not recorded///0016020 // membrane // inferred from electronic annotatior
126.65086	96.12836	117.36861	98.869774	NM_022427///XM_C	0.003	-1.35	***Gpr88***	G-protein coupled receptor 88	0007165 // signal transduction // inferred from electronic annotation///0007186 // G-protein coup
245.21848	190.1479	235.5229	183.3726	NM_026415	0.005	-1.35	***Cysrt1***	cysteine rich tail 1	0070062 // extracellular vesicular exosome // not recorded
92.37111	68.36415	86.19487	101.24989	NM_019577///XM_C	0.003	-1.35	***Ccl24***	chemokine (C-C motif) ligand 24	0001938 // positive regulation of endothelial cell proliferation // not recorded///0006935 // chemo
267.4976	207.83537	221.61696	206.91083	NM_033610///XM_C	0.003	-1.35	***Sncb***	synuclein, beta	0007268 // synaptic transmission // inferred from genetic interaction///0042417 // dopamine meta
154.00327	118.55258	146.33069	109.78352	NM_010381///XM_C	0.005	-1.35	***H2-Ea-ps***	histocompatibility 2, class II antigen E alpha, pseudogene	0002376 // immune system process // inferred from electronic annotation///0002504 // antigen pr
329.50635	256.1405	262.90204	209.00514	NM_009446///NM_(	0.004	-1.35	***Tuba3a///Tuba3b***	tubulin, alpha 3A///tubulin, alpha 3B	0006184 // GTP catabolic process // inferred from electronic annotation///0007017 // microtubule
570.63544	439.5892	718.77344	795.83356	NM_010240///NR_0	0.004	-1.35	***Ftl1///Gm20746///LOC10***	ferritin light chain 1///predicted gene, 20746///ferritin light chain 1-like///microRNA 692-1///microRNA 692-:	0006826 // iron ion transport // inferred from electronic annotation///0006879 // cellular iron ion l
120.8282	91.25962	117.8239	88.65962	NM_028799	0.007	-1.35	***Tgm5***	transglutaminase 5	0018149 // peptide cross-linking // inferred from electronic annotation
316.18216	246.53752	280.0087	225.70671	NM_007847	0.004	-1.35	***Defa-rs2***	defensin, alpha, related sequence 2	0006952 // defense response // inferred from electronic annotation///0032496 // response to lipop
114.57776	86.26772	93.530205	82.40197	NM_011139	0.005	-1.36	***Pou2f3***	POU domain, class 2, transcription factor 3	0006351 // transcription, DNA-templated // inferred from electronic annotation///0006355 // regu
768.80475	586.56995	713.0906	581.60895	NM_001037326///N	0.004	-1.36	***Cstf3***	cleavage stimulation factor, 3' pre-RNA, subunit 3	0006396 // RNA processing // inferred from electronic annotation///0006397 // mRNA processing /
23.973154	16.474997	23.403814	19.023779	NM_001271716///N	0.005	-1.36	***Mc2r***	melanocortin 2 receptor	0001890 // placenta development // inferred from electronic annotation///0007165 // signal transd
111.86112	83.63365	79.2544	69.56707	NM_054095///XM_C	0.002	-1.36	***Necab2***	N-terminal EF-hand calcium binding protein 2	0005509 // calcium ion binding // inferred from electronic annotation///0005515 // protein binding
458.6414	352.86316	424.442	353.3893	NM_011025///XM_C	0.005	-1.36	***Oxt***	oxytocin	0001975 // response to amphetamine // inferred from electronic annotation///0002027 // regulati
136.53804	103.31054	122.66202	98.57161	NM_010601///XM_C	0.004	-1.36	***Kcnh3***	potassium voltage-gated channel, subfamily H (eag-related), member 3	0000160 // phosphorelay signal transduction system // inferred from electronic annotation///0006
64.143814	45.74545	51.07201	46.519894	NM_175427///XM_C	0.002	-1.36	***Fam163b***	family with sequence similarity 163, member B	0016020 // membrane // inferred from electronic annotation///0016021 // integral component of
190.85114	145.67776	180.23357	133.79602	NM_001039049	0.003	-1.37	***Cox8c***	cytochrome c oxidase subunit VIIIc	1902600 // hydrogen ion transmembrane transport // inferred from electronic annotation
793.64496	602.3722	1253.9342	1009.8993	NM_027464///XM_C	0.008	-1.37	***Fam213a***	family with sequence similarity 213, member A	0045670 // regulation of osteoclast differentiation // not recorded///0055114 // oxidation-reductic
101.43361	74.699875	103.13095	89.84359	NM_177897///XR_3	0.002	-1.37	***B4galnt4***	beta-1,4-N-acetyl-galactosaminyl transferase 4	0008152 // metabolic process // not recorded
109.99309	81.30346	47.028076	54.925083	NM_010883	0.008	-1.38	***Ndp***	Norrie disease (pseudoglioma) (human)	0001890 // placenta development // inferred from mutant phenotype///0016055 // Wnt signaling p
65.868324	46.16065	49.529713	49.381584	NM_022888///NM_:	0.004	-1.38	***Folr4***	folate receptor 4 (delta)	0007155 // cell adhesion // inferred from mutant phenotype///0007338 // single fertilization // inf
50.97551	35.07701	43.97094	39.995914	NM_177448	0.004	-1.38	***Mogat2***	monoacylglycerol O-acyltransferase 2	0006071 // glycerol metabolic process // inferred from electronic annotation///0006629 // lipid me
27.947567	18.82351	20.317287	20.69878	NM_011996	0.003	-1.38	***Adh4***	alcohol dehydrogenase 4 (class II), pi polypeptide	0001523 // retinoid metabolic process // not recorded///0006066 // alcohol metabolic process // n
37.492348	25.617674	33.39885	27.986418	NM_001167879	0.002	-1.38	***Gareml***	GRB2 associated, regulator of MAPK1-like	
1077.6061	804.4449	967.66095	831.316	NM_026050///NM_:	0.005	-1.39	***Fam220a***	family with sequence similarity 220, member A	0005634 // nucleus // inferred from electronic annotation
44.142605	29.923456	35.93549	33.383488	NM_001171640	0.009	-1.39	***Actl10***	actin-like 10	
52.81152	36.34758	58.475872	50.886513	NM_021325	0.009	-1.39	***Cd200r1***	CD200 receptor 1	0005886 // plasma membrane // inferred from electronic annotation///0009897 // external side of
132.99223	98.174416	126.482025	98.58774	NM_147221///XM_C	0.003	-1.39	***Glis1***	GLIS family zinc finger 1	0000122 // negative regulation of transcription from RNA polymerase II promoter // inferred from e
85.20198	60.45209	77.21883	75.55018	NM_001290452///N	0.005	-1.40	***Mzf1***	myeloid zinc finger 1	0006351 // transcription, DNA-templated // inferred from electronic annotation///0006355 // regu
32.57386	21.949392	26.60658	22.348583	NM_021340///XM_C	0.006	-1.40	***Rgr***	retinal G protein coupled receptor	0006935 // chemotaxis // inferred from electronic annotation///0007165 // signal transduction // i
910.95795	672.1001	746.25885	507.74216	NM_146008	0.006	-1.40	***Tcp11l2***	t-complex 11 (mouse) like 2	
185.12082	137.07129	166.49597	140.58675	NM_001172136///N	0.003	-1.41	***Exog***	endo/exonuclease (5'-3'), endonuclease G-like	0000737 // DNA catabolic process, endonucleolytic // not recorded
164.9194	121.29307	159.21935	134.45238	NM_001033433	0.002	-1.41	***Tmem102***	transmembrane protein 102	0006915 // apoptotic process // inferred from electronic annotation///0007165 // signal transducti
243.08191	179.99983	216.03056	181.9311	NM_001081327///N	0.001	-1.41	***Hs3st2***	heparan sulfate (glucosamine) 3-O-sulfotransferase 2	0007623 // circadian rhythm // not recorded///0008152 // metabolic process // inferred from elect
266.2963	195.99948	226.59492	196.62822	NM_008701///XM_C	0.004	-1.42	***Nkx2-9***	NK2 homeobox 9	0006351 // transcription, DNA-templated // not recorded///0006355 // regulation of transcription,
729.0145	531.8995	741.53864	542.4106	NM_008866///XM_C	0.009	-1.42	***Lypla1***	lysophospholipase 1	0002084 // protein depalmitoylation // inferred from mutant phenotype///0006629 // lipid metabo
28.390142	18.53598	22.333838	19.802942	NM_027072	0.006	-1.43	***Plac8l1***	PLAC8-like 1	
1052.9667	758.3665	981.7409	635.7656	NM_013772///XM_C	0.001	-1.44	***LOC100041483///Tcl1b3***	protein TCL1B3-like///T cell leukemia/lymphoma 1B, 3	
105.735435	73.731705	115.80032	76.45401	NM_001099632	0.001	-1.45	***Rnf39***	ring finger protein 39	0005515 // protein binding // inferred from electronic annotation///0008270 // zinc ion binding //
417.6363	304.8422	316.3287	257.26706	NM_009338///NM_:	0.001	-1.45	***Acat2///Acat3***	acetyl-Coenzyme A acetyltransferase 2///acetyl-Coenzyme A acetyltransferase 3	0008152 // metabolic process // inferred from electronic annotation///0008610 // lipid biosyntheti
2521.7776	1759.38	2140.292	1843.2498	NM_001142952///XI	0.010	-1.45	***Fam46c***	family with sequence similarity 46, member C	
332.6392	240.5345	273.74942	220.21518	NM_001113246///N	0.009	-1.46	***Chn1***	chimerin 1	0007165 // signal transduction // inferred from electronic annotation///0007399 // nervous systen
123.77513	86.37831	112.16693	94.96171		0.002	-1.46	***AI043046***	expressed sequence AI043046	
35.13173	22.373497	30.004507	24.36192	NM_008177	0.002	-1.48	***Grpr***	gastrin releasing peptide receptor	0007165 // signal transduction // inferred from electronic annotation///0007186 // G-protein coup
118.34083	81.60523	101.33044	91.47962	NM_021380///XM_C	0.003	-1.48	***Il20***	interleukin 20	0007262 // STAT protein import into nucleus // inferred from sequence or structural similarity///00
60.153282	38.781097	45.226604	39.583744	NM_175525///XM_C	0.002	-1.49	***Ssu2***	ssu-2 homolog (C. elegans)	0031072 // heat shock protein binding // inferred from electronic annotation///0051082 // unfolde
36.701405	23.111998	31.469898	24.574776	NM_001177438///N	0.005	-1.50	***1700055N04Rik///Aldh3b***	RIKEN cDNA 1700055N04 gene///aldehyde dehydrogenase 3 family, member B2///aldehyde dehydrogenase !	0006081 // cellular aldehyde metabolic process // inferred from electronic annotation///0008152 /
963.528	654.9564	940.2358	716.4789	NM_009433	0.010	-1.52	***Tspyl1***	testis-specific protein, Y-encoded-like 1	0006334 // nucleosome assembly // inferred from electronic annotation

[ICR, Air, PBS]	[ICR, Air, SFN](ı	[ICR, O2, PBS](	[ICR, O2, SFN](ı	RefSeq Transcript ID	p	FC Air-PBS:Air-SFN	*Gene Symbol*	Gene Title	Gene Ontology
152.2644	102.52239	134.26436	104.37595	NM_029667	0.001	-1.52	***Lce1i***	late cornified envelope 1I	0008544 // epidermis development // inferred from electronic annotation
101.94964	66.872055	81.4693	76.863785	NM_001136237///N	0.002	-1.52	***Slc39a5***	solute carrier family 39 (metal ion transporter), member 5	0006810 // transport // inferred from electronic annotation///0006811 // ion transport // inferred
90.53483	58.597626	54.37917	45.056515	NM_019538///XM_C	0.004	-1.53	***Plac1***	placental specific protein 1	0001890 // placenta development // inferred from expression pattern///0001890 // placenta devel
66.74981	42.613354	57.500587	50.571495	NM_013797///XM_C	0.004	-1.53	***Slco1a1***	solute carrier organic anion transporter family, member 1a1	0006810 // transport // inferred from electronic annotation///0006811 // ion transport // inferred
604.0621	408.2919	385.23013	427.03336	NM_145587///XM_C	0.009	-1.54	***Sbk1***	SH3-binding kinase 1	0006468 // protein phosphorylation // inferred from electronic annotation///0016310 // phosphor
197.70314	132.63347	421.5775	396.7446	NM_010501	0.008	-1.56	***Ifit3***	interferon-induced protein with tetratricopeptide repeats 3	0002376 // immune system process // inferred from electronic annotation///0008285 // negative r
2791.4875	1792.2322	2109.443	1831.4321	NM_007563///XM_C	0.006	-1.56	***Bpgm***	2,3-bisphosphoglycerate mutase	0006096 // glycolytic process // inferred from electronic annotation///0008152 // metabolic proces
124.81598	79.31379	87.99361	72.32608	NM_027292	0.003	-1.60	***Tspo2***	translocator protein 2	0006810 // transport // inferred from electronic annotation
24.214493	13.980096	20.205318	16.6023	NM_029536	0.001	-1.64	***Gpr165***	G protein-coupled receptor 165	0007165 // signal transduction // inferred from electronic annotation///0007186 // G-protein coup

Table S2. Prenatal sulforaphane-altered 918 lung genes in postnatal air-exposed *Nrf2* $^{-/-}$ mice (n=918, moderated T-Test, P<=0.01)

RIKEN cDNA and predicted genes excluded. Fold Change (FC): Blue (-) - fold lower in SFN than PBS, Red - fold higher in SFN than PBS.

[Nrf2-KO, Air, PBS]	[Nrf2-KO, Air, SFN]	RefSeq Transcript ID	p	FC PBS:SFN	Gene Symbol	Gene Title	Gene Ontology
248.11972	666.50073	NM_001030294///XN	0.007	2.73	***Olfm4***	olfactomedin 4	0007155 // cell adhesion // inferred from electronic annotation///0043124 // negative regulation of I-kappaB kinas
484.7015	1256.8951	NR_003513	0.000	2.66	***Neat1***	nuclear paraspeckle assembly transcript 1 (non-protein coding)///---	0030575 // nuclear body organization // inferred from mutant phenotype///0043954 // cellular component mainte
231.03038	564.68445	NM_144513///NR_0C	0.001	2.46	***Meg3***	maternally expressed 3///---	0001701 // in utero embryonic development // inferred from mutant phenotype///0001889 // liver development /
919.72174	2061.3145	NM_001082543///NN	0.009	2.33	***BC100530///Stfa1***	cDNA sequence BC100530///stefin A1	0010466 // negative regulation of peptidase activity // inferred from electronic annotation///0010951 // negative
30.045393	63.700813	NM_139134///XM_0	0.000	2.04	***Chodl***	chondrolectin	0005540 // hyaluronic acid binding // inferred from sequence or structural similarity///0030246 // carbohydrate bi
47.849865	102.033646	NM_011410///XM_0	0.001	2.02	***Slfn4***	schlafen 4	0005524 // ATP binding // inferred from electronic annotation
491.72003	962.13226	NM_023665	0.000	2.00	***Rsrp1***	arginine/serine rich protein 1	
1075.8644	2057.7217	NM_001042634///NN	0.000	1.98	***Clk1***	CDC-like kinase 1	0006468 // protein phosphorylation // not recorded///0016310 // phosphorylation // inferred from electronic ann
30.43497	62.552834	NM_001110505///NN	0.000	1.98	***Amy1///Rnpc3***	amylase 1, salivary///RNA-binding region (RNP1, RRM) containing 3	0005975 // carbohydrate metabolic process // inferred from electronic annotation///0008152 // metabolic proces
83.35101	168.04236	NM_001037743///XN	0.002	1.96	***4921506M07Rik///LOC102642336***	RIKEN cDNA 4921506M07 gene///tetratricopeptide repeat protein 6-like	0005515 // protein binding // inferred from electronic annotation
521.4066	989.1873	NM_018856///NM_2	0.000	1.93	***Ccnl2***	cyclin L2	0000079 // regulation of cyclin-dependent protein serine/threonine kinase activity // inferred from electronic anno
48.200565	94.03042	NM_178278///XM_0	0.001	1.84	***Caps2***	calcyphosphine 2	0005509 // calcium ion binding // inferred from electronic annotation///0046872 // metal ion binding // inferred fi
349.55603	617.0719	NM_144847///XM_0	0.004	1.78	***Nrbp2***	nuclear receptor binding protein 2	0006468 // protein phosphorylation // inferred from electronic annotation///0007399 // nervous system developn
230.99739	407.30475	NM_009903	0.005	1.77	***Cldn4***	claudin 4	0007565 // female pregnancy // inferred from electronic annotation///0016338 // calcium-independent cell-cell ac
54.208763	100.84891	NR_015505///NR_02	0.000	1.76	***Firre***	functional intergenic repeating RNA element	
163.32114	285.9527	NR_015531	0.006	1.75	***Dancr***	differentiation antagonizing non-protein coding RNA	
51.093113	94.192444	NM_001037743///XN	0.002	1.73	***4921506M07Rik///LOC102642336***	RIKEN cDNA 4921506M07 gene///tetratricopeptide repeat protein 6-like	0005515 // protein binding // inferred from electronic annotation
47.613567	86.71949	NM_007749	0.005	1.73	***Cox7c***	cytochrome c oxidase subunit VIIc	1902600 // hydrogen ion transmembrane transport // inferred from electronic annotation
360.36276	616.44116	NM_172779///XM_0	0.000	1.72	***Ddx26b***	DEAD/H (Asp-Glu-Ala-Asp/His) box polypeptide 26B	
406.63916	691.392	NM_001159632///NN	0.000	1.72	***Atp6v1c2***	ATPase, H+ transporting, lysosomal V1 subunit C2	0006200 // ATP catabolic process // inferred from direct assay///0006810 // transport // inferred from electronic a
33.014507	59.086693	NM_172864	0.010	1.72	***Wdr63***	WD repeat domain 63	0005515 // protein binding // inferred from electronic annotation
314.8336	536.64435	NM_023755///XM_0	0.003	1.72	***Tfcp2l1***	transcription factor CP2-like 1	0000122 // negative regulation of transcription from RNA polymerase II promoter // inferred from direct assay///0
396.84558	672.9599	NM_001290535///NN	0.000	1.71	***Ogt***	O-linked N-acetylglucosamine (GlcNAc) transferase (UDP-N-acetylglucosamine:poly	0006110 // regulation of glycolytic process // not recorded///0006486 // protein glycosylation // inferred from ele
1034.6279	1705.6106	NM_010821///XM_0	0.002	1.70	***Mpeg1***	macrophage expressed gene 1	0016020 // membrane // inferred from electronic annotation///0016021 // integral component of membrane // in
82.523415	145.46419	NM_175430///XM_0	0.007	1.68	***Ccdc40***	coiled-coil domain containing 40	0001947 // heart looping // not recorded///0003341 // cilium movement // inferred from mutant phenotype///00
118.005165	203.55351	NR_029382///NR_02	0.000	1.68	***Mir17hg///Mir92-1***	Mir17 host gene 1 (non-protein coding)///microRNA 92-1	0001701 // in utero embryonic development // inferred from genetic interaction///0002329 // pre-B cell differenti
185.878	308.80588	NM_007714///XM_0	0.000	1.65	***Clk4***	CDC like kinase 4	0006468 // protein phosphorylation // inferred from electronic annotation///0016310 // phosphorylation // inferr
433.29846	705.4861	NM_001111316///NN	0.000	1.65	***Ptprc***	protein tyrosine phosphatase, receptor type, C	0000187 // activation of MAPK activity // inferred from mutant phenotype///0001915 // negative regulation of T c
863.275	1382.106	NM_025951///NM_0	0.003	1.65	***Pi4k2b***	phosphatidylinositol 4-kinase type 2 beta	0016310 // phosphorylation // inferred from electronic annotation///0046854 // phosphatidylinositol phosphoryla
168.4762	278.56406	NM_026985///XM_0	0.003	1.64	***Mcemp1***	mast cell expressed membrane protein 1	0016020 // membrane // inferred from electronic annotation///0016021 // integral component of membrane // in
156.02037	253.80077	NM_001252401///NN	0.001	1.61	***Tle2***	transducin-like enhancer of split 2, homolog of Drosophila E(spl)	0006351 // transcription, DNA-templated // inferred from electronic annotation///0006355 // regulation of transcr
18.54299	29.746069	NM_010870///XM_0	0.009	1.60	***Naip5***	NLR family, apoptosis inhibitory protein 5	0002376 // immune system process // inferred from electronic annotation///0006915 // apoptotic process // infer
87.88481	147.03099	NM_001099633///XN	0.004	1.60	***Dnah9***	dynein, axonemal, heavy chain 9	0006200 // ATP catabolic process // inferred from electronic annotation///0007018 // microtubule-based moveme
5237.8335	8342.986	NM_001079694///NN	0.002	1.60	***Srsf5***	serine/arginine-rich splicing factor 5	0006351 // transcription, DNA-templated // inferred from electronic annotation///0006355 // regulation of transcr
224.72978	357.68903	NM_001025566///NN	0.001	1.59	***Chka***	choline kinase alpha	0006629 // lipid metabolic process // inferred from electronic annotation///0006646 // phosphatidylethanolamine
152.43263	243.70963	NM_010877///XM_0	0.003	1.58	***Ncf2***	neutrophil cytosolic factor 2	0006742 // NADP catabolic process // not recorded///0006801 // superoxide metabolic process // not recorded///
333.5353	517.65094	NM_026110///XR_38	0.000	1.57	***Paxbp1***	PAX3 and PAX7 binding protein 1	0006351 // transcription, DNA-templated // inferred from electronic annotation///0006355 // regulation of transcr
319.89084	494.23187	NM_009841	0.002	1.55	***Cd14***	CD14 antigen	0002237 // response to molecule of bacterial origin // inferred from direct assay///0002376 // immune system pro
353.4351	543.24054	NR_027008///NR_02	0.001	1.55	***Gt(ROSA)26Sor***	gene trap ROSA 26, Philippe Soriano	
173.75519	271.15042	NM_001159299///NN	0.003	1.55	***Itih4***	inter alpha-trypsin inhibitor, heavy chain 4	0006953 // acute-phase response // inferred from electronic annotation///0010466 // negative regulation of pepti
1384.8971	2067.0303	NM_007705///XM_0	0.001	1.54	***Cirbp***	cold inducible RNA binding protein	0006950 // response to stress // inferred from electronic annotation///0009409 // response to cold // inferred fro
540.30457	814.6418	NR_030711	0.000	1.53	***Mir22hg***	Mir22 host gene (non-protein coding)	
12.886861	19.568853	NM_001085518///NN	0.005	1.52	***Gm14085///Slc28a2***	predicted gene 14085///solute carrier family 28 (sodium-coupled nucleoside transp	0001895 // retina homeostasis // not recorded///0006810 // transport // inferred from electronic annotation///00
266.34436	405.37885	NM_177268///XM_0	0.001	1.52	***Ankrd16***	ankyrin repeat domain 16	0005515 // protein binding // inferred from electronic annotation
4281.399	6319.4473	NM_001123037///NN	0.002	1.51	***Eif4a2***	eukaryotic translation initiation factor 4A2	0006200 // ATP catabolic process // inferred from electronic annotation///0006412 // translation // inferred from
965.78796	1409.3214	NM_023842///XM_0	0.004	1.51	***Dsp***	desmoplakin	0002934 // desmosome organization // inferred from mutant phenotype///0003223 // ventricular compact myoca
14.523185	21.862753	NR_028555	0.005	1.51	***Snord89***	small nucleolar RNA, C/D box 89	
47.770977	75.73093	NM_030110///XM_0	0.002	1.51	***Micu3***	mitochondrial calcium uptake family, member 3	0005509 // calcium ion binding // inferred from electronic annotation///0046872 // metal ion binding // inferred fi
1796.5664	2621.1614	NM_001271538///NN	0.001	1.51	***Myh14***	myosin, heavy polypeptide 14	0003009 // skeletal muscle contraction // not recorded///0006200 // ATP catabolic process // inferred from direct
146.00955	222.34581	NM_001163489///NN	0.001	1.50	***Sema4a***	sema domain, immunoglobulin domain (Ig), transmembrane domain (TM) and short	0001525 // angiogenesis // inferred from electronic annotation///0002292 // T cell differentiation involved in imm
324.76068	484.28998	NM_001160378///NN	0.004	1.50	***Fam46a***	family with sequence similarity 46, member A	0044822 // poly(A) RNA binding // not recorded
59.42976	95.02456	NM_183276	0.000	1.50	***Nbeal2***	neurobeachin-like 2	0007596 // blood coagulation // inferred from mutant phenotype///0030220 // platelet formation // not recorded
444.4374	659.4524	NM_181595///XM_0	0.003	1.50	***Ppp1r9a***	protein phosphatase 1, regulatory (inhibitor) subunit 9A	0007015 // actin filament organization // inferred from physical interaction///0019722 // calcium-mediated signali
37.036602	57.648235	NM_001244617///NN	0.005	1.49	***B4galnt1***	beta-1,4-N-acetyl-galactosaminyl transferase 1	0001574 // ganglioside biosynthetic process // inferred from genetic interaction///0001574 // ganglioside biosynth
59.582714	94.57021	NR_033641///NR_11	0.002	1.49	***Crnde***	colorectal neoplasia differentially expressed (non-protein coding)	
221.61835	330.93765	NM_016805///XR_37	0.002	1.49	***AI503316///Hnrnpu***	expressed sequence AI503316///heterogeneous nuclear ribonucleoprotein U	0001649 // osteoblast differentiation // not recorded///0006397 // mRNA processing // inferred from electronic ar
265.57697	395.9591	NM_001163507///NN	0.006	1.49	***Mmrn1***	multimerin 1	0005509 // calcium ion binding // inferred from electronic annotation///0005515 // protein binding // inferred fro
558.2761	815.70386	NM_020008	0.007	1.49	***Clec7a***	C-type lectin domain family 7, member a	0001878 // response to yeast // inferred from direct assay///0001879 // detection of yeast // inferred from direct
67.38696	106.11947	NM_001037138///NN	0.000	1.49	***Cd300lb***	CD300 antigen like family member B	0002376 // immune system process // inferred from electronic annotation///0002446 // neutrophil mediated imm
42.761967	66.288	NM_001033371///NN	0.004	1.48	***Lrrc36***	leucine rich repeat containing 36	
1258.657	1816.5428	NM_008259///XM_0	0.006	1.48	***Foxa1***	forkhead box A1	0000122 // negative regulation of transcription from RNA polymerase II promoter // inferred from mutant phenoty
525.38477	761.0276	NM_182995///XM_0	0.001	1.48	***Ccp110***	centriolar coiled coil protein 110	0007099 // centriole replication // not recorded///0030030 // cell projection organization // inferred from electror
39.66738	61.26164	NM_001081330///NN	0.004	1.47	***Dnah2***	dynein, axonemal, heavy chain 2	0001539 // cilium or flagellum-dependent cell motility // inferred from electronic annotation///0006200 // ATP cat
138.30579	208.84721	NM_145931///NR_02	0.001	1.47	***Zc3h7a***	zinc finger CCCH type containing 7 A	0005515 // protein binding // inferred from electronic annotation///0044822 // poly(A) RNA binding // not recorde
88.47535	137.02032	NM_001048148///NN	0.001	1.47	***Lime1///Zgpat***	Lck interacting transmembrane adaptor 1///zinc finger, CCCH-type with G patch dor	0002376 // immune system process // inferred from electronic annotation///0006351 // transcription, DNA-templa
113.04274	172.02422	NM_001286037///NN	0.007	1.46	***Ncf1***	neutrophil cytosolic factor 1	0001878 // response to yeast // inferred from mutant phenotype///0001909 // leukocyte mediated cytotoxicity //
721.21643	1035.0679	NM_001285917///NN	0.005	1.46	***Dapk1***	death associated protein kinase 1	0006417 // regulation of translation // inferred from electronic annotation///0006468 // protein phosphorylation /
107.57254	163.78413	NM_001169153///NN	0.006	1.46	***Cd300lf***	CD300 antigen like family member F	0002376 // immune system process // inferred from electronic annotation///0030316 // osteoclast differentiation
1211.1276	1722.7161	NM_001042565///NN	0.002	1.46	***Wsb1***	WD repeat and SOCS box-containing 1	0016567 // protein ubiquitination // inferred from electronic annotation///0035556 // intracellular signal transduc

[Nrf2-KO, Air, PBS]	[Nrf2-KO, Air, SFN]	RefSeq Transcript ID	p	FC PBS:SFN	Gene Symbol	Gene Title	Gene Ontology
420.41507	606.72095	NM_010153	0.001	1.46	***Erbb3***	v-erb-b2 erythroblastic leukemia viral oncogene homolog 3 (avian)	0003197 // endocardial cushion development // inferred from mutant phenotype///0006468 // protein phosphory
65.2575	101.230415	NM_010172///XM_0(	0.001	1.45	***F7***	coagulation factor VII	0002690 // positive regulation of leukocyte chemotaxis // not recorded///0006508 // proteolysis // not recorded//
1635.5033	2296.8918	NM_001081066///XN	0.005	1.45	***Dennd3***	DENN/MADD domain containing 3	0008333 // endosome to lysosome transport // inferred from genetic interaction///0032313 // regulation of Rab G
637.93494	906.65656	NM_009663	0.003	1.45	***Alox5ap***	arachidonate 5-lipoxygenase activating protein	0002540 // leukotriene production involved in inflammatory response // inferred from mutant phenotype///00026
605.808	851.91	NM_001083319///NI	0.003	1.44	***Ubp1***	upstream binding protein 1	0001525 // angiogenesis // inferred from mutant phenotype///0006351 // transcription, DNA-templated // inferre
263.9472	379.15244	NM_008518	0.004	1.43	***Ltb***	lymphotoxin B	0006955 // immune response // inferred from electronic annotation///0010467 // gene expression // inferred fror
269.98145	384.48294	NM_001177646///NI	0.001	1.43	***Sirpa***	signal-regulatory protein alpha	0002244 // hematopoietic progenitor cell differentiation // inferred from mutant phenotype///0006910 // phagoc
895.12146	1246.6874	NM_013706	0.003	1.43	***Cd52***	CD52 antigen	0005886 // plasma membrane // inferred from electronic annotation///0016020 // membrane // inferred from ele
415.05365	588.68115	NM_001033261///XN	0.002	1.43	***Zfc3h1***	zinc finger, C3H1-type containing	0006396 // RNA processing // inferred from electronic annotation
76.22841	115.73562	NM_001289877///NI	0.002	1.43	***Per3***	period circadian clock 3	0000122 // negative regulation of transcription from RNA polymerase II promoter // inferred from direct assay///0
404.38428	571.02747	NM_001081170///NI	0.003	1.42	***Pacs2***	phosphofurin acidic cluster sorting protein 2	0006915 // apoptotic process // inferred from electronic annotation///0072661 // protein targeting to plasma mer
98.16076	146.41182	NM_133898///XM_0(	0.009	1.42	***N4bp2l1***	NEDD4 binding protein 2-like 1	0005524 // ATP binding // inferred from electronic annotation///0016301 // kinase activity // inferred from electro
10063.819	13977.084	NM_017372	0.001	1.42	***Lyz2***	lysozyme 2	0008152 // metabolic process // inferred from electronic annotation///0016998 // cell wall macromolecule catabo
760.2875	1051.3357	NM_001114174///XN	0.002	1.42	***Fam189a2***	family with sequence similarity 189, member A2	0016021 // integral component of membrane // inferred from electronic annotation
319.055	448.22928	NM_008580///XM_0(	0.001	1.41	***Map3k5***	mitogen-activated protein kinase kinase kinase 5	0000165 // MAPK cascade // inferred from direct assay///0000165 // MAPK cascade // not recorded///0000186 //
402.90393	563.9757	NM_001040400///NI	0.008	1.41	***Tet2***	tet methylcytosine dioxygenase 2	0001822 // kidney development // inferred from mutant phenotype///0006211 // 5-methylcytosine catabolic proc
272.56906	382.9671	NM_030075///XM_0(	0.007	1.41	***Klhdc8b***	kelch domain containing 8B	0005515 // protein binding // inferred from electronic annotation
562.46075	777.54803	NM_010686///XM_0(	0.005	1.41	***Laptm5***	lysosomal-associated protein transmembrane 5	0006810 // transport // inferred from electronic annotation
1160.15	1591.2454	NM_010091///XM_0(	0.006	1.40	***Dvl1***	dishevelled, dsh homolog 1 (Drosophila)	0001505 // regulation of neurotransmitter levels // inferred from mutant phenotype///0001932 // regulation of pr
1918.7164	2618.1584	NM_001162926///NI	0.002	1.40	***Fam84b***	family with sequence similarity 84, member B	0005515 // protein binding // inferred from electronic annotation
1219.1124	1655.5789	NM_019521	0.008	1.40	***Gas6***	growth arrest specific 6	0001764 // neuron migration // inferred from genetic interaction///0001934 // positive regulation of protein phos
146.06009	208.26578	NM_028608///XM_0(	0.009	1.40	***Glipr1***	GLI pathogenesis-related 1 (glioma)	0005576 // extracellular region // inferred from electronic annotation///0016020 // membrane // inferred from ele
343.43198	474.72345	NM_172154///XM_0(	0.001	1.40	***Lcor***	ligand dependent nuclear receptor corepressor	0000122 // negative regulation of transcription from RNA polymerase II promoter // inferred from direct assay///0
408.04907	564.2121	NM_175518///XM_0(	0.003	1.40	***Tmem245***	transmembrane protein 245	0016020 // membrane // inferred from electronic annotation///0016021 // integral component of membrane // in
243.98155	339.63016	NM_001113333///NI	0.003	1.39	***Cry2***	cryptochrome 2 (photolyase-like)	0000122 // negative regulation of transcription from RNA polymerase II promoter // not recorded///0006281 // DI
329.148	455.91876	NM_172771///XM_0(	0.009	1.39	***Dmxl2***	Dmx-like 2	0005515 // protein binding // inferred from electronic annotation///0017137 // Rab GTPase binding // not recorde
240.03908	334.49823	NM_001037937///NI	0.007	1.39	***Deptor***	DEP domain containing MTOR-interacting protein	0006469 // negative regulation of protein kinase activity // not recorded///0032007 // negative regulation of TOR
98.49426	144.27295	NM_001024474///XN	0.008	1.39	***Diras2***	DIRAS family, GTP-binding RAS-like 2	0006184 // GTP catabolic process // inferred from electronic annotation///0007165 // signal transduction // inferr
37.372078	54.18356	NM_007696	0.003	1.39	***Ovgp1***	oviductal glycoprotein 1	0005975 // carbohydrate metabolic process // inferred from electronic annotation///0006032 // chitin catabolic pr
30.447065	43.374638	NM_001033170///XN	0.004	1.39	***Fam83e***	family with sequence similarity 83, member E	
165.02611	232.23625	NM_001109040///NI	0.010	1.39	***Kif21a***	kinesin family member 21A	0007018 // microtubule-based movement // inferred from electronic annotation///0008152 // metabolic process /
210.46295	294.49002	NR_029468	0.001	1.39	***Snhg12***	small nucleolar RNA host gene 12	
158.4037	224.60265	NM_018828///XM_0(	0.000	1.39	***Fnbp4***	formin binding protein 4	0005515 // protein binding // inferred from physical interaction
204.90239	286.09262	NM_008359///XM_0(	0.001	1.38	***Il17ra***	interleukin 17 receptor A	0032747 // positive regulation of interleukin-23 production // not recorded///0071345 // cellular response to cyto
244.3725	337.89264	NM_001033348///XN	0.005	1.38	***Ralgapa2***	Ral GTPase activating protein, alpha subunit 2 (catalytic)	0032484 // Ral protein signal transduction // inferred from mutant phenotype///0032859 // activation of Ral GTPa
14.291265	19.655941	NM_001159424///NI	0.004	1.38	***Il12a***	interleukin 12a	0001916 // positive regulation of T cell mediated cytotoxicity // not recorded///0002860 // positive regulation of n
497.98553	681.2343	NM_001290573///NI	0.002	1.38	***Kmt2b***	lysine (K)-specific methyltransferase 2B	0001541 // ovarian follicle development // inferred from mutant phenotype///0006351 // transcription, DNA-temp
1064.6938	1431.9719	NM_026313///XM_0(	0.002	1.38	***Luc7l3///AI503301***	LUC7-like 3 (S. cerevisiae)///expressed sequence AI503301	0006376 // mRNA splice site selection // inferred from electronic annotation///0006397 // mRNA processing // inf
695.4854	933.27936	NR_002845	0.008	1.38	***Copg2os2***	coatomer protein complex, subunit gamma 2, opposite strand 2	
118.432434	169.44498	NM_001271962///NI	0.004	1.38	***Eya2***	eyes absent 2 homolog (Drosophila)	0006281 // DNA repair // inferred from electronic annotation///0006351 // transcription, DNA-templated // inferr
274.48474	375.48	NM_080575	0.003	1.37	***Acss1***	acyl-CoA synthetase short-chain family member 1	0006085 // acetyl-CoA biosynthetic process // inferred from direct assay///0006085 // acetyl-CoA biosynthetic pro
3024.07	4055.1255	NM_030238	0.001	1.37	***Dync1h1***	dynein cytoplasmic 1 heavy chain 1	0003341 // cilium movement // inferred by curator///0006200 // ATP catabolic process // inferred from electronic
360.07608	488.89444	NM_147219///XM_0(	0.007	1.37	***Abca5***	ATP-binding cassette, sub-family A (ABC1), member 5	0006200 // ATP catabolic process // inferred from electronic annotation///0006810 // transport // inferred from el
20.263027	27.717836	NM_010400	0.004	1.37	***H60a***	histocompatibility 60a	0001913 // T cell mediated cytotoxicity // ---///0002839 // positive regulation of immune response to tumor cell //
14.996394	20.318047	NM_001145977///NI	0.009	1.37	***Cadm2***	cell adhesion molecule 2	0007155 // cell adhesion // inferred from electronic annotation///0007420 // brain development // inferred from
1704.8632	2272.303	NM_001164564///NI	0.004	1.37	***Egfl7***	EGF-like domain 7	0001525 // angiogenesis // inferred from electronic annotation///0001568 // blood vessel development // inferrec
474.69678	642.2886	NM_016756///NM_1	0.004	1.37	***Cdk2***	cyclin-dependent kinase 2	0000082 // G1/S transition of mitotic cell cycle // inferred from direct assay///0006281 // DNA repair // inferred fr
12.984719	17.603836	NM_001004182///XN	0.001	1.36	***A930009E05Rik///Tmem178b***	RIKEN cDNA A930009E05 gene///transmembrane protein 178B	0016021 // integral component of membrane // inferred from electronic annotation
218.60852	299.85828	NM_001029856///XN	0.002	1.36	***Atad5***	ATPase family, AAA domain containing 5	0006974 // cellular response to DNA damage stimulus // inferred from electronic annotation///0008152 // metabo
35.079273	49.494316	NM_133203///XM_0(	0.005	1.36	***Klra17***	killer cell lectin-like receptor, subfamily A, member 17	0030246 // carbohydrate binding // inferred from electronic annotation
1136.2365	1499.445	NM_001012330///NI	0.002	1.36	***Zbtb18***	zinc finger and BTB domain containing 18	0000122 // negative regulation of transcription from RNA polymerase II promoter // inferred from direct assay///0
1016.2122	1338.975	NM_001163215///NI	0.003	1.36	***Fgfr3***	fibroblast growth factor receptor 3	0000122 // negative regulation of transcription from RNA polymerase II promoter // inferred from mutant phenoty
189.85846	261.89014	NM_178440///XM_0(	0.002	1.36	***Myo1g***	myosin IG	0008152 // metabolic process // inferred from electronic annotation
51.388817	73.38285	NM_013825	0.006	1.36	***Ly75***	lymphocyte antigen 75	0006897 // endocytosis // inferred from electronic annotation
77.229774	111.39694	NM_001162415///NI	0.007	1.36	***Pfkfb2***	6-phosphofructo-2-kinase/fructose-2,6-biphosphatase 2	0006000 // fructose metabolic process // inferred from electronic annotation///0006003 // fructose 2,6-bisphosph
246.10506	333.69586	NM_153122///XM_0(	0.002	1.36	***Oplah***	5-oxoprolinase (ATP-hydrolysing)	0006749 // glutathione metabolic process // inferred from sequence or structural similarity
288.03516	388.91666	NM_144913///XM_0(	0.001	1.36	***Mepce***	methylphosphate capping enzyme	0000122 // negative regulation of transcription from RNA polymerase II promoter // inferred from genetic interact
363.11362	488.96133	NM_013830///XM_0(	0.001	1.36	***Prpf4b***	PRP4 pre-mRNA processing factor 4 homolog B (yeast)	0006397 // mRNA processing // inferred from electronic annotation///0006468 // protein phosphorylation // infer
275.38635	372.4274	NM_009151///XM_0(	0.004	1.36	***Selplg***	selectin, platelet (p-selectin) ligand	0007155 // cell adhesion // inferred from electronic annotation///0050901 // leukocyte tethering or rolling // infer
198.40002	273.2777	NM_030168///XM_0(	0.003	1.36	***Rictor***	RPTOR independent companion of MTOR, complex 2	0001938 // positive regulation of endothelial cell proliferation // not recorded///0007275 // multicellular organism
210.31165	288.02737	NM_007692///NM_0	0.009	1.36	***Chkb///ChkbCpt1b///Cpt1b***	choline kinase beta///Chkb-Cpt1b readthrough transcript (NMD candidate)///carnit	0006629 // lipid metabolic process // inferred from electronic annotation///0006631 // fatty acid metabolic proces
5232.082	6952.707	NM_027878///XM_0(	0.003	1.35	***Dram1***	DNA-damage regulated autophagy modulator 1	0006914 // autophagy // inferred from electronic annotation///0006915 // apoptotic process // inferred from elec
846.0644	1117.9769	NM_001290676///NI	0.001	1.35	***Cpeb4***	cytoplasmic polyadenylation element binding protein 4	0002931 // response to ischemia // inferred from direct assay///0035235 // ionotropic glutamate receptor signalin
18.109804	24.377796	NM_011033	0.002	1.35	***Pabpc2***	poly(A) binding protein, cytoplasmic 2	0006378 // mRNA polyadenylation // traceable author statement
662.10724	877.84503	NM_026573///XM_0(	0.004	1.35	***Upf3b***	UPF3 regulator of nonsense transcripts homolog B (yeast)	0000184 // nuclear-transcribed mRNA catabolic process, nonsense-mediated decay // not recorded///0006986 // r
178.81508	245.7998	NM_001099319///NI	0.001	1.35	***Gm12942///Zmym6***	predicted gene 12942///zinc finger, MYM-type 6	0007010 // cytoskeleton organization // not recorded///0022604 // regulation of cell morphogenesis // not record
832.45593	1097.4224	NM_001038999///NI	0.002	1.35	***Atp8a1***	ATPase, aminophospholipid transporter (APLT), class I, type 8A, member 1	0006810 // transport // inferred from electronic annotation///0006812 // cation transport // inferred from electro
584.45013	775.4994	NM_011240	0.001	1.35	***Ranbp2***	RAN binding protein 2	0000413 // protein peptidyl-prolyl isomerization // inferred from electronic annotation///0006457 // protein foldir
271.43558	365.693	NM_146013///XM_0(	0.003	1.35	***Sec14l4***	SEC14-like 4 (S. cerevisiae)	0006810 // transport // inferred from electronic annotation
216.18271	293.67044	NR_040628///NM_13	0.001	1.35	***Gm17066///Rbm26***	predicted gene 17066///RNA binding motif protein 26	0006397 // mRNA processing // inferred from electronic annotation///0010923 // negative regulation of phosphat
1014.3445	1326.374	NM_010207///NM_2	0.007	1.35	***Fgfr2***	fibroblast growth factor receptor 2	0000122 // negative regulation of transcription from RNA polymerase II promoter // inferred from mutant phenoty

[Nrf2-KO, Air, PBS]	[Nrf2-KO, Air, SFN]	RefSeq Transcript ID	p	FC PBS:SFN	Gene Symbol	Gene Title	Gene Ontology
472.12253	631.63904	NM_001288625///NI	0.005	1.34	***Arid3a***	AT rich interactive domain 3A (BRIGHT-like)	0006351 // transcription, DNA-templated // inferred from electronic annotation///0006355 // regulation of transcı
917.3113	1203.6443	NM_011436	0.001	1.34	***Sorl1***	sortilin-related receptor, LDLR class A repeats-containing	0000042 // protein targeting to Golgi // not recorded///0006605 // protein targeting // not recorded///0006622 //
488.9552	653.52966	NM_001122675///NI	0.001	1.34	***Zcchc2***	zinc finger, CCHC domain containing 2	0007154 // cell communication // inferred from electronic annotation
1314.8274	1714.4128	NM_010664///XM_0	0.009	1.34	***Krt18***	keratin 18	0033209 // tumor necrosis factor-mediated signaling pathway // inferred from genetic interaction///0043000 // Gc
433.61078	571.9356	NM_025845///XM_0	0.004	1.33	***Prpf38b***	PRP38 pre-mRNA processing factor 38 (yeast) domain containing B	0006397 // mRNA processing // inferred from electronic annotation///0008380 // RNA splicing // inferred from ele
298.26135	394.98712	NM_011814	0.001	1.33	***Fxr2***	fragile X mental retardation, autosomal homolog 2	0003723 // RNA binding // inferred from electronic annotation///0005515 // protein binding // inferred from phys
2606.6987	3370.1484	NM_001199136///NI	0.001	1.33	***Macf1***	microtubule-actin crosslinking factor 1	0001707 // mesoderm formation // inferred from mutant phenotype///0006200 // ATP catabolic process // inferre
10575.217	13705.487	NM_011402///XM_0	0.003	1.33	***Slc34a2***	solute carrier family 34 (sodium phosphate), member 2	0001701 // in utero embryonic development // inferred from mutant phenotype///0006810 // transport // inferre
35.97612	49.56613	NM_153529///XM_0	0.008	1.33	***Nrn1***	neuritin 1	0007399 // nervous system development // not recorded///0007409 // axonogenesis // not recorded
396.4879	520.0435		0.004	1.33	***AK077428***	Mus musculus 8 days embryo whole body cDNA, RIKEN full-length enriched library, clone:5730407P16 product:unclassifiable, full insert sequence.	
233.65855	311.17303	NM_001271587///NI	0.002	1.33	***Eps8***	epidermal growth factor receptor pathway substrate 8	0008344 // adult locomotory behavior // inferred from mutant phenotype///0008360 // regulation of cell shape //
301.84064	398.9988	NM_001165997///NI	0.002	1.33	***Spopl***	speckle-type POZ protein-like	0016567 // protein ubiquitination // inferred from electronic annotation///0031397 // negative regulation of prote
609.439	790.1372	NM_001289690///NI	0.004	1.33	***Zfp740***	zinc finger protein 740	0006351 // transcription, DNA-templated // inferred from electronic annotation///0006355 // regulation of transcı
180.18382	242.82791	NM_009343///XM_0	0.003	1.33	***Phf1***	PHD finger protein 1	0006351 // transcription, DNA-templated // inferred from electronic annotation///0006355 // regulation of transcı
609.3109	791.4686	NM_134115///XM_0	0.005	1.33	***Stk38***	serine/threonine kinase 38	0006464 // cellular protein modification process // not recorded///0006468 // protein phosphorylation // not reco
424.4299	556.1001	NM_011207///XM_0	0.007	1.32	***Ptpn3***	protein tyrosine phosphatase, non-receptor type 3	0006470 // protein dephosphorylation // not recorded///0016310 // phosphorylation // inferred from electronic a
197.18408	264.76764	NM_030014///XM_0	0.003	1.32	***Hook1***	hook homolog 1 (Drosophila)	0006810 // transport // inferred from electronic annotation///0007032 // endosome organization // not recorded/
376.61676	493.999	NM_001031814///NI	0.002	1.32	***Smg1***	SMG1 homolog, phosphatidylinositol 3-kinase-related kinase (C. elegans)	0000184 // nuclear-transcribed mRNA catabolic process, nonsense-mediated decay // not recorded///0006281 // I
688.90015	888.1753	NM_176836///XM_0	0.006	1.32	***Fam76b***	family with sequence similarity 76, member B	0005634 // nucleus // not recorded///0016607 // nuclear speck // inferred from electronic annotation///0031965
838.38586	1082.0266	NM_008551	0.002	1.32	***Mapkapk2***	MAP kinase-activated protein kinase 2	0002224 // toll-like receptor signaling pathway // inferred from mutant phenotype///0006468 // protein phosphor
332.17795	435.83673	NM_144916	0.007	1.32	***Tmem150a***	transmembrane protein 150A	0009056 // catabolic process // inferred from electronic annotation
200.45071	267.08893	NM_025806///XM_0	0.010	1.32	***Plbd1***	phospholipase B domain containing 1	0006629 // lipid metabolic process // inferred from electronic annotation///0016042 // lipid catabolic process // in
585.27673	757.1203	NR_045008	0.005	1.32	***Gm20300***	predicted gene, 20300	
418.44238	546.31775	NM_019776///XM_0	0.003	1.32	***Snd1***	staphylococcal nuclease and tudor domain containing 1	0001649 // osteoblast differentiation // not recorded///0006351 // transcription, DNA-templated // inferred from
33.39544	45.240223	NM_170671///XM_0	0.001	1.32	***Mycbpap***	MYCBP associated protein	0006897 // endocytosis // inferred from electronic annotation///0007268 // synaptic transmission // inferred from
28.863644	38.33517	NM_001293795///NI	0.001	1.32	***Tm6sf2***	transmembrane 6 superfamily member 2	0016020 // membrane // inferred from electronic annotation///0016021 // integral component of membrane // in
261.2709	342.62677	NM_001163766///NI	0.003	1.31	***Wdr90***	WD repeat domain 90	0005515 // protein binding // inferred from electronic annotation
275.40002	361.2524	NM_001271358///NI	0.004	1.31	***Arrb2***	arrestin, beta 2	0001932 // regulation of protein phosphorylation // inferred from direct assay///0001932 // regulation of protein
800.39984	1029.9666	NM_009427	0.004	1.31	***Tob1***	transducer of ErbB-2.1	0007184 // SMAD protein import into nucleus // inferred from direct assay///0008285 // negative regulation of cel
1727.8665	2195.1538	NM_176849///XM_0	0.002	1.31	***Arglu1***	arginine and glutamate rich 1	0005515 // protein binding // inferred from electronic annotation
386.68106	502.51056	NM_001013414///NI	0.009	1.31	***Neurl4***	neuralized homolog 4 (Drosophila)	0005737 // cytoplasm // inferred from electronic annotation///0005814 // centriole // inferred from electronic anı
1565.5444	2000.6212	NM_001293667///NI	0.003	1.31	***Atp11a***	ATPase, class VI, type 11A	0006810 // transport // inferred from electronic annotation///0006812 // cation transport // inferred from electro
169.2596	226.04684	NM_001162945///XI	0.004	1.31	***Mtx3***	metaxin 3	0006626 // protein targeting to mitochondrion // inferred from electronic annotation
39.551662	54.26534		0.005	1.31	***Dnmbp***	Mus musculus dynamin binding protein (Dnmbp), mRNA.	
383.36273	497.33185	NM_001081151	0.005	1.31	***Gan***	giant axonal neuropathy	0007010 // cytoskeleton organization // not recorded///0008219 // cell death // inferred from electronic annotatio
23.972837	31.475975	NM_001042767///NI	0.006	1.31	***Proc***	protein C	0006508 // proteolysis // inferred from direct assay///0007596 // blood coagulation // inferred from electronic anı
2014.5023	2557.106	NM_013605	0.008	1.31	***Muc1***	mucin 1, transmembrane	0001666 // response to hypoxia // inferred from electronic annotation///0007565 // female pregnancy // inferred
394.33856	510.55887	NM_001025307///NI	0.005	1.31	***Stx3***	syntaxin 3	0006810 // transport // inferred from electronic annotation///0006836 // neurotransmitter transport // inferred fr
439.56625	569.4507	NM_018824///XM_0	0.005	1.30	***Slc23a2***	solute carrier family 23 (nucleobase transporters), member 2	0006810 // transport // inferred from electronic annotation///0006811 // ion transport // inferred from electronic
437.76752	566.42926	NM_153177///XM_0	0.007	1.30	***Ago4***	argonaute RISC catalytic subunit 4	0006402 // mRNA catabolic process // not recorded///0006417 // regulation of translation // inferred from electro
3274.0938	4193.465	NM_009864	0.006	1.30	***Cdh1***	cadherin 1	0001701 // in utero embryonic development // inferred from mutant phenotype///0001829 // trophectodermal ce
12.587478	16.356964	NM_007769///XM_0	0.007	1.30	***Dmbt1***	deleted in malignant brain tumors 1	0001824 // blastocyst development // inferred from mutant phenotype///0001833 // inner cell mass cell proliferat
459.502	594.1198	NM_001197046///NI	0.006	1.30	***Fgfr1op***	Fgfr1 oncogene partner	0006469 // negative regulation of protein kinase activity // not recorded///0008284 // positive regulation of cell pı
661.6627	842.575	NM_001025250///NI	0.004	1.30	***Vegfa***	vascular endothelial growth factor A	0000122 // negative regulation of transcription from RNA polymerase II promoter // not recorded///0001525 // an
728.02295	924.3662	NM_172645///XM_0	0.002	1.30	***Suco***	SUN domain containing ossification factor	0001503 // ossification // inferred from electronic annotation///0007275 // multicellular organismal development
265.25412	344.7731	NM_009201///XM_0	0.003	1.30	***Slc1a5***	solute carrier family 1 (neutral amino acid transporter), member 5	0003333 // amino acid transmembrane transport // inferred from direct assay///0003333 // amino acid transmem
346.88977	448.51886	NM_133921///XM_0	0.008	1.30	***Nfxl1***	nuclear transcription factor, X-box binding-like 1	0006355 // regulation of transcription, DNA-templated // inferred from electronic annotation
37.38658	50.13584	NM_013486///XM_0	0.006	1.30	***Cd2***	CD2 antigen	0007155 // cell adhesion // inferred from electronic annotation///0016337 // single organismal cell-cell adhesion /
1231.9764	1553.1554	NM_008986	0.008	1.30	***Ptrf***	polymerase I and transcript release factor	0006351 // transcription, DNA-templated // inferred from electronic annotation///0006353 // DNA-templated trar
516.6606	665.30255	NM_153056///XM_0	0.005	1.30	***Sirt7***	sirtuin 7	0000122 // negative regulation of transcription from RNA polymerase II promoter // not recorded///0001701 // in
2233.261	2811.2349	NM_009170///XM_0	0.002	1.30	***Shh***	sonic hedgehog	0000122 // negative regulation of transcription from RNA polymerase II promoter // inferred from direct assay///0
278.69446	360.3511	NM_145151///NR_07	0.004	1.30	***Crebzf***	CREB/ATF bZIP transcription factor	0006351 // transcription, DNA-templated // not recorded///0006355 // regulation of transcription, DNA-templatec
139.60599	186.0955	NM_177372///XR_38	0.005	1.29	***Dna2***	DNA replication helicase 2 homolog (yeast)	0000076 // DNA replication checkpoint // not recorded///0000729 // DNA double-strand break processing // not r
3971.3213	5054.2417	NM_139149///XM_0	0.003	1.29	***Fus***	fused in sarcoma	0000166 // nucleotide binding // inferred from electronic annotation///0003676 // nucleic acid binding // inferred
695.9025	879.6022	NM_019774///XM_0	0.003	1.29	***Akap8***	A kinase (PRKA) anchor protein 8	0007076 // mitotic chromosome condensation // inferred from direct assay///0007076 // mitotic chromosome cor
233.93326	304.2318	NM_177806///XM_0	0.008	1.29	***Prpf39***	PRP39 pre-mRNA processing factor 39 homolog (yeast)	0006396 // RNA processing // inferred from electronic annotation///0006397 // mRNA processing // inferred from
470.99567	603.21295	NM_001005507///NI	0.004	1.29	***Smg7***	Smg-7 homolog, nonsense mediated mRNA decay factor (C. elegans)	0000184 // nuclear-transcribed mRNA catabolic process, nonsense-mediated decay // inferred from electronic anı
730.8331	919.7546	NM_001160319	0.002	1.29	***Ubr4***	ubiquitin protein ligase E3 component n-recognin 4	0016567 // protein ubiquitination // inferred from electronic annotation///0042787 // protein ubiquitination invol
191.96724	251.37547	NM_146222///XM_0	0.004	1.29	***Msantd2***	Myb/SANT-like DNA-binding domain containing 2	
648.00916	814.9779	NM_001115130///NI	0.001	1.29	***Zbtb44***	zinc finger and BTB domain containing 44	0006351 // transcription, DNA-templated // inferred from electronic annotation///0006355 // regulation of transcı
1493.5106	1867.6942	NM_001039581///NI	0.003	1.28	***Abca3***	ATP-binding cassette, sub-family A (ABC1), member 3	0006200 // ATP catabolic process // inferred from electronic annotation///0006810 // transport // not recorded//
1055.8202	1326.7117	NM_001159963///NI	0.002	1.28	***Fbxl5***	F-box and leucine-rich repeat protein 5	0016567 // protein ubiquitination // not recorded///0031146 // SCF-dependent proteasomal ubiquitin-dependent
146.54016	193.85947	NM_207202	0.003	1.28	***Ccdc120***	coiled-coil domain containing 120	0007165 // signal transduction // inferred from electronic annotation
2566.4402	3209.8403	NM_026499	0.004	1.28	***Srsf6***	serine/arginine-rich splicing factor 6	0000380 // alternative mRNA splicing, via spliceosome // not recorded///0000381 // regulation of alternative mRN
74.15291	101.289955	NR_028380///NR_02	0.008	1.28	***Ftx***	Ftx transcript, Xist regulator (non-protein coding)	0006325 // chromatin organization // inferred from mutant phenotype///0010468 // regulation of gene expressior
1120.0303	1391.6128	NM_008532	0.003	1.28	***Epcam***	epithelial cell adhesion molecule	0001657 // ureteric bud development // inferred from expression pattern///0008284 // positive regulation of cell
903.1811	1124.0533	NM_173378	0.005	1.27	***Trp53bp2***	transformation related protein 53 binding protein 2	0006915 // apoptotic process // inferred from electronic annotation///0007049 // cell cycle // inferred from electr
534.7251	676.7039	NM_007651	0.006	1.27	***Cd53***	CD53 antigen	0016337 // single organismal cell-cell adhesion // inferred from direct assay///0045661 // regulation of myoblast d
167.33719	217.21092	NM_001168290///NI	0.009	1.27	***Sugp2***	SURP and G patch domain containing 2	0006396 // RNA processing // inferred from electronic annotation///0006397 // mRNA processing // inferred from
219.17746	281.06815	NM_178601///XR_10	0.009	1.27	***D1Ertd448e///Imp4***	DNA segment, Chr 1, ERATO Doi 448, expressed///IMP4, U3 small nucleolar ribonuc	0006364 // rRNA processing // traceable author statement///0042254 // ribosome biogenesis // inferred from elec
1251.5516	1546.7518	NM_001034851///NI	0.005	1.27	***Fam134b***	family with sequence similarity 134, member B	0019233 // sensory perception of pain // not recorded

[Nrf2-KO, Air, PBS]	[Nrf2-KO, Air, SFN]	RefSeq Transcript ID	p	FC PBS:SFN	Gene Symbol	Gene Title	Gene Ontology
411.46655	516.79956	NM_001205353///NI	0.003	1.27	***Gramd4***	GRAM domain containing 4	0006915 // apoptotic process // inferred from electronic annotation
608.4214	758.4442	NM_007462///XM_0	0.009	1.27	***Apc***	adenomatosis polyposis coli	0000281 // mitotic cytokinesis // inferred from mutant phenotype///0000281 // mitotic cytokinesis // not recorde
565.25287	706.62305	NM_027349///XM_0	0.005	1.27	***Rbm25***	RNA binding motif protein 25	0000381 // regulation of alternative mRNA splicing, via spliceosome // inferred from electronic annotation///0000
399.18823	500.93253	NM_153790///XM_0	0.006	1.27	***Scarf2***	scavenger receptor class F, member 2	0006898 // receptor-mediated endocytosis // inferred from direct assay///0007155 // cell adhesion // inferred fro
2450.2734	3027.0522	NM_001145824///NI	0.005	1.27	***Hipk3***	homeodomain interacting protein kinase 3	0000122 // negative regulation of transcription from RNA polymerase II promoter // inferred from sequence or stru
1519.6454	1867.6646	NM_008149///XM_0	0.005	1.27	***Gpam***	glycerol-3-phosphate acyltransferase, mitochondrial	0006629 // lipid metabolic process // inferred from electronic annotation///0006631 // fatty acid metabolic proces
518.90076	652.2919	NM_001081171///XI	0.006	1.27	***Lama5***	laminin, alpha 5	0001658 // branching involved in ureteric bud morphogenesis // inferred from mutant phenotype///0001738 // m
746.5401	925.05383	NM_001081039///NI	0.005	1.26	***Dock9***	dedicator of cytokinesis 9	0007264 // small GTPase mediated signal transduction // inferred from electronic annotation///0032319 // regulat
24.511972	31.13356	NM_001281955///XI	0.005	1.26	***Csmd2***	CUB and Sushi multiple domains 2	
535.93176	671.21704	NM_001081150	0.006	1.26	***Lonrf1***	LON peptidase N-terminal domain and ring finger 1	0006200 // ATP catabolic process // inferred from electronic annotation///0006508 // proteolysis // inferred from
704.786	870.77216	NM_144925///XM_0	0.010	1.26	***Tnrc6a***	trinucleotide repeat containing 6a	0006417 // regulation of translation // inferred from electronic annotation///0009267 // cellular response to starv
412.89673	516.568	NM_001045523///XI	0.010	1.26	***Bahd1***	bromo adjacent homology domain containing 1	0006351 // transcription, DNA-templated // inferred from electronic annotation///0006355 // regulation of transcr
4610.6074	5672.172	NM_001081286///XI	0.003	1.26	***Fat1***	FAT tumor suppressor homolog 1 (Drosophila)	0007015 // actin filament organization // inferred from mutant phenotype///0007155 // cell adhesion // inferred f
1721.9828	2111.8345	NM_001025387///NI	0.010	1.26	***Brd2***	bromodomain containing 2	0006334 // nucleosome assembly // not recorded///0006351 // transcription, DNA-templated // inferred from ele
204.48326	260.37762	NM_001136088///NI	0.008	1.26	***Sh3bp2***	SH3-domain binding protein 2	0005515 // protein binding // inferred from physical interaction///0005543 // phospholipid binding // inferred fro
395.9646	493.76358	NM_001045529///XI	0.008	1.26	***Morc3***	microrchidia 3	0006468 // protein phosphorylation // not recorded///0007569 // cell aging // not recorded///0009791 // post-em
580.33966	720.61646	NM_018759///NR_03	0.002	1.26	***Zfp326***	zinc finger protein 326	0006351 // transcription, DNA-templated // inferred from electronic annotation///0006355 // regulation of transcr
418.92673	522.90533	NM_133829///NM_1	0.009	1.26	***Mfsd6***	major facilitator superfamily domain containing 6	0002376 // immune system process // inferred from electronic annotation///0055085 // transmembrane transport
587.98303	728.3994	NM_001145813///NI	0.009	1.26	***Elf5***	E74-like factor 5	0006351 // transcription, DNA-templated // inferred from electronic annotation///0006355 // regulation of transcr
138.86198	181.14723	NM_133770///XM_0	0.003	1.26	***Adck4***	aarF domain containing kinase 4	0006468 // protein phosphorylation // inferred from electronic annotation///0008152 // metabolic process // infe
280.98007	352.43027	NM_001164184///NI	0.004	1.26	***Lsr***	lipolysis stimulated lipoprotein receptor	0001889 // liver development // inferred from mutant phenotype///0006898 // receptor-mediated endocytosis //
487.2165	607.7341	NM_017463	0.004	1.26	***Pbx2***	pre B cell leukemia homeobox 2	0006351 // transcription, DNA-templated // inferred from electronic annotation///0006355 // regulation of transcr
667.5678	820.72095	NM_001285870///NI	0.008	1.26	***Trim3***	tripartite motif-containing 3	0006810 // transport // inferred from electronic annotation///0015031 // protein transport // inferred from electr
1311.7941	1596.239	NM_001287180///NI	0.006	1.26	***Atf4***	activating transcription factor 4	0006094 // gluconeogenesis // inferred from direct assay///0006351 // transcription, DNA-templated // inferred fr
329.62616	411.33902	NM_080553///XM_0	0.004	1.25	***Itpr3***	inositol 1,4,5-triphosphate receptor 3	0006810 // transport // inferred from electronic annotation///0006811 // ion transport // inferred from electronic
382.41815	478.77054	NM_001166648///NI	0.008	1.25	***Zfp280c***	zinc finger protein 280C	0006351 // transcription, DNA-templated // inferred from electronic annotation///0006355 // regulation of transcr
1088.8774	1335.6722	NM_001271599///NI	0.009	1.25	***Sort1***	sortilin 1	0001503 // ossification // inferred from electronic annotation///0006810 // transport // inferred from electronic a
500.95636	622.4535	NM_001146176///NI	0.010	1.25	***Max***	Max protein	0006351 // transcription, DNA-templated // inferred from electronic annotation///0006355 // regulation of transcr
233.95839	295.29355	NM_026888///XM_0	0.005	1.25	***Phkg2***	phosphorylase kinase, gamma 2 (testis)	0005975 // carbohydrate metabolic process // inferred from electronic annotation///0005977 // glycogen metabol
356.93933	445.84897	NM_001081049///XI	0.003	1.25	***Kmt2a***	lysine (K)-specific methyltransferase 2A	0006306 // DNA methylation // inferred from mutant phenotype///0006351 // transcription, DNA-templated // inf
731.56586	895.31793	NM_010690///XM_0	0.009	1.25	***Lats1***	large tumor suppressor	0000086 // G2/M transition of mitotic cell cycle // not recorded///0000819 // sister chromatid segregation // not r
380.66827	473.32748	NM_172652///XM_0	0.004	1.25	***Kansl3***	KAT8 regulatory NSL complex subunit 3	0016568 // chromatin modification // inferred from electronic annotation///0043981 // histone H4-K5 acetylation
626.3747	766.0071	NM_144837///XM_0	0.008	1.25	***Ice1***	interactor of little elongation complex ELL subunit 1	
727.53564	891.14105	NM_172866///XM_0	0.003	1.25	***Rgp1***	RGP1 retrograde golgi transport homolog (S. cerevisiae)	
1297.6232	1574.0868	NM_177301///XM_0	0.006	1.25	***Hnrnpl***	heterogeneous nuclear ribonucleoprotein L	0006397 // mRNA processing // inferred from electronic annotation
1788.1697	2166.5437	NM_011519///XM_0	0.004	1.25	***Sdc1***	syndecan 1	0001657 // ureteric bud development // inferred from mutant phenotype///0006954 // inflammatory response
205.466	259.38693	NM_009886///XM_0	0.008	1.25	***Celsr1***	cadherin, EGF LAG seven-pass G-type receptor 1 (flamingo homolog, Drosophila)	0001702 // gastrulation with mouth forming second // non-traceable author statement///0001736 // establishmer
321.0384	396.3768	NM_009461///XM_0	0.004	1.24	***Ubr1***	ubiquitin protein ligase E3 component n-recognin 1	0006511 // ubiquitin-dependent protein catabolic process // inferred from direct assay///0006511 // ubiquitin-dep
1534.4547	1861.2865	NM_178920	0.006	1.24	***Mal2***	mal, T cell differentiation protein 2	0005515 // protein binding // inferred from electronic annotation
254.1309	316.2108	NM_023229///XM_0	0.010	1.24	***Fastk***	Fas-activated serine/threonine kinase	0006468 // protein phosphorylation // not recorded///0006915 // apoptotic process // inferred from electronic an
264.01752	328.5765	NM_001253752///NI	0.004	1.24	***Zmym5***	zinc finger, MYM-type 5	0008270 // zinc ion binding // inferred from electronic annotation///0046872 // metal ion binding // inferred from
810.8788	977.0885	NM_010585///XM_0	0.006	1.24	***Itpr1***	inositol 1,4,5-trisphosphate receptor 1	0001666 // response to hypoxia // inferred from direct assay///0001666 // response to hypoxia // not recorded///
506.74896	623.7485	NM_029868///XM_0	0.008	1.24	***Gpbp1l1***	GC-rich promoter binding protein 1-like 1	0006351 // transcription, DNA-templated // inferred from electronic annotation///0006355 // regulation of transcr
399.1241	491.10736	NM_001290783///NI	0.006	1.24	***Wdr13***	WD repeat domain 13	0005515 // protein binding // inferred from electronic annotation
296.8177	365.09445	NM_001289429///NI	0.007	1.23	***Cipc***	CLOCK interacting protein, circadian	0006351 // transcription, DNA-templated // inferred from electronic annotation///0006355 // regulation of transcr
345.6085	423.71396	NM_201362///XM_0	0.008	1.23	***Ccdc68***	coiled-coil domain containing 68	
3586.9675	4410.366	NM_007569	0.008	1.23	***Btg1***	B cell translocation gene 1, anti-proliferative	0006479 // protein methylation // inferred from direct assay///0006979 // response to oxidative stress // inferred
91.4678	119.54797	NM_001163749///NI	0.006	1.23	***Camsap3***	calmodulin regulated spectrin-associated protein family, member 3	0000226 // microtubule cytoskeleton organization // not recorded///0010923 // negative regulation of phosphatas
1254.9084	1502.8506	NM_001164503///XI	0.010	1.23	***Akap11***	A kinase (PRKA) anchor protein 11	0010738 // regulation of protein kinase A signaling // not recorded///0042325 // regulation of phosphorylation //
411.1055	502.8651	NM_133757///XM_0	0.009	1.23	***Pgs1***	phosphatidylglycerophosphate synthase 1	0006629 // lipid metabolic process // inferred from electronic annotation///0006655 // phosphatidylglycerol biosy
541.69824	660.2406	NM_001173547///NI	0.008	1.23	***Taz***	tafazzin	0006936 // muscle contraction // not recorded///0007507 // heart development // not recorded///0007519 // ske
317.12598	388.85678	NM_198170///XM_0	0.009	1.23	***Szt2***	seizure threshold 2	0007417 // central nervous system development // inferred from mutant phenotype///0009790 // embryo develo
1376.9081	1637.7526	NM_021604///XM_0	0.010	1.23	***Agrn***	agrin	0001932 // regulation of protein phosphorylation // not recorded///0001934 // positive regulation of protein phos
213.4828	264.86884	NM_028047///XM_0	0.010	1.23	***Smg9***	smg-9 homolog, nonsense mediated mRNA decay factor (C. elegans)	0000184 // nuclear-transcribed mRNA catabolic process, nonsense-mediated decay // not recorded
409.05075	497.6229	NM_018748///XM_0	0.008	1.22	***Golga4***	golgi autoantigen, golgin subfamily a, 4	0000042 // protein targeting to Golgi // inferred from electronic annotation///0006355 // regulation of transcripti
2219.8306	2625.3403	NM_001146120///NI	0.007	1.22	***Psap***	prosaposin	0006629 // lipid metabolic process // inferred from electronic annotation///0006665 // sphingolipid metabolic pro
927.72565	1105.54	NM_001040459///XI	0.006	1.22	***Shroom4***	shroom family member 4	0000902 // cell morphogenesis // inferred from sequence or structural similarity///0007015 // actin filament orgar
6579.4795	7937.7295	NM_001171034///NI	0.009	1.22	***Tmbim6***	transmembrane BAX inhibitor motif containing 6	0006915 // apoptotic process // inferred from electronic annotation///0006986 // response to unfolded protein //
740.0734	880.781	NM_009796///XM_0	0.009	1.22	***Capn7***	calpain 7	0006508 // proteolysis // not recorded///0010634 // positive regulation of epithelial cell migration // not recorded
830.67487	984.8433	NM_001276292///NI	0.008	1.22	***Wwp1***	WW domain containing E3 ubiquitin protein ligase 1	0016567 // protein ubiquitination // inferred from direct assay///0016567 // protein ubiquitination // traceable au
18.76382	22.56707	NM_146086///XM_0	0.006	1.21	***Pde6a***	phosphodiesterase 6A, cGMP-specific, rod, alpha	0007165 // signal transduction // inferred from electronic annotation///0007601 // visual perception // inferred fr
1052.1464	1251.0677	NM_025611	0.009	1.21	***Cul7***	cullin 7	0000226 // microtubule cytoskeleton organization // inferred from sequence or structural similarity///0000281 //
918.99493	1085.121	NM_001290792///NI	0.007	1.21	***Wdr45***	WD repeat domain 45	0000045 // autophagic vacuole assembly // not recorded///0006914 // autophagy // not recorded///0050790 // re
2174.6592	2561.4438	NM_144900	0.010	1.21	***Atp1a1***	ATPase, Na+/K+ transporting, alpha 1 polypeptide	0002026 // regulation of the force of heart contraction // inferred from mutant phenotype///0002028 // regulatio
2213.297	2597.9736	NM_206924	0.009	1.21	***Jtb***	jumping translocation breakpoint	0000910 // cytokinesis // not recorded///0006915 // apoptotic process // inferred from electronic annotation///00
232.40111	282.9332	NM_001170433///NI	0.007	1.21	***Ppfibp1***	PTPRF interacting protein, binding protein 1 (liprin beta 1)	0005515 // protein binding // inferred from electronic annotation
523.5016	627.2216	NM_001165894///NI	0.006	1.21	***Akt1***	thymoma viral proto-oncogene 1	0000060 // protein import into nucleus, translocation // not recorded///0001649 // osteoblast differentiation // in
342.91522	411.1354	NM_001253885///NI	0.009	1.21	***Apbb1***	amyloid beta (A4) precursor protein-binding, family B, member 1	0001764 // neuron migration // inferred from genetic interaction///0006302 // double-strand break repair // infer
931.9625	1098.9344	NM_011682///XM_0	0.007	1.21	***Utrn***	utrophin	0001954 // positive regulation of cell-matrix adhesion // not recorded///0007527 // adult somatic muscle develop
694.9422	819.30725	NM_001252326///NI	0.010	1.21	***Pan2***	PAN2 polyA specific ribonuclease subunit homolog (S. cerevisiae)	0000184 // nuclear-transcribed mRNA catabolic process, nonsense-mediated decay // inferred from electronic ann
76.91728	68.19983	NM_029182	0.008	-1.19	***Rasd2***	RASD family, member 2	0001963 // synaptic transmission, dopaminergic // inferred from mutant phenotype///0006184 // GTP catabolic pr

[Nrf2-KO, Air, PBS]	[Nrf2-KO, Air, SFN]	RefSeq Transcript ID	p	FC PBS:SFN	Gene Symbol	Gene Title	Gene Ontology
11392.251	9199.467	NM_001077529///NI	0.009	-1.20	***Nme2***	NME/NM23 nucleoside diphosphate kinase 2	0002762 // negative regulation of myeloid leukocyte differentiation // not recorded///0006165 // nucleoside diphc
1646.1176	1348.7761	NM_001161724///NI	0.010	-1.20	***Ilk***	integrin linked kinase	0001558 // regulation of cell growth // not recorded///0001658 // branching involved in ureteric bud morphogene
205.99272	177.18608	NM_001042556///NI	0.009	-1.20	***Rpf2***	ribosome production factor 2 homolog (S. cerevisiae)	0044822 // poly(A) RNA binding // not recorded
125.604965	111.07297	NM_029101///XR_38	0.008	-1.20	***Rrp7a***	ribosomal RNA processing 7 homolog A (S. cerevisiae)	0000166 // nucleotide binding // inferred from electronic annotation///0003723 // RNA binding // inferred from el
48.495747	41.14084	NM_008024	0.007	-1.21	***Foxl1***	forkhead box L1	0000122 // negative regulation of transcription from RNA polymerase II promoter // ---///0006351 // transcription
405.3909	335.4976	NM_026062	0.009	-1.21	***Fam69a***	family with sequence similarity 69, member A	0005515 // protein binding // inferred from physical interaction
569.2749	467.8062	NM_001024384///NI	0.008	-1.21	***Crls1***	cardiolipin synthase 1	0006629 // lipid metabolic process // inferred from electronic annotation///0008654 // phospholipid biosynthetic
421.15665	348.99915	NM_153160///XM_0	0.010	-1.21	***Zcchc17***	zinc finger, CCHC domain containing 17	0003676 // nucleic acid binding // inferred from electronic annotation///0003723 // RNA binding // traceable auth
238.09058	201.72334	NM_001127338///NI	0.008	-1.21	***Aldh7a1***	aldehyde dehydrogenase family 7, member A1	0008152 // metabolic process // inferred from electronic annotation///0019285 // glycine betaine biosynthetic prc
112.27865	98.583206	NM_027201///XM_0	0.010	-1.21	***Zfp511***	zinc finger protein 511	0006351 // transcription, DNA-templated // inferred from electronic annotation///0006355 // regulation of transcı
119.83843	104.907814	NM_138649///XM_0	0.007	-1.21	***Syt17***	synaptotagmin XVII	0006810 // transport // inferred from electronic annotation///0006887 // exocytosis // inferred from electronic an
73.42475	63.656742	NM_001044386///NI	0.009	-1.22	***Zfa-ps///Zfx***	zinc finger protein, autosomal, pseudogene///zinc finger protein X-linked	0001541 // ovarian follicle development // inferred from mutant phenotype///0006351 // transcription, DNA-temp
117.91455	103.2395	NM_001276442///NI	0.010	-1.22	***Acy1***	aminoacylase 1	0006508 // proteolysis // inferred from electronic annotation///0006520 // cellular amino acid metabolic process /
60.109657	51.34152	NM_010827///XM_0	0.009	-1.22	***Msc***	musculin	0000122 // negative regulation of transcription from RNA polymerase II promoter // inferred from direct assay///0
1380.3821	1116.8933	NM_001163256///NI	0.009	-1.22	***Fblim1***	filamin binding LIM protein 1	0007155 // cell adhesion // inferred from electronic annotation///0008360 // regulation of cell shape // inferred fr
277.51715	230.42418	NM_001134902///NI	0.007	-1.22	***AU019823***	expressed sequence AU019823	
2221.04	1777.0048	NM_011967	0.007	-1.22	***Psma5***	proteasome (prosome, macropain) subunit, alpha type 5	0006508 // proteolysis // inferred from electronic annotation///0006511 // ubiquitin-dependent protein catabolic
16.359215	13.376959	NM_011272	0.008	-1.22	***Rln1***	relaxin 1	0007188 // adenylate cyclase-modulating G-protein coupled receptor signaling pathway // not recorded///001074
291.30392	241.39128	NM_001085508///XI	0.006	-1.22	***Tmem8b***	transmembrane protein 8B	0007155 // cell adhesion // inferred from electronic annotation///0007160 // cell-matrix adhesion // not recorded
515.34766	421.1316	NM_001080742///NI	0.007	-1.22	***Vamp5***	vesicle-associated membrane protein 5	0006887 // exocytosis // not recorded///0006906 // vesicle fusion // not recorded///0007269 // neurotransmitter
890.584	719.23444	NM_024282///XM_0	0.005	-1.22	***Desi2***	desumoylating isopeptidase 2	0006508 // proteolysis // inferred from electronic annotation
146.01375	126.43931	NM_001014395///XI	0.008	-1.22	***Fbxw22***	F-box and WD-40 domain protein 22	0005515 // protein binding // inferred from electronic annotation
265.46164	220.79482	NM_001163239///NI	0.008	-1.22	***Nqo2***	NAD(P)H dehydrogenase, quinone 2	0007613 // memory // inferred from electronic annotation///0055114 // oxidation-reduction process // inferred fr
65.82909	56.313026	NM_001033312///XI	0.005	-1.22	***Fbxl18***	F-box and leucine-rich repeat protein 18	0005515 // protein binding // inferred from electronic annotation
307.13614	253.10446	NM_011785///XM_0	0.010	-1.22	***Akt3***	thymoma viral proto-oncogene 3	0000002 // mitochondrial genome maintenance // not recorded///0006468 // protein phosphorylation // not reco
989.39264	794.8956	NM_008723///NR_0	0.006	-1.23	***Npm3///Npm3-ps1***	nucleoplasmin 3///nucleoplasmin 3, pseudogene 1	0006364 // rRNA processing // inferred from direct assay///0009303 // rRNA transcription // inferred from direct a
989.39264	794.8956	NM_008723///NR_0	0.006	-1.23	***Npm3///Npm3-ps1***	nucleoplasmin 3///nucleoplasmin 3, pseudogene 1	0006364 // rRNA processing // inferred from direct assay///0009303 // rRNA transcription // inferred from direct a
539.2666	438.72354	NM_019432	0.006	-1.23	***Tmem37***	transmembrane protein 37	0006810 // transport // inferred from electronic annotation///0006811 // ion transport // inferred from electronic
21.371939	17.196777	NR_045047	0.006	-1.23	***Tunar***	Tcl1 upstream neural differentiation associated RNA	
879.75104	706.1113	NM_015792///XM_0	0.007	-1.23	***Fbxo18***	F-box protein 18	0008152 // metabolic process // inferred from electronic annotation///0032508 // DNA duplex unwinding // inferr
192.7467	163.50467	NM_207298///XM_0	0.010	-1.23	***Cercam***	cerebral endothelial cell adhesion molecule	0007155 // cell adhesion // not recorded///0009103 // lipopolysaccharide biosynthetic process // inferred from ele
453.47842	368.91324	NM_172575///NM_1	0.008	-1.23	***Zfp277***	zinc finger protein 277	0070301 // cellular response to hydrogen peroxide // inferred from mutant phenotype///2000772 // regulation of
117.295425	101.215195	NM_148938	0.004	-1.23	***Slc1a3***	solute carrier family 1 (glial high affinity glutamate transporter), member 3	0006536 // glutamate metabolic process // inferred from mutant phenotype///0006537 // glutamate biosynthetic
622.91656	501.30685	NM_009148///XM_0	0.010	-1.23	***Exoc4***	exocyst complex component 4	0006612 // protein targeting to membrane // not recorded///0006810 // transport // inferred from electronic ann
43.16491	35.877193	XR_105083///XR_107	0.009	-1.23	***Gm12758***	predicted gene 12758	
174.06857	148.93547	NM_001159331///NI	0.005	-1.23	***N6amt1***	N-6 adenine-specific DNA methyltransferase 1 (putative)	0006479 // protein methylation // not recorded///0030307 // positive regulation of cell growth // not recorded///
157.58698	135.20943	NM_172945///XM_0	0.006	-1.23	***Ankrd13b***	ankyrin repeat domain 13b	0005515 // protein binding // inferred from electronic annotation
929.68524	743.5187	NM_011591///XM_0	0.006	-1.23	***Timm17b***	translocase of inner mitochondrial membrane 17b	0006810 // transport // inferred from electronic annotation///0006886 // intracellular protein transport // inferred
95.04872	81.67747	NM_010844///XM_0	0.009	-1.23	***Muc5ac***	mucin 5, subtypes A and C, tracheobronchial/gastric	0010193 // response to ozone // inferred from electronic annotation///0010477 // response to sulfur dioxide // inf
86.94642	74.40261	NM_001160330	0.010	-1.24	***Mocs3***	molybdenum cofactor synthesis 3	0002098 // tRNA wobble uridine modification // not recorded///0002143 // tRNA wobble position uridine thiolatic
2443.5093	1936.4417	NM_011970///XM_0	0.008	-1.24	***Psmb2***	proteasome (prosome, macropain) subunit, beta type 2	0006508 // proteolysis // inferred from electronic annotation///0010243 // response to organonitrogen compounc
493.4236	397.2164	NM_025798///XM_0	0.007	-1.24	***Hint3***	histidine triad nucleotide binding protein 3	0008152 // metabolic process // inferred from electronic annotation
351.0264	285.8242	NM_001271873///NI	0.005	-1.24	***Ano10***	anoctamin 10	0006812 // cation transport // not recorded///0006821 // chloride transport // not recorded///1902476 // chloride
1409.6046	1121.9183	NM_011185	0.007	-1.24	***Psmb1***	proteasome (prosome, macropain) subunit, beta type 1	0006508 // proteolysis // inferred from electronic annotation///0051603 // proteolysis involved in cellular protein
55.610065	46.34353	NM_008189	0.008	-1.24	***Guca1a***	guanylate cyclase activator 1a (retina)	0007165 // signal transduction // inferred from electronic annotation///0007601 // visual perception // inferred fr
255.35707	211.1263	NM_025931	0.007	-1.24	***Ift27***	intraflagellar transport 27	0006184 // GTP catabolic process // inferred from electronic annotation///0007165 // signal transduction // inferr
1503.741	1198.7594	NM_026114	0.010	-1.24	***Eif2s1***	eukaryotic translation initiation factor 2, subunit 1 alpha	0006412 // translation // inferred from direct assay///0006412 // translation // not recorded///0006413 // transla
158.36768	135.47089	NM_001081292///NI	0.009	-1.24	***Map3k10***	mitogen-activated protein kinase kinase kinase 10	0000165 // MAPK cascade // inferred from electronic annotation///0000186 // activation of MAPKK activity // infe
49.822277	40.91523	NM_028474///XM_0	0.006	-1.24	***Ptchd4***	patched domain containing 4	0007165 // signal transduction // inferred from electronic annotation
266.6047	220.19589	NM_008738///XM_0	0.008	-1.24	***Nrtn***	neurturin	0001755 // neural crest cell migration // not recorded///0007169 // transmembrane receptor protein tyrosine kina
410.37567	332.3897	NM_134024	0.005	-1.24	***Tubg1***	tubulin, gamma 1	0000212 // meiotic spindle organization // inferred from expression pattern///0006184 // GTP catabolic process //
2634.9792	2072.5742	NM_011971	0.009	-1.24	***Psmb3***	proteasome (prosome, macropain) subunit, beta type 3	0006508 // proteolysis // not recorded///0030163 // protein catabolic process // traceable author statement///00
123.25133	105.12771	NM_007922	0.010	-1.24	***Elk1***	ELK1, member of ETS oncogene family	0006351 // transcription, DNA-templated // inferred from electronic annotation///0006355 // regulation of transcı
787.7596	625.82385	NM_025574	0.010	-1.24	***Pyurf***	Pigy upstream reading frame	0006506 // GPI anchor biosynthetic process // not recorded///0009893 // positive regulation of metabolic process
137.68909	117.15207	NM_001037736///NI	0.005	-1.24	***Arhgef10***	Rho guanine nucleotide exchange factor (GEF) 10	0022011 // myelination in peripheral nervous system // not recorded///0032319 // regulation of Rho GTPase activ
46.323574	37.967438	XR_376884///XR_376	0.006	-1.24	***2210406O10Rik***	RIKEN cDNA 2210406O10 gene	
24.71157	19.674719	NM_029416///XM_0	0.007	-1.24	***Klf17***	Kruppel-like factor 17	0006351 // transcription, DNA-templated // inferred from electronic annotation///0006355 // regulation of transcı
649.7859	516.76373	NM_001177945///NI	0.005	-1.24	***Aamdc***	adipogenesis associated Mth938 domain containing	0043066 // negative regulation of apoptotic process // inferred from mutant phenotype///0045600 // positive regu
232.44029	192.35313	NM_001033314///XI	0.003	-1.25	***Ccdc61***	coiled-coil domain containing 61	0005813 // centrosome // not recorded
989.9023	783.88446	NM_029354///XM_0	0.004	-1.25	***Mzt2***	mitotic spindle organizing protein 2	0005737 // cytoplasm // inferred from electronic annotation///0005813 // centrosome // not recorded///0005815
101.54686	86.47637	NM_001294308///NI	0.008	-1.25	***Ngb***	neuroglobin	0006810 // transport // inferred from electronic annotation///0006915 // apoptotic process // inferred from electı
612.22876	488.40732	NM_001164212///NI	0.009	-1.25	***Rerg***	RAS-like, estrogen-regulated, growth-inhibitor	0006184 // GTP catabolic process // not recorded///0007165 // signal transduction // inferred from electronic ann
384.34775	310.46234	NM_026113///XM_0	0.006	-1.25	***Gtf3c6***	general transcription factor IIIC, polypeptide 6, alpha	0006351 // transcription, DNA-templated // not recorded///0006383 // transcription from RNA polymerase III pror
234.521	193.99869	NM_026015///XM_0	0.008	-1.25	***Zmat5***	zinc finger, matrin type 5	0006397 // mRNA processing // inferred from electronic annotation///0008380 // RNA splicing // inferred from ele
470.45953	377.14816	NM_021428///XM_0	0.007	-1.25	***Dexi***	dexamethasone-induced transcript	
46.05425	37.707077	NM_001122639///NI	0.008	-1.25	***Galnt9***	UDP-N-acetyl-alpha-D-galactosamine:polypeptide N-acetylgalactosaminyltransferas	0006486 // protein glycosylation // inferred from electronic annotation///0008152 // metabolic process // inferred
66.13625	55.53621	NM_028411///XM_0	0.007	-1.25	***Tmem138***	transmembrane protein 138	0030030 // cell projection organization // inferred from electronic annotation///0042384 // cilium assembly // not
39.58425	32.221634	NM_001080934///XI	0.009	-1.25	***Slc16a5***	solute carrier family 16 (monocarboxylic acid transporters), member 5	0006810 // transport // inferred from electronic annotation///0055085 // transmembrane transport // inferred frc
720.2045	570.7497	NM_025375///XM_0	0.006	-1.25	***Wbscr22***	Williams Beuren syndrome chromosome region 22	0006351 // transcription, DNA-templated // inferred from electronic annotation///0006355 // regulation of transcı
163.35278	138.26282	NM_001254762///NI	0.007	-1.25	***Mospd3***	motile sperm domain containing 3	0007507 // heart development // inferred from mutant phenotype
156.05014	131.78233	NM_009088///XM_0	0.008	-1.25	***Polr1a***	polymerase (RNA) I polypeptide A	0006351 // transcription, DNA-templated // inferred from electronic annotation///0006360 // transcription from R

[Nrf2-KO, Air, PBS]	[Nrf2-KO, Air, SFN]	RefSeq Transcript ID	p	FC PBS:SFN	Gene Symbol	Gene Title	Gene Ontology
535.29517	425.36746	NM_001162521///NI	0.005	-1.25	***Dguok***	deoxyguanosine kinase	0006139 // nucleobase-containing compound metabolic process // traceable author statement///0006468 // prote
26.434654	20.879622	NM_001007573///XI	0.006	-1.25	***Maneal***	mannosidase, endo-alpha-like	0008152 // metabolic process // inferred from electronic annotation
1734.5222	1356.5464	NM_019953///XM_0	0.005	-1.25	***Cnpy2***	canopy 2 homolog (zebrafish)	0010629 // negative regulation of gene expression // inferred from mutant phenotype///0010988 // regulation of
162.99133	137.81621	NM_001081179///NI	0.006	-1.25	***Heatr5b***	HEAT repeat containing 5B	0005488 // binding // inferred from electronic annotation///0005515 // protein binding // inferred from physical ir
1230.6725	963.49365	NM_026126	0.006	-1.26	***Fundc2***	FUN14 domain containing 2	0005634 // nucleus // not recorded///0005739 // mitochondrion // not recorded
230.83437	189.71242	NM_172252///XM_0	0.007	-1.26	***Mrpl21***	mitochondrial ribosomal protein L21	0006412 // translation // inferred from electronic annotation
284.14886	229.68207	NM_025525	0.003	-1.26	***Rnf113a2***	ring finger protein 113A2	0005515 // protein binding // inferred from electronic annotation///0008270 // zinc ion binding // inferred from el
1228.8027	963.52545	NM_013902	0.003	-1.26	***Fkbp3***	FK506 binding protein 3	0000413 // protein peptidyl-prolyl isomerization // not recorded///0006457 // protein folding // inferred from elec
558.4193	442.84793	NM_026230///NM_2	0.009	-1.26	***H2afy2///H2afy3***	H2A histone family, member Y2///H2A histone family, member Y3	0000122 // negative regulation of transcription from RNA polymerase II promoter // not recorded///0006334 // nu
65.65393	54.662155	NM_008593	0.009	-1.26	***Foxd2***	forkhead box D2	0000122 // negative regulation of transcription from RNA polymerase II promoter // not recorded///0001658 // br
449.9475	357.03183	NM_029632	0.005	-1.26	***Ppp1r11***	protein phosphatase 1, regulatory (inhibitor) subunit 11	0043086 // negative regulation of catalytic activity // inferred from direct assay
33.219917	26.500816	NM_009890	0.010	-1.26	***Ch25h***	cholesterol 25-hydroxylase	0006629 // lipid metabolic process // inferred from electronic annotation///0006633 // fatty acid biosynthetic proc
348.38257	278.60934	NM_001285899///NI	0.005	-1.26	***Eri3***	exoribonuclease 3	0008152 // metabolic process // inferred from electronic annotation///0090305 // nucleic acid phosphodiester bon
98.80254	83.39764	NM_001113211///NI	0.010	-1.26	***1700012D01Rik///Tmem194***	RIKEN cDNA 1700012D01 gene///transmembrane protein 194	0016020 // membrane // inferred from electronic annotation///0016021 // integral component of membrane // in
64.66186	53.546825	NM_023695///XM_0	0.006	-1.26	***Crybb1***	crystallin, beta B1	0005212 // structural constituent of eye lens // not recorded///0005515 // protein binding // inferred from physica
2929.6816	2277.4053	NM_025511	0.008	-1.26	***Cox20***	COX20 Cox2 chaperone	0005739 // mitochondrion // inferred from direct assay///0005739 // mitochondrion // not recorded///0016020 //
39.658688	31.834368	NM_008262	0.003	-1.26	***Onecut1***	one cut domain, family member 1	0001889 // liver development // inferred from genetic interaction///0001952 // regulation of cell-matrix adhesion
187.26993	155.51958	NM_023556///XM_0	0.007	-1.26	***Mvk***	mevalonate kinase	0006629 // lipid metabolic process // inferred from electronic annotation///0006694 // steroid biosynthetic proces
764.88684	598.97253	NM_023587///XM_0	0.003	-1.26	***Ptplb***	protein tyrosine phosphatase-like (proline instead of catalytic arginine), member b	0006629 // lipid metabolic process // inferred from electronic annotation///0006631 // fatty acid metabolic proces
1018.7957	799.08026	NM_011895///XM_0	0.006	-1.26	***Slc35a1***	solute carrier family 35 (CMP-sialic acid transporter), member 1	0006810 // transport // inferred from electronic annotation///0008643 // carbohydrate transport // inferred from
24.682493	19.322618	NM_001099631	0.003	-1.26	***Sh2d5***	SH2 domain containing 5	0005515 // protein binding // inferred from electronic annotation
296.38144	238.13055	NM_001163170	0.006	-1.26	***Lix1l***	Lix1-like	
415.78082	330.5474	NM_001122960///NI	0.005	-1.26	***Ccdc53***	coiled-coil domain containing 53	0005515 // protein binding // inferred from electronic annotation
132.59949	110.72405	NM_029857///XM_0	0.005	-1.26	***Tmco4***	transmembrane and coiled-coil domains 4	0016020 // membrane // inferred from electronic annotation///0016021 // integral component of membrane // in
208.34454	171.5037	NM_027323///XM_0	0.006	-1.26	***Srrd***	SRR1 domain containing	0048511 // rhythmic process // inferred from electronic annotation
80.88303	67.87937	NM_027982///XM_0	0.006	-1.26	***Ppm1j***	protein phosphatase 1J	0006470 // protein dephosphorylation // inferred from direct assay
267.62848	216.19978	NM_028448///XM_0	0.003	-1.27	***Cenpv***	centromere protein V	0001667 // ameboidal cell migration // not recorded///0007049 // cell cycle // inferred from electronic annotation
118.02771	99.15969	NM_177391///XM_0	0.002	-1.27	***Fam109b***	family with sequence similarity 109, member B	0001881 // receptor recycling // not recorded///0007032 // endosome organization // not recorded///0042147 //
32.795536	25.93246	NM_001029929///XI	0.009	-1.27	***Zmynd15***	zinc finger, MYND-type containing 15	0006351 // transcription, DNA-templated // inferred from electronic annotation///0006355 // regulation of transcı
103.3836	86.55347	NM_029926	0.006	-1.27	***Irak4***	interleukin-1 receptor-associated kinase 4	0001816 // cytokine production // inferred from direct assay///0002376 // immune system process // inferred fror
404.31158	319.5324	NM_001253731///NI	0.004	-1.27	***Ints9***	integrator complex subunit 9	0016180 // snRNA processing // not recorded
511.71274	402.5081	NM_001163454///NI	0.008	-1.27	***Prorsd1***	prolyl-tRNA synthetase domain containing 1	0006450 // regulation of translational fidelity // inferred from electronic annotation
148.44363	123.3462	NM_145432///XM_0	0.007	-1.27	***Heatr6***	HEAT repeat containing 6///Mus musculus HEAT repeat containing 6 (Heatr6), mRN	0005488 // binding // inferred from electronic annotation///0044822 // poly(A) RNA binding // not recorded
60.447395	49.469555	NM_009314	0.003	-1.27	***Tacr2***	tachykinin receptor 2	0007165 // signal transduction // inferred from electronic annotation///0007186 // G-protein coupled receptor sig
108.19862	90.95807	NM_030060///XM_0	0.005	-1.27	***Batf3***	basic leucine zipper transcription factor, ATF-like 3	0006351 // transcription, DNA-templated // inferred from electronic annotation///0006355 // regulation of transcı
294.87262	235.92267	NM_174960	0.004	-1.27	***Gimap9***	GTPase, IMAP family member 9	0005525 // GTP binding // inferred from electronic annotation
1913.2892	1485.0658	NM_028632	0.007	-1.27	***Fcf1***	FCF1 small subunit (SSU) processome component homolog (S. cerevisiae)	0006364 // rRNA processing // inferred from electronic annotation///0042254 // ribosome biogenesis // inferred fı
607.50995	472.9059	NM_001286033///NI	0.006	-1.27	***Stx2***	syntaxin 2	0006886 // intracellular protein transport // inferred from electronic annotation///0007340 // acrosome reaction /
67.4342	55.28693	NM_176917///XM_0	0.007	-1.27	***Mettl4***	methyltransferase like 4	0006139 // nucleobase-containing compound metabolic process // inferred from electronic annotation///0032259
1108.5682	865.7077	NM_026455	0.006	-1.27	***Fam32a***	family with sequence similarity 32, member A	0006915 // apoptotic process // inferred from electronic annotation///0007049 // cell cycle // inferred from electr
81.807014	67.638535	NM_020596	0.006	-1.28	***Egr4***	early growth response 4	0006351 // transcription, DNA-templated // inferred from electronic annotation///0006355 // regulation of transcı
1756.3993	1353.6064	NM_016798///XM_0	0.006	-1.28	***Pdlim3***	PDZ and LIM domain 3	0007015 // actin filament organization // inferred from direct assay///0007507 // heart development // inferred fr
945.5018	730.1995	NM_001271724///NI	0.003	-1.28	***Surf1***	surfeit gene 1	0006119 // oxidative phosphorylation // not recorded///0006754 // ATP biosynthetic process // not recorded///00
238.46631	192.21892	NM_029321///XM_0	0.002	-1.28	***Ttc32***	tetratricopeptide repeat domain 32	0005515 // protein binding // inferred from electronic annotation///0042802 // identical protein binding // inferre
474.50665	371.48984	NM_152895///XM_0	0.006	-1.28	***Kdm5b***	lysine (K)-specific demethylase 5B	0006351 // transcription, DNA-templated // inferred from electronic annotation///0006355 // regulation of transcı
140.98708	117.24186	NM_008451///NR_0	0.008	-1.28	***Klc2***	kinesin light chain 2	0007018 // microtubule-based movement // traceable author statement///0008088 // axon cargo transport // infe
362.21988	284.94547	NM_025455///XM_0	0.004	-1.28	***Ccdc28b***	coiled coil domain containing 28B	0030030 // cell projection organization // inferred from electronic annotation///0042384 // cilium assembly // not
52.466618	41.99371	NM_001081971	0.005	-1.28	***Ankrd63***	ankyrin repeat domain 63	0005515 // protein binding // inferred from electronic annotation
482.1801	375.5232	NM_021436///XM_0	0.005	-1.28	***Tmeff1***	transmembrane protein with EGF-like and two follistatin-like domains 1	0007275 // multicellular organismal development // inferred from electronic annotation
457.9985	357.0014	NM_028493///XM_0	0.007	-1.28	***Rhobtb3***	Rho-related BTB domain containing 3	0006200 // ATP catabolic process // not recorded///0006810 // transport // inferred from electronic annotation///
909.24036	703.30695	NM_173740	0.009	-1.28	***Maoa***	monoamine oxidase A	0006584 // catecholamine metabolic process // inferred from electronic annotation///0042135 // neurotransmitte
775.07916	597.96216	NM_001168244///NI	0.010	-1.28	***Syce2***	synaptonemal complex central element protein 2	0007049 // cell cycle // inferred from electronic annotation///0007126 // meiotic nuclear division // inferred from
529.28296	412.5257	NM_009675///XM_0	0.006	-1.28	***Aoc3***	amine oxidase, copper containing 3	0002523 // leukocyte migration involved in inflammatory response // not recorded///0002675 // positive regulatic
978.7117	752.5945	NM_030131	0.003	-1.28	***Cnih4***	cornichon homolog 4 (Drosophila)	0035556 // intracellular signal transduction // inferred from electronic annotation
52.12504	41.544483	NM_033145	0.008	-1.28	***Lcn8***	lipocalin 8	0006810 // transport // inferred from electronic annotation///0009725 // response to hormone // inferred from di
101.99454	84.24645	NM_008035///XM_0	0.004	-1.28	***Folr2***	folate receptor 2 (fetal)	0006620 // posttranslational protein targeting to membrane // traceable author statement///0006810 // transport
212.474	171.83119	NM_133932///XM_0	0.009	-1.28	***Tada3***	transcriptional adaptor 3	0001932 // regulation of protein phosphorylation // inferred from mutant phenotype///0006351 // transcription, I
460.60928	358.6683	NM_001285826///NI	0.004	-1.28	***Ppil3***	peptidylprolyl isomerase (cyclophilin)-like 3	0000413 // protein peptidyl-prolyl isomerization // inferred from electronic annotation///0006397 // mRNA proces
1726.8342	1324.4606	NM_021519///XM_0	0.004	-1.28	***Edf1///Gm11964***	endothelial differentiation-related factor 1///predicted gene 11964	0006351 // transcription, DNA-templated // inferred from electronic annotation///0006355 // regulation of transcı
1726.8342	1324.4606	NM_021519///XM_0	0.004	-1.28	***Edf1///Gm11964***	endothelial differentiation-related factor 1///predicted gene 11964	0006351 // transcription, DNA-templated // inferred from electronic annotation///0006355 // regulation of transcı
1353.7998	1038.3312	NM_001166493///NI	0.008	-1.28	***Rasgrp3***	RAS, guanyl releasing protein 3	0007264 // small GTPase mediated signal transduction // inferred from electronic annotation///0007265 // Ras prc
34.8133	27.217539	NM_053178///XM_0	0.010	-1.28	***Acsbg1***	acyl-CoA synthetase bubblegum family member 1	0000038 // very long-chain fatty acid metabolic process // not recorded///0000038 // very long-chain fatty acid me
556.77026	432.66962	NM_078479///XM_0	0.004	-1.29	***Mrps21***	mitochondrial ribosomal protein S21	0006412 // translation // inferred from electronic annotation
102.587	84.5812	NM_001199151///NI	0.002	-1.29	***Sncaip***	synuclein, alpha interacting protein (synphilin)	0042417 // dopamine metabolic process // not recorded///0046928 // regulation of neurotransmitter secretion //
633.3487	488.12845	NM_029760///XM_0	0.007	-1.29	***Nubpl***	nucleotide binding protein-like	0032981 // mitochondrial respiratory chain complex I assembly // not recorded///0070584 // mitochondrion morp
167.7549	138.43306	NM_001081441///NI	0.008	-1.29	***Wdr86***	WD repeat domain 86	
121.73956	100.23331	NM_013848///XM_0	0.008	-1.29	***Ermap***	erythroblast membrane-associated protein	0005515 // protein binding // inferred from electronic annotation
325.30963	254.96643	NM_028232///XM_0	0.007	-1.29	***Sgol1***	shugoshin-like 1 (S. pombe)	0007049 // cell cycle // inferred from electronic annotation///0007059 // chromosome segregation // not recordec
40.117584	31.673004	NM_001160353///XI	0.006	-1.29	***Grm2///LOC102636702***	glutamate receptor, metabotropic 2///uncharacterized LOC102636702	0001662 // behavioral fear response // traceable author statement///0007165 // signal transduction // inferred frc
117.50854	97.1269	NM_172747///XM_0	0.005	-1.29	***Kctd13***	potassium channel tetramerisation domain containing 13	0006260 // DNA replication // not recorded///0016477 // cell migration // not recorded///0016567 // protein ubic
1548.231	1186.4258	NM_024478	0.008	-1.29	***Grpel1***	GrpE-like 1, mitochondrial	0006457 // protein folding // inferred from electronic annotation///0050790 // regulation of catalytic activity // inf

[Nrf2-KO, Air, PBS]	[Nrf2-KO, Air, SFN]	RefSeq Transcript ID	p	FC PBS:SFN	Gene Symbol	Gene Title	Gene Ontology
476.4157	369.01642	NM_013586	0.002	-1.29	***Loxl3***	lysyl oxidase-like 3	0001837 // epithelial to mesenchymal transition // not recorded///0006898 // receptor-mediated endocytosis // ir
114.405396	94.29895	NM_028825///XM_0	0.005	-1.29	***Tex37***	testis expressed 37	0005634 // nucleus // inferred from electronic annotation
125.175735	102.549065	NM_172462	0.004	-1.29	***Zfp11***	zinc finger protein 11	0006355 // regulation of transcription, DNA-templated // not recorded
195.63348	158.35936	NM_026144	0.003	-1.29	***Dhdds***	dehydrodolichyl diphosphate synthase	0006486 // protein glycosylation // inferred from electronic annotation///0008152 // metabolic process // inferred
149.88881	122.41641	NM_001293791///NM	0.001	-1.29	***Ints10***	integrator complex subunit 10	0016180 // snRNA processing // not recorded
250.27039	198.5933	NM_001110017///NM	0.002	-1.29	***Dzip3***	DAZ interacting protein 3, zinc finger	0000209 // protein polyubiquitination // not recorded///0016567 // protein ubiquitination // inferred from electro
90.20711	73.66294	NM_174877///XM_0	0.003	-1.29	***Zar1***	zygote arrest 1	0007275 // multicellular organismal development // inferred from electronic annotation
25.734549	19.6784	NM_130457///XM_0	0.004	-1.29	***Cntnap4***	contactin associated protein-like 4	0007155 // cell adhesion // inferred from electronic annotation///0032225 // regulation of synaptic transmission, c
509.5329	391.52274	NM_001284409///NM	0.006	-1.29	***Casp3***	caspase 3	0001782 // B cell homeostasis // inferred from mutant phenotype///0001836 // release of cytochrome c from mito
76.96827	62.538654	NM_009779	0.004	-1.30	***C3ar1***	complement component 3a receptor 1	0002430 // complement receptor mediated signaling pathway // not recorded///0002462 // tolerance induction to
202.84416	163.37778	NM_027122	0.005	-1.30	***Mfsd3***	major facilitator superfamily domain containing 3	0006810 // transport // inferred from electronic annotation///0055085 // transmembrane transport // inferred fro
107.17194	87.640625	NM_009767///XM_0	0.006	-1.30	***Chic1***	cysteine-rich hydrophobic domain 1	0005886 // plasma membrane // inferred from electronic annotation///0016020 // membrane // inferred from ele
109.01	89.50757	NM_024171///XM_0	0.009	-1.30	***LOC101055909///Sec61b***	protein transport protein Sec61 subunit beta-like///Sec61 beta subunit	0000060 // protein import into nucleus, translocation // not recorded///0006810 // transport // inferred from elec
382.34888	296.6227	NM_013841	0.003	-1.30	***Vps45***	vacuolar protein sorting 45 (yeast)	0006810 // transport // inferred from electronic annotation///0006904 // vesicle docking involved in exocytosis //
867.3517	662.38306	NM_026616	0.002	-1.30	***Rnaseh2c***	ribonuclease H2, subunit C	0006401 // RNA catabolic process // not recorded
501.81345	385.24234	NM_197987///XM_0	0.003	-1.30	***Trim37***	tripartite motif-containing 37	0016567 // protein ubiquitination // inferred from electronic annotation///0032088 // negative regulation of NF-ka
183.90825	149.64224	NM_178732///XM_0	0.009	-1.30	***Zfp324***	zinc finger protein 324	0006355 // regulation of transcription, DNA-templated // inferred from electronic annotation
2115.042	1600.8473	NM_007483	0.007	-1.30	***Rhob***	ras homolog gene family, member B	0000910 // cytokinesis // not recorded///0001525 // angiogenesis // inferred from electronic annotation///00061
18.135279	13.792897	NM_001282054///NM	0.009	-1.30	***Msh4***	mutS homolog 4 (E. coli)	0001541 // ovarian follicle development // inferred from mutant phenotype///0006200 // ATP catabolic process //
17.628971	13.596579	NR_045271///XR_37	0.004	-1.30	***Fcor***	Foxo1 corepressor	0001659 // temperature homeostasis // inferred from mutant phenotype///0001678 // cellular glucose homeostas
53.980125	42.4273		0.003	-1.30	***Ccdc42os***	coiled-coil domain containing 42, opposite strand	
169.78061	138.70511	NM_020505///NM_1	0.004	-1.30	***Vav3***	vav 3 oncogene	0001525 // angiogenesis // inferred from electronic annotation///0006906 // vesicle fusion // inferred from geneti
328.12372	254.36638	NM_025784///XM_0	0.003	-1.30	***Bcs1l***	BCS1-like (yeast)	0007005 // mitochondrion organization // not recorded///0008152 // metabolic process // inferred from electroni
156.58208	127.365	NM_001008700///NM	0.006	-1.30	***Il4ra***	interleukin 4 receptor, alpha	0002376 // immune system process // inferred from electronic annotation///0002532 // production of molecular m
221.88066	176.4187	NM_028713///XM_0	0.009	-1.30	***Rftn2***	raftlin family member 2	0033227 // dsRNA transport // inferred from mutant phenotype///0043330 // response to exogenous dsRNA // inf
185.7343	150.49617	NM_001163495///NM	0.006	-1.30	***Arhgap19***	Rho GTPase activating protein 19	0007165 // signal transduction // inferred from electronic annotation///0043547 // positive regulation of GTPase a
171.35478	138.85974	NM_019726///XM_0	0.003	-1.30	***Gps2***	G protein pathway suppressor 2	0000122 // negative regulation of transcription from RNA polymerase II promoter // not recorded///0046329 // ne
126.33621	103.3327	NM_013415	0.007	-1.30	***Atp1b2***	ATPase, Na+/K+ transporting, beta 2 polypeptide	0006200 // ATP catabolic process // not recorded///0006810 // transport // inferred from electronic annotation///
74.2149	59.768456	NM_001290011///NM	0.007	-1.30	***Pemt***	phosphatidylethanolamine N-methyltransferase	0006629 // lipid metabolic process // inferred from electronic annotation///0006644 // phospholipid metabolic pro
63.96999	51.13846	NM_183141///XM_0	0.006	-1.31	***Elfn2***	leucine rich repeat and fibronectin type III, extracellular 2	0010923 // negative regulation of phosphatase activity // not recorded
102.39672	82.71131	NM_031402///XM_0	0.009	-1.31	***Crispld1***	cysteine-rich secretory protein LCCL domain containing 1	0060325 // face morphogenesis // not recorded
44.30608	34.54517		0.009	-1.31	***D14Ertd16e***	DNA segment, Chr 14, ERATO Doi 16, expressed	
2942.6	2202.6	NM_001291108///NM	0.009	-1.31	***Dynlrb1***	dynein light chain roadblock-type 1	0006810 // transport // inferred from electronic annotation///0007632 // visual behavior // not recorded///00081
964.6669	730.3289	NM_001004363	0.007	-1.31	***Nuak1***	NUAK family, SNF1-like kinase, 1	0006468 // protein phosphorylation // not recorded///0006974 // cellular response to DNA damage stimulus // inf
156.2616	125.32957	NM_019833///XM_0	0.010	-1.31	***Fam69b***	family with sequence similarity 69, member B	0005783 // endoplasmic reticulum // inferred from electronic annotation///0005789 // endoplasmic reticulum me
3318.7725	2497.4377	NM_016753	0.003	-1.31	***Lxn***	latexin	0006954 // inflammatory response // inferred from electronic annotation///0010466 // negative regulation of pep
320.244	247.02145	NM_018797///XM_0	0.006	-1.31	***Plxnc1***	plexin C1	0007165 // signal transduction // inferred from electronic annotation///0007275 // multicellular organismal develo
384.0148	294.40323	NM_029339	0.002	-1.31	***Ccdc101***	coiled-coil domain containing 101	0006351 // transcription, DNA-templated // inferred from electronic annotation///0006355 // regulation of transcr
1307.3048	981.4865	NM_026474	0.002	-1.31	***Sugt1***	SGT1, suppressor of G2 allele of SKP1 (S. cerevisiae)	0005515 // protein binding // inferred from electronic annotation
358.14755	274.22086	NM_028354///XM_0	0.002	-1.31	***Tdp1***	tyrosyl-DNA phosphodiesterase 1	0000012 // single strand break repair // inferred from mutant phenotype///0000012 // single strand break repair /
206.38547	163.76834	NM_029037	0.005	-1.31	***Pomk***	protein-O-mannose kinase	0001764 // neuron migration // inferred from mutant phenotype///0006468 // protein phosphorylation // inferred
40.81918	31.549173	NM_001033223///NM	0.003	-1.32	***Lin7a***	lin-7 homolog A (C. elegans)	0006810 // transport // inferred from electronic annotation///0006887 // exocytosis // inferred from electronic an
321.9184	247.47173	NM_025904	0.004	-1.32	***Yae1d1***	Yae1 domain containing 1	
1677.59	1258.4897	NM_028260	0.007	-1.32	***Immp1l***	IMP1 inner mitochondrial membrane peptidase-like (S. cerevisiae)	0006508 // proteolysis // inferred from electronic annotation///0006627 // protein processing involved in protein
1576.7903	1184.5223	NM_021335	0.002	-1.32	***Snrpb2***	U2 small nuclear ribonucleoprotein B	0000398 // mRNA splicing, via spliceosome // inferred from electronic annotation///0006397 // mRNA processing ,
508.1397	383.1983	NM_026033	0.004	-1.32	***Gatad1***	GATA zinc finger domain containing 1	0006355 // regulation of transcription, DNA-templated // inferred from electronic annotation
454.81827	344.5323	NM_025301	0.008	-1.32	***Mrpl17***	mitochondrial ribosomal protein L17	0000002 // mitochondrial genome maintenance // traceable author statement///0006412 // translation // not reco
138.9319	111.432	NM_008961///XM_0	0.004	-1.32	***Pter***	phosphotriesterase related	0009056 // catabolic process // inferred from electronic annotation
257.0675	200.38725	NM_001286030///NM	0.002	-1.32	***Dhx32***	DEAH (Asp-Glu-Ala-His) box polypeptide 32	0008152 // metabolic process // inferred from electronic annotation
179.49509	143.51959	NM_009062///XM_0	0.005	-1.32	***Rgs4***	regulator of G-protein signaling 4	0007186 // G-protein coupled receptor signaling pathway // traceable author statement///0009968 // negative reg
470.15674	355.09567	NM_175535///XM_0	0.002	-1.32	***Arhgap20***	Rho GTPase activating protein 20	0007165 // signal transduction // inferred from electronic annotation///0043547 // positive regulation of GTPase a
34.29709	26.080038	NM_007741	0.002	-1.32	***Col9a2***	collagen, type IX, alpha 2	0005576 // extracellular region // inferred from electronic annotation///0005578 // proteinaceous extracellular ma
372.35675	283.19815	NM_028812///XM_0	0.001	-1.32	***Gtf2e1***	general transcription factor II E, polypeptide 1 (alpha subunit)	0006351 // transcription, DNA-templated // inferred from electronic annotation///0006355 // regulation of transcr
287.86557	222.41658	NM_001004361///NM	0.002	-1.32	***Tpgs2***	tubulin polyglutamylase complex subunit 2	0005737 // cytoplasm // inferred from electronic annotation///0005856 // cytoskeleton // inferred from electronic
1024.6508	769.35126	NM_001083316///NM	0.008	-1.32	***Pdgfra***	platelet derived growth factor receptor, alpha polypeptide	0001553 // luteinization // inferred from genetic interaction///0001553 // luteinization // inferred from mutant ph
443.96613	335.73587	NM_025582	0.006	-1.32	***Fam213b***	family with sequence similarity 213, member B	0001516 // prostaglandin biosynthetic process // inferred from direct assay///0006629 // lipid metabolic process /
292.38272	225.41544	NM_027256///XM_0	0.002	-1.32	***Ints4***	integrator complex subunit 4	0005488 // binding // inferred from electronic annotation///0005515 // protein binding // inferred from electronic
907.28186	679.8016	NM_001164155///NM	0.006	-1.32	***Tnfrsf19***	tumor necrosis factor receptor superfamily, member 19	0001942 // hair follicle development // inferred from genetic interaction///0043123 // positive regulation of I-kapp
61.947998	48.295303	NR_045780	0.003	-1.33	***Zfp652os***	zinc finger protein 652, opposite strand	
258.80014	199.8342	NM_001038230///NM	0.002	-1.33	***Anapc11***	anaphase promoting complex subunit 11	0007049 // cell cycle // inferred from electronic annotation///0007067 // mitotic nuclear division // inferred from
107.020515	85.44347	NM_001033534///XM	0.006	-1.33	***Layn***	layilin	0005540 // hyaluronic acid binding // not recorded///0030246 // carbohydrate binding // inferred from electronic
1219.4607	908.34406	NM_175526///XM_0	0.006	-1.33	***Clec1a***	C-type lectin domain family 1, member a	0030246 // carbohydrate binding // inferred from electronic annotation
91.95072	73.07658	NM_026740	0.002	-1.33	***Slc46a1***	solute carrier family 46, member 1	0006810 // transport // inferred from electronic annotation///0015884 // folic acid transport // not recorded///00
205.46031	161.19536	NM_022419	0.010	-1.33	***Abhd8***	abhydrolase domain containing 8	0008152 // metabolic process // inferred from electronic annotation
582.2013	435.2796	NM_029353///XM_0	0.006	-1.33	***Malsu1***	mitochondrial assembly of ribosomal large subunit 1	0042273 // ribosomal large subunit biogenesis // not recorded///0070130 // negative regulation of mitochondrial t
842.72205	627.8816	NM_146236///XM_0	0.007	-1.33	***Tceal1***	transcription elongation factor A (SII)-like 1	0006351 // transcription, DNA-templated // inferred from electronic annotation///0006355 // regulation of transcr
184.22528	145.36548	NM_177292	0.002	-1.33	***Wscd2***	WSC domain containing 2	0016020 // membrane // inferred from electronic annotation///0016021 // integral component of membrane // in
332.28314	251.74788	NM_178407///XM_0	0.009	-1.33	***Arap2***	ArfGAP with RhoGAP domain, ankyrin repeat and PH domain 2	0007165 // signal transduction // inferred from electronic annotation///0032312 // regulation of ARF GTPase activ
62.405388	48.419334	NM_013788	0.009	-1.33	***Peg12***	paternally expressed 12	0000578 // embryonic axis specification // inferred from direct assay///0016055 // Wnt signaling pathway // inferr
337.7341	256.47308	NM_026933	0.010	-1.33	***Triap1***	TP53 regulated inhibitor of apoptosis 1	0006810 // transport // inferred from electronic annotation///0006869 // lipid transport // inferred from electroni
577.74335	431.9178	NM_027181///XM_0	0.010	-1.33	***Gm6851///Gm7301///Pin4***	predicted pseudogene 6851///predicted gene 7301///protein (peptidyl-prolyl cis/tr	0000413 // protein peptidyl-prolyl isomerization // inferred from electronic annotation///0006364 // rRNA process

[Nrf2-KO, Air, PBS]	[Nrf2-KO, Air, SFN]	RefSeq Transcript ID	p	FC PBS:SFN	Gene Symbol	Gene Title	Gene Ontology
1507.0249	1115.6831	NM_026027	0.002	-1.33	***Pfdn1***	prefoldin 1	0006457 // protein folding // inferred from electronic annotation///0021537 // telencephalon development // infe
333.24176	252.68587	NM_198622	0.007	-1.33	***H1fx***	H1 histone family, member X	0006334 // nucleosome assembly // inferred from electronic annotation
308.87	234.86922	NM_178683///XM_0	0.006	-1.33	***Depdc1b***	DEP domain containing 1B	0007165 // signal transduction // inferred from electronic annotation///0035556 // intracellular signal transductio
145.13907	115.28207	NM_019496	0.005	-1.33	***Ammecr1***	Alport syndrome, mental retardation, midface hypoplasia and elliptocytosis chromosomal region gene 1	
905.43353	674.23114	NM_001038592///NI	0.001	-1.33	***Glrx2***	glutaredoxin 2 (thioltransferase)	0006810 // transport // inferred from electronic annotation///0008152 // metabolic process // inferred from elect
123.114204	98.10153	NM_177752///XM_0	0.001	-1.34	***Eme1***	essential meiotic endonuclease 1 homolog 1 (S. pombe)	0006281 // DNA repair // inferred from electronic annotation///0006310 // DNA recombination // inferred from el
631.705	470.5964	NM_026389///XR_38	0.001	-1.34	***Poldip2***	polymerase (DNA-directed), delta interacting protein 2	0070584 // mitochondrion morphogenesis // inferred from mutant phenotype
385.67322	290.74564	NM_024438	0.001	-1.34	***Dusp19***	dual specificity phosphatase 19	0000188 // inactivation of MAPK activity // inferred from direct assay///0006469 // negative regulation of protein
2123.547	1552.8875	NM_175503	0.007	-1.34	***Aard***	alanine and arginine rich domain containing protein	0030324 // lung development // inferred from electronic annotation
280.47403	214.833	NM_080451///XM_0	0.008	-1.34	***Synpo2***	synaptopodin 2	0003779 // actin binding // not recorded///0005515 // protein binding // inferred from physical interaction///0051
125.17739	99.526985	NM_028001///XM_0	0.001	-1.34	***Jsrp1***	junctional sarcoplasmic reticulum protein 1	0003009 // skeletal muscle contraction // not recorded///0006941 // striated muscle contraction // inferred from
444.32578	333.25278	NM_001093759///NI	0.002	-1.34	***Trappc13***	trafficking protein particle complex 13	
80.734375	63.63642	NM_031183///XM_0	0.004	-1.34	***Gm11532///Sp6***	---///Mus musculus adult male testis cDNA, RIKEN full-length enriched library, clone	0006351 // transcription, DNA-templated // inferred from electronic annotation///0006355 // regulation of transc
1090.2358	799.1294	NM_026904///XM_0	0.001	-1.34	***Anapc10***	anaphase promoting complex subunit 10	0007049 // cell cycle // inferred from electronic annotation///0007067 // mitotic nuclear division // inferred from
2230.1128	1612.0186	NM_021896///XM_0	0.003	-1.34	***Gucy1a3***	guanylate cyclase 1, soluble, alpha 3	0006182 // cGMP biosynthetic process // inferred from mutant phenotype///0006182 // cGMP biosynthetic proce
77.53497	60.74349	NM_001159289///NI	0.007	-1.35	***Bcl11a***	B cell CLL/lymphoma 11A (zinc finger protein)	0006351 // transcription, DNA-templated // inferred from electronic annotation///0006355 // regulation of transc
374.49072	280.0011	NM_139291///XM_0	0.002	-1.35	***Cdc26***	cell division cycle 26	0007049 // cell cycle // inferred from electronic annotation///0007067 // mitotic nuclear division // inferred from
525.8893	388.74933	NM_146188///XM_0	0.002	-1.35	***Kctd15***	potassium channel tetramerisation domain containing 15	0007275 // multicellular organismal development // inferred from electronic annotation///0051260 // protein hon
52.727173	39.983593	NM_144809///XM_0	0.009	-1.35	***Prdm9***	PR domain containing 9	0006311 // meiotic gene conversion // not recorded///0006351 // transcription, DNA-templated // inferred from e
155.33177	121.84986	NM_008836///XR_03	0.007	-1.35	***Phxr5***	per-hexamer repeat gene 5	
22.407635	16.37769	NM_025732///XM_0	0.001	-1.36	***Ankrd61***	ankyrin repeat domain 61	0003677 // DNA binding // inferred from electronic annotation///0005515 // protein binding // inferred from elect
34.08868	25.10627	NM_001163691///NI	0.001	-1.36	***Cacna1h***	calcium channel, voltage-dependent, T type, alpha 1H subunit	0006810 // transport // inferred from electronic annotation///0006811 // ion transport // inferred from electronic
121.592545	94.95176	NM_146041///XM_0	0.002	-1.36	***Gmds***	GDP-mannose 4, 6-dehydratase	0007219 // Notch signaling pathway // inferred from sequence or structural similarity///0019673 // GDP-mannose
1800.4291	1306.4193	NM_023223	0.006	-1.36	***Cdc20***	cell division cycle 20	0007049 // cell cycle // inferred from electronic annotation///0007067 // mitotic nuclear division // inferred from
110.34057	85.95164	NM_001122822///NI	0.002	-1.36	***Wrn***	Werner syndrome homolog (human)	0000723 // telomere maintenance // inferred from genetic interaction///0000723 // telomere maintenance // infe
475.57684	349.3024	NM_001033132///NI	0.009	-1.36	***Commd6***	COMM domain containing 6	0032088 // negative regulation of NF-kappaB transcription factor activity // not recorded
768.7931	558.8233	NM_025569///NM_0	0.006	-1.36	***Mgst3***	microsomal glutathione S-transferase 3	0008152 // metabolic process // ---///0008152 // metabolic process // inferred from electronic annotation///0055
1736.8685	1260.1621	NM_025387///XM_0	0.005	-1.36	***Tmem14c***	transmembrane protein 14C	0006783 // heme biosynthetic process // inferred from electronic annotation
330.44254	246.32713	NM_009791///XM_0	0.008	-1.36	***Aspm***	asp (abnormal spindle)-like, microcephaly associated (Drosophila)	0001764 // neuron migration // inferred from genetic interaction///0001764 // neuron migration // inferred from
426.0496	315.11758	NM_001111025///NI	0.001	-1.36	***Pigx***	phosphatidylinositol glycan anchor biosynthesis, class X	0006506 // GPI anchor biosynthetic process // inferred from electronic annotation
245.00476	184.7495	NM_010255///XM_0	0.005	-1.36	***Gamt***	guanidinoacetate methyltransferase	0006601 // creatine biosynthetic process // inferred from mutant phenotype///0006601 // creatine biosynthetic p
50.294907	37.466606	NM_008023	0.001	-1.37	***Foxb2***	forkhead box B2	0000122 // negative regulation of transcription from RNA polymerase II promoter // not recorded///0006351 // tr
166.62358	129.84332	NM_008001///XM_0	0.008	-1.37	***Fgd1***	FYVE, RhoGEF and PH domain containing 1	0007010 // cytoskeleton organization // inferred from direct assay///0008360 // regulation of cell shape // inferre
439.23337	322.7223	NM_053159	0.006	-1.37	***Mrpl3***	mitochondrial ribosomal protein L3	0006412 // translation // not recorded
386.2567	284.17725	NM_001190448///NI	0.007	-1.37	***Ddc***	dopa decarboxylase	0006520 // cellular amino acid metabolic process // inferred from electronic annotation///0007623 // circadian rh
1970.7715	1402.2107	NM_026065///XM_0	0.003	-1.37	***Mrpl42***	mitochondrial ribosomal protein L42	0044822 // poly(A) RNA binding // not recorded
225.99126	170.91162	NM_011851	0.009	-1.37	***Nt5e***	5' nucleotidase, ecto	0006164 // purine nucleotide biosynthetic process // not recorded///0006196 // AMP catabolic process // inferred
537.7537	390.30692	NM_001195338///NI	0.002	-1.37	***Bbip1***	BBSome interacting protein 1	0042384 // cilium assembly // not recorded///0042755 // eating behavior // inferred from mutant phenotype///00
111.102486	85.97542	NM_178793///XM_0	0.002	-1.37	***Ccbe1***	collagen and calcium binding EGF domains 1	0001525 // angiogenesis // inferred from electronic annotation///0001946 // lymphangiogenesis // inferred from
569.5305	412.53387	NM_011917///XM_0	0.001	-1.37	***Xrn2***	5'-3' exoribonuclease 2	0000738 // DNA catabolic process, exonucleolytic // not recorded///0006139 // nucleobase-containing compound
327.6235	241.07658	NM_028133	0.002	-1.38	***Egln3***	egl-9 family hypoxia-inducible factor 3	0001666 // response to hypoxia // not recorded///0006915 // apoptotic process // inferred from electronic annota
161.39618	124.33198	NM_001164573///NI	0.008	-1.38	***Myo1h***	myosin 1H	0008152 // metabolic process // inferred from electronic annotation
127.47597	98.30433	NM_011845///XM_0	0.004	-1.38	***Mid2***	midline 2	0016567 // protein ubiquitination // inferred from electronic annotation///0032897 // negative regulation of viral
96.04039	73.03002	NM_029942	0.010	-1.38	***Prelid2***	PRELI domain containing 2	0015914 // phospholipid transport // not recorded
103.34752	78.79734	NM_001081407///NI	0.007	-1.38	***Plb1***	phospholipase B1	0006629 // lipid metabolic process // inferred from electronic annotation///0006644 // phospholipid metabolic pr
416.43024	302.74265	NM_175432	0.000	-1.39	***Tmem132c***	transmembrane protein 132C	0010923 // negative regulation of phosphatase activity // inferred from sequence or structural similarity
264.32852	196.49113	NM_008137///XM_0	0.002	-1.39	***Gna14***	guanine nucleotide binding protein, alpha 14	0006184 // GTP catabolic process // not recorded///0007165 // signal transduction // inferred from electronic ann
331.20117	241.22412	NM_025449	0.001	-1.39	***Nicn1***	nicolin 1	0005634 // nucleus // inferred from electronic annotation///0005874 // microtubule // inferred from electronic an
99.15262	75.449936	NM_027010	0.001	-1.39	***Crygf***	crystallin, gamma F	0001654 // eye development // inferred from mutant phenotype
777.90686	554.2607	NM_020026///XM_0	0.002	-1.39	***B3galnt1***	UDP-GalNAc:betaGlcNAc beta 1,3-galactosaminyltransferase, polypeptide 1	0006486 // protein glycosylation // inferred from electronic annotation///0009312 // oligosaccharide biosynthetic
269.31265	199.10187	NM_016662///XR_38	0.004	-1.39	***Mxd3***	Max dimerization protein 3	0006351 // transcription, DNA-templated // inferred from electronic annotation///0006355 // regulation of transc
145.31427	110.45515	NM_001145949///NI	0.005	-1.39	***Dlgap5***	discs, large (Drosophila) homolog-associated protein 5	0000087 // mitotic M phase // not recorded///0007049 // cell cycle // inferred from electronic annotation///0007
415.23203	301.2986	NM_025566	0.004	-1.39	***Tnfaip8l1***	tumor necrosis factor, alpha-induced protein 8-like 1	
68.68397	51.35937	NM_001142792///NI	0.003	-1.39	***Tmem150b***	transmembrane protein 150B	0016020 // membrane // inferred from electronic annotation///0016021 // integral component of membrane // in
32.962685	23.446203	NM_007500	0.005	-1.39	***Atoh1***	atonal homolog 1 (Drosophila)	0001764 // neuron migration // inferred from mutant phenotype///0006351 // transcription, DNA-templated // in
1513.469	1063.2493	NM_001163522///NI	0.006	-1.40	***Emcn***	endomucin	0001525 // angiogenesis // inferred from sequence or structural similarity///0016337 // single organismal cell-cell
155.33069	117.66943	NM_001013778///XI	0.003	-1.40	***Tvp23a***	trans-golgi network vesicle protein 23A	0016021 // integral component of membrane // inferred from electronic annotation
2260.7068	1571.7618	NM_027395	0.002	-1.40	***Basp1***	brain abundant, membrane attached signal protein 1	0045892 // negative regulation of transcription, DNA-templated // not recorded///0045892 // negative regulation
415.6346	299.18332	NM_001135172///NI	0.008	-1.40	***C1qtnf7***	C1q and tumor necrosis factor related protein 7	0051260 // protein homooligomerization // inferred from direct assay
689.79517	487.75818	NM_175429///XM_0	0.003	-1.40	***Kctd12b***	potassium channel tetramerisation domain containing 12b	0006810 // transport // inferred from electronic annotation///0006811 // ion transport // inferred from electronic
214.93875	160.29922	NM_029798	0.010	-1.40	***Flywch2***	FLYWCH family member 2	0044822 // poly(A) RNA binding // not recorded
594.3807	420.10086	NM_009848///XM_0	0.005	-1.40	***Entpd1***	ectonucleoside triphosphate diphosphohydrolase 1	0006200 // ATP catabolic process // inferred from direct assay///0007186 // G-protein coupled receptor signaling
216.45532	161.25957	NM_001110162///NI	0.001	-1.40	***Cdca2***	cell division cycle associated 2	0007049 // cell cycle // inferred from electronic annotation///0007059 // chromosome segregation // inferred fro
391.05972	281.36353	NM_172911	0.005	-1.40	***D8Ertd82e***	DNA segment, Chr 8, ERATO Doi 82, expressed	0006468 // protein phosphorylation // inferred from electronic annotation///0016310 // phosphorylation // infer
514.2754	366.67722	NM_031863///XM_0	0.007	-1.40	***Cenpq***	centromere protein Q	0000775 // chromosome, centromeric region // inferred from electronic annotation///0005634 // nucleus // not re
123.98846	93.721504	NM_008550///XM_0	0.007	-1.40	***Man2b2***	mannosidase 2, alpha B2	0005975 // carbohydrate metabolic process // inferred from electronic annotation///0006013 // mannose metabo
154.26088	116.62781	NM_019517///XM_0	0.001	-1.40	***Bace2***	beta-site APP-cleaving enzyme 2	0006508 // proteolysis // inferred from electronic annotation///0006509 // membrane protein ectodomain proteo
153.0885	115.90007	NM_029102///XM_0	0.003	-1.41	***Glt8d2***	glycosyltransferase 8 domain containing 2	0008152 // metabolic process // inferred from electronic annotation
390.39645	280.12698	NM_001290660///NI	0.003	-1.41	***Cd302***	CD302 antigen	0006909 // phagocytosis // not recorded
953.11487	669.10876	NM_001285833///NI	0.006	-1.41	***Nox4***	NADPH oxidase 4	0000902 // cell morphogenesis // inferred from mutant phenotype///0001666 // response to hypoxia // inferred f
168.86012	126.63981	NM_025864	0.003	-1.41	***Tmem206***	transmembrane protein 206	0016020 // membrane // inferred from electronic annotation///0016021 // integral component of membrane // in

[Nrf2-KO, Air, PBS]	[Nrf2-KO, Air, SFN]	RefSeq Transcript ID	p	FC PBS:SFN	Gene Symbol	Gene Title	Gene Ontology
1962.9341	1367.4845	NM_001012335///N	0.003	-1.41	***Mdk***	midkine	0001662 // behavioral fear response // inferred from mutant phenotype///0007275 // multicellular organismal de
470.00073	332.3818	NM_024273///XM_0	0.001	-1.42	***Timmdc1***	translocase of inner mitochondrial membrane domain containing 1	0005634 // nucleus // not recorded///0005739 // mitochondrion // inferred from direct assay///0005739 // mitoc
385.82672	275.2029	NM_030206	0.007	-1.42	***Cygb***	cytoglobin	0006810 // transport // inferred from electronic annotation///0006979 // response to oxidative stress // not recor
20.431938	14.452564	NM_178797///XM_0	0.009	-1.42	***Far2***	fatty acyl CoA reductase 2	0006629 // lipid metabolic process // inferred from electronic annotation///0006694 // steroid biosynthetic proce
238.75812	174.8036	NM_029532///XM_0	0.002	-1.42	***Snrnp35***	small nuclear ribonucleoprotein 35 (U11/U12)	0006397 // mRNA processing // inferred from electronic annotation///0008380 // RNA splicing // inferred from ele
572.60443	402.72873	NM_026282///XR_37	0.006	-1.42	***Spc24***	SPC24, NDC80 kinetochore complex component, homolog (S. cerevisiae)	0007049 // cell cycle // inferred from electronic annotation///0007067 // mitotic nuclear division // inferred from
75.08952	55.27078	NM_029741///XM_0	0.001	-1.42	***Ppfia3***	protein tyrosine phosphatase, receptor type, f polypeptide (PTPRF), interacting prot	0007269 // neurotransmitter secretion // not recorded///0048172 // regulation of short-term neuronal synaptic pl
66.133415	47.96016	NM_001040695///N	0.003	-1.42	***Uevld***	UEV and lactate/malate dehyrogenase domains	0005975 // carbohydrate metabolic process // inferred from electronic annotation///0006464 // cellular protein m
171.16832	127.0191	NM_001081328	0.001	-1.42	***Chsy3***	chondroitin sulfate synthase 3	0008152 // metabolic process // inferred from electronic annotation
333.6103	236.88177	NM_001161618///N	0.000	-1.42	***Cul5***	cullin 5	0006511 // ubiquitin-dependent protein catabolic process // inferred from electronic annotation///0006970 // res
738.95154	513.9807	NM_173047	0.001	-1.42	***Cbr3***	carbonyl reductase 3	0008152 // metabolic process // inferred from electronic annotation///0042376 // phylloquinone catabolic proces
220.60121	161.55362	NM_025962	0.010	-1.43	***Mmachc***	methylmalonic aciduria cblC type, with homocystinuria	0009236 // cobalamin biosynthetic process // inferred from electronic annotation
151.97545	112.48833	NM_152817///XM_0	0.001	-1.43	***Ttc27***	tetratricopeptide repeat domain 27	0005515 // protein binding // inferred from electronic annotation
301.6617	216.92056	NM_001290430///N	0.005	-1.43	***Ftsj1***	FtsJ homolog 1 (E. coli)	0001510 // RNA methylation // inferred from electronic annotation///0002128 // tRNA nucleoside ribose methylat
147.6291	108.6102	NM_026992///NR_03	0.008	-1.43	***Dnajc24***	DnaJ (Hsp40) homolog, subfamily C, member 24	0006810 // transport // inferred from electronic annotation///0017183 // peptidyl-diphthamide biosynthetic proce
1268.8545	873.6018	NM_007428	0.002	-1.43	***Agt***	angiotensinogen (serpin peptidase inhibitor, clade A, member 8)	0001543 // ovarian follicle rupture // inferred from mutant phenotype///0001568 // blood vessel development // i
279.01852	199.68459	NM_007467	0.004	-1.43	***Aplp1***	amyloid beta (A4) precursor-like protein 1	0006378 // mRNA polyadenylation // inferred from direct assay///0006417 // regulation of translation // inferred f
360.3249	252.59795	NM_001079847///N	0.008	-1.43	***Gpr64***	G protein-coupled receptor 64	0007165 // signal transduction // inferred from electronic annotation///0007166 // cell surface receptor signaling
107.3546	79.08241	NM_007426	0.001	-1.44	***Angpt2***	angiopoietin 2	0001525 // angiogenesis // inferred from genetic interaction///0001666 // response to hypoxia // inferred from el
113.00433	83.67344	NM_177878	0.002	-1.44	***Mblac1***	metallo-beta-lactamase domain containing 1	0008152 // metabolic process // inferred from electronic annotation
5645.628	3857.808	NM_053247	0.009	-1.44	***Lyve1***	lymphatic vessel endothelial hyaluronan receptor 1	0006027 // glycosaminoglycan catabolic process // inferred from direct assay///0006810 // transport // inferred fr
121.45598	89.89753	NM_001012324	0.001	-1.44	***Ecm2***	extracellular matrix protein 2, female organ and adipocyte specific	0010811 // positive regulation of cell-substrate adhesion // inferred from direct assay///0030198 // extracellular m
891.39557	612.89484	NM_053267	0.004	-1.44	***Selm***	selenoprotein M	0010269 // response to selenium ion // inferred from mutant phenotype///0035264 // multicellular organism grov
555.9156	384.99414	NM_026526///XM_0	0.001	-1.44	***N6amt2***	N-6 adenine-specific DNA methyltransferase 2 (putative)	0032259 // methylation // inferred from electronic annotation
250.12187	179.43602	NM_001077713	0.007	-1.44	***Acn9***	ACN9 homolog (S. cerevisiae)	0005739 // mitochondrion // inferred from electronic annotation///0005758 // mitochondrial intermembrane spa
237.71887	171.39177	NM_178149///XM_0	0.002	-1.44	***Pik3ip1***	phosphoinositide-3-kinase interacting protein 1	0014067 // negative regulation of phosphatidylinositol 3-kinase signaling // inferred from direct assay///0014067 /
63.661663	45.763233	NM_008764	0.005	-1.44	***Tnfrsf11b***	tumor necrosis factor receptor superfamily, member 11b (osteoprotegerin)	0006915 // apoptotic process // inferred from electronic annotation///0007165 // signal transduction // inferred fr
339.59152	239.31725	NM_001044741///N	0.004	-1.44	***Cacnb3***	calcium channel, voltage-dependent, beta 3 subunit	0006810 // transport // inferred from electronic annotation///0006811 // ion transport // inferred from electronic
268.11597	190.65865	NM_021715	0.002	-1.45	***Chst7***	carbohydrate (N-acetylglucosamino) sulfotransferase 7	0005975 // carbohydrate metabolic process // inferred from electronic annotation///0006044 // N-acetylglucosam
164.04744	120.011406	NM_001168318///N	0.007	-1.45	***Scara5***	scavenger receptor class A, member 5 (putative)	0006810 // transport // inferred from electronic annotation///0006811 // ion transport // inferred from electronic
687.8254	473.63486	NM_053134	0.004	-1.45	***Pcdhb9***	protocadherin beta 9	0007155 // cell adhesion // inferred from electronic annotation///0007156 // homophilic cell adhesion // inferred
1108.9451	758.71906	NM_054042	0.003	-1.45	***Cd248***	CD248 antigen, endosialin	0008284 // positive regulation of cell proliferation // inferred from mutant phenotype///0016477 // cell migration
137.27562	100.30771	NM_011427	0.007	-1.45	***Snai1***	snail family zinc finger 1	0000122 // negative regulation of transcription from RNA polymerase II promoter // inferred from direct assay///0
68.82652	49.30296	NM_001166645	0.001	-1.45	***Zfp882***	zinc finger protein 882	0006355 // regulation of transcription, DNA-templated // inferred from electronic annotation
81.829	59.284855	NM_001035509///N	0.000	-1.45	***Zcchc18***	zinc finger, CCHC domain containing 18	0030509 // BMP signaling pathway // not recorded
185.50455	135.20992	NM_019438///XR_37	0.007	-1.45	***Ncapg***	non-SMC condensin I complex, subunit G	0007076 // mitotic chromosome condensation // not recorded
690.9488	471.4112	NM_008005///NR_1	0.000	-1.45	***Fgf18***	fibroblast growth factor 18	0001503 // ossification // inferred from mutant phenotype///0001525 // angiogenesis // inferred from mutant phe
265.34882	188.1758	NM_001033409///X	0.002	-1.46	***Lgr6***	leucine-rich repeat-containing G protein-coupled receptor 6	0007165 // signal transduction // inferred from electronic annotation///0007186 // G-protein coupled receptor sig
483.5801	331.87967	NM_008289	0.003	-1.46	***Hsd11b2***	hydroxysteroid 11-beta dehydrogenase 2	0001666 // response to hypoxia // inferred from electronic annotation///0002017 // regulation of blood volume b
23.81685	16.124252	NM_178678	0.009	-1.46	***Lrrtm3***	leucine rich repeat transmembrane neuronal 3	1902004 // positive regulation of beta-amyloid formation // inferred from mutant phenotype///1902004 // positiv
342.65283	238.53189	NM_009773///XM_0	0.008	-1.46	***Bub1b***	budding uninhibited by benzimidazoles 1 homolog, beta (S. cerevisiae)	0006468 // protein phosphorylation // inferred from electronic annotation///0006915 // apoptotic process // infer
78.83165	56.429066	NM_001081224///X	0.004	-1.46	***Prr16***	proline rich 16	
283.43787	199.3061	NM_024245///XM_0	0.005	-1.46	***Kif23***	kinesin family member 23	0000910 // cytokinesis // not recorded///0007018 // microtubule-based movement // inferred from electronic an
63.59395	44.91379	NM_001166631///N	0.001	-1.47	***Havcr1***	hepatitis A virus cellular receptor 1	0033005 // positive regulation of mast cell activation // inferred from direct assay
272.941	191.12143	NM_029790///XM_0	0.004	-1.47	***Mettl15***	methyltransferase like 15	0032259 // methylation // inferred from electronic annotation
109.840904	79.17547	NR_028264	0.000	-1.47	***Dleu2***	deleted in lymphocytic leukemia, 2	0010977 // negative regulation of neuron projection development // inferred from genetic interaction///0014043
69.909775	49.55919	NM_010692	0.000	-1.47	***Lbx2***	ladybird homeobox homolog 2 (Drosophila)	0006351 // transcription, DNA-templated // inferred from electronic annotation///0006355 // regulation of transc
1923.5459	1284.3878	NM_001013753///X	0.009	-1.47	***Pcdh17***	protocadherin 17	0007155 // cell adhesion // inferred from electronic annotation///0007156 // homophilic cell adhesion // inferred
127.83292	92.35753	NM_019950	0.001	-1.48	***Chst5***	carbohydrate (N-acetylglucosamine 6-O) sulfotransferase 5	0005975 // carbohydrate metabolic process // inferred from electronic annotation///0006044 // N-acetylglucosam
125.35397	89.58526	NM_182783	0.003	-1.48	***Fam167b***	family with sequence similarity 167, member B	
178.85297	128.3001	NM_001199293///N	0.001	-1.48	***Tex14***	testis expressed gene 14	0006468 // protein phosphorylation // inferred from electronic annotation///0007049 // cell cycle // inferred from
771.8504	513.0981	NM_008939	0.002	-1.48	***Prss12***	protease, serine 12 neurotrypsin (motopsin)	0006508 // proteolysis // inferred from direct assay///0006508 // proteolysis // inferred from mutant phenotype//
348.84506	239.82242	NM_021525///XM_0	0.010	-1.49	***Rcl1***	RNA terminal phosphate cyclase-like 1	0006364 // rRNA processing // not recorded///0006396 // RNA processing // inferred from electronic annotation//
226.09624	158.50426	NM_001143683///N	0.001	-1.49	***Mpped2***	metallophosphoesterase domain containing 2	0008152 // metabolic process // inferred from electronic annotation
208.80586	146.52118	NM_001081114///X	0.001	-1.50	***Clip3***	CAP-GLY domain containing linker protein 3	0001934 // positive regulation of protein phosphorylation // inferred from direct assay///0010828 // positive regul
248.34535	172.27098	NM_001168475///N	0.008	-1.50	***Ttc23***	tetratricopeptide repeat domain 23	0005515 // protein binding // inferred from electronic annotation
101.84433	71.86929	NM_172951///XM_0	0.009	-1.50	***Sntg2***	syntrophin, gamma 2	0003779 // actin binding // inferred from electronic annotation///0005515 // protein binding // inferred from elec
554.669	366.9357	NM_178753	0.000	-1.50	***Spin4***	spindlin family, member 4	0007276 // gamete generation // inferred from electronic annotation
94.122795	65.68825	NM_001271757///N	0.000	-1.51	***Wnt5b***	wingless-type MMTV integration site family, member 5B	0002062 // chondrocyte differentiation // inferred from electronic annotation///0007165 // signal transduction //
278.63016	191.40471	NM_001289487///N	0.005	-1.51	***Exoc3l4***	exocyst complex component 3-like 4	0006887 // exocytosis // inferred from electronic annotation
265.62103	181.28967	NM_009364///XM_0	0.004	-1.52	***Tfpi2***	tissue factor pathway inhibitor 2	0007596 // blood coagulation // inferred from electronic annotation///0007599 // hemostasis // inferred from ele
1151.1533	758.62775	NM_010423///XM_0	0.009	-1.52	***Hey1***	hairy/enhancer-of-split related with YRPW motif 1	0000122 // negative regulation of transcription from RNA polymerase II promoter // inferred from direct assay///0
214.86055	148.62045	NM_001110512///N	0.001	-1.52	***Mettl8***	methyltransferase like 8	0007519 // skeletal muscle tissue development // inferred from direct assay///0008152 // metabolic process // inf
463.74387	306.95325	NM_001033331///N	0.010	-1.52	***Gas2l3***	growth arrest-specific 2 like 3	0000226 // microtubule cytoskeleton organization // not recorded///0007050 // cell cycle arrest // inferred from e
101.85328	70.21146	NM_001127233///N	0.005	-1.52	***Trp53***	transformation related protein 53	0000060 // protein import into nucleus, translocation // inferred from direct assay///0000122 // negative regulatio
93.869415	64.52021	NM_010460///NM_0	0.008	-1.52	***Hoxb7///Hoxb8***	homeobox B7///homeobox B8	0006351 // transcription, DNA-templated // inferred from electronic annotation///0006355 // regulation of transc
42.14293	27.84835	NM_001081651///X	0.005	-1.53	***Rab42***	RAB42, member RAS oncogene family	0007264 // small GTPase mediated signal transduction // inferred from electronic annotation///0015031 // protei
3557.841	2307.1895	NM_032398	0.000	-1.53	***Plvap***	plasmalemma vesicle associated protein	0000165 // MAPK cascade // not recorded///0002693 // positive regulation of cellular extravasation // inferred fro
2217.2612	1414.6968	NM_001008497///N	0.004	-1.53	***P2ry14***	purinergic receptor P2Y, G-protein coupled, 14	0006955 // immune response // not recorded///0007165 // signal transduction // inferred from electronic annota
133.75133	93.684616	NM_013811///XM_0	0.009	-1.53	***Dnah8***	dynein, axonemal, heavy chain 8	0006200 // ATP catabolic process // inferred from electronic annotation///0007018 // microtubule-based moveme
110.893295	76.06643	NM_009116///XM_0	0.005	-1.53	***Prrx2***	paired related homeobox 2	0002053 // positive regulation of mesenchymal cell proliferation // inferred from genetic interaction///0006355 //

[Nrf2-KO, Air, PBS]	[Nrf2-KO, Air, SFN]	RefSeq Transcript ID	p	FC PBS:SFN	Gene Symbol	Gene Title	Gene Ontology
152.97404	105.081436	NM_011270	0.000	-1.54	***Rhd***	Rh blood group, D antigen	0015696 // ammonium transport // inferred from mutant phenotype///0048821 // erythrocyte development // inf
226.04362	154.1083	NM_013552///XM_0	0.002	-1.54	***Hmmr***	hyaluronan mediated motility receptor (RHAMM)	0000160 // phosphorelay signal transduction system // inferred from electronic annotation
208.20135	142.04077	NM_011943///XM_0	0.006	-1.54	***Map2k6***	mitogen-activated protein kinase kinase 6	0000165 // MAPK cascade // not recorded///0000165 // MAPK cascade // inferred from physical interaction///000
235.67747	158.48277	NM_178890	0.007	-1.55	***Abtb2***	ankyrin repeat and BTB (POZ) domain containing 2	0097237 // cellular response to toxic substance // inferred from mutant phenotype
66.024605	43.979137	NM_001081257///XN	0.005	-1.56	***Hpse2***	heparanase 2	0005975 // carbohydrate metabolic process // inferred from electronic annotation
96.50908	65.044495	NM_001281859///NN	0.002	-1.56	***Cysltr1***	cysteinyl leukotriene receptor 1	0006816 // calcium ion transport // inferred from direct assay///0006935 // chemotaxis // inferred from direct ass
106.63459	72.27501	NM_001039554///XN	0.002	-1.56	***Angptl7***	angiopoietin-like 7	0005576 // extracellular region // inferred from electronic annotation
459.28854	296.07187	NM_001161782///NN	0.002	-1.57	***Trim32***	tripartite motif-containing 32	0000209 // protein polyubiquitination // not recorded///0009411 // response to UV // inferred from direct assay//
2627.7434	1645.6454	NM_026730///XM_0	0.003	-1.57	***Gpihbp1***	GPI-anchored HDL-binding protein 1	0006810 // transport // inferred from electronic annotation///0006869 // lipid transport // inferred from direct as
148.71043	100.89615	NM_008002	0.001	-1.57	***Fgf10***	fibroblast growth factor 10	0000132 // establishment of mitotic spindle orientation // inferred from genetic interaction///0000187 // activatio
1063.8348	666.0999	NM_026753	0.007	-1.59	***Fam96b***	family with sequence similarity 96, member B	0007059 // chromosome segregation // not recorded
554.43036	349.15582	NM_008872///XM_0	0.003	-1.59	***Plat***	plasminogen activator, tissue	0001666 // response to hypoxia // inferred from mutant phenotype///0006508 // proteolysis // inferred from dire
77.80748	51.41216	NM_010014///NM_1	0.001	-1.59	***Dab1***	disabled 1	0001764 // neuron migration // inferred from genetic interaction///0001764 // neuron migration // inferred from
908.5482	561.06116	NM_026125///XM_0	0.007	-1.60	***Fam132a***	family with sequence similarity 132, member A	0010906 // regulation of glucose metabolic process // inferred from direct assay///0035774 // positive regulation
65.46931	41.820446	NM_007606	0.001	-1.60	***Car3***	carbonic anhydrase 3	0006730 // one-carbon metabolic process // inferred from electronic annotation///0006979 // response to oxidati
2056.5046	1261.5541	NM_001111274///NN	0.004	-1.61	***Igf1***	insulin-like growth factor 1	0001649 // osteoblast differentiation // inferred from expression pattern///0001775 // cell activation // not recor
102.78103	66.57678	NM_030728///XM_0	0.005	-1.61	***Cemip***	cell migration inducing protein, hyaluronan binding	0008152 // metabolic process // inferred from electronic annotation///0010800 // positive regulation of peptidyl-t
331.3779	210.5923	NM_029007	0.001	-1.62	***Fam84a***	family with sequence similarity 84, member A	
168.87688	110.31698	NM_001101479///XN	0.001	-1.62	***Pabpc4l***	poly(A) binding protein, cytoplasmic 4-like	0000166 // nucleotide binding // inferred from electronic annotation///0003676 // nucleic acid binding // inferred
65.818535	41.88387	NM_001103367///NN	0.000	-1.62	***Rai2***	retinoic acid induced 2	0005515 // protein binding // inferred from electronic annotation
128.29865	82.591	NM_001159711///NN	0.001	-1.63	***Ly96***	lymphocyte antigen 96	0002221 // pattern recognition receptor signaling pathway // not recorded///0002376 // immune system process ,
118.47577	76.61016	NM_001009978///NN	0.001	-1.64	***Pde1a***	phosphodiesterase 1A, calmodulin-dependent	0006198 // cAMP catabolic process // not recorded///0007165 // signal transduction // inferred from electronic ar
80.8306	51.583954	NM_173425///XM_0	0.002	-1.64	***Fam124b***	family with sequence similarity 124, member B	
94.2049	59.792088	NM_011670	0.007	-1.64	***Uchl1***	ubiquitin carboxy-terminal hydrolase L1	0002931 // response to ischemia // inferred from mutant phenotype///0006508 // proteolysis // inferred from ele
953.23206	575.52185	NM_001111100///NN	0.006	-1.66	***Lipa***	lysosomal acid lipase A	0000902 // cell morphogenesis // inferred from mutant phenotype///0001816 // cytokine production // inferred fr
340.11542	207.1582	NM_009627	0.004	-1.67	***Adm***	adrenomedullin	0001570 // vasculogenesis // not recorded///0001666 // response to hypoxia // inferred from electronic annotatio
129.80984	81.81148	NM_009860///XM_0	0.005	-1.67	***Cdc25c***	cell division cycle 25C	0000086 // G2/M transition of mitotic cell cycle // not recorded///0000087 // mitotic M phase // inferred from ele
95.38972	57.462685	NM_001195084///NN	0.005	-1.72	***Plscr2***	phospholipid scramblase 2	0071222 // cellular response to lipopolysaccharide // inferred from expression pattern
337.92392	200.06259	NM_177157	0.006	-1.74	***Gchfr***	GTP cyclohydrolase I feedback regulator	0006461 // protein complex assembly // not recorded///0008152 // metabolic process // inferred from electronic
112.232086	67.218216	NM_018769///XM_0	0.001	-1.75	***Dfna5***	deafness, autosomal dominant 5 (human)	0006915 // apoptotic process // inferred from electronic annotation///0007605 // sensory perception of sound //
111.56142	66.486885	NM_001042653	0.007	-1.76	***Oip5***	Opa interacting protein 5	0007049 // cell cycle // inferred from electronic annotation///0007067 // mitotic nuclear division // inferred from
750.7436	417.03296	NM_008250	0.002	-1.79	***Hlx***	H2.0-like homeobox	0001889 // liver development // inferred from mutant phenotype///0006351 // transcription, DNA-templated // ir
107.776245	63.390053	NM_177161///XM_0	0.009	-1.80	***P4ha3***	procollagen-proline, 2-oxoglutarate 4-dioxygenase (proline 4-hydroxylase), alpha pc	0019511 // peptidyl-proline hydroxylation // not recorded///0055114 // oxidation-reduction process // not record
131.0536	76.33556	NM_177355	0.006	-1.84	***Plcxd3***	phosphatidylinositol-specific phospholipase C, X domain containing 3	0006629 // lipid metabolic process // inferred from electronic annotation///0007165 // signal transduction // infer
254.54904	140.26909	NM_008813///XM_0	0.000	-1.90	***Enpp1***	ectonucleotide pyrophosphatase/phosphodiesterase 1	0006091 // generation of precursor metabolites and energy // not recorded///0006200 // ATP catabolic process //
278.44623	152.16171	NM_028222///XM_0	0.001	-1.91	***Cdkn3***	cyclin-dependent kinase inhibitor 3	0006470 // protein dephosphorylation // inferred from electronic annotation///0007049 // cell cycle // inferred fro
166.40152	85.868385	NM_001039676///XN	0.009	-2.03	***Slc39a2***	solute carrier family 39 (zinc transporter), member 2	0006829 // zinc ion transport // inferred from direct assay///0030001 // metal ion transport // inferred from electr
111.33941	56.383278	NM_173414	0.000	-2.06	***Lancl3***	LanC lantibiotic synthetase component C-like 3 (bacterial)	0008152 // metabolic process // inferred from electronic annotation
111.55397	56.51285	NM_023612	0.001	-2.07	***Esm1***	endothelial cell-specific molecule 1	0001525 // angiogenesis // inferred from electronic annotation///0001558 // regulation of cell growth // inferred f
2052.631	939.8809	NM_008342///XM_0	0.003	-2.15	***Igfbp2***	insulin-like growth factor binding protein 2	0001558 // regulation of cell growth // inferred from electronic annotation///0006950 // response to stress // infe
140.03244	68.467514	NM_029847///XM_0	0.007	-2.18	***Arsk***	arylsulfatase K	0008152 // metabolic process // inferred from electronic annotation
224.33	108.98248	NM_173422	0.003	-2.19	***Colec10***	collectin sub-family member 10	0006952 // defense response // non-traceable author statement///0007157 // heterophilic cell-cell adhesion // no
288.8033	136.99895	NM_144944	0.002	-2.22	***Prokr2***	prokineticin receptor 2	0007165 // signal transduction // inferred from electronic annotation///0007186 // G-protein coupled receptor sig
53.607018	21.961576	NM_172829///XM_0	0.005	-2.44	***St6gal2***	beta galactoside alpha 2,6 sialyltransferase 2	0005975 // carbohydrate metabolic process // inferred from electronic annotation///0006054 // N-acetylneuramir
157.1751	66.76568	NM_028176///XM_0	0.001	-2.48	***Cda***	cytidine deaminase	0009972 // cytidine deamination // not recorded///0030308 // negative regulation of cell growth // not recorded/,

Table S3. Prenatal sulforaphane-neonatal hyperoxia interaction 258 genes in *Nrf2*$^{+/+}$ mice (2-Way ANOVA, p<=0.01)

RIKEN cDNA and predicted genes excluded. Fold Change (FC): Blue (-) - fold lower in SFN:O2 than PBS:O2, Red - fold higher in SFN:O2 than PBS:O2.

[ICR, Air, PBS]	[ICR, Air, SFN	[ICR, O2, PBS](	[ICR, O2, SFN](	RefSeq Transcript ID	p (Prenatal Treatment-Exposure)	FC [Air-PBS: O2-PBS	FC [O2 PBS:SFN]	Gene Symbol	Gene Title	Gene Ontology
69.962	72.55377	31.495602	73.17308	NM_020265///XM_0065	0.005	-2.22	2.32	***Dkk2***	dickkopf homolog 2 (Xenopus laevis)	0002376 // immune system process // inferred from electronic annotation///0006810 // transpor
1435.3619	1514.5039	329.68805	624.2319	NM_178738///XM_0065	0.002	-4.35	1.89	***Prss35***	protease, serine 35	0001889 // liver development // inferred from electronic annotation///0006725 // cellular aroma
1782.2644	1798.7755	1049.2744	1986.1266	NM_001198811///NM_1	0.008	-1.70	1.89	***Frem1***	Fras1 related extracellular matrix protein 1	0007155 // cell adhesion // inferred from electronic annotation///0031016 // pancreas developm
343.08368	283.36444	147.0619	273.31763	NM_019662///XM_0065	0.004	-2.33	1.86	***Rrad***	Ras-related associated with diabetes	0006629 // lipid metabolic process // inferred from electronic annotation///0016042 // lipid catab
12.493289	13.344014	12.067748	21.504776	NM_173427///NR_0355	0.009	-1.04	1.78	***Klhdc7a///Mir2139***	kelch domain containing 7A///microRNA 2139	0008152 // metabolic process // inferred from electronic annotation///0016310 // phosphorylatio
2220.1902	1995.4437	1351.5151	2271.5432	NM_001163577///NM_C	0.007	-1.64	1.68	***Prom1***	prominin 1	0000122 // negative regulation of transcription from RNA polymerase II promoter // inferred from
289.77322	328.9068	393.5178	649.8168	NM_001081322///XM_0	0.002	1.36	1.65	***Myo5c***	myosin VC	0006468 // protein phosphorylation // not recorded///0006811 // ion transport // inferred from
14.985249	13.548375	11.287953	18.416538	NR_045273	0.007	-1.33	1.63	***Med9os***	mediator complex subunit 9, opposite strand	0034220 // ion transmembrane transport // not recorded
182.24828	164.31073	124.71969	200.49524	NM_178908	0.007	-1.46	1.61	***Fam26e***	family with sequence similarity 26, member E	0001656 // metanephros development // inferred from sequence or structural similarity///00016
153.06573	143.52809	98.77891	149.83705	NM_181815///XM_0065	0.008	-1.55	1.52	***Cep128***	centrosomal protein 128	0009887 // organ morphogenesis // inferred from mutant phenotype///0018149 // peptide cross-
285.66928	308.43195	296.21262	443.75516	NM_029022///XM_0065	0.003	1.04	1.50	***Scrn3***	secernin 3	0007155 // cell adhesion // inferred from electronic annotation///0007156 // homophilic cell adh
38.758457	32.76064	39.00012	57.417824	NM_011136	0.004	1.01	1.47	***Pou2af1***	POU domain, class 2, associating factor 1	0001503 // ossification // not recorded///0006874 // cellular calcium ion homeostasis // traceabl
414.32886	354.26688	255.0949	375.13043	NM_001146031///NM_1	0.002	-1.62	1.47	***Nrcam***	neuron-glia-CAM-related cell adhesion molecule	0005576 // extracellular region // inferred from electronic annotation///0005737 // cytoplasm //
212.25095	213.30676	175.44528	257.22845	NM_194269///XM_0065	0.009	-1.21	1.47	***Morn2***	MORN repeat containing 2	0042552 // myelination // not recorded///1902043 // positive regulation of extrinsic apoptotic sig
21.415405	18.123074	23.46498	34.23301	NM_001013756///XM_0	0.006	1.10	1.46	***Grhl3***	grainyhead-like 3 (Drosophila)	0044237 // cellular metabolic process // inferred from electronic annotation///0055114 // oxidati
744.06445	696.336	373.4801	542.73157	NM_023245	0.001	-1.99	1.45	***Palmd***	palmdelphin	
274.52957	272.0314	272.9089	388.82977	NM_008884///NM_178C	0.000	-1.01	1.42	***Pml***	promyelocytic leukemia	0006810 // transport // inferred from electronic annotation///0006811 // ion transport // inferre
11671.783	11961.448	8171.2534	11585.314	NM_019759	0.006	-1.43	1.42	***Dpt***	dermatopontin	0006352 // DNA-templated transcription, initiation // inferred from electronic annotation///0006
2468.152	2428.5479	1506.5468	2106.177	NM_054041///XM_0065	0.008	-1.64	1.40	***Antxr1***	anthrax toxin receptor 1	0008088 // axon cargo transport // inferred from mutant phenotype///0008152 // metabolic proc
1153.7617	1322.3784	1018.63007	1423.494	NM_007984	0.002	-1.13	1.40	***Fscn1***	fascin homolog 1, actin bundling protein (Strongylocentrotus purpuratus)	0008152 // metabolic process // inferred from electronic annotation///0032254 // establishment
235.98412	232.56755	241.1119	329.556	NM_001163464///NM_C	0.009	1.02	1.37	***Abi3***	ABI gene family, member 3	0006417 // regulation of translation // inferred from electronic annotation///0016070 // RNA me
313.80157	295.3039	382.37112	522.4244	NM_133234///XM_0065	0.007	1.22	1.37	***Bbc3***	BCL2 binding component 3	0006355 // regulation of transcription, DNA-templated // not recorded
557.9463	540.34314	537.37665	730.9747	NM_009315///XM_0065	0.002	-1.04	1.36	***Taf6***	TAF6 RNA polymerase II, TATA box binding protein (TBP)-associated factor	0002238 // response to molecule of fungal origin // inferred from mutant phenotype///0002376 /
353.16513	395.15637	369.247	495.5319	NM_017391	0.008	1.05	1.34	***Slc5a3***	solute carrier family 5 (inositol transporters), member 3	0001934 // positive regulation of protein phosphorylation // not recorded///0002376 // immune
97.9636	104.04598	68.37857	90.27526	NM_031998///XM_0065	0.009	-1.43	1.32	***Cep41***	centrosomal protein 41	0006351 // transcription, DNA-templated // inferred from electronic annotation///0006355 // reg
276.13083	270.76517	172.2666	226.55972	NM_001081243///XM_0	0.000	-1.60	1.32	***Filip1***	filamin A interacting protein 1	0006355 // regulation of transcription, DNA-templated // not recorded
158.55287	172.46252	80.823364	106.18038	NM_001110162///NM_1	0.006	-1.96	1.31	***Cdca2***	cell division cycle associated 2	0005739 // mitochondrion // inferred from direct assay///0016020 // membrane // inferred from
147.0618	163.17853	165.08623	216.28656	NM_199449	0.003	1.12	1.31	***Zhx2***	zinc fingers and homeoboxes 2	0006412 // translation // not recorded
615.59863	645.599	511.9526	669.8806	NM_009288///XM_0065	0.005	-1.20	1.31	***Stk10***	serine/threonine kinase 10	0006310 // DNA recombination // inferred from electronic annotation///0006810 // transport // i
310.10034	306.23703	250.48381	323.60477	NM_001290701///NM_C	0.008	-1.24	1.29	***Meaf6***	MYST/Esa1-associated factor 6	0001836 // release of cytochrome c from mitochondria // not recorded///0001836 // release of c
48.34705	41.120743	37.52458	47.858074	NM_144786///XM_0064	0.006	-1.29	1.28	***Ggt7***	gamma-glutamyltransferase 7	0005515 // protein binding // inferred from physical interaction///0008022 // protein C-terminus
4880.149	4793.979	4049.3477	5153.279	NM_001170981///NM_C	0.000	-1.21	1.27	***Hnrnpc***	heterogeneous nuclear ribonucleoprotein C	0006928 // cellular component movement // not recorded///0007155 // cell adhesion // not reco
19.93411	17.67726	15.712506	19.89045	NM_025420///XM_0065	0.001	-1.27	1.27	***Lce1m***	late cornified envelope 1M	0016567 // protein ubiquitination // inferred from electronic annotation
309.1059	247.5186	313.42593	396.6182	NM_153143	0.006	1.01	1.27	***Kctd11***	potassium channel tetramerisation domain containing 11	0007010 // cytoskeleton organization // inferred from electronic annotation///0051693 // actin fi
1425.3546	1387.6141	1091.5663	1372.098	NM_177715///NR_0395	0.006	-1.31	1.26	***Kctd12///Mir5130***	potassium channel tetramerisation domain containing 12///microRNA 5130	0000054 // ribosomal subunit export from nucleus // inferred from electronic annotation///0006
2518.802	2448.97	1718.7509	2158.0605	NM_009450	0.002	-1.47	1.26	***Tubb2a***	tubulin, beta 2A class IIA	0007165 // signal transduction // inferred from electronic annotation///0007186 // G-protein cou
730.66693	715.09106	752.08075	943.2594	NM_001130149///NM_C	0.002	1.03	1.25	***Drosha***	drosha, ribonuclease type III	0000226 // microtubule cytoskeleton organization // not recorded///0010923 // negative regulati
252.06018	245.30936	235.94566	291.79364	NM_026641///XM_0065	0.007	-1.07	1.24	***Ift80***	intraflagellar transport 80	0006810 // transport // inferred from electronic annotation///0006817 // phosphate ion transpor
240.73627	227.8107	198.6216	243.10745	NM_001165951///NM_1	0.010	-1.21	1.22	***Vangl1***	vang-like 1 (van gogh, Drosophila)	0016020 // membrane // inferred from electronic annotation///0016021 // integral component o
467.52026	421.4644	506.38968	619.50287	NM_009029///XM_0065	0.010	1.08	1.22	***Rb1***	retinoblastoma 1	0006412 // translation // inferred from electronic annotation///0032543 // mitochondrial translat
389.43506	343.54913	246.42226	300.32742	NM_177906///XM_0065	0.001	-1.58	1.22	***Opcml***	opioid binding protein/cell adhesion molecule-like	0016020 // membrane // inferred from electronic annotation///0016021 // integral component o
1617.7766	1402.6871	563.3106	686.4046	NM_023223	0.004	-2.87	1.22	***Cdc20***	cell division cycle 20	0006591 // ornithine metabolic process // inferred from direct assay///0006596 // polyamine bio
250.1217	239.53566	204.21277	246.47789	NM_029790///XM_0065	0.006	-1.22	1.21	***Mettl15***	methyltransferase like 15	0016020 // membrane // inferred from electronic annotation///0016021 // integral component o
1382.3983	1401.8483	1098.0057	1320.7719	NM_026057///XM_0065	0.009	-1.26	1.20	***Zfp422***	zinc finger protein 422	0006464 // cellular protein modification process // inferred from direct assay///0008152 // metab
275.35104	253.3229	315.2326	377.73175	NM_001081054///XR_38	0.003	1.14	1.20	***Qrsl1***	glutaminyl-tRNA synthase (glutamine-hydrolyzing)-like 1	0000122 // negative regulation of transcription from RNA polymerase II promoter // inferred from
234.62373	185.58629	203.69936	243.78697	NM_001162947///NM_C	0.008	-1.15	1.20	***Nek3***	NIMA (never in mitosis gene a)-related expressed kinase 3	0045454 // cell redox homeostasis // inferred from electronic annotation///0055114 // oxidation-
313.19293	322.58344	324.98703	388.3312	NM_001006122///NM_C	0.007	1.04	1.19	***Leo1***	Leo1, Paf1/RNA polymerase II complex component, homolog (S. cerevisiae)	0006810 // transport // inferred from electronic annotation///0006811 // ion transport // inferre
1405.0721	1398.3351	1507.5071	1798.3237	NM_010305	0.009	1.07	1.19	***Gnai1***	guanine nucleotide binding protein (G protein), alpha inhibiting 1	0001916 // positive regulation of T cell mediated cytotoxicity // inferred from direct assay///0001
583.93726	556.37476	2041.3582	2420.064	NM_010145	0.004	3.50	1.19	***Ephx1***	epoxide hydrolase 1, microsomal	0001736 // establishment of planar polarity // inferred from genetic interaction///0006351 // tra
140.51112	136.01991	142.88734	168.73933	NM_001159948///NM_C	0.009	1.02	1.18	***Als2***	amyotrophic lateral sclerosis 2 (juvenile)	0015937 // coenzyme A biosynthetic process // inferred from electronic annotation///0016310 //
125.8917	123.192924	110.34735	129.88864	NM_025473///XM_0065	0.009	-1.14	1.18	***Fam3a***	family with sequence similarity 3, member A	0000082 // G1/S transition of mitotic cell cycle // inferred from mutant phenotype///0000122 // r
92.37111	68.36415	86.19487	101.24989	NM_019577///XM_0065	0.007	-1.07	1.17	***Ccl24***	chemokine (C-C motif) ligand 24	0006397 // mRNA processing // inferred from electronic annotation///0008380 // RNA splicing //
21.38159	17.56756	13.705908	16.02138	NM_001037906	0.005	-1.56	1.17	***Nell1***	NEL-like 1	0006184 // GTP catabolic process // not recorded///0007049 // cell cycle // inferred from electro
335.91214	347.8988	289.04532	337.23972	NM_145221///XM_0065	0.001	-1.16	1.17	***Appl1***	adaptor protein, phosphotyrosine interaction, PH domain and leucine zipper cc	0001824 // blastocyst development // inferred from direct assay
21.299736	19.633522	20.586864	23.849493	NR_003283	0.000	-1.03	1.16	***Rab11fip4os1***	RAB11 family interacting protein 4 (class II), opposite strand 1	0005737 // cytoplasm // inferred from electronic annotation///0005813 // centrosome // not rec
322.906	281.8248	247.32037	283.85355	NM_001276719///NM_C	0.005	-1.31	1.15	***Ackr2***	atypical chemokine receptor 2	0006184 // GTP catabolic process // not recorded///0006886 // intracellular protein transport // i
265.85815	261.5832	256.9423	294.46765	NM_053177///XR_37867	0.004	-1.03	1.15	***Mcoln1***	mucolipin 1	0000910 // cytokinesis // not recorded///0006351 // transcription, DNA-templated // inferred fro
44.707375	38.64207	32.335384	36.822876	NM_010050	0.003	-1.38	1.14	***Dio2***	deiodinase, iodothyronine, type II	0006020 // inositol metabolic process // inferred from mutant phenotype///0006412 // translatio
725.8644	656.58636	729.48694	826.66895	NM_001190343///NM_C	0.003	1.00	1.13	***Ccm2***	cerebral cavernous malformation 2	0006351 // transcription, DNA-templated // inferred from electronic annotation///0006355 // reg
71.6419	58.942333	57.64307	65.162	NM_026956///XM_0065	0.000	-1.24	1.13	***Cd209f***	CD209f antigen	0001711 // endodermal cell fate commitment // inferred from mutant phenotype///0006351 // tr
1607.85	1282.958	1874.1724	2114.7388	NM_001177556///NM_C	0.003	1.17	1.13	***Gng12***	guanine nucleotide binding protein (G protein), gamma 12	0006508 // proteolysis // inferred from electronic annotation
79.64633	65.18264	75.69151	85.17433	NM_013606///NR_0035	0.009	-1.05	1.13	***Mx2***	myxovirus (influenza virus) resistance 2	0006370 // 7-methylguanosine mRNA capping // not recorded///0006397 // mRNA processing //
293.9439	267.09634	246.3518	276.8707	NM_001033533	0.005	-1.19	1.12	***Ccdc102a***	coiled-coil domain containing 102A	0048565 // digestive tract development // not recorded
52.722187	47.619675	40.566326	45.47538	NM_001001985	0.010	-1.30	1.12	***Nat8l***	N-acetyltransferase 8-like	0001539 // cilium or flagellum-dependent cell motility // inferred from sequence or structural sim
286.68628	266.12122	292.28632	323.16782	NM_028276	0.009	1.02	1.11	***Utp14a***	UTP14, U3 small nucleolar ribonucleoprotein, homolog A (yeast)	0006396 // RNA processing // inferred from electronic annotation///0010468 // regulation of gen
193.88818	167.13765	164.93602	181.00475	NM_146248	0.008	-1.18	1.10	***Cchcr1***	coiled-coil alpha-helical rod protein 1	0001525 // angiogenesis // inferred from electronic annotation///0001938 // positive regulation
278.44363	248.53651	179.86086	195.88142	NM_001004361///NM_C	0.008	-1.55	1.09	***Tpgs2***	tubulin polyglutamylase complex subunit 2	0018108 // peptidyl-tyrosine phosphorylation // not recorded///0030334 // regulation of cell mig
1635.2136	1568.6636	1344.5557	1459.1736	NM_024189///NR_0283	0.008	-1.22	1.09	***Yaf2***	YY1 associated factor 2	0006351 // transcription, DNA-templated // inferred from electronic annotation///0006355 // reg
931.02594	858.81775	749.127	811.437	NM_009774///XM_0065	0.009	-1.24	1.08	***Bub3***	budding uninhibited by benzimidazoles 3 homolog (S. cerevisiae)	0006364 // rRNA processing // inferred from electronic annotation///0042254 // ribosome bioger
19.268366	13.963492	15.410205	16.685665	NM_001164682///NM_1	0.006	-1.25	1.08	***Mpp4***	membrane protein, palmitoylated 4 (MAGUK p55 subfamily member 4)	0001921 // positive regulation of receptor recycling // inferred from direct assay///0006461 // pr
4359.0586	4262.3184	4032.455	4360.5083	NM_133933	0.008	-1.08	1.08	***Rpn1***	ribophorin I	0007219 // Notch signaling pathway // inferred from electronic annotation///0034976 // respons
26240.252	25157.268	21278.594	22954.695	NM_011654///XM_0065	0.002	-1.23	1.08	***Gm5620///Tuba1b***	tubulin, alpha 1B pseudogene///tubulin, alpha 1B	0001662 // behavioral fear response // inferred from mutant phenotype///0001881 // receptor re
69.31573	52.203815	35.414585	38.15566	NM_029993	0.009	-1.96	1.08	***Mlana***	melan-A	0006952 // defense response // inferred from electronic annotation
233.28522	222.12654	213.92546	229.1334	NM_001159729///NM_C	0.000	-1.09	1.07	***Rab23***	RAB23, member RAS oncogene family	0007049 // cell cycle // inferred from electronic annotation///0007275 // multicellular organisma
9588.566	9368.786	8762.889	9383.574	NM_001039147///NM_C	0.000	-1.09	1.07	***A730098P11Rik///Gm6747***	mortality factor 4 like 1 pseudogene///predicted gene 6747///mortality factor	0001522 // pseudouridine synthesis // inferred from electronic annotation///0009451 // RNA mo
38.603825	34.09454	31.807558	33.731598	NM_010078///XM_0065	0.001	-1.21	1.06	***Drp2***	dystrophin related protein 2	0006412 // translation // inferred from electronic annotation
27.357296	21.815533	24.821957	26.30147	XR_140713	0.005	-1.10	1.06	***Usp46os2***	ubiquitin specific peptidase 46, opposite strand 2	0006556 // S-adenosylmethionine biosynthetic process // not recorded///0006730 // one-carbon
46.89701	33.35833	38.454548	40.612354	NM_001146002///NM_1	0.006	-1.22	1.06	***Txndc2***	thioredoxin domain containing 2 (spermatozoa)	0007399 // nervous system development // not recorded///0031175 // neuron projection develo
65.49138	54.413994	56.811447	59.714314	NM_001040399///NM_C	0.007	-1.15	1.05	***Larp1b///LOC102632135***	La ribonucleoprotein domain family, member 1B///la-related protein 1B-like	0006351 // transcription, DNA-templated // inferred from electronic annotation///0006355 // reg

[ICR, Air, PBS]	[ICR, Air, SFN	[ICR, O2, PBS](	[ICR, O2, SFN](	RefSeq Transcript ID	p (Prenatal Treatment-Exposure)	FC [Air-PBS: O2-PBS	FC [O2 PBS:SFN]	Gene Symbol	Gene Title	Gene Ontology
22.082022	16.430124	18.113605	18.805227	NM_010666	0.006	-1.22	1.04	***Krt27***	keratin 27	0001568 // blood vessel development // inferred from mutant phenotype///0001570 // vasculoge
33.819256	27.008427	29.399582	30.436289	NM_001129803///NM_C	0.005	-1.15	1.04	***Pcp2***	Purkinje cell protein 2 (L7)	0006355 // regulation of transcription, DNA-templated // not recorded
89.95629	72.791016	77.12211	78.526535	NM_153424///XM_0065	0.004	-1.17	1.02	***Nphp4***	nephronophthisis 4 (juvenile) homolog (human)	0001666 // response to hypoxia // inferred from mutant phenotype///0001666 // response to hyp
27.035168	21.454285	21.628815	21.964022		0.003	-1.25	1.02	***AK007819///Gm13594***	Mus musculus 10 day old male pancreas cDNA, RIKEN full-length enriched libra	0000301 // retrograde transport, vesicle recycling within Golgi // not recorded///0007030 // Golg
88.62353	110.10694	103.15215	104.191345	NM_001163749///NM_C	0.009	1.16	1.01	***Camsap3***	calmodulin regulated spectrin-associated protein family, member 3	0006939 // smooth muscle contraction // inferred from electronic annotation///0007165 // signa
47.30427	37.144222	40.739414	40.724743	NM_031395///NM_1833	0.005	-1.16	-1.00	***LOC101055731///LOC1010***	synaptotagmin-like protein 3-like///synaptotagmin-like 3	
65.868324	46.16065	49.529713	49.381584	NM_022888///NM_1768	0.007	-1.33	-1.00	***Folr4***	folate receptor 4 (delta)	
39.2519	31.465635	32.18398	32.061275	NM_011927	0.010	-1.22	-1.00	***Ceacam9***	carcinoembryonic antigen-related cell adhesion molecule 9	0006810 // transport // inferred from electronic annotation///0006811 // ion transport // inferre
163.18095	227.16814	176.77748	175.95743	NM_026551///XM_0065	0.009	1.08	-1.00	***Dcakd***	dephospho-CoA kinase domain containing	0005515 // protein binding // inferred from electronic annotation
118.85292	92.06245	100.898254	99.16808	NM_013533///XM_0065	0.009	-1.18	-1.02	***Gpr162***	G protein-coupled receptor 162	
56.095997	44.037872	46.720684	45.61277	NM_031873	0.003	-1.20	-1.02	***Tas1r2***	taste receptor, type 1, member 2	0040008 // regulation of growth // inferred from electronic annotation
17355.703	16973.033	17531.281	17099.68	NM_025586///XM_0014	0.007	1.01	-1.03	***Gm10224///Gm4581///Gm***	predicted pseudogene 10224///predicted gene 4581///ribosomal protein L15 p	0006351 // transcription, DNA-templated // inferred from electronic annotation///0006352 // DN
2154.2534	1964.3711	2196.0884	2141.9155	NM_009498///XM_0065	0.003	1.02	-1.03	***Vamp3***	vesicle-associated membrane protein 3	0000122 // negative regulation of transcription from RNA polymerase II promoter // inferred from
3123.0808	2694.7559	3145.3591	3051.4277	NM_016843	0.008	1.01	-1.03	***Atxn10***	ataxin 10	0016020 // membrane // inferred from electronic annotation///0016021 // integral component o
32.235867	24.501385	28.645557	27.645136	NM_032397///XM_0065	0.000	-1.13	-1.04	***Kcnn1***	potassium intermediate/small conductance calcium-activated channel, subfam	0002376 // immune system process // inferred from electronic annotation///0006184 // GTP cata
19.24848	15.291763	18.533731	17.790642	NM_010054	0.000	-1.04	-1.04	***Dlx2***	distal-less homeobox 2	0007126 // meiotic nuclear division // inferred from electronic annotation///0007283 // spermato
188.40999	227.36269	234.92706	223.77342	NM_001039084///NM_C	0.009	1.25	-1.05	***Mrpl1***	mitochondrial ribosomal protein L1	0001649 // osteoblast differentiation // inferred from genetic interaction///0001649 // osteoblas
567.57166	621.99115	756.0955	716.8299	NM_010851	0.008	1.33	-1.05	***Myd88***	myeloid differentiation primary response gene 88	0001938 // positive regulation of endothelial cell proliferation // not recorded///0006935 // chem
889.5055	986.73303	956.73944	903.91754	NM_144904///NM_1781	0.005	1.08	-1.06	***Ptbp3***	polypyrimidine tract binding protein 3	0006281 // DNA repair // inferred from electronic annotation///0006310 // DNA recombination /,
712.7494	759.6339	725.8994	676.767	NM_001039089///NM_C	0.005	1.02	-1.07	***Sel1l***	sel-1 suppressor of lin-12-like (C. elegans)	0006486 // protein glycosylation // inferred from electronic annotation///0018279 // protein N-li
169.6576	194.38829	241.40405	221.69081	NM_001100449///XM_0	0.006	1.42	-1.09	***Taf4b***	TAF4B RNA polymerase II, TATA box binding protein (TBP)-associated factor	0000045 // autophagic vacuole assembly // not recorded///0001843 // neural tube closure // infe
90.67312	114.59314	132.84761	121.66651	NM_025782///NM_0272	0.001	1.47	-1.09	***Ttc39b***	tetratricopeptide repeat domain 39B	0000724 // double-strand break repair via homologous recombination // not recorded///0006281
21.2384	21.939253	19.181307	17.497442	NM_020274	0.001	-1.11	-1.10	***Htr3b***	5-hydroxytryptamine (serotonin) receptor 3B	
94.800545	75.86157	94.42467	85.864525	NM_172794///XM_0065	0.000	1.00	-1.10	***Zfp454***	zinc finger protein 454	0006810 // transport // inferred from electronic annotation///0006811 // ion transport // inferre
2079.7288	2153.2583	2137.7793	1942.9963	NM_011051///XM_0065	0.008	1.03	-1.10	***Pdcd6***	programmed cell death 6	0006810 // transport // inferred from electronic annotation///0006811 // ion transport // inferre
179.09953	189.35603	185.68564	168.6819	NM_001170953///NM_C	0.009	1.04	-1.10	***Rnmt***	RNA (guanine-7-) methyltransferase	0016477 // cell migration // inferred from mutant phenotype///0016477 // cell migration // not r
668.83105	688.84827	644.50146	581.3264	NM_031373///XM_0065	0.007	-1.04	-1.11	***Ogfr***	opioid growth factor receptor	0005576 // extracellular region // inferred from electronic annotation
834.1658	877.58167	778.20465	699.71356	NM_001164156///NM_C	0.000	-1.07	-1.11	***Mcrs1***	microspherule protein 1	0016056 // rhodopsin mediated signaling pathway // inferred from mutant phenotype///0043087
66.30488	65.34878	67.65966	59.837852	NM_001013012///XM_0	0.003	1.02	-1.13	***Zfp787***	zinc finger protein 787	0006468 // protein phosphorylation // inferred from direct assay///0007049 // cell cycle // inferre
95.829056	75.649574	81.587975	72.080086	NM_027010	0.004	-1.17	-1.13	***Crygf***	crystallin, gamma F	0019012 // virion // inferred from electronic annotation///0019013 // viral nucleocapsid // inferre
695.7521	730.0157	805.5714	710.42755	NM_025693///NR_0377	0.001	1.16	-1.13	***Tmem41a***	transmembrane protein 41a	0006886 // intracellular protein transport // inferred from electronic annotation///0006887 // exo
525.6154	540.19104	546.61	481.18762	NM_013924	0.008	1.04	-1.14	***Abt1***	activator of basal transcription 1	0007049 // cell cycle // inferred from electronic annotation///0008283 // cell proliferation // not
101.02462	109.25879	134.81807	118.66679	NM_001081065///XM_0	0.009	1.33	-1.14	***Zfp707***	zinc finger protein 707	0060041 // retina development in camera-type eye // inferred from mutant phenotype
745.66125	801.71515	847.80945	746.1898	NM_001081394///NM_C	0.001	1.14	-1.14	***Tmem248***	transmembrane protein 248	0001654 // eye development // inferred from mutant phenotype
1149.6855	1361.4747	1288.8113	1131.5114	NM_025334	0.003	1.12	-1.14	***Txndc12***	thioredoxin domain containing 12 (endoplasmic reticulum)	0006611 // protein export from nucleus // inferred from direct assay///0007275 // multicellular o
286.95514	322.07632	359.61365	311.13663	NM_183170	0.008	1.25	-1.16	***Mpv17l2***	MPV17 mitochondrial membrane protein-like 2	0007165 // signal transduction // inferred from electronic annotation///0007186 // G-protein cou
12.404591	14.511468	13.22011	11.371908	NM_001039238///NM_C	0.000	1.07	-1.16	***Dcpp1///Dcpp2///Dcpp3***	demilune cell and parotid protein 1///demilune cell and parotid protein 2///de	0007275 // multicellular organismal development // inferred from electronic annotation///00160
681.8119	709.64374	832.71295	715.50055	NM_009057///XM_0065	0.004	1.22	-1.16	***Slc50a1***	solute carrier family 50 (sugar transporter), member 1	
248.0506	270.24194	279.8455	240.37427	NM_029734	0.006	1.13	-1.16	***Wdyhv1***	WDYHV motif containing 1	0001582 // detection of chemical stimulus involved in sensory perception of sweet taste // not re
115.11313	124.87495	147.29564	125.92162	NM_026117///NM_2076	0.002	1.28	-1.17	***Dedd2***	death effector domain-containing DNA binding protein 2	0006468 // protein phosphorylation // not recorded///0006950 // response to stress // not recor
308.2642	380.97968	343.81018	292.78058	NM_080553///XM_0065	0.009	1.12	-1.17	***Itpr3***	inositol 1,4,5-triphosphate receptor 3	0001649 // osteoblast differentiation // not recorded///0006397 // mRNA processing // inferred f
33.810482	47.470654	38.702503	32.906536	NM_029360	0.003	1.14	-1.18	***Tm4sf5***	transmembrane 4 superfamily member 5	
151.4919	210.18614	245.40456	208.30515	NM_001083903///NM_1	0.006	1.62	-1.18	***Sbsn***	suprabasin	0007275 // multicellular organismal development // inferred from electronic annotation
20.539202	20.808317	21.180328	17.902147	NM_001042418///NM_C	0.010	1.03	-1.18	***Cabyr***	calcium-binding tyrosine-(Y)-phosphorylation regulated (fibrousheathin 2)	0050808 // synapse organization // not recorded
1980.7354	1968.3641	1996.457	1682.3652	NM_001199274///NM_1	0.004	1.01	-1.19	***Mat2b***	methionine adenosyltransferase II, beta	0006351 // transcription, DNA-templated // inferred from electronic annotation///0006355 // reg
983.54016	1002.24927	1031.7991	861.6388	NM_145606	0.003	1.05	-1.20	***Chmp1a***	charged multivesicular body protein 1A	0031069 // hair follicle morphogenesis // inferred from mutant phenotype
1007.682	1060.9253	854.2852	711.68445	NM_025915	0.009	-1.18	-1.20	***Tmem88***	transmembrane protein 88	0006457 // protein folding // inferred from direct assay///0006662 // glycerol ether metabolic pro
112.45588	106.81234	128.25092	106.510216	NM_172875///XM_0065	0.007	1.14	-1.20	***Azin2***	antizyme inhibitor 2	0007565 // female pregnancy // inferred from electronic annotation
1369.2303	1433.6484	1616.869	1333.6819	NM_001199009///NM_1	0.009	1.18	-1.21	***Dcaf11***	DDB1 and CUL4 associated factor 11	0032259 // methylation // inferred from electronic annotation
270.13675	307.58252	340.55954	280.72928	NM_001004190	0.004	1.26	-1.21	***Zfp560***	zinc finger protein 560	0000226 // microtubule cytoskeleton organization // inferred from direct assay///0006184 // GTP
924.38574	939.7349	906.2641	737.0504	NM_001199004///NM_C	0.007	-1.02	-1.23	***Golga5***	golgi autoantigen, golgin subfamily a, 5	0006351 // transcription, DNA-templated // inferred from electronic annotation///0006355 // reg
350.96143	360.64636	407.9188	327.77188	NM_013770	0.005	1.16	-1.24	***Slc25a10***	solute carrier family 25 (mitochondrial carrier, dicarboxylate transporter), mem	0000070 // mitotic sister chromatid segregation // inferred from mutant phenotype///0007049 //
239.12836	276.19635	247.31636	196.78394	NM_133733///XM_0065	0.005	1.03	-1.26	***Clmp***	CXADR-like membrane protein	0005537 // mannose binding // inferred from direct assay///0030246 // carbohydrate binding // i
924.40405	916.7021	981.994	779.34906	NM_029354///XM_0065	0.007	1.06	-1.26	***Mzt2***	mitotic spindle organizing protein 2	
109.7685	105.43333	129.01692	102.36427	NM_001164567///NM_C	0.005	1.18	-1.26	***Vill***	villin-like	0035418 // protein localization to synapse // inferred from mutant phenotype
34.793518	34.548595	33.996597	26.567661	NM_001285482///NM_C	0.008	-1.02	-1.28	***Htr1d***	5-hydroxytryptamine (serotonin) receptor 1D	0006351 // transcription, DNA-templated // inferred from electronic annotation///0006355 // reg
4619.4604	4740.4546	9125.774	7125.0015	NM_007801	0.008	1.98	-1.28	***Ctsh***	cathepsin H	0008544 // epidermis development // inferred from electronic annotation
4124.035	4033.8813	4911.382	3813.3694	NM_007657	0.009	1.19	-1.29	***Cd9***	CD9 antigen	0006508 // proteolysis // inferred from electronic annotation///0006749 // glutathione metabolic
58.654034	66.26042	57.22359	44.23415	NR_040406///NR_04040	0.007	-1.02	-1.29	***Gm16845***	predicted gene, 16845	0008152 // metabolic process // not recorded///0051586 // positive regulation of dopamine upta
94.84323	114.48559	238.80887	184.10858	NM_009897///XM_0064	0.003	2.52	-1.30	***Ckmt1***	creatine kinase, mitochondrial 1, ubiquitous	0006898 // receptor-mediated endocytosis // not recorded///0006935 // chemotaxis // inferred f
160.46207	220.5536	441.01245	337.7131	NM_001083894///NM_C	0.005	2.75	-1.31	***Liph***	lipase, member H	0008277 // regulation of G-protein coupled receptor protein signaling pathway // inferred from g
212.60832	182.8558	214.94002	164.22755	NM_028009///XM_0065	0.006	1.01	-1.31	***Rpusd1***	RNA pseudouridylate synthase domain containing 1	
103.562805	122.44802	153.63472	115.27902	NM_001082975///XM_0	0.007	1.48	-1.33	***Sdr39u1***	short chain dehydrogenase/reductase family 39U, member 1	0007155 // cell adhesion // inferred from mutant phenotype///0007338 // single fertilization // ir
609.43494	616.3825	585.8399	433.93536	NM_001270495///NM_C	0.003	-1.04	-1.35	***Tmem254a///Tmem254b/***	transmembrane protein 254a///transmembrane protein 254b///transmembrar	0006590 // thyroid hormone generation // not recorded///0009409 // response to cold // not rec
500.6313	546.75055	652.7085	476.94382	NM_011611///NM_1707	0.006	1.30	-1.37	***Cd40***	CD40 antigen	0007155 // cell adhesion // inferred from electronic annotation///0008285 // negative regulation
247.25008	274.07913	330.198	239.52315	NM_001243584///NM_C	0.005	1.34	-1.38	***Mif4gd***	MIF4G domain containing	0006810 // transport // inferred from electronic annotation///0015031 // protein transport // infe
184.00974	175.80466	252.7704	182.76837	NM_001286038///NM_C	0.009	1.37	-1.38	***Klc3***	kinesin light chain 3	0006810 // transport // inferred from electronic annotation///0006811 // ion transport // inferre
1144.5792	1152.8401	1104.2269	796.5783	NM_001270495///NM_C	0.004	-1.04	-1.39	***Tmem254a///Tmem254b/***	transmembrane protein 254a///transmembrane protein 254b///transmembrar	0001764 // neuron migration // inferred from electronic annotation///0006184 // GTP catabolic p
3097.0762	2950.8845	3751.1406	2688.6208	NM_010261	0.005	1.21	-1.40	***Rabac1***	Rab acceptor 1 (prenylated)	0005737 // cytoplasm // inferred from electronic annotation///0005856 // cytoskeleton // inferre
59.049698	52.857567	55.86813	39.78884	NM_027622	0.006	-1.06	-1.40	***Tex19.2***	testis expressed gene 19.2	0000922 // spindle pole // not recorded///0005737 // cytoplasm // not recorded///0005814 // ce
1979.6515	1891.6511	2086.9712	1478.3418	NM_009101///XM_0065	0.002	1.05	-1.41	***Rras***	Harvey rat sarcoma oncogene, subgroup R	0010468 // regulation of gene expression // not recorded///0030154 // cell differentiation // infe
90.2437	104.66821	185.40825	127.885994	NM_175638///XM_0065	0.007	2.05	-1.45	***Wnk4***	WNK lysine deficient protein kinase 4	
81.064354	73.11081	82.32966	54.972485	NM_001007582	0.000	1.02	-1.50	***AY761184***	cDNA sequence AY761184	
4887.4385	4920.806	9818.816	6502.976	NM_011313	0.010	2.01	-1.51	***S100a6***	S100 calcium binding protein A6 (calcyclin)	0001525 // angiogenesis // inferred from electronic annotation///0007155 // cell adhesion // infe
923.67535	903.7491	1084.5637	705.1583	NM_010579	0.008	1.17	-1.54	***Eif6***	eukaryotic translation initiation factor 6	0007165 // signal transduction // not recorded///0022414 // reproductive process // inferred fror
239.50897	255.41904	419.132	270.9621	NM_009285	0.006	1.75	-1.55	***Stc1***	stanniocalcin 1	0010842 // retina layer formation // inferred from mutant phenotype///0045494 // photorecepto
517.8162	612.18823	828.7832	535.056	NM_001177187///NM_C	0.009	1.60	-1.55	***Mal***	myelin and lymphocyte protein, T cell differentiation protein	0007154 // cell communication // inferred from electronic annotation///0007155 // cell adhesion
74.855606	82.43446	140.45987	80.1871	NM_001161714///NM_C	0.007	1.88	-1.75	***Tgm1***	transglutaminase 1, K polypeptide	0005789 // endoplasmic reticulum membrane // not recorded///0005794 // Golgi apparatus // no
104.49007	86.03608	151.65457	83.80014	NM_001173372///NM_C	0.001	1.45	-1.81	***Fxyd4***	FXYD domain-containing ion transport regulator 4	0007049 // cell cycle // inferred from electronic annotation///0007059 // chromosome segregati
893.774	1192.8127	2710.3591	1236.5773	NM_018857///XM_0065	0.010	3.03	-2.19	***Msln***	mesothelin	0008360 // regulation of cell shape // inferred from electronic annotation
60.04707	104.37409	127.874115	56.07898	NM_177595	0.002	2.13	-2.28	***Mkx***	mohawk homeobox	0006355 // regulation of transcription, DNA-templated // inferred from electronic annotation///0
112.696655	123.45832	204.81178	89.63208	NM_174988///XM_0064	0.005	1.82	-2.29	***Cdh22***	cadherin 22	0006184 // GTP catabolic process // inferred from electronic annotation///0007165 // signal trans

[ICR, Air, PBS]	[ICR, Air, SFN	[ICR, O2, PBS](	[ICR, O2, SFN](	RefSeq Transcript ID	p (Prenatal Treatment-Exposure)	FC [Air-PBS: O2-PBS	FC [O2 PBS:SFN]	Gene Symbol	Gene Title	Gene Ontology
1985.1251	2506.2043	2176.9446	934.4288	NM_001267808///NM_C	0.004	1.10	-2.33	***H2-D1///H2-L///H2-Q2///L***	histocompatibility 2, D region locus 1///histocompatibility 2, D region locus L//,	0007049 // cell cycle // inferred from electronic annotation///0007067 // mitotic nuclear division
1195.5133	1377.2422	5246.056	1710.0326	NM_008491	0.001	4.39	-3.07	***Lcn2***	lipocalin 2	0006508 // proteolysis // inferred from electronic annotation///0008152 // metabolic process // i

Table S4. Prenatal sulforaphane and neonatal hyperoxia interaction 239 genes in *Nrf2* $^{-/-}$ mice (2-Way ANOVA, p<=0.01)

RIKEN cDNA and predicted genes excluded. Fold Change (FC): Blue (-) - fold lower in SFN:O2 than PBS:O2, Red - fold higher in SFN:O2 than PBS:O2.

[Nrf2-KO, Air, PBS](r	[Nrf2-KO, Air, SFN](	[Nrf2-KO, O2, PBS](	[Nrf2-KO, O2, SFN](	RefSeq Transcript ID	p (Prenatal Treatment-Exposure)	FC [Air-PBS:O2-PBS]	FC [O2 PBS:SFN]	Gene Symbol	Gene Title	Gene Ontology
30.43497	62.552834	18.06089	55.91045	NM_001110505///NI	0.002	-1.69	3.10	***Amy1///Rnpc3***	amylase 1, salivary///RNA-binding region (RNP1, RRM) containing 3	0005975 // carbohydrate metabolic process // inferred from electronic annotation///0008152 // metabolic process // inferred from
164.04744	120.011406	80.92515	181.10664	NM_001168318///NI	0.007	-2.03	2.24	***Scara5***	scavenger receptor class A, member 5 (putative)	0006810 // transport // inferred from electronic annotation///0006811 // ion transport // inferred from electronic annotation///00
296.55664	351.29684	165.7877	301.2976	NM_172592///XM_0	0.000	-1.79	1.82	***Srek1***	splicing regulatory glutamine/lysine-rich protein 1	0006397 // mRNA processing // inferred from electronic annotation///0008380 // RNA splicing // inferred from electronic annotatic
149.86345	140.53175	83.280876	149.06094	NM_007986///XM_0	0.008	-1.80	1.79	***Fap***	fibroblast activation protein	0006508 // proteolysis // not recorded///0010716 // negative regulation of extracellular matrix disassembly // not recorded///0043
148.7259	138.15648	86.14557	149.32826	NM_181815///XM_0	0.003	-1.73	1.73	***Cep128***	centrosomal protein 128	0000922 // spindle pole // not recorded///0005737 // cytoplasm // not recorded///0005814 // centriole // not recorded///0005815
501.66388	504.0752	253.45163	418.06332	NM_172274///XM_0	0.005	-1.98	1.65	***Cc2d2a***	coiled-coil and C2 domain containing 2A	0007224 // smoothened signaling pathway // inferred from mutant phenotype///0030030 // cell projection organization // inferred
1138.598	1255.1573	663.9116	1089.2871	NM_001293660///NI	0.002	-1.71	1.64	***Uimc1***	ubiquitin interaction motif containing 1	0006281 // DNA repair // inferred from electronic annotation///0006302 // double-strand break repair // not recorded///0006351 /
580.33966	720.61646	444.56494	722.035	NM_018759///NR_03	0.007	-1.31	1.62	***Zfp326***	zinc finger protein 326	0006351 // transcription, DNA-templated // inferred from electronic annotation///0006355 // regulation of transcription, DNA-temp
403.37616	480.9631	293.49054	461.5749	NM_029852///XM_0	0.007	-1.37	1.57	***Cep83***	centrosomal protein 83	0030030 // cell projection organization // inferred from electronic annotation///0042384 // cilium assembly // inferred from mutan
87.26366	79.30841	56.3444	88.290115	NM_001081091///XI	0.002	-1.55	1.57	***Cep152***	centrosomal protein 152	0007099 // centriole replication // inferred from mutant phenotype///0030030 // cell projection organization // inferred from elect
97.05961	104.466064	67.417816	105.381386	NM_134071///XM_0	0.003	-1.44	1.56	***Ankrd32***	ankyrin repeat domain 32	0005515 // protein binding // inferred from physical interaction
390.91913	356.35443	285.1844	445.35056	NM_013752///XM_0	0.010	-1.37	1.56	***Nbn***	nibrin	0000077 // DNA damage checkpoint // not recorded///0000723 // telomere maintenance // not recorded///0001701 // in utero em
819.4389	794.76324	594.85626	921.46344	NM_019832///XM_0	0.001	-1.38	1.55	***Gkap1***	G kinase anchoring protein 1	0007165 // signal transduction // inferred from direct assay///0007199 // G-protein coupled receptor signaling pathway coupled to
125.97553	157.84042	69.445984	106.32285	NM_146208	0.008	-1.81	1.53	***Neil3***	nei like 3 (E. coli)	0000737 // DNA catabolic process, endonucleolytic // inferred from direct assay///0000737 // DNA catabolic process, endonucleoly
206.59949	229.93423	149.23407	227.45866	NM_001252560///NI	0.007	-1.38	1.52	***Mllt10***	myeloid/lymphoid or mixed-lineage leukemia (trithorax homolog, Drosopl	0045944 // positive regulation of transcription from RNA polymerase II promoter // not recorded
769.2491	593.7449	435.25687	660.087	NM_001159485///NI	0.008	-1.77	1.52	***Mcf2l***	mcf.2 transforming sequence-like	0007266 // Rho protein signal transduction // inferred from direct assay///0032319 // regulation of Rho GTPase activity // inferred f
2090.2502	1846.058	992.71875	1502.7582	NM_133786///XM_0	0.004	-2.11	1.51	***Smc4***	structural maintenance of chromosomes 4	0006281 // DNA repair // inferred from electronic annotation///0006310 // DNA recombination // inferred from electronic annotati
316.98578	366.6966	236.68385	343.1388	NM_194262///NM_1	0.004	-1.34	1.45	***Arid4b***	AT rich interactive domain 4B (RBP1-like)	0006349 // regulation of gene expression by genetic imprinting // inferred from genetic interaction///0006351 // transcription, DNA
359.3893	318.17798	232.2609	335.82376	NM_011782	0.009	-1.55	1.45	***Adamts5***	a disintegrin-like and metallopeptidase (reprolysin type) with thrombospo	0006508 // proteolysis // not recorded///0042742 // defense response to bacterium // inferred from genetic interaction
192.4676	163.65924	145.56058	207.82658	NM_027547///XM_0	0.007	-1.32	1.43	***Prdm5***	PR domain containing 5	0000278 // mitotic cell cycle // not recorded///0006351 // transcription, DNA-templated // inferred from electronic annotation///0
2152.6763	2098.7014	1185.8275	1686.2844	NM_001159382///NI	0.006	-1.82	1.42	***Gjc1***	gap junction protein, gamma 1	0001570 // vasculogenesis // inferred from mutant phenotype///0007154 // cell communication // inferred from electronic annotat
346.87097	313.70596	274.17102	387.09793	NM_001291229///NI	0.003	-1.27	1.41	***Clip1***	CAP-GLY domain containing linker protein 1	0001578 // microtubule bundle formation // not recorded///0006810 // transport // inferred from electronic annotation///003111€
552.95056	542.41986	354.04962	484.8515	NM_001168382///NI	0.000	-1.56	1.37	***Phf14***	PHD finger protein 14	0000122 // negative regulation of transcription from RNA polymerase II promoter // inferred from direct assay///0008285 // negati
616.0399	625.335	360.28082	491.8442	NM_001113573///NI	0.008	-1.71	1.37	***Brd3***	bromodomain containing 3	0006351 // transcription, DNA-templated // inferred from electronic annotation///0006355 // regulation of transcription, DNA-temp
522.3191	470.42346	311.16275	424.3836	NM_028283///XM_0	0.003	-1.68	1.36	***Uaca***	uveal autoantigen with coiled-coil domains and ankyrin repeats	0008630 // intrinsic apoptotic signaling pathway in response to DNA damage // inferred from mutant phenotype///0008631 // intri
605.2108	547.0723	403.34418	545.2563	NM_025695///XM_0	0.005	-1.50	1.35	***Smc6***	structural maintenance of chromosomes 6	0000722 // telomere maintenance via recombination // not recorded///0000724 // double-strand break repair via homologous reco
278.2317	268.71054	188.67926	254.53407	NM_021346///NM_2	0.002	-1.47	1.35	***Zfp318***	zinc finger protein 318	0006351 // transcription, DNA-templated // inferred from electronic annotation///0006355 // regulation of transcription, DNA-temp
6317.777	5908.292	3917.3706	5263.9946	NM_010097///XM_0	0.001	-1.61	1.34	***Sparcl1***	SPARC-like 1	0007165 // signal transduction // inferred from electronic annotation
896.49054	879.40625	611.96857	815.91095	NM_001080129///NI	0.002	-1.46	1.33	***Tmpo***	thymopoietin	0006355 // regulation of transcription, DNA-templated // inferred from direct assay
6165.088	6360.7036	4073.8577	5380.118	NM_025900///XR_38	0.009	-1.51	1.32	***Dek***	DEK oncogene (DNA binding)	0016568 // chromatin modification // inferred from electronic annotation///2000779 // regulation of double-strand break repair //
107.28284	109.170784	67.17059	88.547195	NM_080850///XM_0	0.005	-1.60	1.32	***Pask***	PAS domain containing serine/threonine kinase	0006355 // regulation of transcription, DNA-templated // inferred from electronic annotation///0006468 // protein phosphorylatior
232.44029	192.35313	171.36322	225.11345	NM_001033314///XI	0.004	-1.36	1.31	***Ccdc61***	coiled-coil domain containing 61	0005813 // centrosome // not recorded
642.24615	673.546	644.0773	844.5976	NM_001164325///NI	0.004	1.00	1.31	***Trpm7***	transient receptor potential cation channel, subfamily M, member 7	0006468 // protein phosphorylation // inferred from direct assay///0006468 // protein phosphorylation // not recorded///0006810
46.577065	47.20413	50.454594	65.92652	NM_001013390	0.001	1.08	1.31	***Scn4b***	sodium channel, type IV, beta	0006810 // transport // inferred from electronic annotation///0006811 // ion transport // inferred from electronic annotation///00
1859.1542	1915.0986	1518.8088	1968.3252	NM_001205226///NI	0.004	-1.22	1.30	***Cnot1///Gm615***	CCR4-NOT transcription complex, subunit 1///predicted gene 6158	0000122 // negative regulation of transcription from RNA polymerase II promoter // not recorded///0001829 // trophectodermal ce
123.61311	104.78732	75.78136	98.17516	NM_001024856///NI	0.002	-1.63	1.30	***Ttbk2///Cdan1***	tau tubulin kinase 2///congenital dyserythropoietic anemia, type I (humar	0006468 // protein phosphorylation // inferred from electronic annotation///0006998 // nuclear envelope organization // traceable
370.61127	318.66232	252.30031	326.82578	NM_026083///NM_0	0.003	-1.47	1.30	***Zc3h13***	zinc finger CCCH type containing 13	0005515 // protein binding // inferred from electronic annotation///0046872 // metal ion binding // inferred from electronic annota
134.22327	142.39105	93.42834	119.74962	NM_028301///XM_0	0.002	-1.44	1.28	***Anks3***	ankyrin repeat and sterile alpha motif domain containing 3	0005515 // protein binding // inferred from electronic annotation
529.28296	412.5257	322.8981	410.95877	NM_009675///XM_0	0.001	-1.64	1.27	***Aoc3***	amine oxidase, copper containing 3	0002523 // leukocyte migration involved in inflammatory response // not recorded///0002675 // positive regulation of acute inflam
336.13425	283.21555	189.46014	239.76797	NM_031877///XM_0	0.009	-1.77	1.27	***Wasf1***	WAS protein family, member 1	0016601 // Rac protein signal transduction // not recorded///0030036 // actin cytoskeleton organization // inferred from electronic
125.717606	111.29772	91.893486	115.674736	NM_001025156///NI	0.006	-1.37	1.26	***Ccdc93***	coiled-coil domain containing 93	
231.62047	197.27098	125.02411	156.82053	NM_183091	0.007	-1.85	1.25	***Tonsl***	tonsoku-like, DNA repair protein	0000724 // double-strand break repair via homologous recombination // not recorded///0006281 // DNA repair // inferred from ele
1045.4713	1050.107	761.31744	949.0334	NM_001080819///XI	0.003	-1.37	1.25	***Arid1a***	AT rich interactive domain 1A (SWI-like)	0000122 // negative regulation of transcription from RNA polymerase II promoter // inferred from direct assay///0001843 // neural
576.9472	564.49304	558.7324	694.78467	NM_025816///XM_0	0.005	-1.03	1.24	***Tax1bp1***	Tax1 (human T cell leukemia virus type I) binding protein 1	0006915 // apoptotic process // inferred from electronic annotation///0032088 // negative regulation of NF-kappaB transcription fa
527.58356	492.3979	440.6817	545.8809	NM_011263///XM_0	0.006	-1.20	1.24	***Rest***	RE1-silencing transcription factor	0000122 // negative regulation of transcription from RNA polymerase II promoter // inferred from direct assay///0000122 // negati
1100.5505	996.8207	991.5166	1225.626	NM_012042///XM_0	0.003	-1.11	1.24	***Cul1***	cullin 1	0006511 // ubiquitin-dependent protein catabolic process // inferred by curator///0006513 // protein monoubiquitination // inferre
542.0672	499.36935	298.58173	366.81128	NM_207255///XM_0	0.001	-1.82	1.23	***Zfp532***	zinc finger protein 532	0006351 // transcription, DNA-templated // inferred from electronic annotation///0006355 // regulation of transcription, DNA-temp
342.8479	341.58945	237.26791	291.28525	NM_026236///XM_0	0.010	-1.44	1.23	***Wdr48***	WD repeat domain 48	0000724 // double-strand break repair via homologous recombination // inferred from mutant phenotype///0006974 // cellular res
202.27295	207.67937	150.56552	184.24878	NM_001029979///XI	0.002	-1.34	1.22	***Safb2***	scaffold attachment factor B2	0006351 // transcription, DNA-templated // inferred from electronic annotation///0006355 // regulation of transcription, DNA-temp
455.01553	431.6415	325.21652	397.85287	NM_175550///XM_0	0.001	-1.40	1.22	***Ap4e1***	adaptor-related protein complex AP-4, epsilon 1	0006810 // transport // inferred from electronic annotation///0006886 // intracellular protein transport // traceable author statem
1024.6508	769.35126	432.3137	526.85657	NM_001083316///NI	0.003	-2.37	1.22	***Pdgfra***	platelet derived growth factor receptor, alpha polypeptide	0001553 // luteinization // inferred from genetic interaction///0001553 // luteinization // inferred from mutant phenotype///00017
426.96844	414.52777	327.6742	396.5191	NM_001253755///NI	0.003	-1.30	1.21	***Tdrd3***	tudor domain containing 3	0006397 // mRNA processing // inferred from electronic annotation///0016568 // chromatin modification // inferred from electroni
16.2374	15.597952	15.739256	18.98675	NM_010250///XM_0	0.010	-1.03	1.21	***Gabra1***	gamma-aminobutyric acid (GABA) A receptor, subunit alpha 1	0006810 // transport // inferred from electronic annotation///0006811 // ion transport // inferred from electronic annotation///00
1817.7969	1731.7142	1668.0889	2010.2202	NM_011879	0.001	-1.09	1.21	***Ik***	IK cytokine	0005515 // protein binding // inferred from electronic annotation///0042802 // identical protein binding // not recorded
36.452366	29.949707	31.675726	37.947666	NM_027425///XM_0	0.010	-1.15	1.20	***Rufy2***	RUN and FYVE domain-containing 2	0046872 // metal ion binding // inferred from electronic annotation
1267.6598	1179.8148	899.80347	1073.9934	NM_152810	0.001	-1.41	1.19	***Cdc5l***	cell division cycle 5-like (S. pombe)	0000398 // mRNA splicing, via spliceosome // not recorded///0006351 // transcription, DNA-templated // inferred from electronic a
73.20252	70.93926	73.55992	87.13684	NM_001004185///XI	0.007	1.00	1.18	***Whamm***	WAS protein homolog associated with actin, golgi membranes and microt	0007015 // actin filament organization // inferred from electronic annotation
1864.5806	1702.9619	1214.6344	1437.2703	NM_001012518///NI	0.006	-1.54	1.18	***Ehmt1///LOC10***	euchromatic histone methyltransferase 1///uncharacterized LOC1026412	0000122 // negative regulation of transcription from RNA polymerase II promoter // inferred from mutant phenotype///0006306 //
453.36725	449.99512	413.69885	488.01813	NM_001012309	0.005	-1.10	1.18	***Ccdc55***	coiled-coil domain containing 55	0000381 // regulation of alternative mRNA splicing, via spliceosome // not recorded///0006397 // mRNA processing // inferred fron
403.41855	353.33722	331.53543	390.91965	NM_026141///XM_0	0.007	-1.22	1.18	***Ppil4***	peptidylprolyl isomerase (cyclophilin)-like 4	0000413 // protein peptidyl-prolyl isomerization // inferred from electronic annotation///0006457 // protein folding // inferred fror
17.056084	14.5023575	16.210402	19.06417	NM_001081134///XI	0.008	-1.05	1.18	***Kcng1***	potassium voltage-gated channel, subfamily G, member 1	0006810 // transport // inferred from electronic annotation///0006811 // ion transport // inferred from electronic annotation///00
189.61726	162.25114	131.2024	153.78165	NM_001110212///NI	0.009	-1.45	1.17	***Gigyf2***	GRB10 interacting GYF protein 2	0007631 // feeding behavior // inferred from mutant phenotype///0008344 // adult locomotory behavior // inferred from mutant p
866.5081	710.4623	649.5939	757.8464	NM_198023///XM_0	0.001	-1.33	1.17	***Rcor1***	REST corepressor 1	0000122 // negative regulation of transcription from RNA polymerase II promoter // not recorded///0006351 // transcription, DNA-
332.4119	297.36484	244.24193	283.8639	NM_172674///XM_0	0.008	-1.36	1.16	***Phf20***	PHD finger protein 20	0006351 // transcription, DNA-templated // inferred from electronic annotation///0006355 // regulation of transcription, DNA-temp
2978.911	2767.609	2719.287	3133.1675	NM_026025///XM_0	0.005	-1.10	1.15	***Zcrb1***	zinc finger CCHC-type and RNA binding motif 1	0006397 // mRNA processing // inferred from electronic annotation///0008380 // RNA splicing // inferred from electronic annotatic
386.69894	343.16782	299.62823	340.9427	NM_001007568	0.009	-1.29	1.14	***Zfp251***	zinc finger protein 251	0006355 // regulation of transcription, DNA-templated // not recorded
6624.9424	6139.3486	5813.087	6607.7236	NM_031165	0.003	-1.14	1.14	***Hspa8***	heat shock protein 8	0000902 // cell morphogenesis // inferred from electronic annotation///0006200 // ATP catabolic process // inferred from direct as
48.843365	42.1303	43.581356	49.346752	NM_001191023///NI	0.003	-1.12	1.13	***Dync1i1***	dynein cytoplasmic 1 intermediate chain 1	0006810 // transport // inferred from electronic annotation///0007018 // microtubule-based movement // inferred from electronic
43.61039	39.804005	40.716652	45.90292	NM_007519///XM_0	0.006	-1.07	1.13	***Baat***	bile acid-Coenzyme A: amino acid N-acyltransferase	0001889 // liver development // inferred from electronic annotation///0002152 // bile acid conjugation // not recorded///0006544
1215.8425	1133.2877	1009.72906	1136.1305	NM_001081975///NI	0.004	-1.20	1.13	***Mfap1a///Mfa***	microfibrillar-associated protein 1A///microfibrillar-associated protein 1B,	0044822 // poly(A) RNA binding // not recorded
101.1876	94.33658	83.07875	93.05931	NM_001272028///NI	0.010	-1.22	1.12	***Arvcf***	armadillo repeat gene deleted in velo-cardio-facial syndrome	0007155 // cell adhesion // inferred from electronic annotation///0007275 // multicellular organismal development // inferred from
1798.3915	1653.173	1451.014	1624.3701	NM_001007589///NI	0.002	-1.24	1.12	***Akirin2***	akirin 2	0000122 // negative regulation of transcription from RNA polymerase II promoter // not recorded///0002376 // immune system pro
905.9772	713.10516	690.20703	768.7407	NM_026934	0.008	-1.31	1.11	***Zc3h15***	zinc finger CCCH-type containing 15	0019221 // cytokine-mediated signaling pathway // inferred from direct assay
798.3939	656.43274	688.1765	763.1919	NM_012024///XM_0	0.009	-1.16	1.11	***Ppp2r5e***	protein phosphatase 2, regulatory subunit B', epsilon	0007165 // signal transduction // inferred from electronic annotation///0050790 // regulation of catalytic activity // inferred from e
1899.3019	1697.4177	1663.772	1840.9154	NM_001122963///NI	0.001	-1.14	1.11	***Gpbp1***	GC-rich promoter binding protein 1	0006351 // transcription, DNA-templated // inferred from electronic annotation///0006355 // regulation of transcription, DNA-temp
23.553553	22.690693	22.403118	24.616014	NM_001033382///XI	0.008	-1.05	1.10	***Cacna2d4***	calcium channel, voltage-dependent, alpha 2/delta subunit 4	0006810 // transport // inferred from electronic annotation///0006811 // ion transport // inferred from electronic annotation///00
19.911793	14.746192	16.15946	17.654142	NR_045119///NR_04	0.010	-1.23	1.09	***Nlrp5-ps***	NLR family, pyrin domain containing 5, pseudogene	
1086.2549	889.64136	898.6396	981.12585	NM_145452///XR_38	0.009	-1.21	1.09	***Rasa1***	RAS p21 protein activator 1	0001570 // vasculogenesis // inferred from sequence or structural similarity///0001570 // vasculogenesis // inferred from mutant p
24.682493	19.322618	23.377768	24.90438	NM_001099631	0.001	-1.06	1.07	***Sh2d5***	SH2 domain containing 5	0005515 // protein binding // inferred from electronic annotation
60.109657	51.34152	57.125874	60.139706	NM_010827///XM_0	0.001	-1.05	1.05	***Msc***	musculin	0000122 // negative regulation of transcription from RNA polymerase II promoter // inferred from direct assay///0000122 // negati
60.447395	49.469555	52.858425	55.333218	NM_009314	0.000	-1.14	1.05	***Tacr2***	tachykinin receptor 2	0007165 // signal transduction // inferred from electronic annotation///0007186 // G-protein coupled receptor signaling pathway /
39.58425	32.221634	34.22559	35.684746	NM_001080934///XI	0.007	-1.16	1.04	***Slc16a5***	solute carrier family 16 (monocarboxylic acid transporters), member 5	0006810 // transport // inferred from electronic annotation///0055085 // transmembrane transport // inferred from electronic ann
40.117584	31.673004	35.33147	36.818176	NM_001160353///XR	0.009	-1.14	1.04	***Grm2///LOC10***	glutamate receptor, metabotropic 2///uncharacterized LOC102636702	0001662 // behavioral fear response // traceable author statement///0007165 // signal transduction // inferred from electronic ann
16.182919	13.113488	12.953314	13.494307	NM_172553///XM_0	0.005	-1.25	1.04	***Alx1***	ALX homeobox 1	0000122 // negative regulation of transcription from RNA polymerase II promoter // not recorded///0001843 // neural tube closure
1520.593	1304.0948	1286.7894	1331.0775	NM_023054	0.008	-1.18	1.03	***Utp3***	UTP3, small subunit (SSU) processome component, homolog (S. cerevisiae	0007275 // multicellular organismal development // inferred from electronic annotation///0007420 // brain development // inferre
109.83684	100.4865	93.86389	96.279495	NM_016717///XM_0	0.010	-1.17	1.03	***Scly***	selenocysteine lyase	0001887 // selenium compound metabolic process // inferred from mutant phenotype///0006629 // lipid metabolic process // infer
46.306583	40.846657	46.80687	47.587044	NM_053245///XM_0	0.006	1.01	1.02	***Aipl1***	aryl hydrocarbon receptor-interacting protein-like 1	0001895 // retina homeostasis // inferred from mutant phenotype///0006457 // protein folding // inferred from electronic annotat
64.66186	53.546825	57.562576	57.9626	NM_023695///XM_0	0.003	-1.12	1.01	***Crybb1***	crystallin, beta B1	0005212 // structural constituent of eye lens // not recorded///0005515 // protein binding // inferred from physical interaction
1972.9261	1748.3971	1936.7028	1938.2922	NM_175341///NM_2	0.006	-1.02	1.00	***Mbnl2***	muscleblind-like 2	0000381 // regulation of alternative mRNA splicing, via spliceosome // inferred from mutant phenotype///0000381 // regulation of
24.71157	19.674719	22.441284	21.66642	NM_029416///XM_0	0.002	-1.10	-1.04	***Klf17***	Kruppel-like factor 17	0006351 // transcription, DNA-templated // inferred from electronic annotation///0006355 // regulation of transcription, DNA-temp
77.80748	51.41216	38.58872	36.934734	NM_010014///NM_1	0.004	-2.02	-1.04	***Dab1***	disabled 1	0001764 // neuron migration // inferred from genetic interaction///0001764 // neuron migration // inferred from mutant phenotyp
50.61561	43.89813	43.192368	40.871033	NM_011993///XM_0	0.009	-1.17	-1.06	***Dpysl4***	dihydropyrimidinase-like 4	0006208 // pyrimidine nucleobase catabolic process // inferred from electronic annotation///0008152 // metabolic process // inferr

[Nrf2-KO, Air, PBS](r	[Nrf2-KO, Air, SFN](	[Nrf2-KO, O2, PBS](	[Nrf2-KO, O2, SFN](	RefSeq Transcript ID	p (Prenatal Treatment-Exposure)	FC [Air-PBS:O2-PBS]	FC [O2 PBS:SFN]	Gene Symbol	Gene Title	Gene Ontology
178.3814	216.55286	169.99596	159.91408	NM_001136471///NI	0.009	-1.05	-1.06	***Ccdc9***	coiled-coil domain containing 9	0044822 // poly(A) RNA binding // not recorded
689.30695	776.723	832.0323	771.9213	NM_001081109///XI	0.005	1.21	-1.08	***Lmtk2***	lemur tyrosine kinase 2	0001881 // receptor recycling // not recorded///0006468 // protein phosphorylation // not recorded///0007399 // nervous system
51.17158	62.236813	61.230568	56.107067	NR_003552	0.007	1.20	-1.09	***Cyp2d13***	cytochrome P450, family 2, subfamily d, polypeptide 13	
38.441982	45.65772	46.57363	42.662933	NM_026251///XM_0	0.009	1.21	-1.09	***Patl2***	protein associated with topoisomerase II homolog 2 (yeast)	0010607 // negative regulation of cytoplasmic mRNA processing body assembly // inferred from direct assay///0010607 // negative
233.65855	311.17303	445.2237	404.64017	NM_001271587///NI	0.006	1.91	-1.10	***Eps8***	epidermal growth factor receptor pathway substrate 8	0008344 // adult locomotory behavior // inferred from mutant phenotype///0008360 // regulation of cell shape // inferred from mu
5081.1147	5684.7954	6693.64	6081.206	NM_001205385///NI	0.003	1.32	-1.10	***Actr3***	ARP3 actin-related protein 3	0007163 // establishment or maintenance of cell polarity // inferred from genetic interaction///0008356 // asymmetric cell division
1576.7903	1184.5223	1455.2203	1315.1499	NM_021335	0.007	-1.08	-1.11	***Snrpb2***	U2 small nuclear ribonucleoprotein B	0000398 // mRNA splicing, via spliceosome // inferred from electronic annotation///0006397 // mRNA processing // inferred from e
427.63025	444.12756	414.9527	370.8171	NM_025594	0.006	-1.03	-1.12	***Zmat2***	zinc finger, matrin type 2	0003676 // nucleic acid binding // inferred from electronic annotation///0003677 // DNA binding // inferred from electronic annota
17.193987	19.43262	19.53326	17.419245	NR_045877	0.008	1.14	-1.12	***Gata5os***	GATA binding protein 5, opposite strand	
225.5598	266.09833	315.0299	279.24472	NM_019519///XM_0	0.007	1.40	-1.13	***Rabggta***	Rab geranylgeranyl transferase, a subunit	0018342 // protein prenylation // inferred from electronic annotation///0018344 // protein geranylgeranylation // inferred from mu
14.291265	19.655941	21.588547	18.948	NM_001159424///NI	0.010	1.51	-1.14	***Il12a***	interleukin 12a	0001916 // positive regulation of T cell mediated cytotoxicity // not recorded///0002860 // positive regulation of natural killer cell n
799.7805	908.8924	935.824	820.33746	NM_023239	0.010	1.17	-1.14	***Ndnl2***	necdin-like 2	0006281 // DNA repair // inferred from electronic annotation///0006310 // DNA recombination // inferred from electronic annotati
211.4966	269.9397	220.9002	193.18787	NM_025454///XM_0	0.001	1.04	-1.14	***Ing5***	inhibitor of growth family, member 5	0006260 // DNA replication // not recorded///0006351 // transcription, DNA-templated // inferred from electronic annotation///00
128.65659	142.42221	133.93057	114.52638	XR_376401///XR_376	0.008	1.04	-1.17	***Junos***	jun proto-oncogene, opposite strand	
342.90976	358.30432	377.9458	322.66403	NM_013770	0.002	1.10	-1.17	***Slc25a10***	solute carrier family 25 (mitochondrial carrier, dicarboxylate transporter),	0006810 // transport // inferred from electronic annotation///0006817 // phosphate ion transport // not recorded///0006839 // mi
347.19327	325.31973	372.49265	316.3745	NM_001005419	0.000	1.07	-1.18	***Ado***	2-aminoethanethiol (cysteamine) dioxygenase	0055114 // oxidation-reduction process // inferred from electronic annotation
18.54299	29.746069	22.433779	18.942703	NM_010870///XM_0	0.006	1.21	-1.18	***Naip5***	NLR family, apoptosis inhibitory protein 5	0002376 // immune system process // inferred from electronic annotation///0006915 // apoptotic process // inferred from electror
24.221119	24.03557	28.38428	23.953274	NM_001145806///NI	0.008	1.17	-1.18	***Capn8***	calpain 8	0006508 // proteolysis // not recorded
292.51727	327.10156	387.7769	327.12836	NM_001190830///NI	0.009	1.33	-1.19	***Insl3///Jak3***	insulin-like 3///Janus kinase 3	0001556 // oocyte maturation // not recorded///0001701 // in utero embryonic development // inferred from mutant phenotype//
158.55203	167.59285	194.4012	163.53294	NM_023377	0.009	1.23	-1.19	***Stard5***	StAR-related lipid transfer (START) domain containing 5	0006694 // steroid biosynthetic process // inferred from electronic annotation///0006810 // transport // inferred from electronic ar
475.84665	551.0214	585.07983	491.5338	NM_080843	0.001	1.23	-1.19	***Socs4***	suppressor of cytokine signaling 4	0007175 // negative regulation of epidermal growth factor-activated receptor activity // not recorded///0009968 // negative regula
20.327457	25.023666	25.057018	21.020859	NM_015820///XM_0	0.010	1.23	-1.19	***Hs6st3***	heparan sulfate 6-O-sulfotransferase 3	0015015 // heparan sulfate proteoglycan biosynthetic process, enzymatic modification // inferred from direct assay
22.401001	28.488426	26.29509	21.871614	NM_153135///XM_0	0.007	1.17	-1.20	***Unc5d***	unc-5 homolog D (C. elegans)	0006915 // apoptotic process // inferred from electronic annotation///0007165 // signal transduction // inferred from electronic an
105.5201	117.22253	116.45836	95.70669	NM_001033356///NI	0.003	1.10	-1.22	***Ntn5***	netrin 5	0005515 // protein binding // inferred from electronic annotation
258.07562	258.73788	299.8016	245.97234	NM_001083317///NI	0.006	1.16	-1.22	***Slc35a4***	solute carrier family 35, member A4	0006810 // transport // inferred from electronic annotation///0008643 // carbohydrate transport // inferred from electronic annota
216.6314	258.34293	316.55566	259.44574	NM_001243584///NI	0.002	1.46	-1.22	***Mif4gd***	MIF4G domain containing	0006417 // regulation of translation // inferred from electronic annotation///0016070 // RNA metabolic process // inferred from ele
1953.4537	1872.732	1975.305	1613.3383	NM_009120///XM_0	0.006	1.01	-1.22	***Sar1a***	SAR1 gene homolog A (S. cerevisiae)	0006810 // transport // inferred from electronic annotation///0006886 // intracellular protein transport // inferred from electronic
62.444157	63.72583	80.309326	65.53176	NM_198113///XM_0	0.002	1.29	-1.23	***Ssh3***	slingshot homolog 3 (Drosophila)	0006470 // protein dephosphorylation // not recorded///0008064 // regulation of actin polymerization or depolymerization // not r
1214.1118	1153.2646	1335.6378	1070.0314	NM_013876	0.006	1.10	-1.25	***Rnf11***	ring finger protein 11	0006511 // ubiquitin-dependent protein catabolic process // inferred from physical interaction///0042787 // protein ubiquitination
1433.024	1562.6948	1933.818	1543.865	NM_144846///XM_0	0.006	1.35	-1.25	***Fam49b***	family with sequence similarity 49, member B	0005515 // protein binding // inferred from physical interaction
152.43263	243.70963	265.21783	211.09831	NM_010877///XM_0	0.005	1.74	-1.26	***Ncf2***	neutrophil cytosolic factor 2	0006742 // NADP catabolic process // not recorded///0006801 // superoxide metabolic process // not recorded///0007568 // aging
204.48326	260.37762	231.50429	183.26749	NM_001136088///NI	0.008	1.13	-1.26	***Sh3bp2***	SH3-domain binding protein 2	0005515 // protein binding // inferred from physical interaction///0005543 // phospholipid binding // inferred from electronic anno
147.68709	184.66304	205.35013	162.22568	NM_001285785///NI	0.009	1.39	-1.27	***Arhgap9***	Rho GTPase activating protein 9	0007165 // signal transduction // inferred from electronic annotation///0043547 // positive regulation of GTPase activity // not recc
1136.5819	1152.1871	1160.7415	915.9419	NM_173784	0.001	1.02	-1.27	***Ubtd2***	ubiquitin domain containing 2	0005634 // nucleus // inferred from electronic annotation///0005737 // cytoplasm // inferred from electronic annotation
102.49373	121.98715	126.803825	99.954254	NM_001005508	0.005	1.24	-1.27	***Arhgap30***	Rho GTPase activating protein 30	0007165 // signal transduction // inferred from electronic annotation///0043547 // positive regulation of GTPase activity // inferred
28.131695	31.34334	35.37751	27.84789	NM_054055///XM_0	0.003	1.26	-1.27	***Slc13a3***	solute carrier family 13 (sodium-dependent dicarboxylate transporter), m	0006810 // transport // inferred from electronic annotation///0006811 // ion transport // inferred from electronic annotation///00
49.08609	51.260216	64.956535	51.068718	NM_001018079	0.003	1.32	-1.27	***Lce3f***	late cornified envelope 3F	0008544 // epidermis development // inferred from electronic annotation
124.02399	143.71835	169.32361	132.5389	NM_153800///XM_0	0.010	1.37	-1.28	***Arhgap22***	Rho GTPase activating protein 22	0001525 // angiogenesis // inferred from electronic annotation///0006351 // transcription, DNA-templated // inferred from electro
109.933014	124.74854	174.76006	135.46278	NM_013843	0.009	1.59	-1.29	***Zfp53***	zinc finger protein 53	0006355 // regulation of transcription, DNA-templated // not recorded
800.1221	781.7499	1035.5688	800.20197	NM_001081356///NI	0.007	1.29	-1.29	***Vma21***	VMA21 vacuolar H+-ATPase homolog (S. cerevisiae)	0070072 // vacuolar proton-transporting V-type ATPase complex assembly // inferred from sequence or structural similarity
53.829895	59.38179	61.701645	47.352577	NM_175443///XM_0	0.004	1.15	-1.30	***Etnk2***	ethanolamine kinase 2	0001701 // in utero embryonic development // inferred from mutant phenotype///0001890 // placenta development // inferred frc
99.19818	105.454865	125.31466	96.150444	NM_001081374///XI	0.008	1.26	-1.30	***Prss36***	protease, serine 36	0006508 // proteolysis // inferred by curator///0006508 // proteolysis // not recorded
16.634396	17.573605	20.408335	15.599053	NM_001001319///NI	0.000	1.23	-1.31	***Gm13102///Prc***	predicted gene 13102///preferentially expressed antigen in melanoma like 4	
20.274952	25.744766	22.64833	17.194038	NM_175026	0.006	1.12	-1.32	***Pyhin1***	pyrin and HIN domain family, member 1	0035458 // cellular response to interferon-beta // inferred from direct assay
91.83943	121.43089	125.95235	94.717766	NM_001033711///NI	0.010	1.37	-1.33	***Evi2a***	ecotropic viral integration site 2a	0005737 // cytoplasm // inferred from electronic annotation///0016020 // membrane // inferred from electronic annotation///001
229.71281	321.37045	328.6588	245.776	NM_011401///XM_0	0.008	1.43	-1.34	***Slc2a3***	solute carrier family 2 (facilitated glucose transporter), member 3	0006810 // transport // inferred from electronic annotation///0008643 // carbohydrate transport // inferred from electronic annota
29.438747	36.22453	40.267338	29.710863	NM_020576///XM_0	0.001	1.37	-1.36	***Psors1c2***	psoriasis susceptibility 1 candidate 2 (human)	0005576 // extracellular region // inferred from electronic annotation
48.55573	45.167015	57.52486	42.14403	NM_001190325	0.009	1.18	-1.36	***Igll1***	immunoglobulin lambda-like polypeptide 1	0005515 // protein binding // inferred from physical interaction
155.60416	206.79973	203.70131	149.22815	NM_001033308	0.007	1.31	-1.37	***Themis2***	thymocyte selection associated family member 2	0002376 // immune system process // inferred from electronic annotation///0006954 // inflammatory response // inferred from ele
562.46075	777.54803	1024.3243	746.3881	NM_010686///XM_0	0.006	1.82	-1.37	***Laptm5***	lysosomal-associated protein transmembrane 5	0006810 // transport // inferred from electronic annotation
37.38658	50.13584	44.919537	32.450844	NM_013486///XM_0	0.003	1.20	-1.38	***Cd2***	CD2 antigen	0007155 // cell adhesion // inferred from electronic annotation///0016337 // single organismal cell-cell adhesion // not recorded///
117.40022	156.69054	149.04863	106.81548	NM_001146022///XI	0.007	1.27	-1.40	***Wdfy4***	WD repeat and FYVE domain containing 4	0005488 // binding // inferred from electronic annotation///0005515 // protein binding // inferred from electronic annotation
275.38635	372.4274	435.80994	310.31442	NM_009151///XM_0	0.002	1.58	-1.40	***Selplg***	selectin, platelet (p-selectin) ligand	0007155 // cell adhesion // inferred from electronic annotation///0050901 // leukocyte tethering or rolling // inferred from mutant
255.70065	300.62537	361.25	257.1698	NM_153795///XM_0	0.007	1.41	-1.40	***Fermt3***	fermitin family homolog 3 (Drosophila)	0007155 // cell adhesion // inferred from electronic annotation///0007159 // leukocyte cell-cell adhesion // inferred from mutant p
919.72174	2061.3145	3090.1047	2153.654	NM_001082543///NI	0.009	3.36	-1.43	***BC100530///Stf***	cDNA sequence BC100530///stefin A1	0010466 // negative regulation of peptidase activity // inferred from electronic annotation///0010951 // negative regulation of end
118.33227	132.01094	175.2786	121.78282	NM_010090	0.007	1.48	-1.44	***Dusp2***	dual specificity phosphatase 2	0000188 // inactivation of MAPK activity // ---///0000188 // inactivation of MAPK activity // inferred from electronic annotation///0
263.9472	379.15244	384.85242	264.865	NM_008518	0.005	1.46	-1.45	***Ltb***	lymphotoxin B	0006955 // immune response // inferred from electronic annotation///0010467 // gene expression // inferred from mutant phenoty
30.681562	39.70455	66.29107	45.3258	NM_007819	0.005	2.16	-1.46	***Cyp3a13***	cytochrome P450, family 3, subfamily a, polypeptide 13	0010628 // positive regulation of gene expression // inferred from mutant phenotype///0055114 // oxidation-reduction process // i
175.63327	255.236	369.96017	252.5512	NM_007780///XM_0	0.003	2.11	-1.46	***Csf2rb///Csf2rb***	colony stimulating factor 2 receptor, beta, low-affinity (granulocyte-macro	0019221 // cytokine-mediated signaling pathway // inferred from electronic annotation
277.296	333.89667	497.68277	339.13388	NM_010188///XM_0	0.007	1.79	-1.47	***Fcgr3***	Fc receptor, IgG, low affinity III	0001788 // antibody-dependent cellular cytotoxicity // inferred from mutant phenotype///0001798 // positive regulation of type IIa
76.27075	102.16701	115.48816	77.74376	NM_021407///XM_0	0.010	1.51	-1.49	***Trem3***	triggering receptor expressed on myeloid cells 3	0002374 // cytokine secretion involved in immune response // inferred from genetic interaction///0016477 // cell migration // infer
96.048645	121.56108	127.45082	85.46866	NM_026976	0.005	1.33	-1.49	***Faim3***	Fas apoptotic inhibitory molecule 3	0002376 // immune system process // inferred from electronic annotation
637.93494	906.65656	1170.056	784.0508	NM_009663	0.003	1.83	-1.49	***Alox5ap***	arachidonate 5-lipoxygenase activating protein	0002540 // leukotriene production involved in inflammatory response // inferred from mutant phenotype///0002675 // positive reg
51.4766	86.80862	89.64352	59.103527	NM_013542	0.008	1.74	-1.52	***Gzmb***	granzyme B	0001913 // T cell mediated cytotoxicity // inferred from mutant phenotype///0006508 // proteolysis // not recorded///0006915 // a
158.12302	194.08328	215.21947	141.88217	NM_153505///XM_0	0.009	1.36	-1.52	***Nckap1l***	NCK associated protein 1 like	0001782 // B cell homeostasis // inferred from mutant phenotype///0002262 // myeloid cell homeostasis // inferred from mutant p
1960.5182	3594.696	5147.6445	3387.709	NM_001281852///NI	0.008	2.63	-1.52	***S100a9***	S100 calcium binding protein A9 (calgranulin B)	0002376 // immune system process // inferred from electronic annotation///0002523 // leukocyte migration involved in inflammato
27.371424	31.08582	32.58431	21.030909	NR_045694	0.003	1.19	-1.55	***Gm4961***	kinesin superfamily protein 2C pseudogene	
147.19183	164.36505	233.90303	150.5114	NM_011827	0.006	1.59	-1.55	***Hcst***	hematopoietic cell signal transducer	0014068 // positive regulation of phosphatidylinositol 3-kinase signaling // not recorded
41.177948	50.73057	81.90617	52.200413	NM_001252472///NI	0.006	1.99	-1.57	***Cd84***	CD84 antigen	0007155 // cell adhesion // inferred from electronic annotation
118.95557	138.45306	201.20317	127.144226	NM_173014///XM_0	0.007	1.69	-1.58	***Lpcat2***	lysophosphatidylcholine acyltransferase 2	0006629 // lipid metabolic process // inferred from electronic annotation///0006644 // phospholipid metabolic process // inferred f
286.73444	321.73712	471.6088	296.5702	NM_001113553///NI	0.009	1.64	-1.59	***Irak2***	interleukin-1 receptor-associated kinase 2	0001959 // regulation of cytokine-mediated signaling pathway // not recorded///0006468 // protein phosphorylation // not recorde
215.09772	240.7128	414.07697	259.1528	NM_001171000///NI	0.007	1.93	-1.60	***Ahcyl2***	S-adenosylhomocysteine hydrolase-like 2	0006730 // one-carbon metabolic process // inferred from electronic annotation///0055114 // oxidation-reduction process // inferr
192.43607	252.32658	429.29846	267.9737	NM_019549	0.006	2.23	-1.60	***Plek***	pleckstrin	0002244 // hematopoietic progenitor cell differentiation // inferred from electronic annotation///0002576 // platelet degranulation
76.707504	91.36135	97.80498	59.934425	NM_013762///XM_0	0.002	1.28	-1.63	***Gm12816///Gn***	predicted gene 12816///predicted gene 5879///ribosomal protein L3	0006412 // translation // inferred from electronic annotation///0071353 // cellular response to interleukin-4 // inferred from direct
343.07303	441.70917	643.3556	388.76373	NM_011408	0.006	1.88	-1.65	***Slfn2***	schlafen 2	0008285 // negative regulation of cell proliferation // inferred from direct assay
46.61176	50.47808	58.33073	35.23046	NM_011407	0.001	1.25	-1.66	***Slfn1***	schlafen 1	0000122 // negative regulation of transcription from RNA polymerase II promoter // inferred from direct assay///0001933 // negativ
151.59128	139.02855	217.28122	130.90611	NM_144549	0.001	1.43	-1.66	***Trib1***	tribbles homolog 1 (Drosophila)	0006468 // protein phosphorylation // traceable author statement///0006469 // negative regulation of protein kinase activity // no
112.01277	148.76367	287.8654	172.58711	NM_001033149///XI	0.007	2.57	-1.67	***Ttc9***	tetratricopeptide repeat domain 9	0000413 // protein peptidyl-prolyl isomerization // ---///0018208 // peptidyl-proline modification // not recorded///0061077 // cha
163.26695	178.76898	270.7138	162.25035	NM_145133///XM_0	0.005	1.66	-1.67	***Tifa***	TRAF-interacting protein with forkhead-associated domain	0007249 // I-kappaB kinase/NF-kappaB signaling // inferred from direct assay
273.3637	374.30783	457.0455	273.1511	NM_008677///XM_0	0.003	1.67	-1.67	***Ncf4***	neutrophil cytosolic factor 4	0007154 // cell communication // inferred from electronic annotation///0043085 // positive regulation of catalytic activity // not re
142.47269	176.45187	256.33868	149.9394	NM_008339	0.007	1.80	-1.71	***Cd79b***	CD79B antigen	0002376 // immune system process // inferred from electronic annotation///0007166 // cell surface receptor signaling pathway // i
99.29701	101.34434	185.19426	108.16444	NM_024215	0.009	1.87	-1.71	***Zfp593***	zinc finger protein 593	0006351 // transcription, DNA-templated // inferred from electronic annotation///0006355 // regulation of transcription, DNA-temp
3770.4695	7312.7573	9725.461	5675.529	NM_013650	0.005	2.58	-1.71	***S100a8***	S100 calcium binding protein A8 (calgranulin A)	0002376 // immune system process // inferred from electronic annotation///0002523 // leukocyte migration involved in inflammato
147.31975	215.15489	416.02606	241.93962	NM_183249	0.002	2.82	-1.72	***Wfdc21***	WAP four-disulfide core domain 21	0010466 // negative regulation of peptidase activity // inferred from electronic annotation///0032496 // response to lipopolysaccha
120.68527	151.70645	199.8005	115.298	NM_009515	0.006	1.66	-1.73	***Was***	Wiskott-Aldrich syndrome homolog (human)	0006955 // immune response // not recorded///0007015 // actin filament organization // inferred from electronic annotation///00
76.25336	88.96276	187.3568	105.36641	NM_198664///XM_0	0.007	2.46	-1.78	***Tbc1d2***	TBC1 domain family, member 2	0032313 // regulation of Rab GTPase activity // inferred from electronic annotation///0032851 // positive regulation of Rab GTPase
133.30998	193.00711	299.1578	166.75372	NM_023044	0.004	2.24	-1.79	***Slc15a3***	solute carrier family 15, member 3	0006810 // transport // inferred from electronic annotation///0006857 // oligopeptide transport // inferred from electronic annota
133.55536	155.66481	195.62573	104.50443	NM_027222	0.003	1.46	-1.87	***Mzb1***	marginal zone B and B1 cell-specific protein 1	0002642 // positive regulation of immunoglobulin biosynthetic process // inferred from mutant phenotype///0008284 // positive re
304.89157	487.05194	731.54846	390.23648	NM_001002898///NI	0.008	2.40	-1.87	***LOC100038947/***	signal-regulatory protein beta 1-like///signal-regulatory protein beta 1A//	0035556 // intracellular signal transduction // inferred from direct assay///0050766 // positive regulation of phagocytosis // inferre
65.75871	79.92206	106.8092	56.641823	NM_001037719///NI	0.001	1.62	-1.89	***Btla***	B and T lymphocyte associated	0002376 // immune system process // inferred from electronic annotation///0002768 // immune response-regulating cell surface re
634.97644	727.59283	1426.2726	714.0382	NM_181444///XM_0	0.010	2.25	-2.00	***Gprc5a***	G protein-coupled receptor, family C, group 5, member A	0007165 // signal transduction // inferred from electronic annotation///0007186 // G-protein coupled receptor signaling pathway //
172.69827	187.53242	782.9005	345.8944	NM_011338	0.008	4.53	-2.26	***Ccl9***	chemokine (C-C motif) ligand 9	0006935 // chemotaxis // inferred from electronic annotation///0006955 // immune response // inferred from electronic annotatio
534.7251	676.7039	1280.9734	565.8365	NM_007651	0.003	2.40	-2.26	***Cd53***	CD53 antigen	0016337 // single organismal cell-cell adhesion // inferred from direct assay///0045661 // regulation of myoblast differentiation // i
895.12146	1246.6874	1695.3186	706.67444	NM_013706	0.009	1.89	-2.40	***Cd52***	CD52 antigen	0005886 // plasma membrane // inferred from electronic annotation///0016020 // membrane // inferred from electronic annotatio

[Nrf2-KO, Air, PBS](r	[Nrf2-KO, Air, SFN](	[Nrf2-KO, O2, PBS](	[Nrf2-KO, O2, SFN](	RefSeq Transcript ID	p (Prenatal Treatment-Exposure)	FC [Air-PBS:O2-PBS]	FC [O2 PBS:SFN]	Gene Symbol	Gene Title	Gene Ontology
102.074425	143.36925	415.62582	165.67465	NM_001081205	0.009	4.07	-2.51	***Nipal1***	NIPA-like domain containing 1	0006810 // transport // inferred from electronic annotation///0006811 // ion transport // inferred from electronic annotation///00
558.2761	815.70386	2517.2708	907.5686	NM_020008	0.001	4.51	-2.77	***Clec7a***	C-type lectin domain family 7, member a	0001878 // response to yeast // inferred from direct assay///0001879 // detection of yeast // inferred from direct assay///0002221

Table S5. Prenatal sulforaphane-modulated genes in *Nrf2*$^{+/+}$ placenta (T Test unpaired p-value P <= 0.05).

>=1.5-fold 708 genes

RIKEN cDNA and predicted genes excluded. Fold Change (FC): Blue (-) - fold lower in SFN than PBS, Red - fold higher in SFN than PBS.

[ICR, PBS](	[ICR, SUL](	p	RefSeq Transcript ID	FC [PBS:SFN]	Gene Symbol	Gene Title	Gene Ontology Biological Process
35.40988	266.3408	0.005	NM_025288	7.33	***Stfa3***	stefin A3	0004869 // cysteine-type endopeptidase inhibitor activity // inferred from electronic annotation
112.3694	554.5562	0.009	NM_001082543///NI	4.56	***BC100530///Stfa1***	cDNA sequence BC100530///stefin A1	0004869 // cysteine-type endopeptidase inhibitor activity // inferred from electronic annotation
94.05842	412.6405	0.011	NM_026516	4.33	***Tmem178***	transmembrane protein 178	0005198 // structural molecule activity // inferred from electronic annotation
24.77076	87.45144	0.042	NM_011670	3.69	***Uchl1***	ubiquitin carboxy-terminal hydrolase L1	0007409 // axonogenesis // inferred from mutant phenotype///0019896 // axon transport of mi
77.77694	255.1708	0.011	NM_144848	3.26	***Eppk1***	epiplakin 1	0005737 // cytoplasm // inferred from direct assay///0005856 // cytoskeleton // inferred from e
43.68089	148.7498	0.040	NM_010441	3.10	***Hmga2***	high mobility group AT-hook 2	0051301 // cell division // inferred from electronic annotation///0007049 // cell cycle // inferrec
11.32679	35.95194	0.012	NM_027137	3.04	***Lce1d***	late cornified envelope 1D	
41.48204	109.7844	0.035	NM_199473	2.92	***Col8a2***	collagen, type VIII, alpha 2	0048593 // camera-type eye morphogenesis // inferred from genetic interaction///0007155 // c
211.6307	621.1341	0.018	NM_009209	2.88	***Slc6a2***	solute carrier family 6 (neurotransmitter transporter, noradrenalin), memb	0015844 // monoamine transport // not recorded///0015874 // norepinephrine transport // infe
92.00953	256.2665	0.018	NM_011485	2.87	***Star***	steroidogenic acute regulatory protein	0044255 // cellular lipid metabolic process // inferred from mutant phenotype///0010288 // res
426.3177	1176.62	0.028	NM_007406///NM_0	2.86	***Adcy7***	adenylate cyclase 7	0009190 // cyclic nucleotide biosynthetic process // inferred from electronic annotation///0006
95.5387	257.2281	0.009	NM_001081139	2.80	***Ankrd35***	ankyrin repeat domain 35	
349.9298	911.0887	0.028	NM_001081362	2.79	***Trrap***	transformation/transcription domain-associated protein	0016568 // chromatin modification // inferred from electronic annotation///0006355 // regulati
44.18663	110.3886	0.046	NM_008508	2.77	***Lor***	loricrin///Loricrin, mRNA (cDNA clone IMAGE:4190845)	0030216 // keratinocyte differentiation // inferred from electronic annotation///0031424 // ker
357.6803	1011.711	0.014	NM_033314	2.76	***Slco2a1***	solute carrier organic anion transporter family, member 2a1///Solute carri	0015732 // prostaglandin transport // inferred from direct assay///0006810 // transport // infer
209.9585	566.8061	0.012	NM_177386	2.75	***Sfmbt2***	Scm-like with four mbt domains 2	0045449 // regulation of transcription // inferred from electronic annotation
211.0596	604.8945	0.018	XM_001476334///XI	2.75	***100042151///1000432***	predicted gene, 100039204///hypothetical protein LOC100047648///CWC:	0008380 // RNA splicing // inferred from electronic annotation///0006397 // mRNA processing /
24.54087	68.3346	0.024	NM_025984	2.71	***Lce1a1***	late cornified envelope 1A1	
489.1765	1312.866	0.019	NM_007984	2.66	***Fscn1***	fascin homolog 1, actin bundling protein (Strongylocentrotus purpuratus)	0003779 // actin binding // inferred from electronic annotation///0003779 // actin binding // inf
196.8718	467.3081	0.044	NM_001081098	2.59	***Zfp362***	zinc finger protein 362	0008270 // zinc ion binding // inferred from electronic annotation///0003676 // nucleic acid bin
930.6491	2130.405	0.045	NM_001146268///NI	2.57	***Pdgfrb***	platelet derived growth factor receptor, beta polypeptide	0008284 // positive regulation of cell proliferation // inferred from electronic annotation///0006
118.9973	282.2134	0.022	NM_001109661///NI	2.52	***Bach2***	BTB and CNC homology 2	0006355 // regulation of transcription, DNA-dependent // inferred from direct assay///0006355
161.0767	377.9266	0.030	NM_007872///NM_1	2.51	***Dnmt3a***	DNA methyltransferase 3A	0007283 // spermatogenesis // inferred from mutant phenotype///0006346 // methylation-dep
74.91989	179.8726	0.008	NM_010101	2.51	***S1pr3***	sphingosine-1-phosphate receptor 3	0007165 // signal transduction // inferred from electronic annotation///0032651 // regulation o
61.43885	149.0868	0.023	NM_001081417	2.51	***Chd7***	chromodomain helicase DNA binding protein 7	0030540 // female genitalia development // inferred from mutant phenotype///0042472 // inne
188.5176	481.9748	0.016	XM_001476334///XI	2.50	***100042151///1000432***	predicted gene, 100039204///hypothetical protein LOC100047648///CWC:	0008380 // RNA splicing // inferred from electronic annotation///0006397 // mRNA processing /
282.5281	640.1924	0.038	NM_001081109	2.48	***Lmtk2***	lemur tyrosine kinase 2	0007399 // nervous system development // not recorded///0048011 // nerve growth factor rec
23.55192	57.13178	0.002	NM_009144	2.44	***Sfrp2***	secreted frizzled-related protein 2	0007275 // multicellular organismal development // inferred from electronic annotation///0030
174.2613	389.5284	0.037	NM_010800	2.42	***Bhlha15***	basic helix-loop-helix family, member a15	0048469 // cell maturation // inferred from mutant phenotype///0006851 // mitochondrial calc
313.2889	726.1153	0.016	NM_027373	2.40	***Afap1***	actin filament associated protein 1	0009966 // regulation of signal transduction // not recordec
199.5643	458.1648	0.011	NM_001081279	2.37	***Mfhas1***	malignant fibrous histiocytoma amplified sequence 1	0005515 // protein binding // inferred from electronic annotation
44.1906	106.9033	0.044	NM_008725	2.35	***Nppa***	natriuretic peptide precursor type A	0050880 // regulation of blood vessel size // inferred from electronic annotation///0008217 // r
422.2918	971.3632	0.010	NM_175451	2.31	***Ckap4***	cytoskeleton-associated protein 4	0005515 // protein binding // not recorded
50.9815	123.5872	0.046	NM_021285///NM_0	2.28	***Myl1***	myosin, light polypeptide 1	0003774 // motor activity // inferred from electronic annotation///0005509 // calcium ion bindi
417.8607	906.8631	0.040	NM_175260	2.25	***Myh10***	myosin, heavy polypeptide 10, non-muscle///Myosin, heavy polypeptide 1(	0021678 // third ventricle development // inferred from mutant phenotype///0007097 // nucle
480.0858	1007.058	0.031	NM_001083935///NI	2.25	***Pth1r***	parathyroid hormone 1 receptor	0002076 // osteoblast development // inferred from mutant phenotype///0048469 // cell matu
72.74856	153.3457	0.048	XM_978355///XM_9(	2.23	***Klhl29***	kelch-like 29 (Drosophila)	0005515 // protein binding // inferred from electronic annotation
118.6122	260.5463	0.004	NM_011941	2.23	***Mapkbp1***	mitogen-activated protein kinase binding protein 1	0007256 // activation of JNKK activity // inferred from direct assay
161.7701	366.8025	0.012	NM_133833///NM_1	2.22	***Dst***	dystonin	0031122 // cytoplasmic microtubule organization // inferred from mutant phenotype///0008090
146.2006	316.1447	0.002	NM_001160268///NI	2.21	***Plekha6***	pleckstrin homology domain containing, family A member 6	
133.2459	271.5109	0.046	NM_011214///NM_0	2.19	***Ptpru***	protein tyrosine phosphatase, receptor type, U	0034109 // homotypic cell-cell adhesion // inferred from direct assay///0016311 // dephosphor
205.0009	421.9397	0.028	NM_001025379	2.19	***Sema3g***	sema domain, immunoglobulin domain (Ig), short basic domain, secreted, (	0007275 // multicellular organismal development // inferred from electronic annotation
53.65535	115.8217	0.004	NM_011976	2.19	***Sema4g***	sema domain, immunoglobulin domain (Ig), transmembrane domain (TM)	0007275 // multicellular organismal development // inferred from electronic annotation///0030
1618.126	3295.512	0.039	NM_009932	2.17	***Col4a2***	collagen, type IV, alpha 2	0016525 // negative regulation of angiogenesis // inferred from sequence or structural similarity
253.4368	521.8038	0.036	NM_015734	2.15	***Col5a1***	collagen, type V, alpha 1	0007155 // cell adhesion // inferred from electronic annotation///0051128 // regulation of cellu
423.3071	888.5441	0.037	NM_016710	2.15	***Nsbp1***	nucleosome binding protein 1	0006355 // regulation of transcription, DNA-dependent // inferred from electronic annotation//
1094.84	2244.811	0.034	XM_621314///NM_0:	2.14	***Dsp***	desmoplakin	0016323 // basolateral plasma membrane // inferred from direct assay///0030057 // desmosom
51.63446	106.1434	0.020	NM_177047	2.14	***Auts2***	Autism susceptibility candidate 2, mRNA (cDNA clone IMAGE:5347232)///autism susceptibility candidate 2	
380.7839	772.0258	0.023	NM_001039677	2.12	***Slc30a2***	solute carrier family 30 (zinc transporter), member 2	0006812 // cation transport // inferred from electronic annotation
963.4966	1955.262	0.029	NM_001113488///NI	2.11	***9-Sep***	septin 9	0051301 // cell division // inferred from electronic annotation///0007049 // cell cycle // inferrec
59.62911	120.9713	0.014	NM_178660	2.11	***Rbms3***	RNA binding motif, single stranded interacting protein	0003723 // RNA binding // inferred from electronic annotation///0000166 // nucleotide binding
177.435	377.6742	0.018	NM_173036	2.11	***Gpr97***	G protein-coupled receptor 97	0007165 // signal transduction // inferred from electronic annotation///0007218 // neuropeptic
161.2454	318.297	0.044	NM_172493///NM_0	2.11	***Diap2***	diaphanous homolog 2 (Drosophila)	0030036 // actin cytoskeleton organization // inferred from electronic annotation///0007275 //
545.8066	1091.028	0.034	NM_009059	2.11	***Rgl2***	ral guanine nucleotide dissociation stimulator-like 2	0007264 // small GTPase mediated signal transduction // inferred from electronic annotation///
100.7942	195.8783	0.048	NM_021366	2.10	***Klf13***	Kruppel-like factor 13 (Klf13), mRNA///Kruppel-like factor 13	0006366 // transcription from RNA polymerase II promoter // inferred from sequence or structu
18.04456	37.9812	0.010	XM_001474909///XI	2.10	***Tnik***	TRAF2 and NCK interacting kinase, mRNA (cDNA clone MGC:183916 IMAGI	0006950 // response to stress // inferred from sequence or structural similarity///0007243 // pr
191.2534	402.5042	0.018	NM_153396///XM_9	2.09	***Mical3***	microtubule associated monoxygenase, calponin and LIM domain containi	0008152 // metabolic process // inferred from electronic annotation

[ICR, PBS](	[ICR, SUL](p		RefSeq Transcript ID	FC [PBS:SFN]	Gene Symbol	Gene Title	Gene Ontology Biological Process
118.5959	240.8447	0.008	NM_001040398	2.07	***Setd1b***	SET domain containing 1B	0016568 // chromatin modification // inferred from electronic annotation///0006355 // regulati
291.136	591.001	0.007	NM_175155	2.07	***Sash1***	SAM and SH3 domain containing 1	0007049 // cell cycle // inferred from electronic annotation///0045786 // negative regulation of
626.7794	1221.173	0.042	NM_001040459	2.06	***Shroom4***	shroom family member 4	0007275 // multicellular organismal development // inferred from electronic annotation///0007
176.0527	349.1648	0.017	NM_172457	2.06	***Mobkl2a***	MOB1, Mps One Binder kinase activator-like 2A (yeast)	0005515 // protein binding // inferred from electronic annotation///0046872 // metal ion bindi
9.943523	20.63494	0.009	NR_001586///NM_0(	2.06	***Speer1-ps1///EG54572***	spermatogenesis associated glutamate (E)-rich protein 1, pseudogene 1///predicted gene, EG545728///predicted gene, EG62389E	
132.4335	282.453	0.041	NM_029667	2.06	***Lce1i***	late cornified envelope 1I	
521.2607	1072.452	0.012	NM_001033380	2.05	***Itpripl2***	inositol 1,4,5-triphosphate receptor interacting protein-like 2	0016021 // integral to membrane // inferred from electronic annotation///0016020 // membrar
174.54	345.2822	0.028	NM_015830	2.05	***Solh***	Mus musculus, clone IMAGE:4241884///small optic lobes homolog (Drosoj	0006508 // proteolysis // inferred from electronic annotation
169.1234	323.1221	0.044	NM_001113573///NM	2.05	***Brd3***	bromodomain containing 3	0005634 // nucleus // inferred from electronic annotation
447.3822	873.7873	0.042	NM_172397	2.03	***Limd2***	LIM domain containing 2	0046872 // metal ion binding // inferred from electronic annotation///0008270 // zinc ion bindi
972.4028	1925.166	0.009	NM_025294	2.02	***Gtlf3b***	gene trap locus F3b	
169.0003	332.5705	0.017	NM_010683	2.02	***Lamc1***	laminin, gamma 1	0022617 // extracellular matrix disassembly // inferred from electronic annotation///0016477 //
337.8081	641.7703	0.037	NM_020508///XM_0(	2.02	***Brd4***	bromodomain containing 4	0043388 // positive regulation of DNA binding // inferred from direct assay///0006468 // protei
230.9898	458.0944	0.034	NM_013807	2.02	***Plk3***	polo-like kinase 3 (Drosophila)	0007049 // cell cycle // inferred from electronic annotation///0006468 // protein amino acid ph
46.18642	93.42761	0.026		2.01	***D15Wsu126e***	DNA segment, Chr 15, Wayne State University 126, expressed	
145.1635	285.2369	0.038	NM_201385///NM_2	2.00	***LOC671535///Plec1***	similar to Plec1 protein///plectin 1	0003779 // actin binding // inferred from electronic annotation///0003950 // NAD+ ADP-ribosyl
376.4715	746.1816	0.033	NM_009362	2.00	***Tff1***	trefoil factor 1	0030154 // cell differentiation // inferred from mutant phenotype///0009611 // response to wo
238.0372	449.8092	0.037	NM_172827	2.00	***Lnpep***	leucyl/cystinyl aminopeptidase	0030147 // natriuresis // inferred from electronic annotation///0048169 // regulation of long-te
157.7969	301.0559	0.034	NM_001042623///NM	1.99	***Phc1***	polyhomeotic-like 1 (Drosophila)	0007275 // multicellular organismal development // inferred from electronic annotation
232.68	443.4164	0.026	NM_001081118	1.99	***Phrf1***	PHD and ring finger domains 1	0006366 // transcription from RNA polymerase II promoter // not recorded///0007242 // intrace
164.3662	321.0152	0.029	NM_177409///NM_1	1.99	***Tram2***	translocating chain-associating membrane protein 2	0065002 // intracellular protein transmembrane transport // inferred from electronic annotatio
2025.212	3853.207	0.034	NM_010137///XM_0(	1.98	***LOC100048537///Epas***	endothelial PAS domain protein 1///similar to Endothelial PAS domain prot	0043129 // surfactant homeostasis // inferred from mutant phenotype///0007275 // multicellul
133.997	252.9343	0.026	NM_001024955///NM	1.97	***Pik3r1***	phosphatidylinositol 3-kinase, regulatory subunit, polypeptide 1 (p85 alpha	0007165 // signal transduction // inferred from electronic annotation///0048009 // insulin-like g
66.61494	128.0928	0.027	NM_199068	1.97	***Foxk1***	forkhead box K1	0007275 // multicellular organismal development // inferred from electronic annotation///0045
722.7357	1335.036	0.049	NM_013605	1.97	***Muc1***	mucin 1, transmembrane	0016324 // apical plasma membrane // inferred from direct assay///0005634 // nucleus // infer
134.2631	251.8326	0.040	NM_172882	1.96	***Wdfy3***	WD repeat and FYVE domain containing 3	0016757 // transferase activity, transferring glycosyl groups // inferred from electronic annotati
188.671	360.7327	0.017	NM_016808///NM_1	1.96	***Usp2***	ubiquitin specific peptidase 2	0019941 // modification-dependent protein catabolic process // inferred from electronic annota
62.10377	118.003	0.020	NM_027011	1.96	***Krt5***	keratin 5	0005198 // structural molecule activity // inferred from electronic annotation///0005515 // prot
198.6434	373.9885	0.030	NM_019472	1.95	***Myo10***	myosin X	0007165 // signal transduction // inferred from electronic annotation
203.4426	396.5453	0.024	NM_001145836///NM	1.95	***Ralgds***	ral guanine nucleotide dissociation stimulator	0007264 // small GTPase mediated signal transduction // inferred from electronic annotation///
171.969	325.3518	0.016	NM_173760	1.95	***Hisppd1***	histidine acid phosphatase domain containing 1	0006020 // inositol metabolic process // inferred from sequence or structural similarity
574.0403	1082.442	0.032	NM_011597	1.95	***Tjp2***	tight junction protein 2	0005515 // protein binding // inferred from electronic annotation///0005515 // protein binding
668.043	1290.094	0.017	NM_007883	1.94	***Dsg2***	desmoglein 2///Desmoglein 2 (Dsg2), mRNA	0007156 // homophilic cell adhesion // inferred from electronic annotation///0007155 // cell ad
470.3322	870.0803	0.036	NM_011962	1.94	***Plod3***	procollagen-lysine, 2-oxoglutarate 5-dioxygenase 3	0019538 // protein metabolic process // inferred from electronic annotation///0055114 // oxida
37.30195	71.44573	0.012	NM_011256	1.94	***Pitpnm2***	phosphatidylinositol transfer protein, membrane-associated 2	0006810 // transport // inferred from electronic annotation
89.97656	173.5232	0.023	NM_008641///NM_0	1.94	***Mast2***	microtubule associated serine/threonine kinase 2///Microtubule associate	0048515 // spermatid differentiation // inferred from direct assay///0006468 // protein amino a
104.7456	195.4346	0.030	NM_198109	1.93	***Ssh1***	slingshot homolog 1 (Drosophila)	0016311 // dephosphorylation // inferred from electronic annotation///0006470 // protein amir
56.81121	110.8075	0.033	NM_178406	1.92	***Gpr153***	G protein-coupled receptor 153	0007165 // signal transduction // inferred from electronic annotation///0007186 // G-protein cc
193.6991	361.0289	0.021	NM_173379	1.92	***Leprel1***	leprecan-like 1	0019538 // protein metabolic process // inferred from electronic annotation///0055114 // oxida
516.5957	971.8157	0.020	NM_139140	1.92	***Spats2***	spermatogenesis associated, serine-rich 2	0005737 // cytoplasm // inferred from direct assay///0005737 // cytoplasm // inferred from ele
116.0436	219.221	0.024	NM_172424	1.92	***Med13l***	mediator complex subunit 13-like	0006357 // regulation of transcription from RNA polymerase II promoter // inferred from electro
658.4739	1220.883	0.028	NM_175414	1.92	***Tspan9***	tetraspanin 9	0016021 // integral to membrane // inferred from electronic annotation///0016020 // membrar
75.09846	139.9092	0.025	NM_018775	1.91	***Tbc1d8***	TBC1 domain family, member 8	0032313 // regulation of Rab GTPase activity // inferred from electronic annotation
504.3522	951.1658	0.006	NM_133626///NM_0	1.91	***Rrbp1***	ribosome binding protein 1	0065002 // intracellular protein transmembrane transport // inferred from electronic annotatio
275.4002	519.4771	0.037	NM_144925	1.91	***Tnrc6a***	trinucleotide repeat containing 6a	0009267 // cellular response to starvation // inferred from direct assay///0032507 // maintenan
166.5425	311.159	0.010	NM_009686	1.90	***Apbb2***	amyloid beta (A4) precursor protein-binding, family B, member 2	0030308 // negative regulation of cell growth // inferred from direct assay///0030048 // actin fil
221.0302	412.165	0.008	NM_007842	1.90	***Dhx9***	DEAH (Asp-Glu-Ala-His) box polypeptide 9	0034605 // cellular response to heat // inferred from direct assay
113.2601	209.0205	0.021	NM_010221	1.90	***Fkbp10***	FK506 binding protein 10	0006457 // protein folding // inferred from electronic annotation
198.865	373.955	0.036	NM_011866	1.90	***Pde10a***	phosphodiesterase 10A	0045672 // positive regulation of osteoclast differentiation // inferred from mutant phenotype/,
220.3442	413.3039	0.014	NM_001081337	1.89	***Sipa1l2***	signal-induced proliferation-associated 1 like 2	0051056 // regulation of small GTPase mediated signal transduction // inferred from electronic a
104.6065	196.355	0.036	NM_007403	1.89	***Adam8***	a disintegrin and metallopeptidase domain 8	0007229 // integrin-mediated signaling pathway // inferred from electronic annotation///00065
90.01758	164.9848	0.033	NM_011104	1.89	***Prkce***	protein kinase C, epsilon	0007635 // chemosensory behavior // traceable author statement///0007242 // intracellular sig
18.21033	34.43372	0.014	NM_181540	1.89	***Tm6sf2***	transmembrane 6 superfamily member 2	0016021 // integral to membrane // inferred from electronic annotation///0016020 // membrar
239.9004	446.5806	0.037	NM_178661	1.88	***Creb3l2***	cAMP responsive element binding protein 3-like 2	0006986 // response to unfolded protein // inferred from electronic annotation///0006355 // re
2881.238	5221.864	0.020	NM_009931	1.88	***Col4a1***	collagen, type IV, alpha 1	0005201 // extracellular matrix structural constituent // inferred from electronic annotation///0
152.6381	274.8745	0.032	NM_001122676///NM	1.88	***Zcchc2***	zinc finger, CCHC domain containing 2///Zinc finger, CCHC domain containi	0007154 // cell communication // inferred from electronic annotation
1230.772	2225.907	0.024	NM_013676	1.87	***Supt5h***	suppressor of Ty 5 homolog (S. cerevisiae)	0006357 // regulation of transcription from RNA polymerase II promoter // inferred from electro
24.47387	44.5047	0.012	NM_146061	1.87	***Prr5***	proline rich 5 (renal)	0007049 // cell cycle // inferred from electronic annotation///0030335 // positive regulation of
32.2329	59.10203	0.023	NM_173368	1.87	***Chd6***	chromodomain helicase DNA binding protein 6	0006333 // chromatin assembly or disassembly // inferred from electronic annotation
110.2013	203.1034	0.012	NM_146030	1.87	***Plekhh3***	pleckstrin homology domain containing, family H (with MyTH4 domain) me	0007165 // signal transduction // inferred from electronic annotation

[ICR, PBS](	[ICR, SUL](p		RefSeq Transcript ID	FC [PBS:SFN]	Gene Symbol	Gene Title	Gene Ontology Biological Process
447.5739	820.1391	0.025	NM_016747	1.87	***Dlg3***	discs, large homolog 3 (Drosophila)	0005515 // protein binding // inferred from electronic annotation///0005515 // protein binding
106.0816	189.2577	0.033	NM_013495	1.87	***Cpt1a***	carnitine palmitoyltransferase 1a, liver	0042755 // eating behavior // inferred from electronic annotation///0006810 // transport // inf
107.5898	197.2989	0.019	NM_010700	1.87	***Ldlr***	low density lipoprotein receptor	0008202 // steroid metabolic process // inferred from electronic annotation///0008203 // chole
71.08664	127.684	0.036	NM_197940	1.87	***Wipf2***	WAS/WASL interacting protein family, member 2	0003779 // actin binding // inferred from electronic annotation
8715.003	15611.46	0.046	NM_011120	1.86	***Prl7d1***	prolactin family 7, subfamily d, member 1	0043537 // negative regulation of blood vessel endothelial cell migration // inferred from direct
85.80798	152.241	0.039	NM_181072	1.86	***Myo1e***	myosin IE	0006807 // nitrogen compound metabolic process // inferred from mutant phenotype///000182
295.6276	532.4476	0.019	NM_144922///NM_1	1.86	***Hnrnpul1***	heterogeneous nuclear ribonucleoprotein U-like 1	0006355 // regulation of transcription, DNA-dependent // inferred from electronic annotation//
225.7229	409.9263	0.014	NM_010153	1.86	***Erbb3***	v-erb-b2 erythroblastic leukemia viral oncogene homolog 3 (avian)	0043524 // negative regulation of neuron apoptosis // inferred from mutant phenotype///0007
428.554	751.7423	0.046	NM_023598///XM_0	1.86	***LOC100044968///Arid***	similar to modulator recognition factor 2///AT rich interactive domain 5B (	0060021 // palate development // inferred from mutant phenotype///0060325 // face morphog
111.322	208.9804	0.024	XM_001478845///NN	1.85	***5830417I10Rik///LOC6***	similar to Dingo protein isoform 2///RIKEN cDNA 5830417I10 gene	0006355 // regulation of transcription, DNA-dependent // inferred from electronic annotation
322.5857	596.9042	0.011	NM_010501	1.85	***Ifit3***	interferon-induced protein with tetratricopeptide repeats 3	0005488 // binding // inferred from electronic annotation
19.1484	35.43503	0.018	XM_001478962///XN	1.85	***Muc3***	mucin 3, intestinal	0016324 // apical plasma membrane // inferred from direct assay
58.71035	106.2942	0.025	NM_175268///NM_2	1.84	***Fam53b***	family with sequence similarity 53, member B	
307.8058	567.5307	0.024	NM_177461	1.84	***Micall1***	microtubule associated monoxygenase, calponin and LIM domain containi	0005856 // cytoskeleton // inferred from electronic annotation///0005737 // cytoplasm // infer
665.9888	1232.495	0.015	NM_172588	1.84	***Serinc5***	serine incorporator 5	0006658 // phosphatidylserine metabolic process // inferred from sequence or structural similar
1613.667	2866.976	0.046	NM_001093766///NN	1.83	***Myadm***	myeloid-associated differentiation marker	0016021 // integral to membrane // inferred from electronic annotation///0016020 // membrar
35.24829	62.94902	0.011	XM_001476544///XN	1.83	***Ttc28***	tetratricopeptide repeat domain 28	0005488 // binding // inferred from electronic annotation
787.5598	1384.153	0.047	NM_139149	1.82	***Fus***	fusion, derived from t(12;16) malignant liposarcoma (human)	0045944 // positive regulation of transcription from RNA polymerase II promoter // inferred fro
131.4118	236.1165	0.038	NM_080448	1.81	***Srgap3***	SLIT-ROBO Rho GTPase activating protein 3	0007165 // signal transduction // inferred from electronic annotation
79.89884	139.4792	0.025	NM_010513	1.81	***Igf1r***	insulin-like growth factor I receptor	0048009 // insulin-like growth factor receptor signaling pathway // inferred from sequence or st
750.2532	1312.093	0.023	NM_198703	1.81	***Wnk1***	WNK lysine deficient protein kinase 1	0032776 // DNA methylation on cytosine // inferred from electronic annotation///0006468 // pr
67.28961	118.5715	0.030	NM_011929	1.81	***Clcn6***	chloride channel 6	0006821 // chloride transport // inferred from electronic annotation///0006811 // ion transport
58.44442	104.9145	0.022	NM_011345	1.80	***Sele***	selectin, endothelial cell	0007155 // cell adhesion // inferred from electronic annotation
122.1599	212.6065	0.036	NM_001025395///NN	1.80	***Src***	Rous sarcoma oncogene	0006950 // response to stress // inferred from electronic annotation///0018108 // peptidyl-tyro
316.7528	550.055	0.031	NM_011202///NM_0	1.80	***Ptpn11***	protein tyrosine phosphatase, non-receptor type 11	0007409 // axonogenesis // inferred from mutant phenotype///0016311 // dephosphorylation /
324.0155	569.0248	0.011	NM_020006	1.80	***Cdc42ep4***	CDC42 effector protein (Rho GTPase binding) 4	0031274 // positive regulation of pseudopodium assembly // inferred from electronic annotatio
356.1224	635.7925	0.021	NM_178612	1.80	***Cnpy4***	canopy 4 homolog (zebrafish)	0032880 // regulation of protein localization // not recorded
121.4798	210.217	0.026	NM_007997	1.79	***Fdxr***	ferredoxin reductase	0008202 // steroid metabolic process // inferred from electronic annotation///0055114 // oxida
860.3024	1502.002	0.022	NM_030887	1.79	***Jdp2***	Jun dimerization protein 2	0006355 // regulation of transcription, DNA-dependent // inferred from electronic annotation//
197.5953	338.5231	0.040	NM_011385	1.79	***Ski***	ski sarcoma viral oncogene homolog (avian)	0030326 // embryonic limb morphogenesis // inferred from mutant phenotype
134.4079	240.2669	0.033	NM_001017426	1.79	***Kdm6b***	KDM1 lysine (K)-specific demethylase 6B///Jumonji domain containing 3, n	0016568 // chromatin modification // inferred from electronic annotation///0055114 // oxidatic
256.6292	453.266	0.027	NM_022995	1.79	***Pmepa1***	prostate transmembrane protein, androgen induced 1	0005515 // protein binding // inferred from physical interaction
152.4786	261.2643	0.041	NM_001080819	1.79	***Arid1a***	AT rich interactive domain 1A (SWI-like)	0030520 // estrogen receptor signaling pathway // inferred from sequence or structural similarit
128.4421	224.4745	0.035	NM_199307	1.79	***Ece1***	endothelin converting enzyme 1	0001921 // positive regulation of receptor recycling // inferred from electronic annotation///00
66.02708	113.9495	0.027	NM_019763	1.78	***Spen***	SPEN homolog, transcriptional regulator (Drosophila)	0045892 // negative regulation of transcription, DNA-dependent // inferred from direct assay///
24.74597	44.40036	0.031	NM_010167	1.78	***Eya4***	eyes absent 4 homolog (Drosophila)	0007275 // multicellular organismal development // inferred from electronic annotation///0008
30.8225	53.1494	0.031	XM_001003576///XN	1.78	***Rnf157***	ring finger protein 157	0005515 // protein binding // inferred from electronic annotation///0046872 // metal ion bindir
198.9192	353.2621	0.013	NM_009288	1.77	***Stk10***	serine/threonine kinase 10	0006468 // protein amino acid phosphorylation // inferred from electronic annotation
107.5748	181.7082	0.048	XM_001478275///NN	1.77	***Stk11ip///LOC100047***	similar to serine/threonine kinase 11 interacting protein///serine/threonine kinase 11 interacting protein	
76.59496	132.9639	0.022	NM_026599///XM_0	1.77	***Cgnl1***	cingulin-like 1	0003774 // motor activity // inferred from electronic annotation
367.749	643.698	0.011	NM_011207	1.77	***Ptpn3***	protein tyrosine phosphatase, non-receptor type 3	0016311 // dephosphorylation // inferred from electronic annotation///0006470 // protein amir
41.73602	72.87556	0.006	NM_025958	1.76	***Cand2***	cullin-associated and neddylation-dissociated 2 (putative)	0019941 // modification-dependent protein catabolic process // inferred from electronic annota
66.93405	113.8756	0.029	NM_001037136///NN	1.76	***Agap1***	ArfGAP with GTPase domain, ankyrin repeat and PH domain 1 (Agap1), tra	0007264 // small GTPase mediated signal transduction // inferred from electronic annotation///
220.5379	380.1808	0.016	NM_011046///NM_0	1.76	***Furin***	furin (paired basic amino acid cleaving enzyme)	0019067 // viral assembly, maturation, egress, and release // inferred from electronic annotatio
77.23624	132.5177	0.008	NM_011212	1.76	***Ptpre***	protein tyrosine phosphatase, receptor type, E	0007185 // transmembrane receptor protein tyrosine phosphatase signaling pathway // inferred
253.2188	435.5927	0.018	NM_172257	1.75	***Sidt2***	SID1 transmembrane family, member 2	0016021 // integral to membrane // inferred from electronic annotation///0016020 // membrar
909.9778	1564.273	0.009	NM_022410	1.75	***Myh9***	myosin, heavy polypeptide 9, non-muscle	0007520 // myoblast fusion // inferred from mutant phenotype///0030220 // platelet formation
194.1107	333.0233	0.046	NM_015800	1.75	***Crim1***	cysteine rich transmembrane BMP regulator 1 (chordin like)	0001558 // regulation of cell growth // inferred from electronic annotation
175.302	299.1705	0.024	NM_001025432	1.75	***Crebbp***	CREB binding protein	0030718 // germ-line stem cell maintenance // inferred from mutant phenotype///0006355 // r
73.15476	127.3696	0.005	NM_133779	1.75	***Pigt***	phosphatidylinositol glycan anchor biosynthesis, class T	0051402 // neuron apoptosis // inferred from direct assay///0006506 // GPI anchor biosynthetic
271.6172	465.2588	0.010	NM_010928	1.75	***Notch2***	Notch gene homolog 2 (Drosophila)	0007275 // multicellular organismal development // inferred from electronic annotation///0030
194.5272	332.9098	0.010	XM_001479450///XN	1.74	***Ubr4***	ubiquitin protein ligase E3 component n-recognin 4	0019941 // modification-dependent protein catabolic process // inferred from electronic annota
198.4744	332.1699	0.033	NM_153762	1.74	***Rnf26***	ring finger protein 26	0005515 // protein binding // inferred from electronic annotation///0046872 // metal ion bindir
143.3857	239.6876	0.049	NM_008567	1.74	***Mcm6***	minichromosome maintenance deficient 6 (MIS5 homolog, S. pombe) (S. c	0006270 // DNA replication initiation // inferred from electronic annotation///0006268 // DNA u
716.998	1223.807	0.023	NM_153423	1.74	***Wasf2***	WAS protein family, member 2	0030048 // actin filament-based movement // inferred from mutant phenotype///0006928 // ce
330.8602	563.0998	0.006	NM_015729	1.74	***Acox1***	Acyl-Coenzyme A oxidase 1, palmitoyl (Acox1), mRNA///acyl-Coenzyme A c	0007283 // spermatogenesis // inferred from mutant phenotype///0055114 // oxidation reducti
83.03812	141.2276	0.031	NM_001081290	1.73	***Bat2d***	BAT2 domain containing 1	
109.8652	183.4712	0.042	NM_001130152///NN	1.73	***Arhgef1***	Rho guanine nucleotide exchange factor (GEF) 1	0007242 // intracellular signaling cascade // inferred from electronic annotation///0035023 // r
8.889217	15.54586	0.021	NM_025413///NM_0	1.73	***Lce1g///Lce1f///Lce1c***	late cornified envelope 1G///late cornified envelope 1C///late cornified en	0005515 // protein binding // inferred from physical interaction
68.44147	117.7525	0.002	NM_146258	1.73	***Stard13***	StAR-related lipid transfer (START) domain containing 13	0007049 // cell cycle // inferred from electronic annotation///0007165 // signal transduction //

[ICR, PBS](	[ICR, SUL](p		RefSeq Transcript ID	FC [PBS:SFN]	Gene Symbol	Gene Title	Gene Ontology Biological Process
2843.996	4773.549	0.035	NM_009255	1.73	***Serpine2***	serine (or cysteine) peptidase inhibitor, clade E, member 2	0007275 // multicellular organismal development // inferred from electronic annotation///0030
258.6336	437.6887	0.042	NM_001111121///XN	1.73	***Ccdc6***	coiled-coil domain containing 6	
275.6439	456.0472	0.048	NM_133791	1.73	***Wwc2***	WW, C2 and coiled-coil domain containing 2	0005515 // protein binding // inferred from electronic annotation
219.0385	366.4841	0.019	NM_010813	1.72	***Mnt***	max binding protein	0051726 // regulation of cell cycle // inferred from mutant phenotype///0006355 // regulation c
96.78254	165.8212	0.027	NM_008239	1.72	***Foxq1***	forkhead box Q1	0031069 // hair follicle morphogenesis // inferred from mutant phenotype///0006355 // regulat
16.96264	29.73057	0.039	NM_001081153	1.72	***Unc13c***	unc-13 homolog C (C. elegans)	0006887 // exocytosis // inferred from electronic annotation///0007242 // intracellular signaling
66.70499	111.6266	0.019	NM_009873	1.72	***Cdk6***	cyclin-dependent kinase 6	0007049 // cell cycle // inferred from electronic annotation///0043697 // cell dedifferentiation /
4320.835	7265.781	0.041	NM_001037859	1.72	***Csf1r***	colony stimulating factor 1 receptor	0007169 // transmembrane receptor protein tyrosine kinase signaling pathway // inferred from
65.67841	111.599	0.004	NM_011078	1.72	***Phf2***	PHD finger protein 2	0005515 // protein binding // inferred from electronic annotation///0046872 // metal ion bindir
167.588	282.5595	0.043	NM_177390	1.72	***Myo1d***	myosin ID	0003774 // motor activity // inferred from electronic annotation///0005524 // ATP binding // in
230.6678	385.8307	0.013	NM_176850	1.72	***Bptf***	bromodomain PHD finger transcription factor	0006338 // chromatin remodeling // inferred from sequence or structural similarity///0045449 /
114.484	189.2923	0.038	NM_008224	1.71	***Hcfc1***	host cell factor C1	0007049 // cell cycle // inferred from electronic annotation///0019046 // reactivation of latent v
354.8626	586.1923	0.043	NM_009149	1.71	***Glg1***	golgi apparatus protein 1	0005529 // sugar binding // inferred from electronic annotation
154.8218	258.7969	0.014	NM_001159634///NM	1.71	***Bat2l***	HLA-B associated transcript 2-like	
450.1612	743.838	0.049	NM_015804	1.71	***Atp11a***	ATPase, class VI, type 11A	0015914 // phospholipid transport // inferred from electronic annotation///0006754 // ATP bios
73.63207	123.7675	0.021	NM_172856	1.71	***Lass6***	LAG1 homolog, ceramide synthase 6///LAG1 homolog, ceramide synthase	0030148 // sphingolipid biosynthetic process // inferred from direct assay///0046513 // ceramic
227.1453	384.099	0.017	NM_001013371	1.71	***Dtx3l***	deltex 3-like (Drosophila)	0005515 // protein binding // inferred from electronic annotation///0046872 // metal ion bindir
93.38088	158.1319	0.011	XM_893956///XM_9(	1.71	***Mll5***	myeloid/lymphoid or mixed-lineage leukemia 5	0016568 // chromatin modification // inferred from electronic annotation///0007049 // cell cycl
69.06372	114.7385	0.012	NM_013845	1.71	***Ror1***	receptor tyrosine kinase-like orphan receptor 1	0007169 // transmembrane receptor protein tyrosine kinase signaling pathway // inferred from
57.98469	97.48911	0.022	NM_033072	1.70	***Mbd6***	methyl-CpG binding domain protein 6	0003677 // DNA binding // inferred from electronic annotation
79.57723	132.5319	0.014	NM_001003912	1.70	***Arhgef11***	CDNA clone IMAGE:30669655///Rho guanine nucleotide exchange factor (	0007242 // intracellular signaling cascade // inferred from electronic annotation///0035023 // re
425.1166	702.6921	0.029	NM_010638	1.70	***Klf9***	Kruppel-like factor 9	0007566 // embryo implantation // inferred from direct assay///0006355 // regulation of transc
82.32908	137.7479	0.035	NM_001081247	1.70	***Polr3a***	polymerase (RNA) III (DNA directed) polypeptide A	0006350 // transcription // inferred from electronic annotation
404.1304	662.8152	0.049	NM_001034030///NM	1.70	***Limk2***	LIM motif-containing protein kinase 2	0007283 // spermatogenesis // inferred from mutant phenotype///0006468 // protein amino ac
253.9015	425.1217	0.003	NM_178392	1.70	***Snapc1***	small nuclear RNA activating complex, polypeptide 1	0006355 // regulation of transcription, DNA-dependent // inferred from electronic annotation//
199.7704	330.2058	0.021	XM_204015///NM_0(	1.69	***Rere***	arginine glutamic acid dipeptide (RE) repeats///Arginine glutamic acid dipe	0007275 // multicellular organismal development // inferred from electronic annotation///0006
117.0877	198.0725	0.011	NM_178242///XM_3!	1.69	***Tnrc18***	Zinc finger protein 469, mRNA (cDNA clone IMAGE:5321785)///trinucleotic	0006869 // lipid transport // inferred from electronic annotation
534.7604	880.5059	0.029	NM_138310	1.69	***Apob48r***	apolipoprotein B48 receptor	0008202 // steroid metabolic process // inferred from electronic annotation///0006869 // lipid t
530.3486	889.2313	0.030	NM_001145970///NM	1.69	***Mtap7d1***	microtubule-associated protein 7 domain containing 1	0005819 // spindle // inferred from electronic annotation
508.8031	850.4089	0.007	XM_915167///XM_97	1.69	***Hectd1***	HECT domain containing 1	0019941 // modification-dependent protein catabolic process // inferred from electronic annota
59.86271	97.55676	0.044	NM_134087	1.69	***Fam83h***	family with sequence similarity 83, member H	0001503 // ossification // inferred from electronic annotation
85.27836	141.1566	0.030	XM_907140///XM_13	1.69	***Akap13***	A kinase (PRKA) anchor protein 13 (Akap13), mRNA///A kinase (PRKA) anch	0007242 // intracellular signaling cascade // inferred from electronic annotation///0035023 // re
72.97849	120.9264	0.011	NM_146129	1.69	***Pcif1***	PDX1 C-terminal inhibiting factor 1	0005515 // protein binding // inferred from electronic annotation
576.448	953.1678	0.008	NM_025994	1.69	***Efhd2***	EF hand domain containing 2	0005509 // calcium ion binding // inferred from electronic annotation///0005515 // protein binc
420.6567	697.265	0.025	NM_009868	1.68	***Cdh5***	cadherin 5	0007156 // homophilic cell adhesion // inferred from electronic annotation///0007155 // cell ad
119.2826	197.0566	0.025	NM_178116	1.68	***Camta2***	calmodulin binding transcription activator 2	0006355 // regulation of transcription, DNA-dependent // inferred from electronic annotation//
157.8581	257.3333	0.042	NM_030210	1.68	***Aacs***	acetoacetyl-CoA synthetase	0008152 // metabolic process // inferred from electronic annotation///0006629 // lipid metabol
84.953	139.014	0.022	NM_153062	1.68	***Slc37a1***	solute carrier family 37 (glycerol-3-phosphate transporter), member 1	
564.3482	923.8051	0.026	NM_030249	1.68	***Cttnbp2nl***	CTTNBP2 N-terminal like	0005730 // nucleolus // inferred from electronic annotation///0015629 // actin cytoskeleton // i
340.7094	553.8533	0.043	NM_009282///XM_0(	1.68	***LOC100045442///Stag***	similar to Stromal antigen 1///stromal antigen 1	0051301 // cell division // inferred from electronic annotation///0007049 // cell cycle // inferred
64.18406	103.9823	0.045	NM_001080931	1.68	***Med13***	mediator complex subunit 13	0006357 // regulation of transcription from RNA polymerase II promoter // inferred from electro
870.7371	1435.444	0.049	NM_028072	1.68	***Sulf2***	sulfatase 2	0008152 // metabolic process // inferred from electronic annotation///0006790 // sulfur metab
287.9308	467.1397	0.041	NM_009282///XM_0(	1.67	***LOC100045442///Stag***	similar to Stromal antigen 1///Stromal antigen 1 (Stag1), mRNA///stromal a	0051301 // cell division // inferred from electronic annotation///0007049 // cell cycle // inferred
166.9485	274.9643	0.005	NM_018748	1.67	***Golga4***	golgi autoantigen, golgin subfamily a, 4	0005794 // Golgi apparatus // inferred from direct assay///0000139 // Golgi membrane // inferr
1454.582	2394.269	0.025	NM_145928	1.67	***Tspan14***	tetraspanin 14	0016021 // integral to membrane // inferred from electronic annotation///0016020 // membrar
205.019	332.0504	0.046	NM_001033198	1.67	***Ankrd50***	ankyrin repeat domain 50	
268.3549	432.1902	0.040	NM_007516///NM_0	1.67	***Hnrnpd***	heterogeneous nuclear ribonucleoprotein D	0006355 // regulation of transcription, DNA-dependent // inferred from electronic annotation//
653.0924	1060.732	0.022	NM_010123	1.67	***Eif3a***	eukaryotic translation initiation factor 3, subunit A	0001732 // formation of translation initiation complex // inferred from direct assay///0006412 /
196.5726	318.9371	0.038	NM_011417	1.67	***Smarca4***	SWI/SNF related, matrix associated, actin dependent regulator of chromati	0006334 // nucleosome assembly // traceable author statement///0030198 // extracellular mat
3339.432	5442.125	0.021	NM_026381///NM_0	1.67	***Shisa5***	shisa homolog 5 (Xenopus laevis)	0006915 // apoptosis // inferred from electronic annotation///0006915 // apoptosis // inferred
914.905	1483.796	0.018	NM_010301	1.67	***Gna11***	guanine nucleotide binding protein, alpha 11	0007186 // G-protein coupled receptor protein signaling pathway // inferred from mutant pheno
32.27441	53.55214	0.015	NM_133957///NM_0	1.67	***Nfat5***	Nuclear factor of activated T-cells 5 (Nfat5), transcript variant a, mRNA///n	0001816 // cytokine production // inferred from direct assay///0006355 // regulation of transcri
85.27412	137.1208	0.038	NM_172689	1.66	***Ddx58***	DEAD (Asp-Glu-Ala-Asp) box polypeptide 58	0009615 // response to virus // inferred from electronic annotation///0045087 // innate immun
587.9249	950.9334	0.041	NM_133758	1.66	***Usp47***	ubiquitin specific peptidase 47	0019941 // modification-dependent protein catabolic process // inferred from electronic annota
359.9723	594.2253	0.026	NM_146099	1.66	***D19Wsu162e***	DNA segment, Chr 19, Wayne State University 162, expressed	0016021 // integral to membrane // inferred from electronic annotation///0016020 // membrar
6210.787	9975.187	0.045	NM_011576	1.66	***Tfpi***	tissue factor pathway inhibitor	0007596 // blood coagulation // inferred from electronic annotation
241.8179	390.9808	0.019	NM_133665	1.66	***Mef2d***	myocyte enhancer factor 2D	0006915 // apoptosis // inferred from electronic annotation///0007275 // multicellular organism
154.3802	252.1895	0.014	NM_007865	1.66	***Dll1***	delta-like 1 (Drosophila)	0007275 // multicellular organismal development // inferred from electronic annotation///0009
16.28592	27.11514	0.017	NM_178655///NM_0	1.66	***Ank2***	ankyrin 2, brain	0007165 // signal transduction // inferred from electronic annotation///0034394 // protein loca
176.6948	286.5571	0.033	NM_144868	1.65	***Pcnxl3***	pecanex-like 3 (Drosophila)	0016021 // integral to membrane // inferred from electronic annotation///0016020 // membrar

[ICR, PBS](	[ICR, SUL](p		RefSeq Transcript ID	FC [PBS:SFN]	Gene Symbol	Gene Title	Gene Ontology Biological Process
224.0976	357.7112	0.041	NM_001001144///NM	1.65	***Scap***	SREBF chaperone	0008202 // steroid metabolic process // inferred from electronic annotation///0008203 // chole
254.8455	413.8673	0.006	NM_013651	1.65	***Sf3a2***	splicing factor 3a, subunit 2	0008380 // RNA splicing // inferred from electronic annotation///0006397 // mRNA processing /
82.91287	133.9181	0.006	NM_001081373	1.64	***Cep164***	centrosomal protein 164	0006974 // response to DNA damage stimulus // inferred from electronic annotation///0051301
307.0527	492.6299	0.020	NM_148932	1.64	***Pom121***	nuclear pore membrane protein 121	0051028 // mRNA transport // inferred from electronic annotation///0065002 // intracellular pr
29.4817	49.10988	0.048	NR_001461	1.64	***Kcnq1ot1***	KCNQ1 overlapping transcript 1	0016458 // gene silencing // inferred from direct assay///0010216 // maintenance of DNA meth
121.2904	199.9306	0.030	NM_175666	1.63	***Hist2h2bb***	histone cluster 2, H2bb	0006334 // nucleosome assembly // inferred from electronic annotation
130.1009	209.0963	0.010	NM_009089	1.63	***Polr2a***	polymerase (RNA) II (DNA directed) polypeptide A	0006366 // transcription from RNA polymerase II promoter // inferred from electronic annotatic
92.93684	149.5848	0.008	XR_032210	1.63	***LOC100045684***	similar to N-myristoyltransferase 1	
454.8436	739.0208	0.016	NM_010253	1.63	***Gal***	galanin	0006950 // response to stress // inferred from electronic annotation///0031943 // regulation of
198.0269	318.2334	0.011	NM_010111	1.63	***Efnb2***	ephrin B2	0007275 // multicellular organismal development // inferred from electronic annotation///0001
49.54213	80.23303	0.038	NM_026083	1.63	***Zc3h13***	zinc finger CCCH type containing 13	0046872 // metal ion binding // inferred from electronic annotation///0008270 // zinc ion bindi
539.9053	881.6233	0.038	NM_011058///NM_0	1.63	***Pdgfra***	platelet derived growth factor receptor, alpha polypeptide	0060021 // palate development // inferred from mutant phenotype///0060325 // face morphog
96.56848	156.9758	0.024	NM_170599	1.62	***Igsf11***	immunoglobulin superfamily, member 11	0007155 // cell adhesion // inferred from electronic annotation///0040008 // regulation of grow
306.019	480.1687	0.038	NM_138659	1.62	***Prpf8***	pre-mRNA processing factor 8	0008380 // RNA splicing // inferred from electronic annotation///0006397 // mRNA processing /
66.96044	109.5213	0.043	NM_001033212	1.62	***Rprml***	reprimo-like	0016021 // integral to membrane // inferred from electronic annotation///0016020 // membrar
128.9385	203.384	0.028	NM_057171	1.62	***Bat3***	HLA-B-associated transcript 3	0042981 // regulation of apoptosis // inferred from reviewed computational analysis///0006915
63.85999	100.1216	0.028	NM_011379	1.61	***Sipa1***	signal-induced proliferation associated gene 1	0051726 // regulation of cell cycle // inferred from direct assay///0051056 // regulation of small
180.3007	287.9438	0.023	NM_023544///NM_0	1.61	***Rsc1a1///Ddi2***	DNA-damage inducible protein 2///regulatory solute carrier protein, family	0051051 // negative regulation of transport // inferred from electronic annotation///0050892 //
550.3471	888.0051	0.034	NM_011480	1.61	***Srebf1***	sterol regulatory element binding transcription factor 1	0008202 // steroid metabolic process // inferred from electronic annotation///0006355 // regul
199.9897	312.6738	0.031	NM_022882	1.61	***Lpin2***	Lipin 2, mRNA (cDNA clone MGC:46819 IMAGE:5101211)///lipin 2	0008195 // phosphatidate phosphatase activity // inferred from direct assay
144.9849	229.1207	0.015	NM_028412	1.61	***Ciz1***	CDKN1A interacting zinc finger protein 1	0046872 // metal ion binding // inferred from electronic annotation///0008270 // zinc ion bindi
36.40942	57.92859	0.002	NM_009297	1.61	***Supt6h***	suppressor of Ty 6 homolog (S. cerevisiae)	0006357 // regulation of transcription from RNA polymerase II promoter // inferred from electro
57.29696	91.00245	0.024	NM_001033759///NM	1.61	***Tmem2***	Transmembrane protein 2 (Tmem2), transcript variant 1, mRNA///transme	0016021 // integral to membrane // inferred from electronic annotation///0016020 // membrar
23.34509	37.35414	0.005	NM_009405	1.61	***Tnni2***	troponin I, skeletal, fast 2	0045941 // positive regulation of transcription // inferred from electronic annotation///000300
92.19415	146.5433	0.001	NM_001114386///NM	1.61	***Nedd4l***	neural precursor cell expressed, developmentally down-regulated gene 4-l	0010766 // negative regulation of sodium ion transport // inferred from direct assay///0019941
377.2236	592.6205	0.021	NM_001005331///NM	1.61	***Eif4g1***	eukaryotic translation initiation factor 4, gamma 1	0006412 // translation // inferred from electronic annotation///0016070 // RNA metabolic proc
88.74268	138.6623	0.042	NM_022022///XM_0	1.61	***Ube4b***	ubiquitination factor E4B, UFD2 homolog (S. cerevisiae)///Ubiquitination fa	0019941 // modification-dependent protein catabolic process // inferred from electronic annota
160.3729	252.7411	0.004	NM_153103	1.60	***Kif1c***	kinesin family member 1C	0006890 // retrograde vesicle-mediated transport, Golgi to ER // inferred from direct assay///00
197.9069	313.0269	0.005	NM_021899	1.60	***Foxj2***	forkhead box J2	0006355 // regulation of transcription, DNA-dependent // inferred from electronic annotation//
189.385	307.7082	0.046	NM_024440	1.60	***Derl3***	Der1-like domain family, member 3	0005783 // endoplasmic reticulum // inferred from electronic annotation///0005789 // endopla
414.1126	655.2076	0.021	NM_009593	1.60	***Abcg1***	ATP-binding cassette, sub-family G (WHITE), member 1	0006869 // lipid transport // inferred from electronic annotation///0045449 // regulation of trar
60.70678	95.99432	0.005	NM_144812///NM_1	1.60	***Tnrc6b***	trinucleotide repeat containing 6b	0006417 // regulation of translation // inferred from electronic annotation///0031047 // gene si
599.9963	956.6914	0.031	NM_011498	1.60	***Bhlhe40***	basic helix-loop-helix family, member e40	0045892 // negative regulation of transcription, DNA-dependent // inferred from direct assay///
175.181	271.003	0.043	NM_008633	1.60	***Mtap4***	microtubule-associated protein 4	0007026 // negative regulation of microtubule depolymerization // inferred from electronic ann
1377.441	2192.68	0.027	NM_173182	1.60	***Fndc3b***	fibronectin type III domain containing 3B	0045600 // positive regulation of fat cell differentiation // inferred from physical interaction
186.8104	290.671	0.044	NM_027144	1.60	***Arhgef12***	Rho guanine nucleotide exchange factor (GEF) 12	0007242 // intracellular signaling cascade // inferred from electronic annotation///0007186 // G
1313.981	2044.088	0.029	NM_146130///XM_8	1.59	***LOC100045099///OTTI***	predicted gene, EG627828///predicted gene, OTTMUSG00000004599///sir	0008380 // RNA splicing // inferred from electronic annotation///0006397 // mRNA processing /
99.51416	153.988	0.049	NM_001081255	1.59	***Lrch3***	Leucine-rich repeats and calponin homology (CH) domain containing 3, mR	0005515 // protein binding // inferred from electronic annotation
99.03201	157.2356	0.015	NM_021521	1.59	***Med12***	mediator of RNA polymerase II transcription, subunit 12 homolog (yeast)	0035176 // social behavior // inferred from mutant phenotype///0006357 // regulation of trans
116.5475	181.5619	0.031	NM_138679	1.59	***Ash1l***	ash1 (absent, small, or homeotic)-like (Drosophila)	0016568 // chromatin modification // inferred from electronic annotation///0006355 // regulati
305.6621	481.4534	0.002	NM_001145952///NM	1.59	***Lpp***	LIM domain containing preferred translocation partner in lipoma///LIM do	0007155 // cell adhesion // inferred from electronic annotation
89.41513	141.7813	0.029	XM_001481284///XM	1.59	***Wipf3***	WAS/WASL interacting protein family, member 3	0007275 // multicellular organismal development // inferred from electronic annotation///0030
472.7928	762.8283	0.048	NM_172685	1.59	***Slc25a24***	solute carrier family 25 (mitochondrial carrier, phosphate carrier), member	0006810 // transport // inferred from electronic annotation
148.7044	229.8842	0.043	XM_001476519///NM	1.59	***Brd1///LOC100045983***	similar to bromodomain containing 1///bromodomain containing 1	0005515 // protein binding // inferred from electronic annotation///0046872 // metal ion bindir
61.34346	98.28765	0.032	NM_008616///NM_0	1.59	***Zfp239***	zinc finger protein 239	0006355 // regulation of transcription, DNA-dependent // inferred from electronic annotation//
481.8109	748.8629	0.031	NM_009045	1.59	***Rela***	v-rel reticuloendotheliosis viral oncogene homolog A (avian)	0051092 // positive regulation of NF-kappaB transcription factor activity // inferred from electro
394.1875	616.3745	0.016	NM_175344	1.58	***Ano6***	anoctamin 6	0006811 // ion transport // inferred from electronic annotation///0006810 // transport // inferr
143.186	221.0468	0.026	NM_010613	1.58	***Khsrp***	KH-type splicing regulatory protein	0006412 // translation // inferred from electronic annotation///0051028 // mRNA transport // i
92.5527	144.7332	0.024	NM_001081057	1.58	***Tecpr2***	tectonin beta-propeller repeat containing 2	0005634 // nucleus // inferred from electronic annotation///0005737 // cytoplasm // inferred fr
340.354	533.0895	0.021	NM_001033275	1.58	***Glt8d3***	glycosyltransferase 8 domain containing 3	0016757 // transferase activity, transferring glycosyl groups // inferred from electronic annotati
334.4582	514.9414	0.033	NM_007460	1.58	***Ap3d1***	adaptor-related protein complex 3, delta 1 subunit	0048007 // antigen processing and presentation, exogenous lipid antigen via MHC class Ib // infe
119.3363	183.6027	0.038	NM_145970	1.58	***Cc2d1a***	coiled-coil and C2 domain containing 1A	0006355 // regulation of transcription, DNA-dependent // inferred from electronic annotation//
261.5124	401.6401	0.032	NM_133947	1.58	***Numa1***	Nuclear mitotic apparatus protein 1 (Numa1), mRNA///nuclear mitotic app	0051321 // meiotic cell cycle // inferred from electronic annotation
557.5716	859.9428	0.036	NM_146012///XR_03	1.58	***Ctdsp2///ENSMUSG00***	CTD (carboxy-terminal domain, RNA polymerase II, polypeptide A) small ph	0016791 // phosphatase activity // inferred from electronic annotation///0004721 // phosphopr
59.91959	92.56378	0.015	NM_001114609///NM	1.58	***Ino80d***	INO80 complex subunit D	
178.9488	274.9617	0.027	NM_007795	1.58	***Ctf1***	cardiotrophin 1	0048666 // neuron development // inferred from genetic interaction
40.61821	63.71928	0.006	XM_001477657///XM	1.58	***BC059842***	cDNA sequence BC059842///PREDICTED: Mus musculus cDNA sequence BC059842 (BC059842), mRNA	
272.0418	421.186	0.031	NM_001081298///XR	1.58	***Lphn2///LOC10004805***	latrophilin 2///similar to calcium-independent alpha-latrotoxin receptor hc	0007165 // signal transduction // inferred from electronic annotation///0007218 // neuropeptic
48.78642	75.21652	0.049	NM_017401	1.58	***Polm***	polymerase (DNA directed), mu	0030183 // B cell differentiation // inferred from mutant phenotype///0016446 // somatic hype
112.555	176.6525	0.026	NM_009755	1.57	***Bmp1***	bone morphogenetic protein 1	0001503 // ossification // inferred from electronic annotation///0007275 // multicellular organi:

[ICR, PBS](	[ICR, SUL](p		RefSeq Transcript ID	FC [PBS:SFN]	Gene Symbol	Gene Title	Gene Ontology Biological Process
429.6642	662.8736	0.010	NM_133852///NM_0	1.57	***Golga2***	golgi autoantigen, golgin subfamily a, 2	0005515 // protein binding // inferred from electronic annotation
81.00088	124.2209	0.031	NM_181071	1.57	***Tanc2***	tetratricopeptide repeat, ankyrin repeat and coiled-coil containing 2	0005488 // binding // inferred from electronic annotation
127.7242	198.16	0.000	NM_007772	1.57	***Hivep1***	human immunodeficiency virus type I enhancer binding protein 1	0045892 // negative regulation of transcription, DNA-dependent // inferred from direct assay///
56.00654	86.90212	0.024	NM_177136///NM_0	1.57	***Fryl***	furry homolog-like (Drosophila)	0005488 // binding // inferred from electronic annotation///0005515 // protein binding // inferr
101.7255	155.3127	0.047	NM_001029994///NM	1.57	***Zc3h18***	zinc finger CCCH-type containing 18	0046872 // metal ion binding // inferred from electronic annotation///0008270 // zinc ion bindi
138.0526	214.6047	0.011	NM_001017427	1.57	***Rasef***	RAS and EF hand domain containing	0007264 // small GTPase mediated signal transduction // inferred from electronic annotation///
140.3343	216.3232	0.022	NM_001003815///NM	1.57	***Epb4.1l1***	erythrocyte protein band 4.1-like 1	0030866 // cortical actin cytoskeleton organization // inferred from electronic annotation
89.84402	139.3159	0.050	NM_001039530	1.57	***Parp14***	poly (ADP-ribose) polymerase family, member 14	0006355 // regulation of transcription, DNA-dependent // inferred from electronic annotation//
116.495	179.0293	0.015	NM_177592	1.56	***Tmem164***	transmembrane protein 164///CDNA clone IMAGE:6815971	0016021 // integral to membrane // inferred from electronic annotation///0016020 // membrar
632.6245	970.8284	0.033	NM_145629	1.56	***Pls3***	plastin 3 (T-isoform)	0007015 // actin filament organization // inferred from electronic annotation
74.1358	113.022	0.025	NM_009744	1.56	***Bcl6***	B-cell leukemia/lymphoma 6	0043380 // regulation of memory T cell differentiation // inferred from mutant phenotype///00!
18.41829	28.43762	0.041	NM_199222	1.56	***Lman1l***	lectin, mannose-binding 1 like	0005529 // sugar binding // inferred from electronic annotation
713.3142	1101.267	0.026	NM_207515///NM_1	1.56	***Mbnl2***	muscleblind-like 2///MKIAA4072 protein	0003723 // RNA binding // inferred from electronic annotation///0046872 // metal ion binding /
350.8924	536.263	0.029	NM_054097	1.56	***Pip4k2c***	phosphatidylinositol-5-phosphate 4-kinase, type II, gamma	0046488 // phosphatidylinositol metabolic process // inferred from electronic annotation
170.4751	262.1913	0.007	NM_009848	1.56	***Entpd1***	ectonucleoside triphosphate diphosphohydrolase 1	0030168 // platelet activation // inferred from direct assay///0009181 // purine ribonucleoside
29.36248	44.99492	0.010	NM_011518	1.56	***Syk***	Spleen tyrosine kinase (Syk), mRNA///spleen tyrosine kinase	0050731 // positive regulation of peptidyl-tyrosine phosphorylation // inferred from mutant phe
455.575	697.1745	0.008	NM_181594	1.56	***Edc4***	enhancer of mRNA decapping 4	0005515 // protein binding // inferred from electronic annotation
151.4481	229.5615	0.033	NM_033144	1.55	***8-Sep***	septin 8	0007049 // cell cycle // inferred from electronic annotation
194.0777	294.0106	0.042	XM_001474137///NM	1.55	***LOC100043998///Tpr***	translocated promoter region///similar to nuclear pore complex-associate	0006412 // translation // inferred from electronic annotation///0006434 // seryl-tRNA aminoac
101.6337	157.5316	0.038	NM_183017	1.55	***Ttll12***	TTL domain protein (Ttll12 gene), strain C57BL6///tubulin tyrosine ligase-li	0006464 // protein modification process // inferred from electronic annotation
29.29646	44.46393	0.031	NM_133667	1.55	***Pdk2***	pyruvate dehydrogenase kinase, isoenzyme 2	0007165 // signal transduction // inferred from electronic annotation///0005975 // carbohydrat
219.2237	334.5379	0.019	NM_130863	1.55	***Adrbk1***	adrenergic receptor kinase, beta 1	0016310 // phosphorylation // inferred from electronic annotation///0007165 // signal transduc
1136.67	1717.159	0.043	NM_001033268	1.55	***Fam120a***	family with sequence similarity 120, member A	0003723 // RNA binding // inferred from electronic annotation
68.28994	103.982	0.011	NM_001044719///NM	1.55	***D17Wsu92e***	DNA segment, Chr 17, Wayne State University 92, expressed///DNA segment, Chr 17, Wayne State University 92, expressed, mRNA (cDNA clone MGC:178969 IMAGE:9053	
79.57266	122.4096	0.014	NM_028230	1.55	***Shmt2***	serine hydroxymethyltransferase 2 (mitochondrial)	0006544 // glycine metabolic process // inferred from electronic annotation///0006563 // L-seri
233.1955	357.2964	0.014	NM_007908	1.55	***Eef2k***	eukaryotic elongation factor-2 kinase	0006468 // protein amino acid phosphorylation // inferred from electronic annotation
310.8276	479.9587	0.045	NM_007707	1.55	***Socs3***	suppressor of cytokine signaling 3	0019941 // modification-dependent protein catabolic process // inferred from electronic annota
50.73664	76.76267	0.037	NM_001001738	1.55	***Itprip***	inositol 1,4,5-triphosphate receptor interacting protein	
211.457	322.2477	0.005	NM_153533	1.55	***Tenc1***	tensin like C1 domain-containing phosphatase	0048871 // multicellular organismal homeostasis // inferred from mutant phenotype///0001822
97.72817	148.2722	0.034	NM_144872	1.55	***Eml3***	echinoderm microtubule associated protein like 3	0005874 // microtubule // inferred from electronic annotation///0005737 // cytoplasm // inferr
46.74178	71.18797	0.049	NM_153543	1.55	***Aldh1l2***	aldehyde dehydrogenase 1 family, member L2	0055114 // oxidation reduction // inferred from electronic annotation///0009058 // biosyntheti
66.26363	100.0645	0.019	NM_001110132///NM	1.55	***Cic***	capicua homolog (Drosophila)	0006355 // regulation of transcription, DNA-dependent // inferred from electronic annotation//
104.5636	156.971	0.033	NM_173048	1.54	***Gga3***	golgi associated, gamma adaptin ear containing, ARF binding protein 3	0006810 // transport // inferred from electronic annotation///0016192 // vesicle-mediated tran
105.2669	161.4533	0.011	NM_011424	1.54	***Ncor2***	nuclear receptor co-repressor 2	0045892 // negative regulation of transcription, DNA-dependent // inferred from direct assay///
665.6628	1000.735	0.043	NM_144530	1.54	***Zc3h11a***	zinc finger CCCH type containing 11A	0046872 // metal ion binding // inferred from electronic annotation///0008270 // zinc ion bindi
199.9942	301.5945	0.022	NM_015731	1.54	***Atp9a***	ATPase, class II, type 9A	0008152 // metabolic process // inferred from electronic annotation///0015914 // phospholipid
139.263	207.8604	0.034	NM_023544///NM_0	1.53	***Rsc1a1///Ddi2***	DNA-damage inducible protein 2///regulatory solute carrier protein, family	0051051 // negative regulation of transport // inferred from electronic annotation///0050892 //
910.7809	1369.686	0.015	NM_146130///XM_8	1.53	***LOC100045099///OTTI***	predicted gene, EG627828///predicted gene, OTTMUSG00000004599///si	0008380 // RNA splicing // inferred from electronic annotation///0006397 // mRNA processing /
24.45775	36.78954	0.029	NM_027560	1.53	***Arrdc2***	arrestin domain containing 2	
239.7012	362.9312	0.005	NM_011682	1.53	***Utrn***	utrophin	0007528 // neuromuscular junction development // inferred from electronic annotation///0007
317.4151	474.4151	0.042	NM_133221	1.53	***Slc24a6***	solute carrier family 24 (sodium/potassium/calcium exchanger), member 6	0006816 // calcium ion transport // inferred from electronic annotation///0006814 // sodium io
244.6859	370.7276	0.028	NM_019651	1.53	***Ptpn9***	protein tyrosine phosphatase, non-receptor type 9	0016311 // dephosphorylation // inferred from electronic annotation///0006470 // protein amir
78.12888	116.8522	0.029	NM_178236///NR_0C	1.53	***Asb7***	ankyrin repeat and SOCS box-containing 7	0019941 // modification-dependent protein catabolic process // inferred from electronic annota
34.23529	51.80119	0.045	NM_010820	1.53	***Mpdz***	multiple PDZ domain protein	0007155 // cell adhesion // inferred from direct assay
59.79078	89.56892	0.046	NM_025690///NM_0	1.53	***Sltm***	SAFB-like, transcription modulator	0006915 // apoptosis // inferred from electronic annotation///0006355 // regulation of transcri
59.55757	88.93058	0.033	NM_145416	1.52	***Kri1***	KRI1 homolog (S. cerevisiae)	
21.31657	32.06295	0.027	NM_009004	1.52	***Kif20a***	kinesin family member 20A	0006810 // transport // inferred from electronic annotation///0007018 // microtubule-based m
116.8092	174.9726	0.014	NM_194346	1.52	***Rnf31***	ring finger protein 31	0016874 // ligase activity // inferred from electronic annotation///0046872 // metal ion binding
168.064	251.2349	0.020	NM_133940	1.52	***Fbxl14***	F-box and leucine-rich repeat protein 14	0019941 // modification-dependent protein catabolic process // inferred from electronic annota
62.99466	93.62002	0.036	NM_033523	1.52	***Spred2***	sprouty-related, EVH1 domain containing 2	0007275 // multicellular organismal development // inferred from electronic annotation///0000
133.6452	199.1525	0.018	NM_021280	1.52	***Plcg1***	Phospholipase C, gamma 1 (Plcg1), mRNA///phospholipase C, gamma 1	0050852 // T cell receptor signaling pathway // inferred from direct assay///0016042 // lipid cat
310.7354	463.7519	0.015	XR_035649///NM_00	1.52	***EG666167///Ewsr1***	predicted gene, EG666167///Ewing sarcoma breakpoint region 1	0006355 // regulation of transcription, DNA-dependent // inferred from electronic annotation//
49.95923	73.98124	0.045	XR_034155///NM_00	1.51	***LOC100048010///Cbl***	Casitas B-lineage lymphoma///Casitas B-lineage lymphoma (Cbl), mRNA///	0019941 // modification-dependent protein catabolic process // inferred from electronic annota
188.6133	277.9381	0.024	NM_033371	1.51	***Ppp1r16a***	protein phosphatase 1, regulatory (inhibitor) subunit 16A	0005829 // cytosol // traceable author statement///0016020 // membrane // inferred from elec
140.9047	206.8401	0.031	NM_033563	1.51	***Klf7***	Kruppel-like factor 7 (ubiquitous)	0048813 // dendrite morphogenesis // inferred from mutant phenotype///0007409 // axonoger
171.6281	253.4763	0.021	NM_001081685///NM	1.51	***Zfp295***	zinc finger protein 295	0005622 // intracellular // inferred from electronic annotation
120.1808	178.7498	0.039	NM_001081132	1.50	***Upf2***	UPF2 regulator of nonsense transcripts homolog (yeast)	0006986 // response to unfolded protein // not recorded///0016070 // RNA metabolic process /
70.50968	103.7012	0.029	NM_174868	1.50	***Fam73a***	family with sequence similarity 73, member A	0016021 // integral to membrane // inferred from electronic annotation///0016020 // membrar
120.1555	177.9817	0.026	NM_199028	1.50	***Bend3***	BEN domain containing 3	
466.7545	695.7762	0.009	NM_030238	1.50	***Dync1h1***	dynein cytoplasmic 1 heavy chain 1	0007018 // microtubule-based movement // inferred from electronic annotation

[ICR, PBS](	[ICR, SUL](	p	RefSeq Transcript ID	FC [PBS:SFN]	Gene Symbol	Gene Title	Gene Ontology Biological Process
206.2862	307.6928	0.011	NM_026014	1.50	***Cdt1***	chromatin licensing and DNA replication factor 1	0000076 // DNA replication checkpoint // inferred from electronic annotation///0000076 // DN/
152.0945	226.2159	0.012	NM_001114097///NN	1.50	***Smarcc2***	SWI/SNF related, matrix associated, actin dependent regulator of chromati	0016568 // chromatin modification // inferred from electronic annotation///0006355 // regulati
222.6589	145.6392	0.010	NM_025683	-1.50	***Rpe***	ribulose-5-phosphate-3-epimerase	0008152 // metabolic process // inferred from electronic annotation///0005975 // carbohydrate
656.3445	432.1881	0.004	NM_001130149///NN	-1.50	***LOC100045148///Rnas***	ribonuclease III, nuclear///similar to ribonuclease III, nuclear	0006396 // RNA processing // inferred from electronic annotation
663.184	435.0452	0.025	NM_145392	-1.51	***Bag2***	BCL2-associated athanogene 2	0019538 // protein metabolic process // inferred from electronic annotation///0006915 // apop
980.876	638.2849	0.011	NM_145405	-1.51	***Ubl4***	ubiquitin-like 4	0006464 // protein modification process // inferred from electronic annotation
154.1863	100.299	0.032	NM_145133	-1.51	***Tifa***	TRAF-interacting protein with forkhead-associated domain	0007249 // I-kappaB kinase/NF-kappaB cascade // inferred from direct assay
510.9264	331.0704	0.023	NM_011883///NM_0	-1.51	***Rnf13***	ring finger protein 13	0005515 // protein binding // inferred from electronic annotation///0046872 // metal ion bindir
416.1779	267.4393	0.049	NM_029581	-1.51	***Mtif3***	mitochondrial translational initiation factor 3	0032790 // ribosome disassembly // inferred from sequence or structural similarity///0006446 /
1091.826	710.4048	0.047	XM_001474604///NN	-1.51	***Higd1a///LOC1000457***	predicted gene, ENSMUSG00000044330///HIG1 domain family, member 1	0006950 // response to stress // inferred from electronic annotation
1168.432	769.4538	0.045	NM_026878	-1.52	***Rasl11b***	RAS-like, family 11, member B	0007264 // small GTPase mediated signal transduction // inferred from electronic annotation
1380.347	893.762	0.011	NM_025848	-1.52	***Sdhd***	succinate dehydrogenase complex, subunit D, integral membrane protein	0006810 // transport // inferred from electronic annotation///0006099 // tricarboxylic acid cycl
518.5929	332.9025	0.018	NM_026276	-1.52	***Aasdhppt***	aminoadipate-semialdehyde dehydrogenase-phosphopantetheinyl transfe	0009059 // macromolecule biosynthetic process // inferred from electronic annotation
131.5069	84.73762	0.013	NM_025408	-1.52	***Acer3***	alkaline ceramidase 3	0006672 // ceramide metabolic process // inferred from electronic annotation
1884.869	1213.266	0.034	XM_001478170///NN	-1.52	***Nae1///LOC10004739***	similar to Amyloid beta precursor protein binding protein 1///NEDD8 activ	0019941 // modification-dependent protein catabolic process // inferred from electronic annota
172.7651	111.0249	0.012	NM_194269	-1.52	***Morn2***	MORN repeat containing 2	
1949.802	1261.678	0.017	NM_016778	-1.52	***Bok***	BCL2-related ovarian killer protein	0006915 // apoptosis // inferred from electronic annotation///0006917 // induction of apoptosi
2497.827	1613.335	0.017	NM_029478	-1.52	***Tmem49***	transmembrane protein 49	0005783 // endoplasmic reticulum // inferred from electronic annotation///0033116 // ER-Golgi
83.85282	54.2442	0.020	NM_010839///NM_0	-1.52	***Mtcp1***	mature T-cell proliferation 1	0005739 // mitochondrion // inferred from electronic annotation///0005739 // mitochondrion /
642.4833	411.8186	0.020	NM_026794	-1.52	***Deb1***	differentially expressed in B16F10 1	
953.4912	615.7377	0.007	NM_011851	-1.52	***Nt5e***	5' nucleotidase, ecto///5' nucleotidase, ecto (Nt5e), mRNA	0050728 // negative regulation of inflammatory response // inferred from mutant phenotype///
425.273	274.5216	0.021	NM_026959	-1.52	***Stx18***	syntaxin 18	0006810 // transport // inferred from electronic annotation///0016192 // vesicle-mediated tran
290.2039	187.0178	0.010	NM_175560///NM_1	-1.53	***9130019O22Rik///Zfp7***	zinc finger protein 747///RIKEN cDNA 9130019O22 gene///RIKEN cDNA E4	0006355 // regulation of transcription, DNA-dependent // inferred from electronic annotation//
41.79565	26.98269	0.002	NM_026734	-1.53	***Tmem126b***	transmembrane protein 126B	0016021 // integral to membrane // inferred from electronic annotation///0016020 // membrar
106.1479	68.0648	0.030	NM_001077713	-1.53	***Acn9***	ACN9 homolog (S. cerevisiae)	0006094 // gluconeogenesis // inferred from sequence or structural similarity
3946.493	2540.61	0.029	NM_013470	-1.53	***Anxa3***	annexin A3	0019834 // phospholipase A2 inhibitor activity // inferred from electronic annotation///000554
33.78617	21.66928	0.045	NM_053253	-1.53	***Zmynd10***	zinc finger, MYND domain containing 10	0046872 // metal ion binding // inferred from electronic annotation///0008270 // zinc ion bindir
1462.523	947.2139	0.012	NM_144897	-1.53	***Apoa1bp***	apolipoprotein A-I binding protein	0005515 // protein binding // not recorded
315.7233	202.5707	0.009	NM_172988	-1.53	***Fbxl4***	F-box and leucine-rich repeat protein 4	0019941 // modification-dependent protein catabolic process // inferred from electronic annota
489.8318	317.1462	0.033	NM_001081233///XR	-1.53	***EG433923///Slc25a5//***	predicted gene, EG433923///predicted gene, EG667030///predicted gene,	0006810 // transport // inferred from electronic annotation
2932.246	1880.48	0.048	XM_001474604///NN	-1.53	***Higd1a///LOC1000457***	predicted gene, ENSMUSG00000044330///HIG1 domain family, member 1	0006950 // response to stress // inferred from electronic annotation
921.4403	585.7483	0.020	NM_007530	-1.53	***Bcap29***	B-cell receptor-associated protein 29	0006915 // apoptosis // inferred from electronic annotation///0006810 // transport // inferred
535.4031	342.8602	0.037	NM_024174	-1.54	***Mrps23***	mitochondrial ribosomal protein S23	0003735 // structural constituent of ribosome // inferred from sequence or structural similarity
464.3846	303.42	0.040	NM_175116	-1.54	***P2ry5***	purinergic receptor P2Y, G-protein coupled, 5	0007165 // signal transduction // inferred from electronic annotation///0007186 // G-protein cc
500.6631	318.0464	0.021	NM_001082532	-1.54	***Pigyl***	phosphatidylinositol glycan anchor biosynthesis, class Y-like	0006506 // GPI anchor biosynthetic process // inferred from electronic annotation
13.11494	8.277576	0.030	XM_001480867///XN	-1.54	***Cd27***	CD27 antigen	0048305 // immunoglobulin secretion // non-traceable author statement///0006915 // apoptos
988.4976	628.9379	0.047	NM_144916	-1.54	***Tmem150***	transmembrane protein 150	0016021 // integral to membrane // inferred from electronic annotation///0016021 // integral t
596.5231	377.851	0.028	NM_025301	-1.55	***Mrpl17***	mitochondrial ribosomal protein L17	0006412 // translation // not recorded///0006412 // translation // inferred from electronic annc
476.4685	299.4194	0.026	NM_023203	-1.55	***Dctpp1***	dCTP pyrophosphatase 1	0051289 // protein homotetramerization // inferred from direct assay
91.72947	58.15486	0.013	NM_025469	-1.55	***Clps***	colipase, pancreatic	0016042 // lipid catabolic process // inferred from electronic annotation///0007586 // digestion
713.002	453.6092	0.005	NM_025606	-1.55	***Mrpl16***	mitochondrial ribosomal protein L16	0006412 // translation // inferred from electronic annotation///0006412 // translation // inferre
40.25615	25.29746	0.021	NM_013600///NM_0	-1.55	***Msh5***	mutS homolog 5 (E. coli)	0007292 // female gamete generation // inferred from mutant phenotype///0006986 // respon:
75.99167	48.63821	0.018	NM_013848	-1.55	***Ermap***	erythroblast membrane-associated protein	0031410 // cytoplasmic vesicle // inferred from direct assay///0005886 // plasma membrane //
689.6768	432.4806	0.025	NM_139229	-1.55	***Pdf///Cog8***	peptide deformylase (mitochondrial)///component of oligomeric golgi com	0006810 // transport // inferred from electronic annotation///0015031 // protein transport // ir
38.46425	24.01132	0.036	NM_009250	-1.55	***Serpini1***	Serine (or cysteine) peptidase inhibitor, clade I, member 1, mRNA (cDNA clone MGC:6413 IMAGE:3588856)///serine (or cysteine) peptidase inhibitor, clade I, member 1	
310.948	198.2547	0.022	XM_980440///NM_0	-1.56	***Epb4.1l4b***	erythrocyte protein band 4.1-like 4b	0008092 // cytoskeletal protein binding // inferred from electronic annotation///0005488 // bin
288.2301	181.93	0.029	NM_011227	-1.56	***Rab20***	RAB20, member RAS oncogene family	0007264 // small GTPase mediated signal transduction // inferred from electronic annotation///
294.0204	184.8419	0.019	NM_001127191///NN	-1.56	***Snx16***	sorting nexin 16	0006810 // transport // inferred from electronic annotation///0015031 // protein transport // ir
326.6604	204.8988	0.023	NM_178603	-1.56	***Mrpl50***	mitochondrial ribosomal protein L50	0005739 // mitochondrion // inferred from electronic annotation///0030529 // ribonucleoprote
1876.378	1180.146	0.015	NM_026618	-1.56	***Ccdc56***	coiled-coil domain containing 56	0016021 // integral to membrane // inferred from electronic annotation///0005739 // mitochor
424.6869	265.5952	0.047	NM_133823	-1.56	***Mmaa***	methylmalonic aciduria (cobalamin deficiency) type A	0017111 // nucleoside-triphosphatase activity // inferred from electronic annotation///0005524
292.9338	184.0178	0.014	NM_027091	-1.56	***Nup35***	nucleoporin 35	0051028 // mRNA transport // inferred from electronic annotation///0065002 // intracellular pr
312.6984	197.4192	0.025	NM_133783	-1.56	***Ptges2***	prostaglandin E synthase 2	0046903 // secretion // inferred from direct assay///0045454 // cell redox homeostasis // inferr
1636.327	1027.339	0.047	NM_134007	-1.56	***Cisd1***	CDGSH iron sulfur domain 1	0043457 // regulation of cellular respiration // inferred from mutant phenotype
56.7785	36.335	0.035	NM_029610	-1.56	***Lyrm1***	LYR motif containing 1	
681.6025	428.0858	0.005	NM_024227	-1.57	***Mrpl28***	mitochondrial ribosomal protein L28	0045449 // regulation of transcription // inferred from electronic annotation
244.2457	153.2847	0.008	NM_001159612///NN	-1.57	***Lrrc57***	leucine rich repeat containing 57	0005515 // protein binding // inferred from electronic annotation
2692.067	1692.505	0.034	NM_025403	-1.57	***Nop10***	NOP10 ribonucleoprotein homolog (yeast)	0042254 // ribosome biogenesis // inferred from electronic annotation///0006364 // rRNA proc
296.6109	182.8616	0.045	NM_172575///NM_1	-1.57	***Zfp277***	zinc finger protein 277	0005515 // protein binding // inferred from electronic annotation///0008270 // zinc ion binding
132.542	83.09451	0.018	NM_029321	-1.57	***Ttc32***	tetratricopeptide repeat domain 32	0005488 // binding // inferred from electronic annotation

[ICR, PBS](	[ICR, SUL](	p	RefSeq Transcript ID	FC [PBS:SFN]	Gene Symbol	Gene Title	Gene Ontology Biological Process
60.01769	37.55688	0.002	NM_145146	-1.58	***Afm***	afamin	0006810 // transport // inferred from electronic annotation
591.2401	365.0035	0.018	NM_177910	-1.58	***Gmppb***	GDP-mannose pyrophosphorylase B	0009058 // biosynthetic process // inferred from electronic annotation///0007157 // heterophil
640.9873	399.33	0.003	NM_027296	-1.59	***Trnt1***	tRNA nucleotidyl transferase, CCA-adding, 1	0008033 // tRNA processing // inferred from electronic annotation///0006396 // RNA processing
327.7994	200.8331	0.026	NM_026732	-1.59	***Mrpl14***	mitochondrial ribosomal protein L14///Mitochondrial ribosomal protein L1	0006412 // translation // inferred from electronic annotation
63.00163	38.51973	0.029	NM_026161	-1.59	***C1qtnf4***	C1q and tumor necrosis factor related protein 4, mRNA (cDNA clone IMAGE:3668760)///C1q and tumor necrosis factor related protein 4	
1810.035	1128.829	0.022	NM_018819	-1.59	***Brp44l***	brain protein 44-like	0005739 // mitochondrion // inferred from electronic annotation///0005743 // mitochondrial in
1139.32	703.316	0.014	NM_025450	-1.60	***Mrps17***	mitochondrial ribosomal protein S17	0006412 // translation // inferred from electronic annotation///0006810 // transport // inferred
1642.731	998.7895	0.050	NM_026572	-1.60	***Gcsh***	glycine cleavage system protein H (aminomethyl carrier)	0006546 // glycine catabolic process // inferred from electronic annotation
109.1562	68.12119	0.017	XM_908231///XM_89	-1.60	***Lrrc51***	leucine rich repeat containing 51	0005515 // protein binding // inferred from electronic annotation
87.10313	53.09328	0.029	XR_032922///NM_02	-1.60	***Ppcs///EG667114***	phosphopantothenoylcysteine synthetase///predicted gene, EG667114	
1018.958	629.4298	0.023	NM_026631	-1.60	***Nhp2***	NHP2 ribonucleoprotein homolog (yeast)	0042254 // ribosome biogenesis // inferred from electronic annotation///0031118 // rRNA pseu
177.6615	108.6985	0.022	NM_028836	-1.60	***Ctbs***	chitobiase, di-N-acetyl-	0008152 // metabolic process // inferred from electronic annotation///0005975 // carbohydrate
860.6187	525.7906	0.006	NM_146165	-1.60	***Jtv1***	JTV1 gene	0006412 // translation // inferred from electronic annotation
3141.626	1913.345	0.018	NM_023202	-1.61	***Ndufa7***	NADH dehydrogenase (ubiquinone) 1 alpha subcomplex, 7 (B14.5a) (Ndufa	0042773 // ATP synthesis coupled electron transport // inferred from electronic annotation///00
2050.234	1245.538	0.031	NM_019745	-1.61	***Pdcd10***	programmed cell death 10	0006915 // apoptosis // inferred from electronic annotation
408.1969	252.0951	0.006	NM_001142681///NM	-1.61	***Chid1***	chitinase domain containing 1	0005975 // carbohydrate metabolic process // inferred from electronic annotation///0006032 //
605.7777	366.1319	0.048	NM_133679	-1.62	***Cryzl1***	crystallin, zeta (quinone reductase)-like 1	0055114 // oxidation reduction // inferred from electronic annotation///0008152 // metabolic p
118.8187	71.68528	0.014	NM_001039534	-1.62	***Pstk***	phosphoseryl-tRNA kinase	0006412 // translation // inferred from electronic annotation///0006355 // regulation of transcr
53.58475	33.25836	0.038	NM_007755	-1.62	***Cpeb1***	cytoplasmic polyadenylation element binding protein 1	0007130 // synaptonemal complex assembly // inferred from mutant phenotype///0006417 // r
737.2197	437.0958	0.043	NM_009069	-1.62	***Rit1***	Ras-like without CAAX 1	0007264 // small GTPase mediated signal transduction // inferred from electronic annotation
373.2211	226.2109	0.013	NM_026448	-1.63	***Klhl7***	kelch-like 7 (Drosophila)	0005515 // protein binding // inferred from electronic annotation
1017.697	614.6252	0.013	NM_175277	-1.63	***Bola3***	bolA-like 3 (E. coli)	
576.665	347.6554	0.015	NM_026452	-1.63	***Coq9***	coenzyme Q9 homolog (yeast)	0006744 // ubiquinone biosynthetic process // inferred from electronic annotation
218.0706	130.0511	0.013	NM_029629	-1.64	***Fahd2a***	fumarylacetoacetate hydrolase domain containing 2A	0008152 // metabolic process // inferred from electronic annotation
1010.362	599.4307	0.033	NM_025798	-1.64	***Hint3***	histidine triad nucleotide binding protein 3	0016787 // hydrolase activity // inferred from electronic annotation///0003824 // catalytic activ
7552.051	4567.932	0.004	NM_028618///NM_1	-1.64	***Dmkn***	dermokine	0030154 // cell differentiation // inferred from electronic annotation
446.1181	266.0846	0.029	NM_026526	-1.64	***N6amt2***	N-6 adenine-specific DNA methyltransferase 2 (putative)	0032259 // methylation // inferred from electronic annotation
4272.006	2531.228	0.044	NM_025983	-1.64	***Atp5e***	ATP synthase, H+ transporting, mitochondrial F1 complex, epsilon subunit	0015992 // proton transport // inferred from electronic annotation///0015986 // ATP synthesis
49.65157	29.80688	0.023	NM_144943	-1.64	***Cd207***	CD207 antigen	0005529 // sugar binding // inferred from electronic annotation///0005488 // binding // inferre
126.8392	75.34242	0.025	XM_001473278///NM	-1.64	***LOC100044896///Sccp***	saccharopine dehydrogenase (putative)///similar to Saccharopine dehydro	0055114 // oxidation reduction // inferred from electronic annotation///0008152 // metabolic p
102.178	61.38092	0.008	NM_009544	-1.65	***Zfp105***	zinc finger protein 105	0008270 // zinc ion binding // inferred from electronic annotation///0003676 // nucleic acid bin
644.2593	377.8097	0.030	NM_001033297	-1.65	***Gm561***	gene model 561, (NCBI)	
654.8452	384.9943	0.050	NM_027925	-1.65	***Trnau1ap***	tRNA selenocysteine 1 associated protein 1	0006412 // translation // inferred from electronic annotation///0001514 // selenocysteine incor
560.3851	332.4734	0.031	NM_207625///NM_0	-1.66	***Acsl4***	acyl-CoA synthetase long-chain family member 4	0008152 // metabolic process // inferred from electronic annotation///0019217 // regulation of
43.55812	25.82665	0.001	XR_002074///XR_033	-1.66	***LOC674137///LOC6768***	similar to RT1 class I histocompatibility antigen, AA alpha chain precursor//	0002474 // antigen processing and presentation of peptide antigen via MHC class I // inferred fr
362.2102	211.3004	0.014	NM_001012400	-1.67	***AU022252***	expressed sequence AU022252	
140.0045	82.49772	0.034	NM_001080820///NM	-1.67	***Cass4***	Cas scaffolding protein family member 4	0007155 // cell adhesion // inferred from electronic annotation
305.6148	178.4218	0.024	NM_001004190	-1.67	***Zfp560***	zinc finger protein 560	0006355 // regulation of transcription, DNA-dependent // inferred from electronic annotation//
1170.233	669.1027	0.045	NM_001031808	-1.67	***Mrpl41***	mitochondrial ribosomal protein L41	0006915 // apoptosis // inferred from electronic annotation///0007049 // cell cycle // inferred f
641.6351	378.3987	0.006	NM_026392///NM_0	-1.68	***Tmem70***	transmembrane protein 70	0033615 // mitochondrial proton-transporting ATP synthase complex assembly // inferred from
765.8442	447.9453	0.043	NM_018820	-1.68	***Sertad1***	SERTA domain containing 1	0030308 // negative regulation of cell growth // not recorded///0006355 // regulation of transcr
2987.745	1744.462	0.009	NM_012021	-1.68	***Prdx5***	peroxiredoxin 5	0055114 // oxidation reduction // inferred from electronic annotation///0045454 // cell redox h
328.4195	194.028	0.012	NM_001130149///NM	-1.68	***LOC100045148///Rnas***	ribonuclease III, nuclear///similar to ribonuclease III, nuclear	0006396 // RNA processing // inferred from electronic annotation
223.5362	127.8515	0.036	NR_003946///NM_02	-1.68	***Hyi***	hydroxypyruvate isomerase homolog (E. coli)	0008903 // hydroxypyruvate isomerase activity // inferred from electronic annotation///001685
145.3545	86.19986	0.033	NM_022986	-1.69	***Irak1bp1***	interleukin-1 receptor-associated kinase 1 binding protein 1	0007249 // I-kappaB kinase/NF-kappaB cascade // inferred from direct assay///0006355 // regul
138.8898	80.59638	0.018	NM_001082412///NM	-1.69	***Mcart6***	mitochondrial carrier triple repeat 6	0006810 // transport // inferred from electronic annotation
477.323	278.0555	0.007	NM_019752	-1.70	***Htra2***	HtrA serine peptidase 2	0006915 // apoptosis // inferred from electronic annotation///0008629 // induction of apoptosi
237.1729	136.5268	0.027	XM_001473278///NM	-1.70	***LOC100044896///Sccp***	saccharopine dehydrogenase (putative)///similar to Saccharopine dehydro	0055114 // oxidation reduction // inferred from electronic annotation///0008152 // metabolic p
158.613	91.81495	0.009	NM_029612	-1.70	***Slamf9***	SLAM family member 9	0016021 // integral to membrane // inferred from electronic annotation///0016020 // membrar
23.96309	13.79724	0.041	NM_001077353///NM	-1.70	***Gsta3***	glutathione S-transferase, alpha 3	0008152 // metabolic process // inferred from electronic annotation
455.092	262.7985	0.002	NM_025554	-1.71	***Polr2e***	polymerase (RNA) II (DNA directed) polypeptide E	0006350 // transcription // inferred from electronic annotation
497.4266	284.8073	0.001	NR_003518///XR_035	-1.72	***D030013I16Rik///Pisd***	phosphatidylserine decarboxylase, pseudogene 1///RIKEN cDNA D030013I	0008654 // phospholipid biosynthetic process // inferred from electronic annotation
320.0594	180.3527	0.026	NM_172254///NM_0	-1.72	***Dph3***	DPH3 homolog (KTI11, S. cerevisiae)	0051099 // positive regulation of binding // inferred from sequence or structural similarity///00
36.50731	20.76711	0.038	NM_134160	-1.72	***Mcoln3***	mucolipin 3	0006811 // ion transport // inferred from electronic annotation///0006810 // transport // inferr
1482.056	832.7785	0.025	NM_145517	-1.72	***Ormdl1***	ORM1-like 1 (S. cerevisiae)	0006508 // proteolysis // inferred from electronic annotation
758.034	439.0158	0.039	NM_138721	-1.73	***Lsm10***	U7 snRNP-specific Sm-like protein LSM10	0006398 // histone mRNA 3'-end processing // traceable author statement///0008380 // RNA sp
637.9747	361.2487	0.001	NR_003518///XR_035	-1.74	***D030013I16Rik///Pisd***	phosphatidylserine decarboxylase, pseudogene 1///RIKEN cDNA D030013I	0008654 // phospholipid biosynthetic process // inferred from electronic annotation
341.7427	194.705	0.003	NM_029985	-1.74	***Lrrc42***	leucine rich repeat containing 42	
1848.543	1059.303	0.018	XM_001481167///XM	-1.74	***Tmem181///Dynlt1d/***	transmembrane protein 181///dynein light chain Tctex-type 1D///dynein li	0007017 // microtubule-based process // inferred from direct assay
219.636	121.295	0.032	NM_032003	-1.74	***Enpp5***	ectonucleotide pyrophosphatase/phosphodiesterase 5	0009166 // nucleotide catabolic process // not recorded///0008152 // metabolic process // infe

[ICR, PBS](	[ICR, SUL](p		RefSeq Transcript ID	FC [PBS:SFN]	Gene Symbol	Gene Title	Gene Ontology Biological Process
1129.348	644.199	0.006	NM_053119	-1.74	***Echs1***	enoyl Coenzyme A hydratase, short chain, 1, mitochondrial	0008152 // metabolic process // inferred from electronic annotation///0006629 // lipid metabol
2764.42	1578.652	0.018	XM_001481167///XM	-1.75	***Dynlt1d///Dynlt1///EN***	dynein light chain Tctex-type 1D///dynein light chain Tctex-type 1///predic	0007017 // microtubule-based process // inferred from direct assay
20.48614	11.37225	0.015	NM_173422	-1.75	***Colec10***	collectin sub-family member 10	0009790 // embryonic development // non-traceable author statement///0006952 // defense re
392.2964	216.2384	0.032	NM_011765///NM_0	-1.75	***BC018101///Zfp97***	zinc finger protein 97///cDNA sequence BC018101	0006355 // regulation of transcription, DNA-dependent // inferred from electronic annotation//
257.9928	146.3981	0.040	NM_023196///NM_1	-1.76	***Pla2g12a***	phospholipase A2, group XIIA	0006644 // phospholipid metabolic process // inferred from electronic annotation///0016042 //
85.22612	48.56184	0.014	NM_027208	-1.76	***Bdh2***	3-hydroxybutyrate dehydrogenase, type 2	0055114 // oxidation reduction // inferred from electronic annotation///0008152 // metabolic p
100.7416	55.61209	0.034	NM_025476	-1.76	***Fam82b***	family with sequence similarity 82, member B	0005488 // binding // inferred from electronic annotation
684.1805	374.7329	0.020	NM_016763	-1.77	***Hsd17b10***	hydroxysteroid (17-beta) dehydrogenase 10	0055114 // oxidation reduction // inferred from electronic annotation///0008152 // metabolic p
75.45448	41.3073	0.031	NM_008281///NM_0	-1.77	***Hpn***	hepsin	0016049 // cell growth // inferred from electronic annotation///0006508 // proteolysis // inferre
312.5625	175.3697	0.004	NM_133768	-1.78	***Asl***	argininosuccinate lyase	0000053 // argininosuccinate metabolic process // inferred from electronic annotation///00196
668.3716	362.148	0.036	NM_008403	-1.78	***Itgb1bp1***	integrin beta 1 binding protein 1	0030155 // regulation of cell adhesion // not recorded///0007160 // cell-matrix adhesion // infe
219.8164	119.9206	0.018	NM_027175	-1.78	***Ndufaf1***	NADH dehydrogenase (ubiquinone) 1 alpha subcomplex, assembly factor 1	0008137 // NADH dehydrogenase (ubiquinone) activity // inferred from electronic annotation
311.9056	167.5919	0.030	NM_026816	-1.78	***Gtf2f2***	general transcription factor IIF, polypeptide 2	0045944 // positive regulation of transcription from RNA polymerase II promoter // inferred fro
153.3364	84.44883	0.011	NM_010210	-1.78	***Fhit***	fragile histidine triad gene	0009117 // nucleotide metabolic process // traceable author statement///0006260 // DNA repli
123.403	69.23351	0.050	NM_182783	-1.80	***Fam167b***	family with sequence similarity 167, member B	
130.9017	70.01032	0.023	NM_016746///NM_0	-1.80	***Ccnc***	cyclin C	0006355 // regulation of transcription, DNA-dependent // inferred from electronic annotation//
181.0848	96.50521	0.029	NM_026908	-1.80	***Cab39l***	Calcium binding protein 39-like, mRNA (cDNA clone MGC:27972 IMAGE:35	0005488 // binding // inferred from electronic annotation
143.07	76.97679	0.029	NM_008521	-1.80	***Ltc4s***	leukotriene C4 synthase	0006691 // leukotriene metabolic process // inferred from electronic annotation///0006691 // l
57.41704	31.49134	0.016	NM_013542	-1.81	***Gzmb***	granzyme B	0006915 // apoptosis // inferred from electronic annotation///0019835 // cytolysis // inferred fr
922.3467	501.9294	0.000	NM_008655	-1.81	***Gadd45b***	Growth arrest and DNA-damage-inducible 45 beta, mRNA (cDNA clone MG	0051726 // regulation of cell cycle // inferred from direct assay///0006915 // apoptosis // inferr
342.9352	184.2806	0.005	NM_023175	-1.82	***Nit2***	nitrilase family, member 2	0006807 // nitrogen compound metabolic process // inferred from electronic annotation
465.5807	248.5234	0.017	NM_025351	-1.82	***Chchd6***	coiled-coil-helix-coiled-coil-helix domain containing 6	
2220.494	1160.979	0.037	NM_028260	-1.82	***Immp1l***	IMP1 inner mitochondrial membrane peptidase-like (S. cerevisiae)	0008236 // serine-type peptidase activity // inferred from electronic annotation///0016787 // h
2157.829	1148.805	0.029	NM_026752	-1.82	***Zfyve21***	zinc finger, FYVE domain containing 21	0046872 // metal ion binding // inferred from electronic annotation///0008270 // zinc ion bindi
60.59875	32.02939	0.024	NM_001038654///NM	-1.83	***Slc16a3***	solute carrier family 16 (monocarboxylic acid transporters), member 3	0015711 // organic anion transport // inferred from electronic annotation///0006810 // transpo
65.25829	35.06831	0.001	NM_019686	-1.84	***Cib2***	calcium and integrin binding family member 2	0007229 // integrin-mediated signaling pathway // inferred from electronic annotation
101.9652	54.32904	0.015	NM_023900	-1.84	***Plekhj1***	pleckstrin homology domain containing, family J member 1	0005515 // protein binding // inferred from electronic annotation
348.9084	184.2331	0.010	NM_026981	-1.84	***Dtwd1***	DTW domain containing 1	
20.37019	10.75001	0.005	NM_013930	-1.86	***Aass***	aminoadipate-semialdehyde synthase	0055114 // oxidation reduction // inferred from electronic annotation///0008152 // metabolic p
418.7346	221.8955	0.000	NM_028053	-1.87	***Tmem38b***	transmembrane protein 38B///Transmembrane protein 38B (Tmem38b), n	0006813 // potassium ion transport // inferred from electronic annotation///0006811 // ion tran
313.7644	164.9038	0.025	NM_026465	-1.88	***Ncrna00117***	non-protein coding RNA 117	
775.4144	398.2403	0.023	NM_025654	-1.89	***Rdm1***	RAD52 motif 1	0003677 // DNA binding // inferred from electronic annotation///0003723 // RNA binding // infe
280.8091	144.5645	0.007	NM_016918	-1.90	***Nudt5***	nudix (nucleoside diphosphate linked moiety X)-type motif 5	0006139 // nucleobase, nucleoside, nucleotide and nucleic acid metabolic process // inferred fro
195.6902	102.7243	0.036	NM_019487	-1.91	***Hebp2***	heme binding protein 2	0005737 // cytoplasm // inferred from direct assay///0005737 // cytoplasm // inferred from ele
95.06571	48.2388	0.024	NM_028051///NM_0	-1.91	***Slc39a5***	solute carrier family 39 (metal ion transporter), member 5	0006829 // zinc ion transport // traceable author statement///0030001 // metal ion transport //
92.20453	46.38475	0.019	NM_013754	-1.91	***Insl6***	insulin-like 6	0005179 // hormone activity // inferred from electronic annotation
77.01664	39.70833	0.019	NM_011196	-1.92	***Ptger3***	prostaglandin E receptor 3 (subtype EP3)	0015701 // bicarbonate transport // inferred from mutant phenotype///0007165 // signal transd
58.40881	28.69086	0.037	NM_011760	-1.94	***Zfp54***	zinc finger protein 54	0006355 // regulation of transcription, DNA-dependent // inferred from electronic annotation//
134.536	64.73121	0.033	XM_194572///NM_00	-1.96	***Etohi1***	ethanol induced 1	0006355 // regulation of transcription, DNA-dependent // inferred from electronic annotation//
1034.297	528.4978	0.046	NM_008536	-1.96	***Tm4sf1***	transmembrane 4 superfamily member 1	0005887 // integral to plasma membrane // inferred from electronic annotation///0016021 // in
611.846	311.8536	0.033	NM_009222	-1.97	***Snap23***	synaptosomal-associated protein 23	0006810 // transport // inferred from electronic annotation///0015031 // protein transport // in
106.3862	50.90015	0.025	NM_029942	-2.00	***Prelid2***	PRELI domain containing 2	
222.2006	105.0448	0.035	NM_025429	-2.03	***Serpinb1a***	serine (or cysteine) peptidase inhibitor, clade B, member 1a	0042176 // regulation of protein catabolic process // inferred from physical interaction
75.25092	36.97152	0.027	NM_009014	-2.04	***Rad51l1***	RAD51-like 1 (S. cerevisiae)	0006974 // response to DNA damage stimulus // inferred from electronic annotation///0006310
657.9487	321.7397	0.007	NM_026347	-2.06	***Iah1***	isoamyl acetate-hydrolyzing esterase 1 homolog (S. cerevisiae)	0016042 // lipid catabolic process // inferred from electronic annotation///0006629 // lipid met
217.3756	99.474	0.021	NM_010893	-2.08	***Neu1***	neuraminidase 1	0008152 // metabolic process // inferred from electronic annotation
45.13906	21.32257	0.002	XR_002074///XR_033	-2.10	***LOC674137///LOC6768***	similar to RT1 class I histocompatibility antigen, AA alpha chain precursor//	0002474 // antigen processing and presentation of peptide antigen via MHC class I // inferred fr
433.5234	191.1205	0.044	NM_175095	-2.12	***Commd2***	COMM domain containing 2	0005515 // protein binding // inferred from electronic annotation
119.4844	54.20826	0.024	NM_023523	-2.13	***Pecr***	peroxisomal trans-2-enoyl-CoA reductase	0055114 // oxidation reduction // inferred from electronic annotation///0008152 // metabolic p
25.80614	11.38525	0.032	NM_010391	-2.14	***H2-Q10***	histocompatibility 2, Q region locus 10	0002474 // antigen processing and presentation of peptide antigen via MHC class I // inferred fr
899.5041	416.2256	0.022	NM_025273	-2.18	***Pcbd1***	pterin 4 alpha carbinolamine dehydratase/dimerization cofactor of hepato	0043496 // regulation of protein homodimerization activity // inferred from direct assay///0051
58.36729	26.84178	0.011	NM_018746///NM_0	-2.19	***Itih4***	inter alpha-trypsin inhibitor, heavy chain 4	0030212 // hyaluronan metabolic process // inferred from electronic annotation
509.9585	221.1738	0.047	NM_007994	-2.27	***Fbp2***	fructose bisphosphatase 2	0005975 // carbohydrate metabolic process // inferred from electronic annotation///0006094 //
175.9481	76.97847	0.030	NM_029556	-2.29	***Clybl***	citrate lyase beta like	0006725 // cellular aromatic compound metabolic process // inferred from electronic annotatio
45.35812	17.94804	0.050	NM_020049	-2.35	***Slc6a14***	solute carrier family 6 (neurotransmitter transporter), member 14	0006865 // amino acid transport // inferred from electronic annotation///0006836 // neurotran
147.2613	61.84474	0.037	NM_009437	-2.36	***Tst***	thiosulfate sulfurtransferase, mitochondrial	0008272 // sulfate transport // inferred from electronic annotation
887.5344	359.9771	0.018	NM_027299	-2.37	***Degs2***	degenerative spermatocyte homolog 2 (Drosophila), lipid desaturase	0055114 // oxidation reduction // inferred from electronic annotation///0006629 // lipid metab
283.5951	114.1662	0.012	NM_033612	-2.38	***cela1***	chymotrypsin-like elastase family, member 1	0006508 // proteolysis // inferred from electronic annotation
295.7775	128.9315	0.047	NM_030004	-2.40	***Cryl1***	crystallin, lambda 1	0055114 // oxidation reduction // inferred from electronic annotation///0008152 // metabolic p
98.13062	39.13979	0.048	NM_031198	-2.47	***Tcfec***	transcription factor EC	0006355 // regulation of transcription, DNA-dependent // inferred from electronic annotation//

[ICR, PBS](	[ICR, SUL](p		RefSeq Transcript ID	FC [PBS:SFN]	Gene Symbol	Gene Title	Gene Ontology Biological Process
959.7819	372.2889	0.026	NM_001102468///NM	-2.48	***Calml4***	calmodulin-like 4	0005509 // calcium ion binding // inferred from electronic annotation
82.68385	30.72629	0.015	NM_008964	-2.54	***Ptger2***	prostaglandin E receptor 2 (subtype EP2)	0042127 // regulation of cell proliferation // inferred from genetic interaction///0007165 // sign
237.3564	96.25427	0.028	NM_025393	-2.58	***S100a14***	S100 calcium binding protein A14	0005509 // calcium ion binding // inferred from electronic annotation
297.5703	110.8488	0.030	NM_183136	-2.68	***Spink8***	serine peptidase inhibitor, Kazal type 8	0004866 // endopeptidase inhibitor activity // inferred from electronic annotation///0004867 //
159.78	55.80623	0.039	NM_007443	-2.72	***Ambp***	alpha 1 microglobulin/bikunin	0018298 // protein-chromophore linkage // inferred from electronic annotation///0006810 // tr
102.0721	34.39528	0.035	NM_030601///NM_0	-2.75	***Clca1///Clca2***	chloride channel calcium activated 1///chloride channel calcium activated	0006915 // apoptosis // inferred from direct assay///0006821 // chloride transport // inferred fr
236.1969	86.81435	0.046	NM_007428	-2.76	***Agt***	angiotensinogen (serpin peptidase inhibitor, clade A, member 8)	0001999 // renal response to blood flow during renin-angiotensin regulation of systemic arterial
190.6407	62.39538	0.045	NM_026680	-2.86	***Golt1a***	golgi transport 1 homolog A (S. cerevisiae)	0006810 // transport // inferred from electronic annotation///0016192 // vesicle-mediated tran
277.9861	94.95822	0.014	NM_054098	-2.88	***Steap4***	STEAP family member 4	0045444 // fat cell differentiation // inferred from expression pattern///0055114 // oxidation re
80.82946	25.35468	0.025	NM_030703	-2.92	***Cpn1***	carboxypeptidase N, polypeptide 1	0006508 // proteolysis // inferred from electronic annotation
1170.563	416.8855	0.014	NM_010373	-2.95	***Gzme***	granzyme E	0019835 // cytolysis // inferred from electronic annotation///0006508 // proteolysis // inferred
135.4999	48.5519	0.030	NM_177372	-2.99	***Dna2***	DNA replication helicase 2 homolog (yeast)	0006260 // DNA replication // inferred from electronic annotation
1166.445	422.6965	0.041	NM_133900	-3.02	***Psph***	phosphoserine phosphatase	0008152 // metabolic process // inferred from electronic annotation///0008652 // amino acid b
124.829	43.1871	0.025	NM_010371	-3.05	***Gzmc***	granzyme C	0019835 // cytolysis // inferred from electronic annotation///0006508 // proteolysis // inferred
235.968	70.08734	0.028	NM_030601///NM_0	-3.14	***Clca1///Clca2***	chloride channel calcium activated 1///chloride channel calcium activated	0006915 // apoptosis // inferred from direct assay///0006821 // chloride transport // inferred fr
468.7179	147.8166	0.018	NM_008969	-3.15	***Ptgs1***	prostaglandin-endoperoxide synthase 1	0042127 // regulation of cell proliferation // inferred from genetic interaction///0006979 // resp
624.2955	165.2511	0.045	NM_009393	-3.47	***Tnnc1***	troponin C, cardiac/slow skeletal	0060048 // cardiac muscle contraction // inferred from electronic annotation///0055010 // vent
510.1474	144.6265	0.008	NM_010374	-3.59	***Gzmf***	granzyme F	0019835 // cytolysis // inferred from electronic annotation///0006508 // proteolysis // inferred
3432.359	973.1182	0.039	NM_008557	-3.74	***Fxyd3***	FXYD domain-containing ion transport regulator 3	0006811 // ion transport // inferred from electronic annotation///0006810 // transport // inferr
76.90684	18.04546	0.048	NM_031197	-3.74	***Slc2a2***	solute carrier family 2 (facilitated glucose transporter), member 2	0015758 // glucose transport // inferred from electronic annotation///0055085 // transmembra
155.0606	44.29022	0.040	XM_001475511///NM	-3.77	***Mosc1///LOC1000459***	MOCO sulphurase C-terminal domain containing 1///similar to MOSC dom	0055114 // oxidation reduction // inferred from electronic annotation
65.23593	15.47273	0.015	NM_009996	-3.79	***Cyp24a1***	cytochrome P450, family 24, subfamily a, polypeptide 1	0042359 // vitamin D metabolic process // inferred from mutant phenotype///0055114 // oxida
867.3882	233.5265	0.004	NM_008341	-3.86	***Igfbp1***	insulin-like growth factor binding protein 1	0001558 // regulation of cell growth // inferred from electronic annotation
769.3154	204.8184	0.012	NM_010375	-3.89	***Gzmg***	granzyme G	0019835 // cytolysis // inferred from electronic annotation///0006508 // proteolysis // inferred
541.9498	110.5021	0.045	NM_010361	-3.94	***Gstt2***	glutathione S-transferase, theta 2	0006749 // glutathione metabolic process // not recorded///0006749 // glutathione metabolic p
1153.052	308.8997	0.014	NM_010372	-4.11	***Gzmd***	granzyme D	0019835 // cytolysis // inferred from electronic annotation///0006508 // proteolysis // inferred
125.4495	26.23067	0.006	NM_008185	-4.43	***Gstt1***	glutathione S-transferase, theta 1	0006749 // glutathione metabolic process // inferred from direct assay
135.9316	18.1094	0.047	NM_019395	-5.20	***Fbp1***	fructose bisphosphatase 1	0005975 // carbohydrate metabolic process // inferred from electronic annotation///0006094 /,
289.3531	43.62996	0.049	NM_019545	-5.37	***Hao3***	hydroxyacid oxidase (glycolate oxidase) 3	0055114 // oxidation reduction // inferred from electronic annotation///0008152 // metabolic p
201.1406	24.84246	0.000	NM_010701	-7.84	***Lect1***	leukocyte cell derived chemotaxin 1	0007275 // multicellular organismal development // inferred from electronic annotation///0045
475.6654	25.90099	0.030	NM_025658	-13.01	***Ms4a4d***	membrane-spanning 4-domains, subfamily A, member 4D	0007165 // signal transduction // inferred from electronic annotation
498.221	31.61247	0.000	XR_032349///NM_01	-16.14	***H2-L///LOC636948///***	histocompatibility 2, D region locus 1///similar to H-2 class I histocompatib	0051289 // protein homotetramerization // inferred from direct assay///0006952 // defense res
2475.117	17.70803	0.001	NM_021475	-109.27	***Adamdec1***	ADAM-like, decysin 1	0006508 // proteolysis // inferred from electronic annotation

Table S6. Prenatal sulforaphane-modulated genes in *Nrf2* $^{-/-}$ placenta (T Test unpaired p-value P <= 0.05)

>=1.5-fold 367 genes

RIKEN cDNA and predicted genes excluded. Fold Change (FC): Blue (-) - fold lower in SFN than PBS, Red - fold higher in SFN than PBS.

Genes altered by SFN in common in *Nrf2* -WT and -KO placenta.

[Nrf2-KO, PBS](	[Nrf2-KO, SFN]	p	RefSeq Transcript ID	FC PBS:SFN	Gene Symbol	Gene Title	Gene Ontology
29.654598	139.6766	0.002	NM_010701///XM_0065	4.71	***Lect1***	leukocyte cell derived chemotaxin 1	0001886 // endothelial cell morphogenesis // inferred from direct assay///0001937 // negative re
100.55591	290.45444	0.001	NM_001081212	2.89	***Irs2***	insulin receptor substrate 2	0002053 // positive regulation of mesenchymal cell proliferation // inferred from genetic interact
45.319675	106.36528	0.003	NM_008634///XM_0065	2.35	***Map1b***	microtubule-associated protein 1B	0000226 // microtubule cytoskeleton organization // inferred from electronic annotation///0001
92.82944	209.91774	0.006	NM_009706///XM_0065	2.26	***Arhgap5***	Rho GTPase activating protein 5	0002053 // positive regulation of mesenchymal cell proliferation // inferred from mutant phenot
1987.436	4424.0757	0.002	NM_013724///XM_0065	2.23	***Nrk***	Nik related kinase	0006468 // protein phosphorylation // not recorded///0007256 // activation of JNKK activity // in
17.585354	38.495815	0.000	NM_011670	2.19	***Uchl1***	ubiquitin carboxy-terminal hydrolase L1	0002931 // response to ischemia // inferred from mutant phenotype///0006508 // proteolysis //
107.7787	232.66904	0.007	NM_133249///XM_0065	2.16	***Ppargc1b***	peroxisome proliferative activated receptor, gamma, coactivator 1 beta	0001503 // ossification // inferred from mutant phenotype///0006351 // transcription, DNA-tem
86.64082	184.87636	0.003	NM_001159603///NM_(	2.13	***Pum1***	pumilio RNA-binding family member 1	0006417 // regulation of translation // inferred from electronic annotation
198.3993	410.63806	0.006	NM_001033167///XM_0	2.07	***Slc22a23***	solute carrier family 22, member 23	0006810 // transport // inferred from electronic annotation///0006811 // ion transport // inferre
183.62709	373.68777	0.003	NM_001081395///XM_0	2.04	***Amotl1***	angiomotin-like 1	0003365 // establishment of cell polarity involved in ameboidal cell migration // inferred from mu
236.45654	466.55453	0.002	NM_013813///XM_0065	1.97	***Epb4.1l3***	erythrocyte protein band 4.1-like 3	0002175 // protein localization to paranode region of axon // inferred from mutant phenotype//
224.81752	438.51678	0.001	NM_023598///XM_0065	1.95	***Arid5b***	AT rich interactive domain 5B (MRF1-like)	0001822 // kidney development // inferred from mutant phenotype///0006351 // transcription, I
31.940857	61.276947	0.006	NM_019496	1.92	***Ammecr1***	Alport syndrome, mental retardation, midface hypoplasia and elliptocytosis chromosomal region gene 1	
78.55106	150.46126	0.002	NM_027707///NM_2012	1.92	***Nipbl***	Nipped-B homolog (Drosophila)	0000122 // negative regulation of transcription from RNA polymerase II promoter // not recorded
236.47023	451.6383	0.005	NM_001033422///XM_0	1.91	***Thoc2***	THO complex 2	0000902 // cell morphogenesis // inferred from mutant phenotype///0001824 // blastocyst deve
150.81491	284.48697	0.001	NM_033563///XM_0064	1.89	***Klf7***	Kruppel-like factor 7 (ubiquitous)	0006351 // transcription, DNA-templated // inferred from electronic annotation///0006355 // re
546.5554	1022.07465	0.001	NM_009320///XM_0065	1.87	***Slc6a6***	solute carrier family 6 (neurotransmitter transporter, taurine), member 6	0001762 // beta-alanine transport // inferred from direct assay///0003333 // amino acid transme
259.63635	485.46533	0.009	NM_175229///XM_0065	1.87	***Srrm2***	serine/arginine repetitive matrix 2	0006397 // mRNA processing // inferred from electronic annotation///0008380 // RNA splicing //
212.05516	392.16537	0.002	NM_026666///XM_0065	1.85	***Ubn1***	ubinuclein 1	0006336 // DNA replication-independent nucleosome assembly // not recorded///0010923 // ne
275.78058	508.23175	0.007	NM_001166584///NM_(	1.84	***Tead1***	TEA domain family member 1	0003143 // embryonic heart tube morphogenesis // inferred from genetic interaction///0006351
466.95718	856.161	0.000	NM_010638	1.83	***Klf9***	Kruppel-like factor 9	0006351 // transcription, DNA-templated // inferred from electronic annotation///0006355 // re
213.48146	389.41882	0.001	NM_008448	1.82	***Kif5b***	kinesin family member 5B	0006839 // mitochondrial transport // traceable author statement///0007017 // microtubule-bas
268.03125	487.97748	0.002	NM_001110826///NM_(	1.82	***Ddx6***	DEAD (Asp-Glu-Ala-Asp) box polypeptide 6	0006200 // ATP catabolic process // inferred from electronic annotation///0008152 // metabolic
297.5103	537.5202	0.003	NM_009367///XM_0064	1.81	***Tgfb2***	transforming growth factor, beta 2	0000902 // cell morphogenesis // not recorded///0001501 // skeletal system development // inf
116.7948	208.21835	0.001	NM_010559///XM_0065	1.78	***Il6ra***	interleukin 6 receptor, alpha	0008284 // positive regulation of cell proliferation // not recorded///0010536 // positive regulati
325.14688	579.4461	0.001	NM_023275	1.78	***Rhoj***	ras homolog gene family, member J	0006184 // GTP catabolic process // inferred from sequence or structural similarity///0007165 //
146.65102	259.05176	0.002	NM_008306///XM_0065	1.77	***Ndst1***	N-deacetylase/N-sulfotransferase (heparan glucosaminyl) 1	0000165 // MAPK cascade // inferred from mutant phenotype///0000271 // polysaccharide biosy
60.01903	105.209496	0.003	NM_177628///XM_0065	1.75	***Fam167a***	family with sequence similarity 167, member A	
117.15238	205.0593	0.004	NM_001037221///NM_(	1.75	***Samd4***	sterile alpha motif domain containing 4	0006417 // regulation of translation // inferred from electronic annotation///0017148 // negative
86.87219	151.83783	0.000	NM_153542///XM_0065	1.75	***Lrrc20***	leucine rich repeat containing 20	0005515 // protein binding // inferred from electronic annotation
290.54257	505.5732	0.006	NM_011595	1.74	***Timp3***	tissue inhibitor of metalloproteinase 3	0007269 // neurotransmitter secretion // inferred from electronic annotation///0008152 // meta
221.74672	385.83133	0.003	NM_010415	1.74	***Hbegf***	heparin-binding EGF-like growth factor	0001525 // angiogenesis // non-traceable author statement///0001832 // blastocyst growth // tr
60.15734	104.51836	0.003	NM_001025572///XM_0	1.74	***Ankrd12***	ankyrin repeat domain 12	0005515 // protein binding // inferred from electronic annotation
445.643	771.2975	0.007	NM_009149	1.73	***Glg1***	golgi apparatus protein 1	0010955 // negative regulation of protein processing // inferred from genetic interaction///0010
242.51205	417.68323	0.001	NM_010612	1.72	***Kdr***	kinase insert domain protein receptor	0001525 // angiogenesis // inferred from electronic annotation///0001541 // ovarian follicle deve
29.918562	51.37372	0.001	NM_133829///NM_178(	1.72	***Mfsd6***	major facilitator superfamily domain containing 6	0002376 // immune system process // inferred from electronic annotation///0055085 // transme
153.77072	263.67532	0.004	NM_022018///XM_0065	1.71	***Fam129a***	family with sequence similarity 129, member A	0001933 // negative regulation of protein phosphorylation // inferred from mutant phenotype//
138.10278	236.51712	0.004	NM_027045///NM_0284	1.71	***Ccser2***	coiled-coil serine rich 2	0001578 // microtubule bundle formation // inferred from direct assay
148.1518	253.30557	0.009	NM_001024955///NM_(	1.71	***Pik3r1***	phosphatidylinositol 3-kinase, regulatory subunit, polypeptide 1 (p85 alpha)	0001934 // positive regulation of protein phosphorylation // not recorded///0001953 // negative
48.484287	82.86876	0.001	NM_009179///XM_0065	1.71	***St3gal2***	ST3 beta-galactoside alpha-2,3-sialyltransferase 2	0006486 // protein glycosylation // inferred from direct assay///0006486 // protein glycosylation
31.0933	52.967587	0.002	NM_010216///XM_0065	1.70	***Figf***	c-fos induced growth factor	0001525 // angiogenesis // inferred from electronic annotation///0007275 // multicellular organi
686.9339	1166.1697	0.006	NM_001286944///NM_(	1.70	***Jund***	jun D proto-oncogene	0002076 // osteoblast development // inferred from genetic interaction///0006351 // transcripti
29.975426	50.643105	0.002	NM_172806///XM_0065	1.69	***Btbd7***	BTB (POZ) domain containing 7	0007275 // multicellular organismal development // inferred from electronic annotation///00606
646.1344	1089.8871	0.000	NM_001035532///NM_(	1.69	***Akap2***	A kinase (PRKA) anchor protein 2	0007015 // actin filament organization // inferred from direct assay///0007178 // transmembran
163.66634	275.7104	0.003	NM_001289895///NM_(	1.68	***Tns1***	tensin 1	0007044 // cell-substrate junction assembly // inferred from mutant phenotype///0010761 // fib
55.71182	93.38969	0.008	NM_026083///NM_0273	1.68	***Zc3h13***	zinc finger CCCH type containing 13	0005515 // protein binding // inferred from electronic annotation///0046872 // metal ion binding
470.6281	787.2458	0.006	NM_175260///XM_0065	1.67	***Myh10***	myosin, heavy polypeptide 10, non-muscle	0000281 // mitotic cytokinesis // inferred from mutant phenotype///0000281 // mitotic cytokine
74.19704	123.851494	0.007	NM_001081345	1.67	***Chd2***	chromodomain helicase DNA binding protein 2	0006351 // transcription, DNA-templated // inferred from electronic annotation///0006355 // re
183.03212	304.75214	0.007	NM_173863///XM_0065	1.67	***Crtc3***	CREB regulated transcription coactivator 3	0006351 // transcription, DNA-templated // inferred from electronic annotation///0006355 // re
258.22665	429.66727	0.002	NM_018868///XM_0064	1.66	***Nop58***	NOP58 ribonucleoprotein	0006608 // snRNP protein import into nucleus // not recorded///0042254 // ribosome biogenesis
139.32092	231.62354	0.003	NM_001085472///NM_(	1.66	***Acin1***	apoptotic chromatin condensation inducer 1	0006397 // mRNA processing // inferred from electronic annotation///0006915 // apoptotic proc
783.2815	1300.5016	0.003	NM_019764///XM_0065	1.66	***Amotl2***	angiomotin-like 2	0016055 // Wnt signaling pathway // inferred from electronic annotation///0035329 // hippo sig
123.759094	205.37727	0.006	NM_019827///XM_0065	1.66	***Gsk3b***	glycogen synthase kinase 3 beta	0000320 // re-entry into mitotic cell cycle // inferred from direct assay///0001837 // epithelial to
101.437164	167.71692	0.006	NM_001081290///XM_0	1.65	***Prrc2c***	proline-rich coiled-coil 2C	0002244 // hematopoietic progenitor cell differentiation // inferred from genetic interaction
91.79746	151.67366	0.003	NM_175375///NM_2012	1.65	***Ankhd1///Eif4ebp3///Masl***	ankyrin repeat and KH domain containing 1///eukaryotic translation initiation factor 4E binding protein 3///m	0006417 // regulation of translation // inferred from electronic annotation///0017148 // negative
51.99767	85.33215	0.002	NM_001081308///NM_(	1.64	***Taok3***	TAO kinase 3	0000165 // MAPK cascade // not recorded///0000186 // activation of MAPKK activity // not recor
24.316162	39.710747	0.003	NM_007731///XM_0065	1.63	***Col13a1***	collagen, type XIII, alpha 1	0001503 // ossification // inferred from electronic annotation///0001763 // morphogenesis of a b
138.46472	226.06395	0.001	NM_009125///XM_0065	1.63	***Atxn2***	ataxin 2	0002091 // negative regulation of receptor internalization // not recorded///0021702 // cerebell
114.34457	186.64891	0.002	NM_172627///XM_0065	1.63	***Pggt1b***	protein geranylgeranyltransferase type I, beta subunit	0008152 // metabolic process // inferred from electronic annotation///0008284 // positive regul
852.8893	1386.0612	0.007	NM_010515	1.63	***Igf2r***	insulin-like growth factor 2 receptor	0001889 // liver development // not recorded///0006810 // transport // inferred from electronic
100.08163	162.52353	0.000	NM_016670///NR_0274!	1.62	***Pknox1***	Pbx/knotted 1 homeobox	0001525 // angiogenesis // inferred from mutant phenotype///0006355 // regulation of transcrip
30.79821	49.81701	0.004	NM_009022	1.62	***Aldh1a2***	aldehyde dehydrogenase family 1, subfamily A2	0001523 // retinoid metabolic process // traceable author statement///0001568 // blood vessel d
1311.2756	2117.584	0.002	NM_001198949///NM_(	1.61	***Ralbp1***	ralA binding protein 1	0006200 // ATP catabolic process // not recorded///0006810 // transport // not recorded///0006
436.66263	704.89874	0.005	NM_001286630///NM_(	1.61	***Brd4***	bromodomain containing 4	0000083 // regulation of transcription involved in G1/S transition of mitotic cell cycle // not recor
85.60844	137.84404	0.007	NM_009274///XM_0065	1.61	***Srpk2***	serine/arginine-rich protein specific kinase 2	0000245 // spliceosomal complex assembly // inferred from direct assay///0000245 // spliceosor
128.34276	206.58994	0.008	NM_008139	1.61	***Gnaq***	guanine nucleotide binding protein, alpha q polypeptide	0001501 // skeletal system development // inferred from mutant phenotype///0001508 // action
81.630295	131.2164	0.004	NM_001037136///NM_1	1.61	***Agap1***	ArfGAP with GTPase domain, ankyrin repeat and PH domain 1	0006184 // GTP catabolic process // not recorded///0006810 // transport // inferred from electro

[Nrf2-KO, PBS](	[Nrf2-KO, SFN]	p	RefSeq Transcript ID	FC PBS:SFN	Gene Symbol	Gene Title	Gene Ontology
227.59846	364.3591	0.008	NM_001122594///XM_0	1.60	***Phlpp2***	PH domain and leucine rich repeat protein phosphatase 2	0006470 // protein dephosphorylation // not recorded///0008152 // metabolic process // inferre
46.61532	74.62542	0.002	NM_001081342///NM_1	1.60	***Gpr133***	G protein-coupled receptor 133	0007166 // cell surface receptor signaling pathway // inferred from electronic annotation///0007
175.33382	280.0718	0.010	NM_008608	1.60	***Mmp14***	matrix metallopeptidase 14 (membrane-inserted)	0001503 // ossification // inferred from genetic interaction///0001525 // angiogenesis // inferred
169.87717	270.61682	0.009	NM_001252313///NM_(	1.59	***Ncor1***	nuclear receptor co-repressor 1	0000122 // negative regulation of transcription from RNA polymerase II promoter // inferred fror
112.20864	178.59636	0.004	NM_001256019///NM_(	1.59	***Bmp8a***	bone morphogenetic protein 8a	0001503 // ossification // inferred from electronic annotation///0001649 // osteoblast differenti
76.61679	121.892235	0.002	NM_145469///XM_0065	1.59	***Nipal2***	NIPA-like domain containing 2	0015693 // magnesium ion transport // inferred from electronic annotation
139.39969	221.6585	0.002	NM_001127382///NM_(	1.59	***Rbm47***	RNA binding motif protein 47	0002244 // hematopoietic progenitor cell differentiation // inferred from mutant phenotype
154.64069	245.34033	0.009	NM_001122989///NM_1	1.59	***Cdc14b***	CDC14 cell division cycle 14B	0006281 // DNA repair // inferred from electronic annotation///0006470 // protein dephosphory
267.5616	424.32977	0.005	NM_183186///XM_0065	1.59	***Foxn3***	forkhead box N3	0006351 // transcription, DNA-templated // inferred from electronic annotation///0006355 // re
50.520676	80.09842	0.004	NM_019588///XM_0065	1.59	***Plce1***	phospholipase C, epsilon 1	0000187 // activation of MAPK activity // not recorded///0006629 // lipid metabolic process // in
919.9455	1456.9144	0.010	NM_139149///XM_0065	1.58	***Fus***	fused in sarcoma	0000166 // nucleotide binding // inferred from electronic annotation///0003676 // nucleic acid b
157.5852	249.31615	0.002	NM_138593	1.58	***Larp7***	La ribonucleoprotein domain family, member 7	0000122 // negative regulation of transcription from RNA polymerase II promoter // inferred fror
33.56851	52.969547	0.002	NM_001081267	1.58	***Rsf1***	remodeling and spacing factor 1	0006334 // nucleosome assembly // not recorded///0006338 // chromatin remodeling // not rec
214.85323	338.34213	0.004	NM_029735///XM_0064	1.57	***Eprs***	glutamyl-prolyl-tRNA synthetase	0006412 // translation // inferred from electronic annotation///0006417 // regulation of translat
52.963245	83.01658	0.003	NM_133187///XM_0065	1.57	***Fam198b***	family with sequence similarity 198, member B	0000139 // Golgi membrane // inferred from electronic annotation///0005794 // Golgi apparatus
302.86575	473.274	0.006	NM_030886///NM_198(	1.56	***Ankrd17***	ankyrin repeat domain 17	0001955 // blood vessel maturation // inferred from mutant phenotype///0007492 // endoderm
1870.5076	2920.1187	0.005	NM_001093764///NM_(	1.56	***Myadm***	myeloid-associated differentiation marker	0001933 // negative regulation of protein phosphorylation // not recorded///0010629 // negativ
223.12712	347.63193	0.005	NM_001081203///XM_0	1.56	***Sbno1***	sno, strawberry notch homolog 1 (Drosophila)	0006355 // regulation of transcription, DNA-templated // inferred from electronic annotation///(
72.766525	113.172195	0.001	NM_001109975///NM_1	1.56	***Synpo***	synaptopodin	0030865 // cortical cytoskeleton organization // non-traceable author statement///0032233 // p
386.83777	601.2774	0.002	NM_001113545///NM_(	1.55	***Lima1***	LIM domain and actin binding 1	0030835 // negative regulation of actin filament depolymerization // not recorded///0031529 //
373.1615	578.8367	0.001	NM_001136240///NM_1	1.55	***Chdh***	choline dehydrogenase	0006066 // alcohol metabolic process // inferred from electronic annotation///0016117 // carote
204.4373	316.90594	0.003	NM_001205173///NM_1	1.55	***Iffo2***	intermediate filament family orphan 2	0005198 // structural molecule activity // inferred from electronic annotation
807.49396	1251.062	0.004	NM_010890	1.55	***Nedd4***	neural precursor cell expressed, developmentally down-regulated 4	0000122 // negative regulation of transcription from RNA polymerase II promoter // inferred fror
343.139	531.0809	0.004	NM_010560	1.55	***Il6st***	interleukin 6 signal transducer	0005977 // glycogen metabolic process // inferred from mutant phenotype///0006642 // triglyce
268.3768	415.23944	0.010	NM_009621	1.55	***Adamts1***	a disintegrin-like and metallopeptidase (reprolysin type) with thrombospondin type 1 motif, 1	0001542 // ovulation from ovarian follicle // inferred from mutant phenotype///0001822 // kidne
150.48409	232.54872	0.005	NM_001197321///NM_(	1.55	***Foxp1***	forkhead box P1	0000122 // negative regulation of transcription from RNA polymerase II promoter // inferred fror
219.75166	338.68494	0.001	NM_001252282///NM_(	1.54	***Ogdh***	oxoglutarate (alpha-ketoglutarate) dehydrogenase (lipoamide)	0006091 // generation of precursor metabolites and energy // inferred from sequence or structu
61.148697	94.17461	0.009	NM_001081477///XM_0	1.54	***Brwd3***	bromodomain and WD repeat domain containing 3	0007010 // cytoskeleton organization // not recorded///0008360 // regulation of cell shape // no
2680.0466	4123.3755	0.002	NM_008302	1.54	***Hsp90ab1***	heat shock protein 90 alpha (cytosolic), class B member 1	0001890 // placenta development // inferred from mutant phenotype///0006457 // protein foldi
119.55461	183.71754	0.003	NM_010124	1.54	***Eif4ebp2***	eukaryotic translation initiation factor 4E binding protein 2	0006417 // regulation of translation // inferred from electronic annotation///0006446 // regulati
520.86615	798.30365	0.007	NM_001291212///NM_(	1.53	***Myo18a***	myosin XVIIIA	0007030 // Golgi organization // not recorded///0008152 // metabolic process // inferred from e
52.324303	79.931274	0.007	NM_008449	1.53	***Kif5c***	kinesin family member 5C	0006355 // regulation of transcription, DNA-templated // inferred from electronic annotation///(
37.96012	57.955303	0.006	NM_145430///XM_0065	1.53	***Fam222b***	family with sequence similarity 222, member B	
156.09846	238.06563	0.007	NM_138657///XM_0065	1.53	***Socs7***	suppressor of cytokine signaling 7	0008286 // insulin receptor signaling pathway // inferred from mutant phenotype///0009968 // r
828.1501	1261.3615	0.009	NM_010123	1.52	***Eif3a***	eukaryotic translation initiation factor 3, subunit A	0001731 // formation of translation preinitiation complex // inferred from electronic annotation/
178.14966	270.939	0.006	NM_011213///XM_0065	1.52	***Ptprf***	protein tyrosine phosphatase, receptor type, F	0001960 // negative regulation of cytokine-mediated signaling pathway // not recorded///00064
476.58768	722.8335	0.003	NM_001081251///NM_(	1.52	***Pbrm1***	polybromo 1	0001890 // placenta development // inferred from mutant phenotype///0001974 // blood vessel
201.07	304.79947	0.008	NM_001161362///NM_1	1.52	***Ppp2r3a***	protein phosphatase 2, regulatory subunit B'', alpha	0001754 // eye photoreceptor cell differentiation // not recorded///0007525 // somatic muscle c
187.8261	284.68112	0.005	NM_198303///XM_0064	1.52	***Eif5b***	eukaryotic translation initiation factor 5B	0006184 // GTP catabolic process // inferred from electronic annotation///0006412 // translatior
76.257645	115.54268	0.001	NM_001081350///NM_(	1.52	***Nol8***	nucleolar protein 8	0006260 // DNA replication // not recorded///0006364 // rRNA processing // not recorded///003
103.30395	155.71524	0.007	NM_018785///XM_0064	1.51	***Prpf40a***	PRP40 pre-mRNA processing factor 40 homolog A (yeast)	0006397 // mRNA processing // inferred from electronic annotation///0007010 // cytoskeleton o
135.39261	203.5406	0.002	NM_011894///XM_0065	1.50	***Sh3bp5***	SH3-domain binding protein 5 (BTK-associated)	0006469 // negative regulation of protein kinase activity // inferred from direct assay///0006469
402.42963	604.7821	0.009	NM_024452///XM_0065	1.50	***Luzp1***	leucine zipper protein 1	0003281 // ventricular septum development // inferred from mutant phenotype///0021503 // ne
228.43387	152.31956	0.007	NM_030731///XM_0065	-1.50	***Trim23***	tripartite motif-containing 23	0006184 // GTP catabolic process // not recorded///0006471 // protein ADP-ribosylation // not r
712.6971	474.83206	0.006	NM_001110159///NM_(	-1.50	***Nxt1***	NTF2-related export protein 1	0006405 // RNA export from nucleus // inferred from direct assay///0006611 // protein export fr
1723.9908	1145.6034	0.004	NM_001039368///NM_(	-1.50	***LOC100862456///Polr2k***	DNA-directed RNA polymerases I, II, and III subunit RPABC4-like///polymerase (RNA) II (DNA directed) polype	0006351 // transcription, DNA-templated // inferred from electronic annotation///0006360 // tra
171.257	113.783104	0.006	NM_001253685///NM_(	-1.51	***Dnajc12***	DnaJ (Hsp40) homolog, subfamily C, member 12	
74.34266	49.38743	0.001	NM_029942	-1.51	***Prelid2***	PRELI domain containing 2	0015914 // phospholipid transport // not recorded
30.163132	19.97883	0.001	NM_001080967///NM_2	-1.51	***Tmsb15b2///Tmsb15l///Tm***	thymosin beta 15b2///thymosin beta 15b like///thymosin beta 15b1	0007010 // cytoskeleton organization // inferred from electronic annotation///0030036 // actin c
1187.7072	784.6771	0.003	NM_016905	-1.51	***Galk1***	galactokinase 1	0005975 // carbohydrate metabolic process // inferred from electronic annotation///0006012 //
603.0483	397.4583	0.007	NR_024069	-1.52	***Smim4***	small integral membrane protein 4	0005739 // mitochondrion // inferred from direct assay///0016020 // membrane // inferred from
368.23785	242.32144	0.005	NM_001256100///NM_(	-1.52	***Mtif3***	mitochondrial translational initiation factor 3	0001732 // formation of translation initiation complex // not recorded///0006412 // translation /
6832.678	4477.093	0.000	NM_139198	-1.53	***Plac8***	placenta-specific 8	0008284 // positive regulation of cell proliferation // inferred from direct assay///0009409 // resp
291.448	190.22641	0.007	NM_183275///XM_0065	-1.53	***Tefm***	transcription elongation factor, mitochondrial	0006119 // oxidative phosphorylation // not recorded///0006259 // DNA metabolic process // nc
502.25217	324.9984	0.003	NM_007705///XM_0065	-1.55	***Cirbp***	cold inducible RNA binding protein	0006950 // response to stress // inferred from electronic annotation///0009409 // response to cc
415.6489	263.10162	0.008	NM_008776	-1.58	***Pafah1b3***	platelet-activating factor acetylhydrolase, isoform 1b, subunit 3	0006629 // lipid metabolic process // inferred from electronic annotation///0007283 // spermatc
291.0483	183.60161	0.010	NM_027264///XM_0065	-1.59	***Zfp715***	zinc finger protein 715	0006355 // regulation of transcription, DNA-templated // inferred from electronic annotation
366.44653	230.66063	0.008	NR_029382///NR_02981	-1.59	***Mir17hg///Mir92-1***	Mir17 host gene 1 (non-protein coding)///microRNA 92-1	0001701 // in utero embryonic development // inferred from genetic interaction///0002329 // pr
115.03816	72.38381	0.000	NM_001113401///NM_(	-1.59	***Eaf2***	ELL associated factor 2	0006351 // transcription, DNA-templated // inferred from electronic annotation///0006355 // re
26.701725	16.769487	0.004	NM_001136059///NM_(	-1.59	***Cyp1a1***	cytochrome P450, family 1, subfamily a, polypeptide 1	0001666 // response to hypoxia // inferred from electronic annotation///0001889 // liver develo
62.30257	38.85401	0.001	NM_178417	-1.60	***Zfp867***	zinc finger protein 867	0006355 // regulation of transcription, DNA-templated // inferred from electronic annotation
7075.0547	4396.1826	0.004	NM_009760	-1.61	***Bnip3***	BCL2/adenovirus E1B interacting protein 3	0001666 // response to hypoxia // not recorded///0006309 // apoptotic DNA fragmentation // in
18.792166	11.65461	0.001	NM_027054	-1.61	***Cst12***	cystatin 12	0010466 // negative regulation of peptidase activity // inferred from electronic annotation///001
30.946371	19.152197	0.010	NM_007749	-1.62	***Cox7c***	cytochrome c oxidase subunit VIIc	1902600 // hydrogen ion transmembrane transport // inferred from electronic annotation
541.4956	334.2564	0.009	NM_001039562///XM_0	-1.62	***Ankrd37***	ankyrin repeat domain 37	0005515 // protein binding // inferred from physical interaction
219.12213	134.98166	0.007	NM_011943///XM_0065	-1.62	***Map2k6***	mitogen-activated protein kinase kinase 6	0000165 // MAPK cascade // not recorded///0000165 // MAPK cascade // inferred from physical
252.87946	155.67854	0.001	NM_009944	-1.62	***Cox7a1***	cytochrome c oxidase subunit VIIa 1	0055114 // oxidation-reduction process // inferred from sequence or structural similarity///1902
337.33157	207.07092	0.001	NM_175016///XM_0065	-1.63	***Alkbh2***	alkB, alkylation repair homolog 2 (E. coli)	0006281 // DNA repair // inferred from electronic annotation///0006307 // DNA dealkylation inv
66.03724	40.408676	0.004	NM_001285853///NM_(	-1.63	***Matk***	megakaryocyte-associated tyrosine kinase	0006468 // protein phosphorylation // inferred from electronic annotation///0016310 // phosph
153.09377	93.59858	0.003	NM_146159	-1.64	***Haus3***	HAUS augmin-like complex, subunit 3	0006260 // DNA replication // inferred from electronic annotation///0006261 // DNA-dependent
289.38388	175.44807	0.002	NM_018827///XM_0065	-1.65	***Crlf1***	cytokine receptor-like factor 1	0001657 // ureteric bud development // inferred from expression pattern///0008284 // positive

[Nrf2-KO, PBS](	[Nrf2-KO, SFN]	p	RefSeq Transcript ID	FC PBS:SFN	Gene Symbol	Gene Title	Gene Ontology
942.3501	557.65533	0.004	NM_026080///XM_0065	-1.69	***Mrps24***	mitochondrial ribosomal protein S24	0006412 // translation // inferred from sequence or structural similarity
105.44037	60.948776	0.008	NM_001271729///NM_(	-1.73	***Tk1***	thymidine kinase 1	0001889 // liver development // inferred from electronic annotation///0006260 // DNA replicatio
428.62814	240.13652	0.001	NM_027106///XM_0065	-1.78	***Avpi1***	arginine vasopressin-induced 1	0000187 // activation of MAPK activity // inferred from direct assay///0007049 // cell cycle // infe
632.86444	351.0533	0.006	NM_001005223///XM_0	-1.80	***Znhit3***	zinc finger, HIT type 3	0046872 // metal ion binding // inferred from electronic annotation
67.62999	37.116673	0.001	NM_026358///NR_0281	-1.82	***Mgarp***	mitochondria localized glutamic acid rich protein	0006626 // protein targeting to mitochondrion // inferred from direct assay///0008089 // antero
129.81496	68.594696	0.003	NM_001109914	-1.89	***Apold1***	apolipoprotein L domain containing 1	0001525 // angiogenesis // inferred from electronic annotation///0001666 // response to hypoxi
267.1691	136.2508	0.004	NM_001037134///NM_(	-1.96	***Ccne2***	cyclin E2	0000079 // regulation of cyclin-dependent protein serine/threonine kinase activity // inferred fro
711.34247	361.6959	0.002	NM_030093	-1.97	***Snrnp25***	small nuclear ribonucleoprotein 25 (U11/U12)	0006397 // mRNA processing // inferred from electronic annotation///0008380 // RNA splicing //
73.92938	35.603764	0.005	NM_173869	-2.08	***Stfa2l1***	stefin A2 like 1	0010951 // negative regulation of endopeptidase activity // inferred from electronic annotation
53.3571	25.288898	0.001	NM_001289727///NM_(	-2.11	***Tmem255a***	transmembrane protein 255A	0016020 // membrane // inferred from electronic annotation///0016021 // integral component c
190.04109	88.3881	0.000	NM_025748///XM_0065	-2.15	***Adat2***	adenosine deaminase, tRNA-specific 2	0002100 // tRNA wobble adenosine to inosine editing // inferred from electronic annotation///00
202.0172	91.759605	0.002	NM_025491///NM_0283	-2.20	***Susd3***	sushi domain containing 3	0016020 // membrane // inferred from electronic annotation///0016021 // integral component c
49.01388	16.211506	0.006	XM_006528434///XM_0	-3.02	***Astx///Astx6///LOC102631***	amplified spermatogenic transcripts X encoded///amplified spermatogenic transcripts X encoded 6///uncharacterized LOC102631953///uncharacterized LOC102640642	
48.069675	15.348848	0.004	NM_009484///XM_0065	-3.13	***Uty***	ubiquitously transcribed tetratricopeptide repeat gene, Y chromosome	0001701 // in utero embryonic development // inferred from mutant phenotype///0003007 // he
